# Autoimmune diseases are associated with increased neurodegenerative and cerebrovascular risk, while systemic corticosteroid exposure shows limited neurodegenerative and modest vascular associations

**DOI:** 10.1016/j.ibneur.2026.03.010

**Published:** 2026-03-28

**Authors:** Younes Adam Tabi, Eva Christina Meyer

**Affiliations:** a Department for Neurology, University Medical Centre Kiel, Arnold-Heller-Str 3, Kiel 24105, Germany; b Department for Internal Medicine, Schön Klinik Eckernförde, Schleswiger Str. 114, Eckernförde 24340, Germany

**Keywords:** Autoimmune diseases, Alzheimer disease, Parkinson Disease, Transient ischemic, attack, Stroke, Corticosteroids

## Abstract

Systemic autoimmune diseases (AIDs), characterized by chronic peripheral inflammation and frequent vascular comorbidity, are increasingly linked to adverse central nervous system (CNS) outcomes; however, comparative evidence across diverse AIDs and clarification of the roles of vascular burden, inflammatory activity, and immunomodulatory therapy remain limited. Using the TriNetX Global Collaborative Network, we conducted a sequence of retrospective, propensity score–matched cohort experiments in adults aged 50–85 years to quantify incident Parkinson’s disease (PD), Alzheimer’s disease (AD), transient ischemic attack (TIA), and ischemic stroke across 22 AIDs and to evaluate therapy- and inflammation-stratified risk patterns. In the primary disease–control analysis (Experiment 1 A), AID diagnosis was associated with broadly elevated neurodegenerative and cerebrovascular risk, with stronger and more consistent associations for TIA and ischemic stroke than for PD and AD. To probe robustness, we repeated analyses under tighter control of baseline vascular burden (Experiment 1B: additional matching on circulatory-system diagnoses) and under treatment balancing (Experiment 1 C: additional matching on immune-suppressant exposure). Vascular matching substantially attenuated many associations, particularly for AD, whereas immune-suppressant matching did not materially erase the pervasive cerebrovascular excess. Within-disease CRP stratification (Experiment 2; low vs elevated CRP) did not yield a uniform neurodegenerative gradient but identified a disease-dependent ischemic vulnerability axis in selected inflammatory phenotypes. In treatment substudies, systemic cortisone exposure (Experiment 3) showed little association with PD/AD risk but a modest, heterogeneous increase in TIA/ischemic stroke. Medication-specific strata (Experiment 4) revealed stronger but directionally variable separations, consistent with confounding by indication and severity. Together, these findings position systemic autoimmunity as a robust marker of heightened cerebrovascular risk and a more phenotype-dependent correlate of neurodegenerative risk, supporting intensified vascular surveillance in high-risk AID populations and mechanistic work disentangling inflammation, comorbidity, and treatment.

## Introduction

Autoimmune diseases (AIDs) manifest through uncontrolled immune system responses against self-antigens which creates ongoing systemic inflammation and multi-organ damage. New evidence indicates autoimmunity plays a role in developing both neurodegenerative conditions and cerebrovascular diseases beyond its traditional organ targets. Research based on epidemiological meta-analyses has established small but important correlations between autoimmune diseases (AIDs) and Parkinson’s disease (PD) which show that patients with AIDs face a relative risk increase when compared to the general population (M. Li et al., 2023). Research has discovered multiple susceptibility loci that exist between PD and different autoimmune diseases (Witoelar et al., 2017) which supports the idea that shared immune-regulatory mechanisms may trigger PD development. Further, available evidence demonstrates that Alzheimer’s disease (AD) has a connection with autoimmunity. A research study utilizing electronic health records across multiple sites demonstrated autoimmune disorders increase AD development odds by 1.4–1.7 times (Ramey et al., 2025) with endocrine and gastrointestinal autoimmune disorders playing the most significant role. Clinical observations support this research by showing that AIDs which specifically target multiple sclerosis exhibit Mendelian randomization associations with higher Alzheimer’s disease risks while Sjögren’s shows lower risk (Yeung et al., 2022). Multiple hospital-based cohort studies have found that patients with autoimmune disorders experience a higher incidence of dementia and Alzheimer’s disease than the general population despite typical risk factor adjustment (X. Li et al., 2018). The observed relationship between chronic systemic inflammation and autoimmunity as dementia accelerators strengthens because these factors independently raise the risk of Alzheimer’s disease development. The detection of anti-neuronal autoantibodies together with glial activation in AD patients demonstrates that autoimmunity may contribute to the pathogenesis of AD (Fang et al., 2024).

Research findings demonstrate that autoimmune and inflammatory diseases lead to increased cerebrovascular risk. Research shows that rheumatoid arthritis increase stroke risk by 1.4 to 2.0 times above typical levels according to Liu et al. (Liu et al., 2021). Patients experiencing specific vasculitic conditions face the greatest risk of ischemic events when they are most recently diagnosed because their bodies undergo intense vascular inflammation (Ahn et al., 2021, Ahn et al., 2022). A comprehensive analysis of psoriasis and cardiovascular disease showed both a direct relationship between disease severity and ischemic stroke risk and proof that intense anti-inflammatory treatments can reduce this risk (Gelfand et al., 2009, Hu and Lan, 2017, Olejnik et al., 2025).

Despite accumulating evidence linking individual AIDs to single neurological endpoints, major gaps remain. First, it is unclear which autoimmune phenotypes confer the greatest risk across neurodegenerative and cerebrovascular outcomes when evaluated within a single harmonized EHR framework. Second, vascular and metabolic comorbidity is strongly enriched in several AIDs and may confound or mediate associations—particularly for stroke/TIA and potentially for dementia—necessitating robustness analyses under tighter vascular control. Third, immunomodulatory therapy is difficult to interpret in observational EHR data because treatment exposure often indexes disease severity; therefore, therapy-associated risk patterns require designs that balance baseline treatment profiles and, where possible, examine disease-specific medications. Finally, if systemic inflammation is mechanistically relevant, then within-disease gradients of inflammatory activity (e.g., CRP strata) should track neurological risk more clearly than between-disease comparisons alone. To address these gaps, we used the TriNetX Global Collaborative Network to conduct a structured series of propensity score–matched experiments across 22 AIDs and four neurological outcomes (PD, AD, TIA, ischemic stroke), including (i) primary disease–control comparisons, (ii) robustness analyses with additional matching on vascular burden and immune-suppressant exposure, and (iii) within-disease stratified analyses based on CRP and medication exposure.

## Methods

### Data source and governance

We used the TriNetX Global Collaborative Network, a federated network of de-identified electronic health records (EHRs) from large healthcare organizations (HCOs). All analyses were performed within the TriNetX environment and only aggregate counts were exported. Per TriNetX governance, data are de-identified; institutional review board (IRB) review was not required for this secondary analysis.

Across all experiments, we studied adults aged 50–85 years and performed analyses separately by sex (female, male). For each outcome, patients with the outcome on or before the index date were excluded. Follow-up began 1 day after index; where a fixed window was used (CRP analysis), outcomes were assessed through 1095 days post-index as implemented in TriNetX.

## Autoimmune conditions (22 diseases)

Autoimmune exposures were defined by ICD-10 codes in TriNetX for:

1) Chronic inflammatory demyelinating polyneuropathy (CIDP); 2) Guillain–Barré syndrome (GBS); 3) Myasthenia gravis (MG); 4) Multiple sclerosis (MS); 5) Vitiligo; 6) Rheumatoid vasculitis; 7) Graves’ disease; 8) Vasculitis (unspecified); 9) Dermatomyositis; 10) Arthropathic psoriasis; 11) Addison’s disease; 12) Psoriasis; 13) Sjögren syndrome; 14) Scleroderma; 15) Myositis; 16) Celiac disease; 17) Rheumatoid arthritis (RA); 18) Type 1 diabetes mellitus (T1DM); 19) Autoimmune thyroiditis; 20) Lupus erythematosus; 21) Ulcerative colitis (UC); and 22) Crohn’s disease.

## Cohort construction and index

### Experiment 1 (between-cohort disease–control comparisons; 22 AIDs)

For each autoimmune disease, we constructed an exposed cohort defined by the relevant ICD-10 code and a comparator cohort without that diagnosis sampled from the same network and meeting the same age and encounter criteria. Index date was the first date meeting the cohort definition (autoimmune diagnosis for exposed; qualifying visit for controls). Patients with the outcome on or before index were excluded for that outcome.

• *Experiment 1 A (primary specification):* 1:1 propensity score matching (PSM) within sex strata on age variables (as available in-platform: current age and age at index).
• *Experiment 1B (vascular-burden robustness):* repeated Experiment 1 under tighter vascular control by additionally matching on diseases of the circulatory system.
• *Experiment 1 C (treatment-balance robustness):* repeated Experiment 1 under treatment balancing by additionally matching on immune-suppressant exposure.

### Experiment 2 (within-disease CRP stratification)

Within selected autoimmune diseases, we compared patients with low CRP (≤1.0 mg/L) to those with elevated CRP (3.0–10.0 mg/L), excluding intermediate values to increase contrast, requiring CRP measurement on or after the autoimmune diagnosis. Index date was defined by the first occurrence satisfying the diagnosis-plus-CRP criterion. Outcomes were evaluated in a fixed post-index window (start 1 day after index; end 1095 days after index in the TriNetX run).

### Experiment 3 (within-disease systemic cortisone exposure)

Within each autoimmune disease cohort, we compared patients with documented systemic cortisone exposure (RxNorm 2878) to patients without cortisone exposure. Index date was the first date satisfying the autoimmune diagnosis with (exposed) or without (unexposed) the medication criterion as implemented in TriNetX. These treatment-stratified comparisons are observational and may reflect confounding by indication and disease severity.

### Experiment 4 (within-disease medication-specific strata)

To probe disease-specific therapy patterns beyond a non-specific steroid proxy, we performed within-disease analyses comparing medication-exposed vs unexposed strata for standard-of-care agents (e.g., mesalamine in ulcerative colitis), using index definitions that required medication occurrence on or after the autoimmune diagnosis.

## Propensity score matching and effect estimation

Within each sex stratum, we performed 1:1 nearest-neighbor PSM without replacement using experiment-specific covariate sets: Experiment 1 A matched on age; Experiment 1B on age and circulatory-system diagnoses; Experiment 1 C on age and immune-suppressant exposure; and Experiments 2–4 on age, age at index, and (where available) concurrent immune-suppressant use to ensure baseline clinical comparability within disease cohorts. Covariate balance was confirmed within the platform prior to outcome analyses. TriNetX provided risks, risk differences, risk ratios, and odds ratios; we used post-match event counts to compute sex-specific RRs and Wald 95% CIs in Python, pooled across sexes by inverse-variance fixed-effects meta-analysis of log(RR). Zero-event pairs in both arms were not pooled or interpreted.

## Outcomes

Incident neurological outcomes were defined by first post-index occurrence of: Parkinson’s disease (G20), Alzheimer’s disease (G30), transient ischemic attack (G45), and ischemic stroke (I63).

## Platform estimates

For each matched comparison, TriNetX provided the number at risk, number of events, risks (cumulative incidence), risk differences, risk ratios (RR), and odds ratios (OR). These platform outputs were used to verify event counts and to cross-check our independent synthesis.

## Independent synthesis for figures and tables

From the post-match counts, we computed effect sizes in Python:

• Sex-specific RRs:
  $$\mathrm{RR}=(a/n_{1})/(c/n_{0})$$
  with
  $$\mathrm{SE}\left[\log\mathrm{RR}\right]=\;\sqrt{\frac{1}{a}-\frac{1}{n_{1}}+\frac{1}{c}-\frac{1}{n_{0}}}$$
  Wald 95% CIs were formed on the log scale and exponentiated.
• Sex difference:
  $$∆=\log{\mathrm{RR}}_{\mathrm{Female}}-\log{\mathrm{RR}}_{\mathrm{Male}}$$
  with
  $$\mathrm{SE}\left[∆\right]=\sqrt{{\mathrm{SE}}_{F}^{2}+{\mathrm{SE}}_{M}^{2}}$$
• Pooled effects across sexes (fixed effect):
  Inverse-variance weighting of sex-specific $\log\mathrm{RR}\left(\mathrm{weights}w=\frac{1}{{\mathrm{SE}}^{2}}\right)$ to obtain pooled logRR, then exponentiation to RR with 95% CI.
• Zero-cell handling:

Estimates used observed counts without continuity corrections; cells with zero events in both arms were not pooled/interpreted.

## Results

### Experiment 1 A: Baseline Risk Across 22 Autoimmune Diseases

**Fig. 1:**
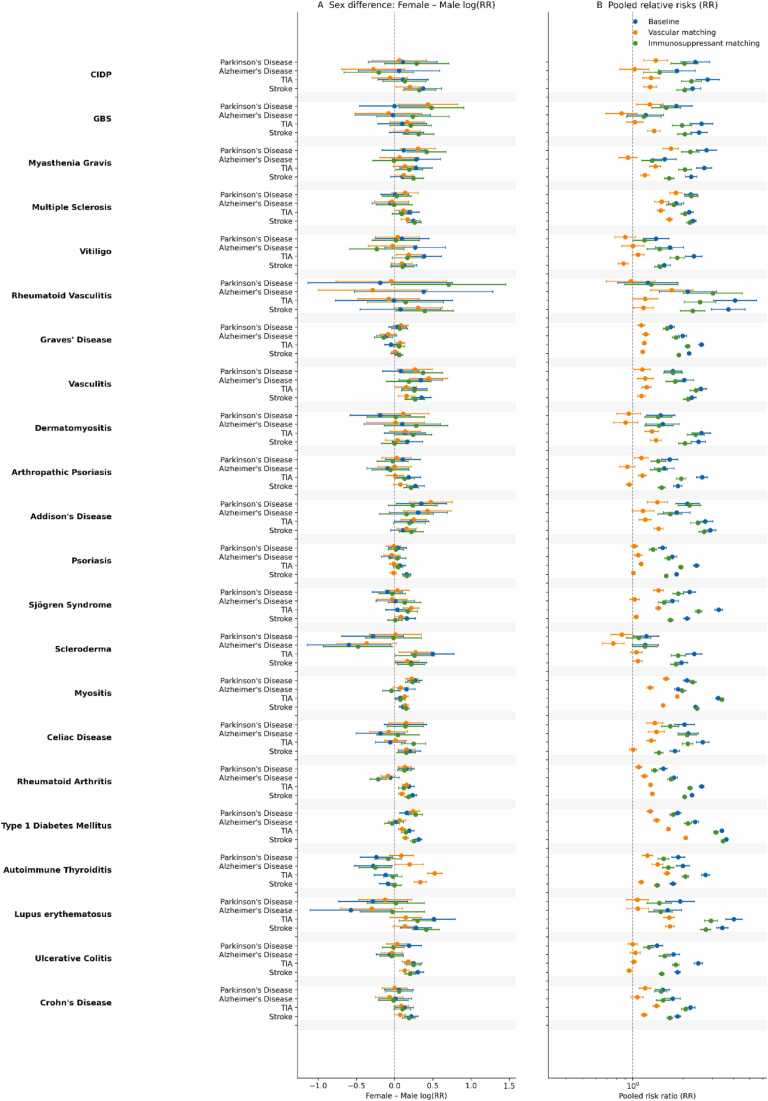
**| Autoimmune disease and neurovascular/neurodegenerative outcomes. Panel A (left):** Female–male differences in log(RR) were small overall but typically favored slightly higher relative risks in females (median female:male RR ratio range 0.99–1.28). **Panel B (right):** Across autoimmune diseases, pooled fixed-effect RRs were generally elevated at baseline and after immunosuppressant matching, with the strongest increases for TIA and stroke (median RR ≈ 2.6/2.2 at baseline and ≈2.1/2.0 after immunosuppressant matching) and more modest elevations for Parkinson’s and Alzheimer’s disease (median RR ≈ 1.6–1.8). Vascular/circulatory matching markedly attenuated associations toward the null (median RR ≈ 1.1–1.3 across outcomes).

Across 22 autoimmune conditions, we observed consistent elevation of risk for PD, AD, TIA, and ischemic stroke compared with matched controls (Fig. 1 **– blue marker, detailed results in Appendix - EXPERIMENT 1 A**). Pooled RRs (fixed effect, across sexes) were > 1 for 85/88 disease–outcome pairs (97%); only three pairs had 95% CIs including 1 (GBS–AD, scleroderma–AD, rheumatoid vasculitis–PD).

By outcome, the across-condition median pooled RR was 1.78 for PD (range 1.07–2.76), 1.76 for AD (1.09–2.36), 2.59 for TIA (2.00–4.09), and 2.24 for ischemic stroke (1.58–3.73). The largest effects tended to cluster in a small set of conditions. For PD, the highest pooled RRs were seen in myasthenia gravis (2.76, 95% CI 2.45–3.10), chronic inflammatory demyelinating polyneuropathy (2.37, 1.99–2.83), and multiple sclerosis (2.23, 2.04–2.43). For AD, type 1 diabetes mellitus (2.36, 2.21–2.52), celiac disease (2.14, 1.88–2.44), and rheumatoid vasculitis (2.13, 1.58–2.87) led. Cerebrovascular outcomes were uniformly stronger: for TIA, rheumatoid vasculitis (4.09, 3.23–5.18), lupus (4.03, 3.61–4.50), and type 1 diabetes (3.41, 3.26–3.57); for ischemic stroke, rheumatoid vasculitis (3.73, 3.19–4.36), type 1 diabetes (3.62, 3.48–3.76), and lupus (3.43, 3.26–3.61).

Sex differences were modest for PD and AD but pronounced for vascular outcomes. The female-to-male RR ratio (F/M) had medians of 1.07 for PD (14/22 conditions F>M; 3 significant), 0.99 for AD (8/22 F>M; 2 significant; 5 significant F<M), 1.28 for TIA (20/22 F>M; 13 significant), and 1.20 for ischemic stroke (21/22 F>M; 14 significant). Conditions with the largest female excess included, for TIA, Addison’s disease (F/M 1.52, 1.34–1.73), vitiligo (1.49, 1.29–1.72), and ulcerative colitis (1.42, 1.28–1.58), and for ischemic stroke, vitiligo (1.40, 1.28–1.54), Graves’ disease (1.35, 1.29–1.41), and Sjögren syndrome (1.32, 1.26–1.38). These patterns indicate that women with autoimmune disease experience a systematically larger *relative* elevation in cerebrovascular risk than men, even after matching.

Although the pattern in Experiment 1 A was broad and consistent, autoimmune diseases cluster strongly with vascular comorbidity, which could inflate associations with ischemic stroke/TIA and even neurodegenerative endpoints. To probe this, Experiment 1B repeated the analysis with additional propensity score matching on circulatory-system diagnoses, testing the robustness of the signal under tighter vascular control.

### Experiment 1B: Adjustment for Baseline Vascular Burden

In this follow-up analysis—using 1:1 propensity score matching on current age plus diseases of the circulatory system—the broad, uniformly elevated risk pattern from Experiment 1 A was substantially attenuated (Fig. 1 **– orange marker, detailed results in Appendix - EXPERIMENT 1B**). Across the autoimmune conditions shown here (up to autoimmune thyroiditis; lupus is truncated in the excerpt), many PD and especially AD associations moved toward the null, while cerebrovascular outcomes (TIA/ischemic stroke) generally remained modestly elevated, most consistently in women and in a smaller subset of high-risk conditions.

Neurodegenerative outcomes (PD, AD) weakened most:

• Parkinson’s disease (PD): Residual associations persisted, but were typically small-to-moderate rather than the ∼2 × effects seen previously. The clearest remaining PD signals clustered in a few conditions—particularly myasthenia gravis (male RR 1.52, female RR 2.06), multiple sclerosis (male 1.66, female 1.90), myositis (male 1.39, female 1.74), and type 1 diabetes (male 1.16, female 1.48). Several conditions were null (e.g., vitiligo both sexes; RA in males; vasculitis in males; rheumatoid vasculitis both sexes).
• Alzheimer’s disease (AD): This outcome showed the strongest shift to null. Entirely null patterns emerged for multiple conditions that were clearly positive in Experiment 1 A (e.g., CIDP, GBS, MG, Sjögren: essentially no excess AD after vascular matching). Where AD remained elevated, effect sizes were generally modest and concentrated in systemic/vascular-metabolic phenotypes: T1DM (male 1.34, female 1.43), autoimmune thyroiditis (male 1.20, female 1.46), Graves’ disease (male 1.28, female 1.17), RA (male 1.25, female 1.15), and myositis (male 1.20, female 1.30). A notable inverse association appeared for female scleroderma (RR 0.72).

Cerebrovascular outcomes persisted, but at lower magnitude than Experiment 1.

• TIA: Effects were mostly small (≈1.1–1.5), with stronger residual elevations in a few conditions—especially myositis (male 1.69, female 1.92), T1DM (male 1.55, female 1.71), autoimmune thyroiditis in females (1.80), and MS (male 1.35, female 1.52). Several pairs were null, including GBS (both sexes) and rheumatoid vasculitis (both sexes).
• Ischemic stroke: Again, most associations were modest, but a clear high-risk tier remained. The strongest residual ischemic stroke signal was T1DM (male 1.94, female 2.24). Consistent elevations persisted for myositis (male 1.39, female 1.59), MS (male 1.47, female 1.74), RA (male 1.22, female 1.35), and Addison’s disease (male 1.31, female 1.52). At the other extreme, several conditions were null or even protective, including vitiligo in males (RR 0.84), autoimmune thyroiditis in males (RR 0.87), and arthropathic psoriasis in males (RR 0.92).

The sex pattern showed that even after vascular matching, women generally retained larger relative elevations than men – most clearly for PD in MG/Addison/T1DM, and for TIA/ischemic stroke across many conditions (e.g., CIDP, GBS, MS, vasculitis, myositis, RA, T1DM, autoimmune thyroiditis). In multiple settings, male signals collapsed to null while female signals persisted (e.g., PD in GBS; several vascular outcomes in vasculitis/scleroderma).

Adding circulatory-system matching suggests that a substantial number of the Experiment 1 A associations – especially for AD and the magnitude of cerebrovascular risk – is likely mediated and/or confounded by vascular comorbidity. However, a residual, clinically relevant cerebrovascular excess remains in a narrower set of conditions (most prominently T1DM, myositis, MS, RA, Addison’s), and it appears systematically stronger in women.

Overall, Experiment 1B indicates that baseline vascular burden is an important contributor, but it does not address whether differences in immunosuppressant exposure – either as a marker of disease severity or a direct risk factor – account for the remaining excess. Experiment 1 C therefore re-matched cohorts on current age and immune suppressants to test whether the residual neurodegenerative and cerebrovascular elevations are robust to treatment balancing.

### Experiment 1 C: Adjustment for Immunosuppressant Exposure

In this third analysis, we repeated 1:1 propensity score matching across the same autoimmune conditions, now matching on current age and immune suppressants (sex-stratified cohorts; standardized differences ∼0 in the larger datasets). In contrast to Experiment 1B (where additional matching on circulatory disease substantially attenuated many associations), immune-suppressant matching did not meaningfully erase the overall signal: risk elevations for TIA and ischemic stroke remained strikingly consistent across essentially all conditions, and PD/AD associations remained broadly positive, with a smaller set of clear null patterns confined to a few disease–outcome pairs (Fig. 1 **– green marker, detailed results in Appendix - EXPERIMENT 1 C**).

A key qualitative shift now emerges as vascular outcomes are the most robust and least sensitive to this form of matching, while neurodegenerative outcomes show more heterogeneity, including several conditions where PD or AD becomes null despite persistent vascular excess – suggesting partial dissociation between neurodegenerative and cerebrovascular risk channels and arguing against immune-suppressant exposure alone as the primary driver.

### Neurodegenerative outcomes

PD was broadly elevated, with condition-specific nulls. Most conditions retained significant PD elevations after matching on immune suppressants, typically in the ∼1.3–2.5 range, with a high-risk tier including myasthenia gravis (male RR 1.93, female 2.92), CIDP (male 1.89, female 2.52), multiple sclerosis (male 2.20, female 2.26), myositis (male 1.99, female 2.53), Addison’s disease (male 1.96, female 2.48), and T1DM (male 1.57, female 2.07). In contrast, vitiligo and scleroderma were essentially null for PD in both sexes, and rheumatoid vasculitis showed no PD signal in males (RR 0.85) with a modest but significant elevation in females (RR 1.71). Gastrointestinal autoimmunity (celiac disease, ulcerative colitis, Crohn’s disease) showed modest but consistent PD elevations (roughly ∼1.2–1.8).

AD was generally positive, with a clearer “vascular–metabolic/systemic inflammation” enrichment. AD risk remained elevated across many conditions, often around ∼1.5–2.2, and was particularly prominent in T1DM (male RR 2.17, female 2.11), celiac disease (male 2.05, female 2.14), autoimmune thyroiditis (male 2.02, female 1.57), Graves’ disease (male 2.02, female 1.75), myositis (male 2.04, female 1.96), and rheumatoid arthritis (male 2.00, female 1.61). The most extreme AD signal occurred in female rheumatoid vasculitis (RR 3.01; AD not reportable in males due to sparse data).

However, several notable null/near-null AD patterns persisted even after this matching strategy – most clearly in GBS (both sexes), CIDP in females, scleroderma in females, and dermatomyositis in males – reinforcing that AD associations are not uniformly driven by treatment exposure and may concentrate in specific systemic/vascular-metabolic phenotypes.

### Cerebrovascular outcomes

TIA was uniformly elevated and often large. TIA associations were consistently significant across conditions and both sexes, with many effects in the ∼1.6–2.6 range and a clearly higher-risk subset. The strongest elevations clustered in myositis (male RR 3.25, female 3.49), T1DM (male 2.92, female 3.39), lupus (male 2.29, female 3.10), and Addison’s disease (male 2.18, female 2.65). Other repeatedly elevated conditions included vasculitis (male 2.00, female 2.60), Sjögren syndrome (male 2.14, female 2.54), dermatomyositis (male 2.04, female 2.60), autoimmune thyroiditis (∼2.06–2.11), and Graves’ disease (∼2.04–2.16).

### Ischemic stroke showed pervasive excess risk, with a pronounced high-risk tier

Ischemic stroke risk also remained robustly elevated across virtually all conditions, with many effects in the ∼1.4–2.6 range and a high-risk tier that is clinically conspicuous. The largest signals occurred in T1DM (male RR 3.11, female 3.98), lupus (male 1.97, female 2.99), Addison’s disease (male 2.37, female 2.95), myositis (male 2.21, female 2.56), and female rheumatoid vasculitis (RR 2.55; males 1.72). Multiple sclerosis also showed sustained ischemic stroke elevation, especially in females (male 1.84, female 2.39). Even conditions with weak/null PD signals (e.g., vitiligo, scleroderma) demonstrated clear TIA and ischemic stroke excess, underscoring that cerebrovascular vulnerability is widespread across autoimmune phenotypes in this matched framework.

Sex pattern showed that a recurring theme is that women often show larger relative elevations than men, most consistently for TIA and ischemic stroke, and in several conditions also for PD (e.g., MG, GBS, vasculitis, Addison’s, T1DM, myositis). Some outcomes show near-parallel male/female effects (e.g., MS for PD), while a few AD signals are stronger in men (e.g., Graves’ disease, vitiligo). Overall, the post-matching profile remains compatible with a systematic female excess in vascular relative risk, with especially prominent separation in the highest-risk phenotypes (e.g., T1DM ischemic stroke RR 3.98 in females vs 3.11 in males; lupus ischemic stroke 2.99 vs 1.97).

In summary, matching on immune suppressants (in addition to age) leaves a highly coherent residual pattern, particularly for cerebrovascular outcomes, indicating that the broad autoimmune–vascular association is unlikely to be explained primarily by immunosuppressant exposure. Instead, the data support a model in which underlying disease biology and/or correlated vascular–metabolic–inflammatory comorbidity (as highlighted by the stronger attenuation seen when matching on circulatory disease in Experiment 1B) contributes substantially to risk. Clinically, a concentrated high-risk tier – T1DM, myositis/dermatomyositis, lupus, Addison’s disease, vasculitis/rheumatoid vasculitis, and MS – continues to stand out as the most compelling target for intensified cerebrovascular surveillance and mechanistic follow-up, while PD/AD signals remain broadly positive but more phenotype-dependent.

Experiment 1 demonstrated broad autoimmune-associated excess risk for PD/AD and, more strongly, cerebrovascular outcomes, and showed that tighter control of baseline vascular comorbidity markedly attenuates many associations (especially AD). Experiment 2 therefore moved from *between-disease* comparisons to a *within-disease* test of heterogeneity, asking whether systemic inflammatory activity, operationalized by CRP strata (≤1.0 vs 3.0–10.0 mg/L) under 1:1 matching on age (and where available age at index and immune-suppressant exposure), tracks residual neurodegenerative and cerebrovascular risk within representative autoimmune phenotypes.

### Experiment 2: Risk Stratification by CRP (CRP 3–10 mg/L vs ≤1 mg/L)

**Fig. 2:**
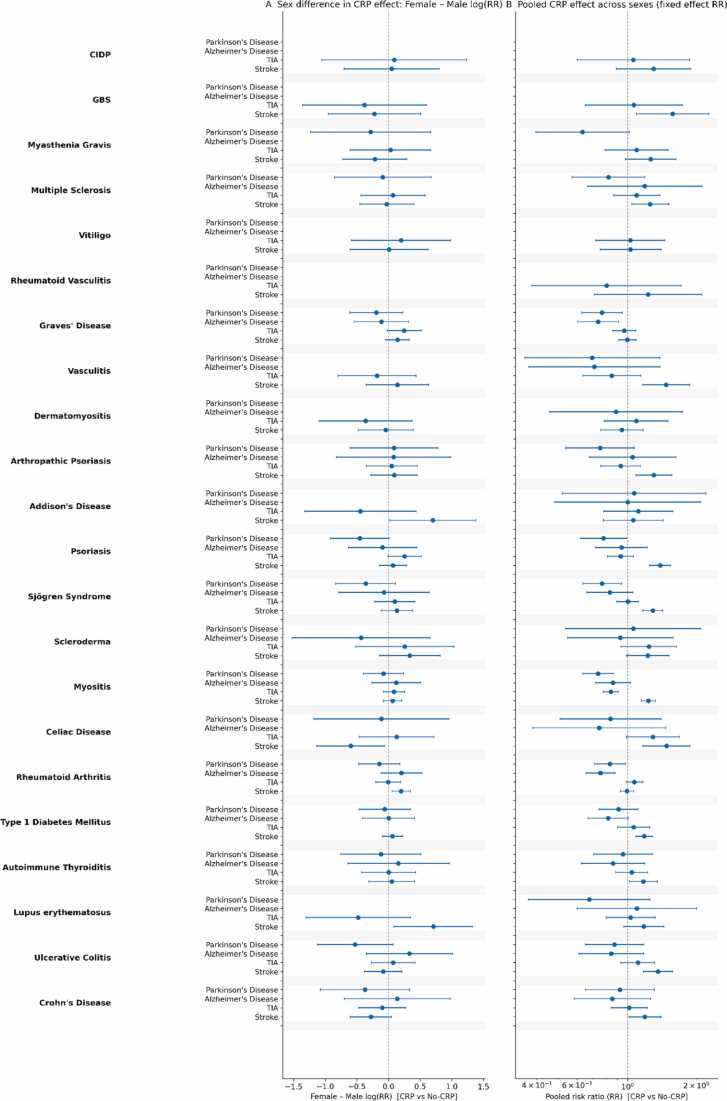
**| Within-Disease Neurodegenerative and Cerebrovascular Risk by CRP (CRP vs No-CRP)**. **Sex differences (Panel A)** were generally small: female:male CRP-associated RR ratios clustered near 1.0 for Alzheimer’s disease (median 1.01) and tended to be slightly lower in females for Parkinson’s disease (0.93) and for TIA/stroke (0.85–0.83). The largest sex divergence was observed for TIA in lupus erythematosus, indicating a stronger CRP-associated effect in males. **Pooled fixed-effect estimates across sexes (Panel B)** showed heterogeneous CRP associations, with the clearest RR> 1 gradients for cerebrovascular events and more modest, often near-null effects for Parkinson’s and Alzheimer’s disease.

CRP stratification produced a qualitatively different pattern than Experiment 1. Neurodegenerative outcomes were largely null, whereas ischemic stroke (I63) showed the clearest and most reproducible elevated-CRP gradient in a subset of inflammatory-vascular phenotypes (Fig. 2**; detailed results in Appendix – EXPERIMENT 2**). Across most diseases, PD and AD were not consistently higher in the elevated-CRP stratum; when differences emerged, they were typically modest, often female-skewed, and sometimes directionally heterogeneous.

For example, in lupus (female cohort), there was no convincing CRP separation for PD or AD, and TIA remained null. In contrast, female ischemic stroke was significantly higher with elevated CRP (RR ≈ 1.28).

High-prevalence conditions showed more selective and disease-specific patterns rather than a universal null tendency. Autoimmune thyroiditis was broadly null across PD/AD/TIA, but female stroke showed a small yet significant elevation with higher CRP (RR ≈ 1.19). Rheumatoid arthritis showed statistically significant differences for AD in males and PD in females; however, these effects were directionally lower in the elevated-CRP stratum (male AD RR ≈ 0.66; female PD RR ≈ 0.79). Type 1 diabetes mellitus showed clear and significant ischemic stroke gradients in both sexes, with higher CRP conferring higher stroke risk (males RR ≈ 1.15; females RR_CRP ≈ 1.22). Celiac disease remained largely null for PD/AD, but the ischemic analysis suggested a strong male-specific stroke signal (male RR ≈ 2.27) with a weaker trend in females (RR ≈ 1.26; female RR ≈ 1.34).

In contrast, several systemic inflammatory phenotypes showed consistent and significant excess I63 incidence in the elevated-CRP stratum. This signal was robust across sexes in dermatologic and myopathic autoimmunity: psoriasis showed clear stroke gradients in males (RR ≈ 1.34) and females (RR ≈ 1.44), and myositis showed significant gradients in males (RR ≈ 1.18) and females (RR ≈ 1.26). A similar female-predominant ischemic gradient was observed in Sjögren syndrome (RR ≈ 1.32), vasculitis (RR ≈ 1.55), and systemic sclerosis/scleroderma (female stroke RR ≈ 1.35), with arthropathic psoriasis also showing a female stroke signal (RR ≈ 1.36).

Neurologic autoimmunity showed selective effects: in multiple sclerosis, female I63 was significant with elevated CRP (RR ≈ 1.25) with no strong CRP separation for PD/AD/TIA, while in GBS, males demonstrated a marked stroke gradient (RR ≈ 1.75).

Overall, Experiment 2 indicates that mild CRP elevation does not act as a universal mediator of autoimmune–neurodegenerative associations, but it does identify a disease-dependent ischemic vulnerability axis, strongest in inflammatory-vascular phenotypes and more evident in women for several conditions –consistent with the female-skewed vascular excess noted in Experiment 1.

### Experiment 3: Impact of Systemic Cortisone Exposure

**Fig. 3:**
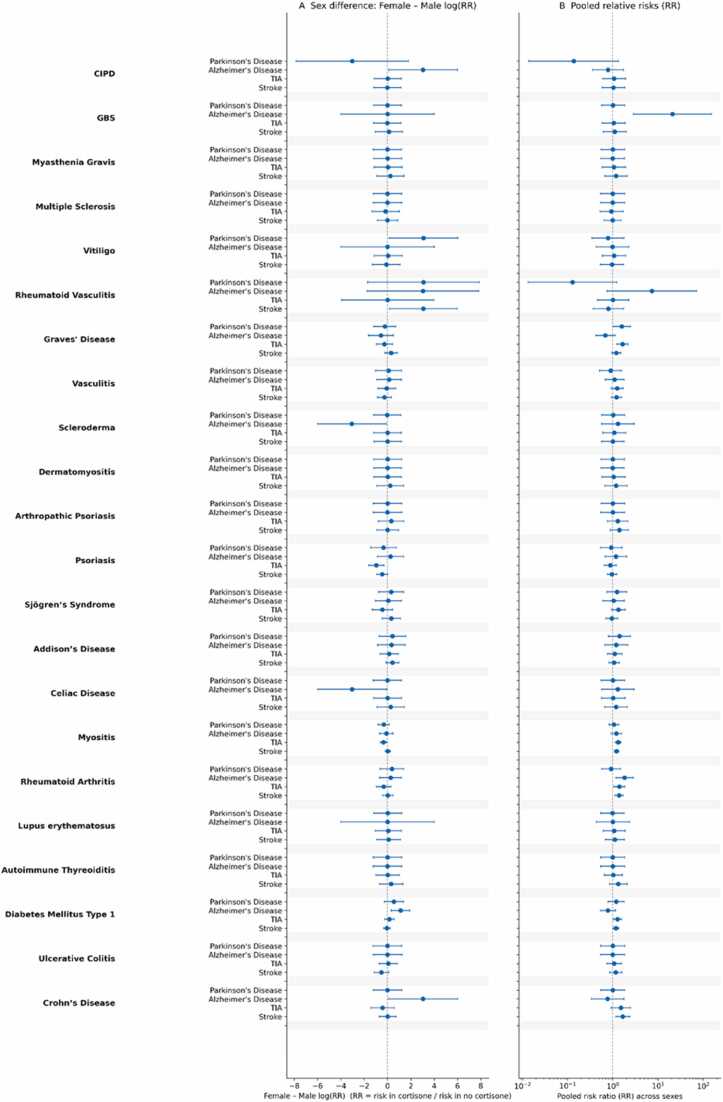
**| Cortisone exposure within autoimmune cohorts. Panel A (left).** Sex contrast is generally modest; only a few disease–outcome pairs diverge meaningfully. **Panel B (right).** Overall cortisone-related effect across sexes; where present, signals concentrate in TIA and stroke and vary by disease. Overview. Cortisone exposure shows little consistent link with Parkinson’s or Alzheimer’s but aligns with higher cerebrovascular events in a subset of diseases.

Comparing cortisone-exposed vs. unexposed patients within each autoimmune condition, associations were generally small for neurodegeneration and modest—but highly heterogeneous—for cerebrovascular disease (Fig. 3 **–detailed results in Appendix – EXPERIMENT 3**). While the across-condition median pooled RR remained near unity (∼1.00 for PD and ∼1.03 for AD), these medians mask significant, condition-specific effects that were often directionally opposed.

For Parkinson’s disease, cortisone exposure was associated with a significant reduction in incidence in several phenotypes, including female CIDP (0.0% vs. 10.9%, p = 0.001), male vitiligo (0.0% vs. 7.2%, p = 0.001), and male rheumatoid vasculitis (0.0% vs. 43.5%, p < 0.001).

Alzheimer’s disease (AD) patterns showed a similarly high degree of variance. Significant protective associations appeared in male CIDP (0.0% vs. 10.8%, p = 0.001) and male Crohn’s disease (0.0% vs. 1.2%, p = 0.002). Conversely, significant AD elevations occurred in several smaller sub-cohorts, most notably female rheumatoid vasculitis (11.8% vs. 0.0%, p = 0.001), male scleroderma (15.6% vs. 0.0%, p = 0.001), and male celiac disease (6.9% vs. 0.0%, p = 0.001). GBS appeared as a major outlier in both sexes due to zero cases occurring in the unexposed groups. Among high-prevalence conditions, rheumatoid arthritis showed a significant AD elevation in females (1.1% vs. 0.6%, p = 0.013).

Cerebrovascular endpoints showed more frequent positive shifts, though signals were concentrated in specific phenotypes. Significant elevations in TIA incidence were observed in Graves’ disease for both females (3.7% vs. 2.4%, p = 0.006) and males (3.2% vs. 1.6%, p = 0.021), as well as male rheumatoid arthritis (2.9% vs. 1.6%, p = 0.035) and male myositis (5.7% vs. 3.5%, p < 0.001).

Ischemic stroke (I63) incidence was significantly higher in the cortisone-exposed groups for female rheumatoid arthritis (4.4% vs. 3.2%, p = 0.010), female myositis (5.8% vs. 4.8%, p = 0.008), and female Crohn’s disease (4.0% vs. 2.4%, p = 0.036). In contrast, high-prevalence conditions such as multiple sclerosis, autoimmune thyroiditis, and Type 1 Diabetes (with the exception of female TIA: 4.7% vs. 3.5%, p = 0.024) were essentially null across most vascular endpoints (all p > 0.05).

Sex modification of cortisone effects was limited for most conditions, with median F/M ratios remaining near 1.0. However, striking sex-specific differences emerged where significant signals were confined to one sex—such as the protective AD effect in CIDP and Crohn’s being exclusive to males.

Overall, Experiment 3 indicates that generic cortisone exposure is unlikely to be the primary driver of the broad autoimmune–neurovascular signal. The associations are highly disease-specific and directionally inconsistent, suggesting that cortisone acts more as a marker for disease-specific inflammatory severity or treatment escalation rather than a universal mediator of risk.

### Experiment 4: Targeted Treatments

**Fig. 4:**
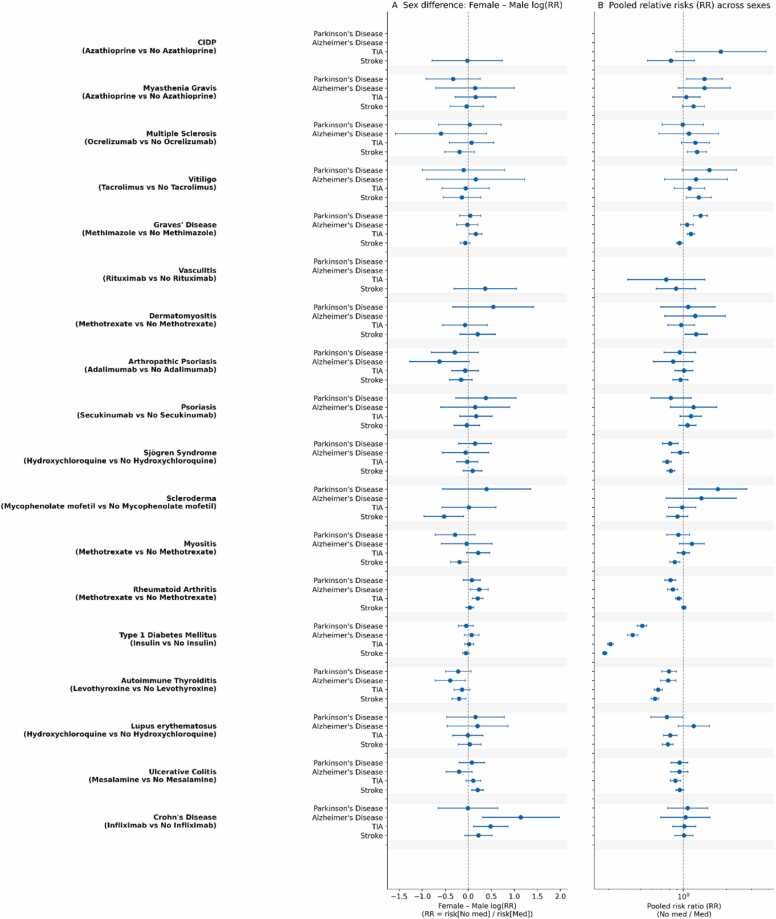
**| Heterogeneous Association Between Standard-of-Care Therapies and Neurological Risk.** Across disease–medication comparisons, pooled fixed-effect RRs (No-med/Med; **Panel B**) varied by indication, with an overall median RR near unity for neurodegenerative outcomes but significant deviations in high-severity cohorts. The strongest deviation was in type 1 diabetes mellitus, where insulin users showed substantially higher risks across all outcomes (all RR < 0.55), likely reflecting confounding by disease severity. In contrast, several therapies showed RRs > 1 consistent with a lower risk in the medicated cohort (e.g., methimazole in Graves’ disease for Parkinson’s disease RR ≈ 1.29; mycophenolate mofetil in scleroderma for Parkinson’s disease RR ≈ 1.40), while sex differences were modest and inconsistent (median female:male RR ratio ≈ 1.02; **Panel A**).

Across drug–disease pairs, most PD/AD associations were small or null, whereas TIA/ischemic stroke more often differed between treated vs untreated strata, with marked heterogeneity by indication and sex (Fig. 4**, detailed results in Appendix - EXPERIMENT 4**). Several outputs were incomplete as outcome numbers in the TriNetX database were too low in numbers, so interpretation is limited to reported endpoints.

Dominant “severity-signature” pattern (strongest across all endpoints).

• Type 1 diabetes (insulin vs no insulin): in both sexes, insulin-treated cohorts had substantially higher incidence of PD, AD, TIA, and ischemic stroke (all p < 0.001), with especially large absolute gaps for vascular outcomes (TIA/ischemic stroke rising from ∼1–2% to ∼3–8%). This is the single most coherent signal in Experiment 4 and most plausibly reflects disease severity/clinical trajectory rather than a medication-specific neurotoxicity claim.

Consistent vascular hot spots (TIA/ischemic stroke separation is robust).

• Autoimmune thyroiditis (levothyroxine): TIA and ischemic stroke were higher in treated cohorts in both sexes (all p < 0.001). Female PD and AD were also higher in treated cohorts (both p < 0.001), while male PD/AD were ∼null.
• Sjögren syndrome (hydroxychloroquine): treated cohorts showed higher PD (males p = 0.043; females p = 0.005) and higher TIA/ischemic stroke in both sexes (males p = 0.028/0.004; females both p < 0.001), with AD ∼null.
• Lupus (hydroxychloroquine): ischemic stroke was higher in treated cohorts in both sexes (males p = 0.017; females p < 0.001) and female TIA was higher (p < 0.001); PD/AD were largely null.

More selective / diagnosis-specific signals.

• Rheumatoid arthritis (methotrexate): treated cohorts showed higher PD and AD in both sexes (PD p = 0.001/0.002; AD p < 0.001/0.015) and higher male TIA (p < 0.001), while ischemic stroke was ∼null.
• Ulcerative colitis (mesalamine): male TIA and ischemic stroke were higher in treated cohorts (p = 0.002; p < 0.001); females were ∼null.
• Crohn’s disease (infliximab): male AD was higher in treated cohorts (p = 0.037); PD/TIA/ischemic stroke were ∼null; females showed a non-significant trend in the opposite direction.
• Myopathy phenotypes with methotrexate diverged: myositis females had higher ischemic stroke in treated cohorts (p < 0.001), whereas dermatomyositis females had lower ischemic stroke in treated cohorts (p = 0.013), emphasizing diagnosis-level heterogeneity.

Signals in the opposite direction (treated lower for some endpoints).

• Graves’ disease (methimazole): PD incidence was lower in treated cohorts in both sexes (males p = 0.005; females p < 0.001); female TIA was lower (p < 0.001) but female ischemic stroke was slightly higher (p = 0.004).
• Myasthenia gravis (azathioprine): male PD was lower in treated cohorts (p = 0.011); other endpoints were mostly null.
• Vitiligo (tacrolimus): male ischemic stroke was lower in treated cohorts (p = 0.040); other outcomes were null.

Compared with cortisone (Experiment 3), medication-specific strata (Experiment 4) reveal stronger and more sex-dependent separations, especially for TIA/ischemic stroke, but with directions that vary by disease and drug –supporting the idea that many “treatment effects” in this framework likely reflect confounding by indication/severity rather than a single shared pharmacologic mechanism.

## Discussion

Our EHR evaluation shows that adults with one of 22 AIDs have elevated incident risk of neurodegenerative diagnoses (PD and AD) and cerebrovascular events (TIA and ischemic stroke) compared with matched controls. Across conditions, median pooled RRs were ∼1.8 for both PD and AD. The effect was even more pronounced for cerebrovascular outcomes, with median RRs of approximately 2.6 for TIA and 2.2 for ischemic stroke. The highest risks were observed in rheumatoid vasculitis for TIA (RR ≈ 4.1) and ischemic stroke (RR ≈ 3.7). Conditions with severe systemic inflammation and vascular involvement showed the highest risk ratios, particularly rheumatoid vasculitis, lupus, and type 1 diabetes. Taken together, these findings position systemic autoimmunity as a robust marker of late-life neurological risk, with the most consistent and strongest associations observed for cerebrovascular outcomes.

Importantly, the magnitude and uniformity of these associations depended on how tightly baseline vascular burden was controlled. When we additionally matched on diseases of the circulatory system (Experiment 1B), many associations – particularly for AD – attenuated substantially and several moved toward the null, whereas residual cerebrovascular excess persisted in a narrower set of high-risk phenotypes. In contrast, additional matching on immune-suppressant exposure (Experiment 1 C) did not materially erase the pervasive TIA/ischemic stroke signal and left PD/AD broadly positive but more heterogeneous. This attenuation profile suggests that vascular comorbidity likely explains a meaningful share of the broad autoimmune–neurological signal (especially for AD), while cerebrovascular vulnerability remains comparatively robust across autoimmune phenotypes.

Our results extend and contextualize several earlier studies. The available meta-analyses indicate that patients with autoimmune diseases have a risk of Parkinson’s disease that is 1.5 times higher than the general population (M. Li et al., 2023). Our study provides more detailed information about how different diseases affect the risk of developing Parkinson's disease. The highest risk of developing Parkinson’s disease was found in patients with myasthenia gravis and chronic inflammatory demyelinating polyneuropathy, with pooled risk ratios of ∼2.4–2.8 (MG RR 2.76; CIDP RR 2.37). The PD risk associations for conditions like rheumatoid arthritis and ulcerative colitis were more modest, with risk ratios ranging from approximately 1.3–1.6. For AD, our findings align with recent cohort studies, showing a 40–70% increase in AD risk in autoimmune patients, particularly those with endocrine and gastrointestinal AIDs (Ramey et al., 2025). However, our vascular-burden robustness analysis indicates that a substantial component of the apparent AD excess across multiple AIDs may be mediated and/or confounded by baseline circulatory comorbidity, as many AD associations moved toward the null under circulatory-system matching (Experiment 1B). Additionally, genetic studies support these findings, showing that autoimmune disorders like multiple sclerosis are genetically linked to higher AD risk (Fominykh et al., 2023).

In terms of cerebrovascular risk, our results corroborate previous studies indicating elevated ischemic stroke and TIA risks in AIDs, especially vasculitic conditions and type 1 diabetes (Ahn et al., 2021, Bajko et al., 2021, Duarte et al., 2016, Geraldes et al., 2024, Janghorbani et al., 2007, Secrest et al., 2013, Tabakovic et al., 2023). Type 1 diabetes, which is known to be an autoimmune disease with microvascular aspects, turned out to be a strong risk factor for ischemic stroke according to our findings. Together, our analysis reveals the nuanced landscape of neurological burden across AIDs, highlighting conditions with disproportionately high cerebrovascular risks, such as vasculitides and type 1 diabetes, while others, like autoimmune thyroiditis, have a more modest impact. Notably, although effect sizes were attenuated under tighter vascular matching (Experiment 1B), a residual ischemic stroke/TIA excess persisted most consistently in a high-risk tier (e.g., T1DM, myositis, MS, RA, Addison’s disease). Across many phenotypes, this residual cerebrovascular excess remained larger in women than in men, consistent with the sex-stratified patterns observed in Experiment 1 A and 1B.

Systemic inflammation is a plausible contributor to autoimmune-associated neurological risk; however, our CRP-stratified analyses indicate that mild CRP elevation does not generate a uniform neurodegenerative gradient and instead highlights a disease-dependent ischemic vulnerability axis in selected inflammatory phenotypes. Scientific research indicates that immune-related genes such as HLA region and LRRK2 are linked to Parkinson’s disease pathogenesis (Herrick and Tansey, 2021, Witoelar et al., 2017). The penetration of immune factors through the blood-brain barrier in chronic inflammation activates glial cells, accelerating amyloid-β accumulation and tau pathology in AD (Chen et al., 2024, Kinney et al., 2018, Zenaro et al., 2017). Consistent with this disease-dependence, CRP stratification in our data was largely null for PD and AD in several high-prevalence AIDs, whereas ischemic stroke separated more reproducibly in inflammatory–vascular phenotypes such as psoriasis, myositis, Sjögren syndrome, vasculitis, and systemic sclerosis. Some observational studies suggest that anti-inflammatory treatments may be associated with lower dementia risk, for example methotrexate in rheumatoid arthritis (Newby et al., 2020). However, our medication-stratified analyses (Experiment 4) show heterogeneous—and in some settings higher—event incidence in treated cohorts, consistent with confounding by indication and severity rather than uniform neuroprotection.

Autoimmune inflammation drives atherosclerosis while simultaneously creating endothelial dysfunction and increasing blood coagulation leading to a hypercoagulable state and ultimately an increase in ischemic stroke risk (Murdaca et al., 2012, Surma and Filipiak, 2022). These mechanistic overlaps highlight the brain’s sensitivity to immune dysregulation, suggesting that the immune system may be a common pathway linking autoimmune diseases to neurological damage.

Most neurodegenerative and cerebrovascular disorders are strongly shaped by age, and the present findings are consistent with the view that some AIDs may converge with biological pathways that increase aging-related neurological vulnerability. Recent work has highlighted the importance of nocturnal physiological dampening and resetting by pineal melatonin and cortisol, particularly during the second half of sleep, in preparing cells and organ systems for the coming day (Anderson, 2024). The approximately 10-fold decline in pineal melatonin between childhood and the ninth decade of life (Karasek, 2004) may be an important contributor to aging through less effective night-time resetting. Related work indicates that sleep is a period that prioritizes mitochondrial function, with melatonin promoting mitochondrial fusion in contrast to daytime processes that favor mitochondrial fission (Richardson & Mailloux, 2023). Reduced pineal melatonin has also been linked to glymphatic dysfunction and less efficient nocturnal debris clearance in the brain (Sun et al., 2025). In this context, many AIDs, including multiple sclerosis, are associated with decreased melatonin (Anderson et al., 2019) and may therefore be viewed, like T1DM and T2DM, as conditions that can amplify age-related vulnerability, partly through melatonin suppression and its downstream consequences for night-time mitochondrial resetting and glymphatic function.

We observed notable sex-specific patterns in neurological risk. While the relative risk increase for neurodegenerative diseases was modest and varied, it was pronounced for cerebrovascular events. Our findings indicate that women with an autoimmune disease experience a systematically larger relative increase in TIA and ischemic stroke risk compared to men with the same condition, a pattern observed across most of the diseases studied. Regarding AD, women with AIDs did not show a greater relative risk increase than men; in fact, the risk increase was modestly smaller in women for several conditions. This is notable given that the absolute incidence of AD remained higher in women, consistent with the female predominance in the general population (Liesinger et al., 2018). These complex patterns likely reflect interacting differences in immune biology, vascular risk trajectories, and neuroinflammatory responses between women and men, including women’s generally stronger immune responses and hormonally modulated inflammatory signaling. This robust immune response of women might lead to higher rates of autoimmunity and thus more severe inflammation-related neurodegenerative conditions (Angum et al., 2020, Garmendia et al., 2024, Klein and Flanagan, 2016, Roved et al., 2017). The influence of estrogen on microglial activation through hormonal mechanisms might also contribute to women's increased risk of developing Alzheimer's disease (Bruce-Keller et al., 2000, Villa et al., 2016).

In Experiment 3, we investigated whether cortisone therapy modified neurological risk, hypothesizing a protective effect due to its anti-inflammatory properties. However, our findings did not support this hypothesis. For neurodegenerative outcomes, cortisone exposure was not materially associated with a change in risk for either Parkinson's or Alzheimer's disease, with risk ratios hovering near 1.00 across most conditions. In contrast, for cerebrovascular disease, we observed a modest but consistent pattern of increased risk. The median RR for TIA was approximately 1.20 and for ischemic stroke 1.29, indicating that patients on cortisone had a 20–30% higher risk of these events compared to patients with the same autoimmune disease who did not receive systemic corticosteroids. This treatment-associated risk was not uniform, being most pronounced and precise in patients with myositis, and rheumatoid arthritis. This association may be influenced by confounding by indication, where cortisone is prescribed for more severe disease activity, which itself carries a higher baseline vascular risk. Beyond confounding by indication, systemic corticosteroids may worsen intermediate vascular risk factors (e.g., hyperglycemia, hypertension, dyslipidemia), offering a plausible pathway for modest vascular risk elevation in susceptible phenotypes. Furthermore, sex-specific differences in corticosteroid pharmacokinetics and pharmacodynamics – such as faster methylprednisolone elimination in women and oestradiol-dependent increases in corticosteroid sensitivity – may contribute to heterogeneous risk patterns between sexes (Farkouh et al., 2021).

Experiment 4 reinforced the interpretation that medication exposure in EHR data frequently indexes disease trajectory and severity rather than isolating pharmacologic effects. Medication-specific strata produced stronger separations than cortisone in several settings, particularly for TIA/ischemic stroke, but directions varied by disease and drug. The most coherent pattern (insulin exposure in T1DM) is most plausibly a ‘severity signature’; similarly heterogeneous patterns across methotrexate, hydroxychloroquine, levothyroxine, biologics, and antithyroid therapy are compatible with confounding by indication and treatment escalation rather than a single shared medication mechanism.

Overall, our findings have immediate clinical implications centered on vascular prevention. Given the robustness and magnitude of the cerebrovascular signal across AIDs—especially in the high-risk tier highlighted in Experiments 1B/1 C—systematic vascular risk assessment and aggressive management of modifiable factors (blood pressure, glycemic control, lipids, smoking) should be prioritized as a core element of care. In parallel, heightened vigilance for parkinsonism and periodic cognitive assessment may help identify susceptible patients earlier. Multidisciplinary care integrating rheumatology/endocrinology and neurology can support coordinated control of systemic disease activity and vascular comorbidity.

The study supports the potential for neurological risk stratification in autoimmune patients, but it also underscores the limits of causal inference from treatment strata in observational EHR data. Rather than interpreting medication associations as neuroprotective or neurotoxic effects, our results suggest that risk stratification should primarily integrate autoimmune phenotype, vascular burden, and (where informative) inflammatory activity patterns. In clinical practice, steroid-sparing strategies may be preferable when clinically appropriate – particularly in patients with high baseline vascular risk – given the modest cerebrovascular risk elevations observed in cortisone-exposed strata. Future prospective studies and target-trial emulations are needed to test whether specific immunomodulatory strategies (e.g., IL-6 inhibitors, TNFα blockade, antimetabolites) modify cerebrovascular events or cognitive outcomes after careful control of disease activity, dose, duration, and indication.

This study benefited from two main strengths: its extensive size along with its robust federated EHR dataset which enabled us to detect small risk differences and perform detailed disease-specific investigations. The implementation of propensity score matching allowed for reduction of bias while the sex-stratified design revealed important findings about sex-specific effects. However, the observational design of this study allows for the possibility of misclassification errors and unknown confounding variables. Several observed associations – particularly in treatment-stratified analyses – may reflect confounding by indication, where medication exposure indexes more severe disease activity. The analysis contains age and sex as covariates, but potential residual confounders could impact the study results. Survivorship bias remains a limitation since patients with aggressive AIDs who develop neurodegenerative diseases might have passed away beforehand. These weaknesses deserve to be addressed in future research. Differential healthcare contact intensity may introduce surveillance bias, potentially inflating detection of PD/AD in autoimmune cohorts. Medication exposure is incompletely characterized with respect to dose, duration, adherence, and time-varying alignment to disease activity, increasing susceptibility to confounding by indication and time-related biases. Finally, diagnostic coding heterogeneity across sites and residual confounding by lifestyle and socioeconomic factors (e.g., smoking, BMI) may persist despite matching. While our analysis accounts for broad treatment categories and systemic inflammatory markers (CRP), EHR data lacks granular detail on specific autoantibody titers (e.g., antiphospholipid antibodies) and individual genetic susceptibility loci, which may further modulate cerebrovascular risk in specific systemic phenotypes.

In conclusion, this research demonstrates that autoimmune diseases produce major neurological threats while demonstrating the requirement for increased monitoring of these patients. Our robustness analyses suggest that vascular comorbidity accounts for a meaningful share of observed associations – particularly for AD – yet a clinically relevant cerebrovascular excess persists across autoimmune phenotypes, supporting vascular risk prevention as the most immediately actionable target while mechanistic work clarifies the roles of inflammation and treatment. Future research should investigate the fundamental mechanisms which connect autoimmune conditions with neurological disease and assess how immunomodulatory treatments affect extended neurological outcomes. Autoimmune population studies on neuroprotective interventions hold promise for developing novel therapeutic strategies to improve patient outcomes. The delivery of optimal autoimmune disease care benefits from close collaboration between neurology and immunology to develop integrated care pathways for patients at elevated neurological risk.

## CRediT authorship contribution statement

**Younes Adam Tabi:** Writing – original draft, Visualization, Validation, Project administration, Methodology, Investigation, Formal analysis, Data curation, Conceptualization. **Eva Christina Meyer:** Writing – review & editing, Visualization, Validation, Project administration, Methodology, Investigation, Formal analysis, Data curation, Conceptualization.

## Declaration of Competing Interest

The authors declare that they have no known competing financial interests or personal relationships that could have appeared to influence the work reported in this paper.
