# Supplementary material for: Autoimmune diseases are associated with increased neurodegenerative and cerebrovascular risk, while systemic corticosteroid exposure shows limited neurodegenerative and modest vascular associations
Source: IBRO Neurosci Rep. 2026 Mar 28;20:596–608. doi: 10.1016/j.ibneur.2026.03.010 (PMC13096927; doi:10.1016/j.ibneur.2026.03.010)
Supplement: Supplementary file 1 — Supplementary material [file mmc1.docx]

**APPENDIX**

**EXPERIMENT 1A**

**Chronic Inflammatory Demyelinating Polyneuropathy (CIDP)**

After 1:1 propensity score matching, the male cohorts consisted of 14 374 patients with CIDP and 14 374 matched controls; the female cohorts comprised 9 786 patients with CIDP and 9 786 matched controls. The characteristics of both cohorts were well balanced on age and sex after matching (standardized differences < 0.01). After matching, the mean current age of male CIDP patients and matched controls was 70.0 years (SD 9.0) in both groups; for females, the mean current age was 67.9 years (SD 9.5) in both CIDP and control cohorts.

*Parkinson’s Disease*
In the male cohort, 228 of 14 070 CIDP patients (1.6 %) and 101 of 14 323 controls (0.7 %) developed Parkinson’s disease, yielding a risk difference of 0.9 % (95 % CI 0.7–1.2), a risk ratio of 2.30 (95 % CI 1.82–2.90), and an odds ratio of 2.32 (95 % CI 1.83–2.94); p < 0.001. In the female cohort, 91 of 9 653 CIDP patients (0.9 %) versus 36 of 9 768 controls (0.4 %) were diagnosed with Parkinson’s disease (RD 0.6 %, 95 % CI 0.3–0.8; RR 2.56, 95 % CI 1.74–3.76; OR 2.57, 95 % CI 1.75–3.79; p < 0.001).

*Alzheimer’s Disease*
Among males, Alzheimer’s disease occurred in 113 of 14 311 CIDP patients (0.8 %) compared with 63 of 14 364 controls (0.4 %) (RD 0.4 %, 95 % CI 0.2–0.5; RR 1.80, 95 % CI 1.32–2.45; OR 1.81, 95 % CI 1.33–2.46; p < 0.001). In females, 59 of 9 749 CIDP patients (0.6 %) versus 31 of 9 774 controls (0.3 %) developed Alzheimer’s disease (RD 0.3 %, 95 % CI 0.1–0.5; RR 1.91, 95 % CI 1.24–2.95; OR 1.91, 95 % CI 1.24–2.96; p = 0.003).

*Transient Ischemic Attack (TIA)*TIA was observed in 325 of 13 993 CIDP males (2.3 %) compared with 124 of 14 340 controls (0.9 %) (RD 1.5 %, 95 % CI 1.2–1.7; RR 2.69, 95 % CI 2.19–3.30; OR 2.73, 95 % CI 2.21–3.36; p < 0.001). In the female cohort, 222 of 9 508 CIDP patients (2.3 %) versus 76 of 9 766 controls (0.8 %) experienced TIA (RD 1.6 %, 95 % CI 1.2–1.9; RR 3.00, 95 % CI 2.32–3.89; OR 3.05, 95 % CI 2.35–3.96; p < 0.001).

*Stroke*
Stroke incidence was 572 of 13 658 CIDP males (4.2 %) versus 296 of 14 223 controls (2.1 %) (RD 2.1 %, 95 % CI 1.7–2.5; RR 2.01, 95 % CI 1.75–2.31; OR 2.06, 95 % CI 1.78–2.37; p < 0.001). Among females, 375 of 9 332 CIDP patients (4.0 %) suffered stroke compared with 134 of 9 726 controls (1.4 %) (RD 2.6 %, 95 % CI 2.2–3.1; RR 2.92, 95 % CI 2.40–3.55; OR 3.00, 95 % CI 2.46–3.66; p < 0.001).

**Guillain–Barré Syndrome (GBS)**

After 1:1 propensity score matching, the male cohorts consisted of 14 610 patients with GBS and 14 610 matched controls; the female cohorts comprised 12 049 patients with GBS and 12 049 matched controls. The characteristics of both cohorts were well balanced on age and sex after matching (standardized differences < 0.01). After matching, the mean current age of male GBS patients and matched controls was 68.3 years (SD 9.4) in both groups; for females, the mean current age was 67.2 years (SD 9.6) in both GBS and control cohorts.

*Parkinson’s Disease*
In the male cohort, 137 of 14 426 GBS patients (0.9 %) and 76 of 14 576 controls (0.5 %) developed Parkinson’s disease, yielding a risk difference of 0.4 % (95 % CI 0.2–0.6), a risk ratio of 1.82 (95 % CI 1.38–2.41), and an odds ratio of 1.83 (95 % CI 1.38–2.42); p < 0.001. In the female cohort, 83 of 11 945 GBS patients (0.7 %) versus 46 of 12 034 controls (0.4 %) were diagnosed with Parkinson’s disease (RD 0.3 %, 95 % CI 0.1–0.5; RR 1.82, 95 % CI 1.27–2.60; OR 1.82, 95 % CI 1.27–2.62; p = 0.001).

*Alzheimer’s Disease*
Among males, Alzheimer’s disease occurred in 70 of 14 574 GBS patients (0.5 %) compared with 58 of 14 599 controls (0.4 %) (RD 0.1 %, 95 % CI –0.1–0.2; RR 1.21, 95 % CI 0.85–1.71; OR 1.21, 95 % CI 0.85–1.72; p = 0.283). In females, 66 of 12 012 GBS patients (0.5 %) versus 56 of 12 035 controls (0.5 %) developed Alzheimer’s disease (RD 0.0 %, 95 % CI –0.1–0.1; RR 1.18, 95 % CI 0.83–1.68; OR 1.18, 95 % CI 0.83–1.69; p = 0.358).

*Transient Ischemic Attack (TIA)*
TIA was observed in 274 of 14 255 GBS males (1.9 %) compared with 114 of 14 585 controls (0.8 %) (RD 1.1 %, 95 % CI 0.9–1.4; RR 2.46, 95 % CI 1.98–3.06; OR 2.49, 95 % CI 2.00–3.10; p < 0.001). In the female cohort, 255 of 11 762 GBS patients (2.2 %) versus 96 of 12 024 controls (0.8 %) experienced TIA (RD 1.4 %, 95 % CI 1.1–1.7; RR 2.72, 95 % CI 2.15–3.43; OR 2.75, 95 % CI 2.17–3.49; p < 0.001).

*Stroke*
Stroke incidence was 552 of 13 759 GBS males (4.0 %) versus 250 of 14 488 controls (1.7 %) (RD 2.3 %, 95 % CI 1.9–2.7; RR 2.33, 95 % CI 2.01–2.70; OR 2.38, 95 % CI 2.05–2.77; p < 0.001). Among females, 457 of 11 435 GBS patients (4.0 %) suffered stroke compared with 176 of 11 984 controls (1.5 %) (RD 2.5 %, 95 % CI 2.1–2.9; RR 2.79, 95 % CI 2.34–3.33; OR 2.79, 95 % CI 2.34–3.33; p < 0.001).

**Myasthenia Gravis (MG)**

After 1:1 propensity score matching, the male cohorts consisted of 27 932 patients with MG and 27 932 matched controls; the female cohorts comprised 28 265 patients with MG and 28 265 matched controls. The characteristics of both cohorts were well balanced on age and sex after matching (standardized differences < 0.01). After matching, the mean current age of male MG patients and matched controls was 71.3 years (SD 9.0) in both groups; for females, the mean current age was 68.6 years (SD 9.8) in both MG and control cohorts.

*Parkinson’s Disease*
In the male cohort, 459 of 27 340 MG patients (1.7 %) and 177 of 27 826 controls (0.6 %) developed Parkinson’s disease, yielding a risk difference of 1.0 % (95 % CI 0.9–1.2), a risk ratio of 2.64 (95 % CI 2.22–3.14), and an odds ratio of 2.67 (95 % CI 2.24–3.18); p < 0.001. In the female cohort, 306 of 27 941 MG patients (1.1 %) versus 104 of 28 225 controls (0.4 %) were diagnosed with Parkinson’s disease (RD 0.7 %, 95 % CI 0.6–0.9; RR 2.97, 95 % CI 2.38–3.71; OR 2.99, 95 % CI 2.40–3.74; p < 0.001).

*Alzheimer’s Disease*
Among males, Alzheimer’s disease occurred in 189 of 27 822 MG patients (0.7 %) compared with 140 of 27 897 controls (0.5 %) (RD 0.2 %, 95 % CI 0.1–0.3; RR 1.35, 95 % CI 1.09–1.68; OR 1.36, 95 % CI 1.09–1.69; p = 0.006). In females, 203 of 28 165 MG patients (0.7 %) versus 113 of 28 231 controls (0.4 %) developed Alzheimer’s disease (RD 0.3 %, 95 % CI 0.2–0.4; RR 1.80, 95 % CI 1.43–2.27; OR 1.81, 95 % CI 1.43–2.28; p < 0.001).

*Transient Ischemic Attack (TIA)*TIA was observed in 592 of 27 055 MG males (2.2 %) compared with 260 of 27 860 controls (0.9 %) (RD 1.3 %, 95 % CI 1.0–1.5; RR 2.35, 95 % CI 2.03–2.71; OR 2.38, 95 % CI 2.05–2.75; p < 0.001). In the female cohort, 638 of 27 346 MG patients (2.3 %) versus 212 of 28 202 controls (0.8 %) experienced TIA (RD 1.6 %, 95 % CI 1.4–1.8; RR 3.10, 95 % CI 2.66–3.62; OR 3.15, 95 % CI 2.70–3.69; p < 0.001).

*Stroke*
Stroke incidence was 1 008 of 26 511 MG males (3.8 %) versus 493 of 27 639 controls (1.8 %) (RD 2.0 %, 95 % CI 1.7–2.3; RR 2.13, 95 % CI 1.92–2.37; OR 2.18, 95 % CI 1.95–2.43; p < 0.001). Among females, 915 of 27 078 MG patients (3.4 %) suffered stroke compared with 402 of 28 099 controls (1.4 %) (RD 2.0 %, 95 % CI 1.7–2.2; RR 2.36, 95 % CI 2.10–2.65; OR 2.41, 95 % CI 2.14–2.71; p < 0.001).

**Multiple Sclerosis (MS)**

After 1:1 propensity score matching, the male cohorts consisted of 55 939 patients with MS and 55 939 matched controls; the female cohorts comprised 154 944 patients with MS and 154 944 matched controls. The characteristics of both cohorts were well balanced on age and sex after matching (standardized differences < 0.01). After matching, the mean current age of male MS patients and matched controls was 64.5 years (SD 9.1) in both groups; for females, the mean current age was 64.2 years (SD 9.0) in both MS and control cohorts.

*Parkinson’s Disease*
In the male cohort, 581 of 55 207 MS patients (1.1 %) and 265 of 55 853 controls (0.5 %) developed Parkinson’s disease, yielding a risk difference of 0.6 % (95 % CI 0.5–0.7), a risk ratio of 2.22 (95 % CI 1.92–2.56), and an odds ratio of 2.23 (95 % CI 1.93–2.58); p < 0.001. In the female cohort, 1 006 of 154 126 MS patients (0.7 %) versus 453 of 154 814 controls (0.3 %) were diagnosed with Parkinson’s disease (RD 0.4 %, 95 % CI 0.3–0.4; RR 2.23, 95 % CI 1.99–2.50; OR 2.24, 95 % CI 2.00–2.50; p < 0.001).

*Alzheimer’s Disease*
Among males, Alzheimer’s disease occurred in 269 of 55 777 MS patients (0.5 %) compared with 143 of 55 914 controls (0.3 %) (RD 0.2 %, 95 % CI 0.2–0.3; RR 1.89, 95 % CI 1.54–2.31; OR 1.89, 95 % CI 1.54–2.32; p < 0.001). In females, 700 of 154 616 MS patients (0.5 %) versus 393 of 154 871 controls (0.3 %) developed Alzheimer’s disease (RD 0.2 %, 95 % CI 0.2–0.2; RR 1.78, 95 % CI 1.58–2.02; OR 1.79, 95 % CI 1.58–2.02; p < 0.001).

*Transient Ischemic Attack (TIA)*TIA was observed in 868 of 55 071 MS males (1.6 %) compared with 466 of 55 839 controls (0.8 %) (RD 0.7 %, 95 % CI 0.6–0.9; RR 1.89, 95 % CI 1.69–2.11; OR 1.90, 95 % CI 1.70–2.13; p < 0.001). In the female cohort, 2 730 of 152 839 MS patients (1.8 %) versus 1 207 of 154 706 controls (0.8 %) experienced TIA (RD 1.0 %, 95 % CI 0.9–1.1; RR 2.29, 95 % CI 2.14–2.45; OR 2.31, 95 % CI 2.16–2.48; p < 0.001).

*Stroke*
Stroke incidence was 1 886 of 53 801 MS males (3.5 %) versus 1 006 of 55 582 controls (1.8 %) (RD 1.7 %, 95 % CI 1.5–1.9; RR 1.94, 95 % CI 1.80–2.09; OR 1.97, 95 % CI 1.82–2.13; p < 0.001). Among females, 4 888 of 150 720 MS patients (3.2 %) suffered stroke compared with 2 022 of 154 317 controls (1.3 %) (RD 1.9 %, 95 % CI 1.8–2.0; RR 2.48, 95 % CI 2.35–2.60; OR 2.53, 95 % CI 2.40–2.66; p < 0.001).

**Vitiligo**

After 1:1 propensity score matching, the male cohorts consisted of 23 558 patients with Vitiligo and 23 558 matched controls; the female cohorts comprised 32 109 patients with Vitiligo and 32 109 matched controls. The characteristics of both cohorts were well balanced on age and sex after matching (standardized differences < 0.01). After matching, the mean current age of male Vitiligo patients and matched controls was 66.0 years (SD 9.5) in both groups; for females, the mean current age was 65.9 years (SD 9.4) in both Vitiligo and control cohorts.

*Parkinson’s Disease*In the male cohort, 155 of 23 417 Vitiligo patients (0.7 %) and 118 of 23 507 controls (0.5 %) developed Parkinson’s disease, yielding a risk difference of 0.2 % (95 % CI 0.0–0.3), a risk ratio of 1.32 (95 % CI 1.04–1.67), and an odds ratio of 1.32 (95 % CI 1.04–1.68); p = 0.023. In the female cohort, 142 of 32 024 Vitiligo patients (0.4 %) versus 98 of 32 082 controls (0.3 %) were diagnosed with Parkinson’s disease (RD 0.1 %, 95 % CI 0.0–0.2; RR 1.45, 95 % CI 1.12–1.88; OR 1.45, 95 % CI 1.12–1.88; p = 0.004).

*Alzheimer’s Disease*
Among males, Alzheimer’s disease occurred in 99 of 23 504 Vitiligo patients (0.4 %) compared with 70 of 23 541 controls (0.3 %) (RD 0.1 %, 95 % CI 0.0–0.2; RR 1.42, 95 % CI 1.04–1.92; OR 1.42, 95 % CI 1.04–1.93; p = 0.025). In females, 176 of 32 044 Vitiligo patients (0.5 %) versus 95 of 32 090 controls (0.3 %) developed Alzheimer’s disease (RD 0.2 %, 95 % CI 0.2–0.4; RR 1.86, 95 % CI 1.45–2.38; OR 1.86, 95 % CI 1.45–2.39; p < 0.001).

*Transient Ischemic Attack (TIA)*
TIA was observed in 371 of 23 203 Vitiligo males (1.6 %) compared with 201 of 23 505 controls (0.9 %) (RD 0.7 %, 95 % CI 0.5–0.9; RR 1.87, 95 % CI 1.58–2.22; OR 1.88, 95 % CI 1.58–2.24; p < 0.001). In the female cohort, 612 of 31 577 Vitiligo patients (1.9 %) versus 226 of 32 051 controls (0.7 %) experienced TIA (RD 1.2 %, 95 % CI 1.1–1.4; RR 2.75, 95 % CI 2.36–3.20; OR 2.78, 95 % CI 2.39–3.24; p < 0.001).

*Stroke*
Stroke incidence was 603 of 22 989 Vitiligo males (2.6 %) versus 424 of 23 378 controls (1.8 %) (RD 0.8 %, 95 % CI 0.5–1.1; RR 1.45, 95 % CI 1.28–1.64; OR 1.46, 95 % CI 1.29–1.65; p < 0.001). Among females, 741 of 31 474 Vitiligo patients (2.4 %) suffered stroke compared with 461 of 31 943 controls (1.4 %) (RD 0.9 %, 95 % CI 0.7–1.1; RR 1.63, 95 % CI 1.46–1.83; OR 1.65, 95 % CI 1.46–1.85; p < 0.001).

**Rheumatoid Vasculitis (RV)**

After 1:1 propensity score matching, the male cohorts consisted of 1 906 patients with RV and 1 906 matched controls; the female cohorts comprised 6 426 patients with RV and 6 426 matched controls. The characteristics of both cohorts were well balanced on age and sex after matching (standardized differences < 0.01). After matching, the mean current age of male RV patients and matched controls was 69.0 years (SD 9.1) in both groups; for females, the mean current age was 68.1 years (SD 9.4) in both RV and control cohorts.

*Parkinson’s Disease*In the male cohort, 14 of 1 881 RV patients (0.7 %) and 10 of 1 901 controls (0.5 %) developed Parkinson’s disease, yielding a risk difference of 0.2 % (95 % CI –0.3–0.7), a risk ratio of 1.42 (95 % CI 0.63–3.18), and an odds ratio of 1.42 (95 % CI 0.63–3.20); p = 0.398. In the female cohort, 35 of 6 381 RV patients (0.5 %) versus 30 of 6 415 controls (0.5 %) were diagnosed with Parkinson’s disease (RD 0.1 %, 95 % CI –0.2–0.3; RR 1.17, 95 % CI 0.72–1.91; OR 1.17, 95 % CI 0.72–1.91; p = 0.520).

*Alzheimer’s Disease*Among males, Alzheimer’s disease occurred in 16 of 1 901 RV patients (0.8 %) compared with 10 of 1 903 controls (0.5 %) (RD 0.3 %, 95 % CI –0.2–0.8; RR 1.60, 95 % CI 0.73–3.52; OR 1.61, 95 % CI 0.73–3.55; p = 0.237). In females, 63 of 6 397 RV patients (1.0 %) versus 27 of 6 419 controls (0.4 %) developed Alzheimer’s disease (RD 0.6 %, 95 % CI 0.3–0.9; RR 2.34, 95 % CI 1.49–3.67; OR 2.36, 95 % CI 1.50–3.70; p < 0.001).

*Transient Ischemic Attack (TIA)*
TIA was observed in 40 of 1 847 RV males (2.2 %) compared with 10 of 1 901 controls (0.5 %) (RD 1.6 %, 95 % CI 0.9–2.4; RR 4.12, 95 % CI 2.07–8.21; OR 4.19, 95 % CI 2.09–8.40; p < 0.001). In the female cohort, 174 of 6 210 RV patients (2.8 %) versus 44 of 6 408 controls (0.7 %) experienced TIA (RD 2.1 %, 95 % CI 1.7–2.6; RR 4.08, 95 % CI 2.94–5.67; OR 4.17, 95 % CI 2.99–5.82; p < 0.001).

*Stroke*
Stroke incidence was 80 of 1 794 RV males (4.5 %) versus 24 of 1 894 controls (1.3 %) (RD 3.2 %, 95 % CI 2.1–4.3; RR 3.52, 95 % CI 2.24–5.53; OR 3.64, 95 % CI 2.29–5.77; p < 0.001). Among females, 244 of 6 124 RV patients (4.0 %) suffered stroke compared with 67 of 6 393 controls (1.0 %) (RD 2.9 %, 95 % CI 2.4–3.5; RR 3.80, 95 % CI 2.91–4.97; OR 3.92, 95 % CI 2.98–5.15; p < 0.001).

**Graves’ Disease**

After 1:1 propensity score matching, the male cohorts consisted of 131 104 patients with Graves’ Disease and 131 104 matched controls; the female cohorts comprised 375 670 patients with Graves’ Disease and 375 670 matched controls. The characteristics of both cohorts were well balanced on age and sex after matching (standardized differences < 0.01). After matching, the mean current age of male Graves’ Disease patients and matched controls was 67.8 years (SD 9.5) in both groups; for females, the mean current age was 66.7 years (SD 9.7) in both Graves’ Disease and control cohorts.

*Parkinson’s Disease*In the male cohort, 1 222 of 129 652 Graves’ Disease patients (0.9 %) and 749 of 130 750 controls (0.6 %) developed Parkinson’s disease, yielding a risk difference of 0.4 % (95 % CI 0.3–0.4), a risk ratio of 1.65 (95 % CI 1.50–1.80), and an odds ratio of 1.65 (95 % CI 1.51–1.81); p < 0.001. In the female cohort, 2 075 of 373 710 Graves’ Disease patients (0.6 %) versus 1 217 of 375 126 controls (0.3 %) were diagnosed with Parkinson’s disease (RD 0.3 %, 95 % CI 0.2–0.3; RR 1.71, 95 % CI 1.60–1.84; OR 1.72, 95 % CI 1.60–1.84; p < 0.001).

*Alzheimer’s Disease*
Among males, Alzheimer’s disease occurred in 913 of 130 516 Graves’ Disease patients (0.7 %) compared with 426 of 131 002 controls (0.3 %) (RD 0.4 %, 95 % CI 0.3–0.4; RR 2.15, 95 % CI 1.92–2.41; OR 2.16, 95 % CI 1.92–2.42; p < 0.001). In females, 2 650 of 374 140 Graves’ Disease patients (0.7 %) versus 1 370 of 375 311 controls (0.4 %) developed Alzheimer’s disease (RD 0.3 %, 95 % CI 0.3–0.4; RR 1.94, 95 % CI 1.82–2.07; OR 1.95, 95 % CI 1.82–2.08; p < 0.001).

*Transient Ischemic Attack (TIA)*
TIA was observed in 2 761 of 127 991 Graves’ Disease males (2.2 %) compared with 1 060 of 130 814 controls (0.8 %) (RD 1.3 %, 95 % CI 1.3–1.4; RR 2.66, 95 % CI 2.48–2.86; OR 2.70, 95 % CI 2.51–2.90; p < 0.001). In the female cohort, 7 439 of 368 419 Graves’ Disease patients (2.0 %) versus 2 985 of 374 914 controls (0.8 %) experienced TIA (RD 1.2 %, 95 % CI 1.2–1.3; RR 2.54, 95 % CI 2.43–2.65; OR 2.57, 95 % CI 2.46–2.68; p < 0.001).

*Stroke*
Stroke incidence was 4 782 of 124 827 Graves’ Disease males (3.8 %) versus 2 323 of 130 076 controls (1.8 %) (RD 2.0 %, 95 % CI 1.9–2.2; RR 2.15, 95 % CI 2.04–2.25; OR 2.19, 95 % CI 2.08–2.30; p < 0.001). Among females, 10 799 of 364 219 Graves’ Disease patients (3.0 %) suffered stroke compared with 5 085 of 373 755 controls (1.4 %) (RD 1.6 %, 95 % CI 1.5–1.7; RR 2.18, 95 % CI 2.11–2.25; OR 2.22, 95 % CI 2.14–2.29; p < 0.001).

**Vasculitis**

After 1:1 propensity score matching, the male cohorts consisted of 25 868 patients with Vasculitis and 25 868 matched controls; the female cohorts comprised 54 658 patients with Vasculitis and 54 658 matched controls. The characteristics of both cohorts were well balanced on age and sex after matching (standardized differences < 0.01). After matching, the mean current age of male Vasculitis patients and matched controls was 68.7 years (SD 9.6) in both groups; for females, the mean current age was 68.1 years (SD 9.7) in both Vasculitis and control cohorts.

*Parkinson’s Disease*
In the male cohort, 317 of 25 610 Vasculitis patients (1.2 %) and 192 of 25 783 controls (0.7 %) developed Parkinson’s disease, yielding a risk difference of 0.5 % (95 % CI 0.3–0.7), a risk ratio of 1.66 (95 % CI 1.39–1.99), and an odds ratio of 1.67 (95 % CI 1.40–2.00); p < 0.001. In the female cohort, 414 of 54 373 Vasculitis patients (0.8 %) versus 231 of 54 575 controls (0.4 %) were diagnosed with Parkinson’s disease (RD 0.3 %, 95 % CI 0.2–0.4; RR 1.80, 95 % CI 1.53–2.11; OR 1.81, 95 % CI 1.54–2.12; p < 0.001).

*Alzheimer’s Disease*
Among males, Alzheimer’s disease occurred in 164 of 25 780 Vasculitis patients (0.6 %) compared with 104 of 25 846 controls (0.4 %) (RD 0.2 %, 95 % CI 0.1–0.4; RR 1.58, 95 % CI 1.24–2.02; OR 1.59, 95 % CI 1.24–2.03; p < 0.001). In females, 553 of 54 454 Vasculitis patients (1.0 %) versus 249 of 54 619 controls (0.5 %) developed Alzheimer’s disease (RD 0.6 %, 95 % CI 0.5–0.7; RR 2.23, 95 % CI 1.92–2.59; OR 2.24, 95 % CI 1.93–2.60; p < 0.001).

*Transient Ischemic Attack (TIA)*TIA was observed in 656 of 25 224 Vasculitis males (2.6 %) compared with 312 of 25 804 controls (1.2 %) (RD 1.4 %, 95 % CI 1.2–1.6; RR 2.15, 95 % CI 1.88–2.46; OR 2.18, 95 % CI 1.90–2.50; p < 0.001). In the female cohort, 1 547 of 53 160 Vasculitis patients (2.9 %) versus 570 of 54 578 controls (1.0 %) experienced TIA (RD 1.9 %, 95 % CI 1.7–2.0; RR 2.79, 95 % CI 2.53–3.07; OR 2.84, 95 % CI 2.58–3.13; p < 0.001).

*Stroke*
Stroke incidence was 1 085 of 24 796 Vasculitis males (4.4 %) versus 619 of 25 653 controls (2.4 %) (RD 2.0 %, 95 % CI 1.6–2.3; RR 1.81, 95 % CI 1.65–2.00; OR 1.85, 95 % CI 1.67–2.05; p < 0.001). Among females, 2 228 of 52 837 Vasculitis patients (4.2 %) suffered stroke compared with 890 of 54 408 controls (1.6 %) (RD 2.6 %, 95 % CI 2.4–2.8; RR 2.58, 95 % CI 2.39–2.78; OR 2.65, 95 % CI 2.45–2.86; p < 0.001).

**Dermatomyositis**

After 1:1 propensity score matching, the male cohorts consisted of 11 362 patients with Dermatomyositis and 11 362 matched controls; the female cohorts comprised 23 157 patients with Dermatomyositis and 23 157 matched controls. The characteristics of both cohorts were well balanced on age and sex after matching (standardized differences < 0.01). After matching, the mean current age of male Dermatomyositis patients and matched controls was 68.2 years (SD 9.4) in both groups; for females, the mean current age was 67.3 years (SD 9.4) in both Dermatomyositis and control cohorts.

*Parkinson’s Disease*In the male cohort, 118 of 11 288 Dermatomyositis patients (1.0 %) and 73 of 11 338 controls (0.6 %) developed Parkinson’s disease, yielding a risk difference of 0.4 % (95 % CI 0.2–0.6), a risk ratio of 1.62 (95 % CI 1.21–2.17), and an odds ratio of 1.63 (95 % CI 1.22–2.19); p = 0.001. In the female cohort, 126 of 23 037 Dermatomyositis patients (0.5 %) versus 94 of 23 121 controls (0.4 %) were diagnosed with Parkinson’s disease (RD 0.1 %, 95 % CI 0.0–0.3; RR 1.35, 95 % CI 1.03–1.76; OR 1.35, 95 % CI 1.03–1.76; p = 0.029).

*Alzheimer’s Disease*Among males, Alzheimer’s disease occurred in 52 of 11 341 Dermatomyositis patients (0.5 %) compared with 37 of 11 352 controls (0.3 %) (RD 0.2 %, 95 % CI –0.0–0.3; RR 1.41, 95 % CI 0.92–2.14; OR 1.41, 95 % CI 0.92–2.15; p = 0.110). In females, 132 of 23 118 Dermatomyositis patients (0.6 %) versus 85 of 23 138 controls (0.4 %) developed Alzheimer’s disease (RD 0.2 %, 95 % CI 0.1–0.3; RR 1.56, 95 % CI 1.19–2.04; OR 1.56, 95 % CI 1.19–2.05; p = 0.001).

*Transient Ischemic Attack (TIA)*TIA was observed in 269 of 11 152 Dermatomyositis males (2.4 %) compared with 116 of 11 335 controls (1.0 %) (RD 1.4 %, 95 % CI 1.0–1.7; RR 2.36, 95 % CI 1.90–2.93; OR 2.39, 95 % CI 1.92–2.98; p < 0.001). In the female cohort, 536 of 22 713 Dermatomyositis patients (2.4 %) versus 202 of 23 110 controls (0.9 %) experienced TIA (RD 1.5 %, 95 % CI 1.3–1.7; RR 2.70, 95 % CI 2.30–3.18; OR 2.74, 95 % CI 2.33–3.23; p < 0.001).

*Stroke*
Stroke incidence was 490 of 10 925 Dermatomyositis males (4.5 %) versus 226 of 11 268 controls (2.0 %) (RD 2.5 %, 95 % CI 2.0–2.9; RR 2.24, 95 % CI 1.91–2.61; OR 2.29, 95 % CI 1.96–2.69; p < 0.001). Among females, 865 of 22 565 Dermatomyositis patients (3.8 %) suffered stroke compared with 335 of 23 052 controls (1.5 %) (RD 2.3 %, 95 % CI 2.1–2.7; RR 2.64, 95 % CI 2.33–2.99; OR 2.70, 95 % CI 2.38–3.07; p < 0.001).

**Arthropathic Psoriasis**

After 1:1 propensity score matching, the male cohorts consisted of 52 848 patients with Arthropathic Psoriasis and 52 848 matched controls; the female cohorts comprised 75 297 patients with Arthropathic Psoriasis and 75 297 matched controls. The characteristics of both cohorts were well balanced on age and sex after matching (standardized differences < 0.01). After matching, the mean current age of male Arthropathic Psoriasis patients and matched controls was 65.8 years (SD 9.0) in both groups; for females, the mean current age was 65.2 years (SD 9.1) in both Arthropathic Psoriasis and control cohorts.

*Parkinson’s Disease*In the male cohort, 431 of 52 563 Arthropathic Psoriasis patients (0.8 %) and 272 of 52 712 controls (0.5 %) developed Parkinson’s disease, yielding a risk difference of 0.3 % (95 % CI 0.2–0.4), a risk ratio of 1.59 (95 % CI 1.37–1.85), and an odds ratio of 1.59 (95 % CI 1.37–1.86); p < 0.001. In the female cohort, 360 of 75 060 Arthropathic Psoriasis patients (0.5 %) versus 204 of 75 210 controls (0.3 %) were diagnosed with Parkinson’s disease (RD 0.2 %, 95 % CI 0.1–0.3; RR 1.77, 95 % CI 1.49–2.10; OR 1.77, 95 % CI 1.49–2.11; p < 0.001).

*Alzheimer’s Disease*
Among males, Alzheimer’s disease occurred in 225 of 52 769 Arthropathic Psoriasis patients (0.4 %) compared with 138 of 52 811 controls (0.3 %) (RD 0.2 %, 95 % CI 0.1–0.2; RR 1.63, 95 % CI 1.32–2.02; OR 1.63, 95 % CI 1.32–2.02; p < 0.001). In females, 333 of 75 180 Arthropathic Psoriasis patients (0.4 %) versus 224 of 75 252 controls (0.3 %) developed Alzheimer’s disease (RD 0.1 %, 95 % CI 0.1–0.2; RR 1.49, 95 % CI 1.26–1.76; OR 1.49, 95 % CI 1.26–1.77; p < 0.001).

*Transient Ischemic Attack (TIA)*
TIA was observed in 917 of 52 079 Arthropathic Psoriasis males (1.8 %) compared with 396 of 52 742 controls (0.8 %) (RD 1.0 %, 95 % CI 0.9–1.1; RR 2.35, 95 % CI 2.09–2.64; OR 2.37, 95 % CI 2.11–2.67; p < 0.001). In the female cohort, 1 366 of 74 082 Arthropathic Psoriasis patients (1.8 %) versus 492 of 75 162 controls (0.7 %) experienced TIA (RD 1.2 %, 95 % CI 1.1–1.3; RR 2.82, 95 % CI 2.54–3.12; OR 2.85, 95 % CI 2.57–3.16; p < 0.001).

*Stroke*
Stroke incidence was 1 379 of 51 660 Arthropathic Psoriasis males (2.7 %) versus 867 of 52 420 controls (1.7 %) (RD 1.0 %, 95 % CI 0.8–1.2; RR 1.61, 95 % CI 1.48–1.76; OR 1.63, 95 % CI 1.50–1.78; p < 0.001). Among females, 1 804 of 73 797 Arthropathic Psoriasis patients (2.4 %) suffered stroke compared with 865 of 74 904 controls (1.2 %) (RD 1.3 %, 95 % CI 1.2–1.4; RR 2.12, 95 % CI 1.95–2.29; OR 2.15, 95 % CI 1.98–2.33; p < 0.001).

**Addison’s Disease**

After 1:1 propensity score matching, the male cohorts consisted of 18 557 patients with Addison’s disease and 18 557 matched controls; the female cohorts comprised 27 298 patients with Addison’s disease and 27 298 matched controls. The characteristics of both cohorts were well balanced on age and sex after matching (standardized differences < 0.01). After matching, the mean current age of male Addison’s disease patients and matched controls was 68.7 years (SD 9.4) in both groups; for females, the mean current age was 66.9 years (SD 9.6) in both Addison’s disease and control cohorts.

*Parkinson’s Disease*
In the male cohort, 225 of 18 281 Addison’s disease patients (1.2 %) and 125 of 18 515 controls (0.7 %) developed Parkinson’s disease, yielding a risk difference of 0.6 % (95 % CI 0.4–0.8), a risk ratio of 1.82 (95 % CI 1.47–2.27), and an odds ratio of 1.83 (95 % CI 1.47–2.28); p < 0.001. In the female cohort, 226 of 27 079 Addison’s disease patients (0.8 %) versus 88 of 27 260 controls (0.3 %) were diagnosed with Parkinson’s disease (RD 0.5 %, 95 % CI 0.4–0.6; RR 2.58, 95 % CI 2.02–3.30; OR 2.60, 95 % CI 2.03–3.32; p < 0.001).

*Alzheimer’s Disease*
Among males, Alzheimer’s disease occurred in 103 of 18 469 Addison’s disease patients (0.6 %) compared with 69 of 18 550 controls (0.4 %) (RD 0.2 %, 95 % CI 0.0–0.3; RR 1.50, 95 % CI 1.11–2.03; OR 1.50, 95 % CI 1.11–2.04; p = 0.009). In females, 224 of 27 198 Addison’s disease patients (0.8 %) versus 110 of 27 282 controls (0.4 %) developed Alzheimer’s disease (RD 0.4 %, 95 % CI 0.3–0.6; RR 2.04, 95 % CI 1.63–2.56; OR 2.05, 95 % CI 1.63–2.58; p < 0.001).

*Transient Ischemic Attack (TIA)*
TIA was observed in 427 of 18 036 Addison’s disease males (2.4 %) compared with 185 of 18 524 controls (1.0 %) (RD 1.4 %, 95 % CI 1.1–1.6; RR 2.37, 95 % CI 2.00–2.81; OR 2.40, 95 % CI 2.02–2.86; p < 0.001). In the female cohort, 729 of 26 461 Addison’s disease patients (2.8 %) versus 253 of 27 247 controls (0.9 %) experienced TIA (RD 1.8 %, 95 % CI 1.6–2.1; RR 2.97, 95 % CI 2.58–3.42; OR 3.02, 95 % CI 2.62–3.50; p < 0.001).

*Stroke*
Stroke incidence was 886 of 17 366 Addison’s disease males (5.1 %) versus 345 of 18 425 controls (1.9 %) (RD 3.2 %, 95 % CI 2.8–3.6; RR 2.72, 95 % CI 2.41–3.08; OR 2.82, 95 % CI 2.48–3.20; p < 0.001). Among females, 1 287 of 25 932 Addison’s disease patients (5.0 %) suffered stroke compared with 442 of 27 160 controls (1.6 %) (RD 3.3 %, 95 % CI 3.0–3.6; RR 3.05, 95 % CI 2.74–3.39; OR 3.15, 95 % CI 2.83–3.52; p < 0.001).

**Psoriasis**

After 1:1 propensity score matching, the male cohorts consisted of 228 002 patients with Psoriasis and 228 002 matched controls; the female cohorts comprised 267 763 patients with Psoriasis and 267 763 matched controls. The characteristics of both cohorts were well balanced on age and sex after matching (standardized differences < 0.01). After matching, the mean current age of male Psoriasis patients and matched controls was 66.5 years (SD 9.4) in both groups; for females, the mean current age was 66.3 years (SD 9.4) in both Psoriasis and control cohorts.

*Parkinson’s Disease*
In the male cohort, 1 728 of 226 587 Psoriasis patients (0.8 %) and 1 171 of 227 451 controls (0.5 %) developed Parkinson’s disease, yielding a risk difference of 0.2 % (95 % CI 0.2–0.3), a risk ratio of 1.48 (95 % CI 1.38–1.60), and an odds ratio of 1.49 (95 % CI 1.38–1.60); p < 0.001. In the female cohort, 1 283 of 266 801 Psoriasis patients (0.5 %) versus 834 of 267 372 controls (0.3 %) were diagnosed with Parkinson’s disease (RD 0.2 %, 95 % CI 0.1–0.2; RR 1.54, 95 % CI 1.41–1.68; OR 1.54, 95 % CI 1.42–1.68; p < 0.001).

*Alzheimer’s Disease*
Among males, Alzheimer’s disease occurred in 1 172 of 227 487 Psoriasis patients (0.5 %) compared with 661 of 227 856 controls (0.3 %) (RD 0.2 %, 95 % CI 0.2–0.3; RR 1.78, 95 % CI 1.62–1.95; OR 1.78, 95 % CI 1.62–1.96; p < 0.001). In females, 1 627 of 267 121 Psoriasis patients (0.6 %) versus 965 of 267 531 controls (0.4 %) developed Alzheimer’s disease (RD 0.2 %, 95 % CI 0.2–0.3; RR 1.69, 95 % CI 1.56–1.83; OR 1.69, 95 % CI 1.56–1.83; p < 0.001).

*Transient Ischemic Attack (TIA)*TIA was observed in 4 042 of 224 650 Psoriasis males (1.8 %) compared with 1 769 of 227 484 controls (0.8 %) (RD 1.0 %, 95 % CI 1.0–1.1; RR 2.31, 95 % CI 2.19–2.45; OR 2.34, 95 % CI 2.21–2.47; p < 0.001). In the female cohort, 4 884 of 263 473 Psoriasis patients (1.9 %) versus 2 000 of 267 264 controls (0.7 %) experienced TIA (RD 1.1 %, 95 % CI 1.0–1.2; RR 2.48, 95 % CI 2.35–2.61; OR 2.51, 95 % CI 2.38–2.64; p < 0.001).

*Stroke*
Stroke incidence was 6 599 of 222 195 Psoriasis males (3.0 %) versus 3 970 of 226 219 controls (1.8 %) (RD 1.2 %, 95 % CI 1.1–1.3; RR 1.69, 95 % CI 1.63–1.76; OR 1.71, 95 % CI 1.65–1.78; p < 0.001). Among females, 6 935 of 262 148 Psoriasis patients (2.6 %) suffered stroke compared with 3 559 of 266 376 controls (1.3 %) (RD 1.3 %, 95 % CI 1.2–1.4; RR 1.98, 95 % CI 1.90–2.06; OR 2.01, 95 % CI 1.93–2.09; p < 0.001).

**Sjögren Syndrome**

After 1:1 propensity score matching, the male cohorts consisted of 31 437 patients with Sjögren Syndrome and 31 437 matched controls; the female cohorts comprised 188 145 patients with Sjögren Syndrome and 188 145 matched controls. The characteristics of both cohorts were well balanced on age and sex after matching (standardized differences < 0.01). After matching, the mean current age of male Sjögren Syndrome patients and matched controls was 69.0 years (SD 9.4) in both groups; for females, the mean current age was 67.4 years (SD 9.4) in both Sjögren Syndrome and control cohorts.

*Parkinson’s Disease*In the male cohort, 431 of 30 931 Sjögren Syndrome patients (1.4 %) and 186 of 31 357 controls (0.6 %) developed Parkinson’s disease, yielding a risk difference of 0.8 % (95 % CI 0.6–1.0), a risk ratio of 2.35 (95 % CI 1.98–2.79), and an odds ratio of 2.37 (95 % CI 1.99–2.82); p < 0.001. In the female cohort, 1 320 of 187 003 Sjögren Syndrome patients (0.7 %) versus 620 of 187 841 controls (0.3 %) were diagnosed with Parkinson’s disease (RD 0.4 %, 95 % CI 0.3–0.4; RR 2.14, 95 % CI 1.94–2.35; OR 2.15, 95 % CI 1.95–2.36; p < 0.001).

*Alzheimer’s Disease*Among males, Alzheimer’s disease occurred in 188 of 31 346 Sjögren Syndrome patients (0.6 %) compared with 110 of 31 404 controls (0.4 %) (RD 0.2 %, 95 % CI 0.1–0.4; RR 1.71, 95 % CI 1.35–2.16; OR 1.72, 95 % CI 1.35–2.17; p < 0.001). In females, 1 230 of 187 739 Sjögren Syndrome patients (0.7 %) versus 713 of 187 972 controls (0.4 %) developed Alzheimer’s disease (RD 0.3 %, 95 % CI 0.2–0.3; RR 1.73, 95 % CI 1.58–1.89; OR 1.73, 95 % CI 1.58–1.90; p < 0.001).

*Transient Ischemic Attack (TIA)*
TIA was observed in 785 of 30 466 Sjögren Syndrome males (2.6 %) compared with 255 of 31 371 controls (0.8 %) (RD 1.8 %, 95 % CI 1.6–2.0; RR 3.18, 95 % CI 2.75–3.65; OR 3.23, 95 % CI 2.80–3.72; p < 0.001). In the female cohort, 4 700 of 183 682 Sjögren Syndrome patients (2.6 %) versus 1 464 of 187 770 controls (0.8 %) experienced TIA (RD 1.8 %, 95 % CI 1.7–1.9; RR 3.28, 95 % CI 3.10–3.48; OR 3.34, 95 % CI 3.15–3.55; p < 0.001).

*Stroke*
Stroke incidence was 1 020 of 30 213 Sjögren Syndrome males (3.4 %) versus 570 of 31 173 controls (1.8 %) (RD 1.6 %, 95 % CI 1.3–1.8; RR 1.85, 95 % CI 1.67–2.05; OR 1.88, 95 % CI 1.69–2.08; p < 0.001). Among females, 5 257 of 183 573 Sjögren Syndrome patients (2.9 %) suffered stroke compared with 2 470 of 187 118 controls (1.3 %) (RD 1.5 %, 95 % CI 1.5–1.6; RR 2.17, 95 % CI 2.07–2.28; OR 2.20, 95 % CI 2.10–2.31; p < 0.001).

**Scleroderma**

After 1:1 propensity score matching, the male cohorts consisted of 9 703 patients with Scleroderma and 9 703 matched controls; the female cohorts comprised 44 982 patients with Scleroderma and 44 982 matched controls. The characteristics of both cohorts were well balanced on age and sex after matching (standardized differences < 0.01). After matching, the mean current age of male Scleroderma patients and matched controls was 67.6 years (SD 9.1) in both groups; for females, the mean current age was 67.7 years (SD 9.4) in both Scleroderma and control cohorts.

*Parkinson’s Disease*
In the male cohort, 77 of 9 623 Scleroderma patients (0.8 %) and 52 of 9 684 controls (0.5 %) developed Parkinson’s disease, yielding a risk difference of 0.3 % (95 % CI 0.0–0.5), a risk ratio of 1.49 (95 % CI 1.05–2.12), and an odds ratio of 1.49 (95 % CI 1.05–2.13); p = 0.025. In the female cohort, 198 of 44 803 Scleroderma patients (0.4 %) versus 177 of 44 910 controls (0.4 %) were diagnosed with Parkinson’s disease (RD 0.0 %, 95 % CI –0.0–0.1; RR 1.12, 95 % CI 0.92–1.37; OR 1.12, 95 % CI 0.92–1.38; p = 0.267).

*Alzheimer’s Disease*Among males, Alzheimer’s disease occurred in 44 of 9 682 Scleroderma patients (0.5 %) compared with 22 of 9 692 controls (0.2 %) (RD 0.2 %, 95 % CI 0.1–0.4; RR 2.00, 95 % CI 1.20–3.34; OR 2.01, 95 % CI 1.20–3.35; p = 0.007). In females, 216 of 44 921 Scleroderma patients (0.5 %) versus 196 of 44 935 controls (0.4 %) developed Alzheimer’s disease (RD 0.0 %, 95 % CI –0.0–0.1; RR 1.10, 95 % CI 0.91–1.34; OR 1.10, 95 % CI 0.91–1.34; p = 0.322).

*Transient Ischemic Attack (TIA)*TIA was observed in 153 of 9 513 Scleroderma males (1.6 %) compared with 100 of 9 682 controls (1.0 %) (RD 0.6 %, 95 % CI 0.3–0.9; RR 1.56, 95 % CI 1.21–2.00; OR 1.57, 95 % CI 1.22–2.02; p < 0.001). In the female cohort, 891 of 44 335 Scleroderma patients (2.0 %) versus 352 of 44 885 controls (0.8 %) experienced TIA (RD 1.2 %, 95 % CI 1.1–1.4; RR 2.56, 95 % CI 2.27–2.90; OR 2.60, 95 % CI 2.29–2.94; p < 0.001).

*Stroke*
Stroke incidence was 290 of 9 399 Scleroderma males (3.1 %) versus 180 of 9 639 controls (1.9 %) (RD 1.2 %, 95 % CI 0.8–1.7; RR 1.65, 95 % CI 1.38–1.99; OR 1.67, 95 % CI 1.39–2.02; p < 0.001). Among females, 1 311 of 43 966 Scleroderma patients (3.0 %) suffered stroke compared with 654 of 44 754 controls (1.5 %) (RD 1.5 %, 95 % CI 1.3–1.7; RR 2.04, 95 % CI 1.86–2.24; OR 2.07, 95 % CI 1.89–2.28; p < 0.001).

**Myositis**

After 1:1 propensity score matching, the male cohorts consisted of 184 171 patients with Myositis and 184 171 matched controls; the female cohorts comprised 430 485 patients with Myositis and 430 485 matched controls. The characteristics of both cohorts were well balanced on age and sex after matching (standardized differences < 0.01). After matching, the mean current age of male Myositis patients and matched controls was 66.5 years (SD 9.5) in both groups; for females, the mean current age was 66.3 years (SD 9.4) in both Myositis and control cohorts.

*Parkinson’s Disease*In the male cohort, 1 982 of 182 730 Myositis patients (1.1 %) and 1 112 of 183 831 controls (0.6 %) developed Parkinson’s disease, yielding a risk difference of 0.5 % (95 % CI 0.4–0.6), a risk ratio of 1.79 (95 % CI 1.67–1.93), and an odds ratio of 1.80 (95 % CI 1.67–1.94); p < 0.001. In the female cohort, 3 620 of 428 821 Myositis patients (0.8 %) versus 1 550 of 430 099 controls (0.4 %) were diagnosed with Parkinson’s disease (RD 0.4 %, 95 % CI 0.3–0.4; RR 2.34, 95 % CI 2.21–2.49; OR 2.35, 95 % CI 2.22–2.50; p < 0.001).

*Alzheimer’s Disease*
Among males, Alzheimer’s disease occurred in 1 071 of 183 834 Myositis patients (0.6 %) compared with 645 of 184 090 controls (0.4 %) (RD 0.2 %, 95 % CI 0.2–0.3; RR 1.66, 95 % CI 1.51–1.83; OR 1.67, 95 % CI 1.51–1.84; p < 0.001). In females, 3 354 of 429 888 Myositis patients (0.8 %) versus 1 730 of 430 319 controls (0.4 %) developed Alzheimer’s disease (RD 0.4 %, 95 % CI 0.4–0.4; RR 1.94, 95 % CI 1.83–2.06; OR 1.95, 95 % CI 1.84–2.07; p < 0.001).

*Transient Ischemic Attack (TIA)*TIA was observed in 5 584 of 179 895 Myositis males (3.1 %) compared with 1 853 of 183 823 controls (1.0 %) (RD 2.1 %, 95 % CI 2.0–2.2; RR 3.08, 95 % CI 2.92–3.24; OR 3.15, 95 % CI 2.98–3.32; p < 0.001). In the female cohort, 14 064 of 421 948 Myositis patients (3.3 %) versus 4 334 of 429 818 controls (1.0 %) experienced TIA (RD 2.3 %, 95 % CI 2.3–2.4; RR 3.30, 95 % CI 3.20–3.43; OR 3.39, 95 % CI 3.27–3.51; p < 0.001).

*Stroke*
Stroke incidence was 8 349 of 178 005 Myositis males (4.7 %) versus 3 885 of 183 070 controls (2.1 %) (RD 2.6 %, 95 % CI 2.4–2.7; RR 2.21, 95 % CI 2.13–2.30; OR 2.27, 95 % CI 2.18–2.36; p < 0.001). Among females, 17 909 of 421 018 Myositis patients (4.3 %) suffered stroke compared with 7 404 of 428 918 controls (1.7 %) (RD 2.6 %, 95 % CI 2.5–2.7; RR 2.46, 95 % CI 2.40–2.53; OR 2.53, 95 % CI 2.46–2.60; p < 0.001).

**Celiac Disease**

After 1:1 propensity score matching, the male cohorts consisted of 26 409 patients with Celiac disease and 26 409 matched controls; the female cohorts comprised 66 544 patients with Celiac disease and 66 544 matched controls. The characteristics of both cohorts were well balanced on age and sex after matching (standardized differences < 0.01). After matching, the mean current age of male Celiac disease patients and matched controls was 66.7 years (SD 9.7) in both groups; for females, the mean current age was 65.2 years (SD 9.7) in both Celiac disease and control cohorts.

*Parkinson’s Disease*
In the male cohort, 254 of 26 172 Celiac disease patients (1.0 %) and 136 of 26 329 controls (0.5 %) developed Parkinson’s disease, yielding a risk difference of 0.5 % (95 % CI 0.3–0.6), a risk ratio of 1.88 (95 % CI 1.53–2.31), and an odds ratio of 1.89 (95 % CI 1.53–2.33); p < 0.001. In the female cohort, 357 of 66 273 Celiac disease patients (0.5 %) versus 165 of 66 467 controls (0.2 %) were diagnosed with Parkinson’s disease (RD 0.3 %, 95 % CI 0.2–0.4; RR 2.17, 95 % CI 1.81–2.61; OR 2.18, 95 % CI 1.81–2.62; p < 0.001).

*Alzheimer’s Disease*
Among males, Alzheimer’s disease occurred in 174 of 26 317 Celiac disease patients (0.7 %) compared with 71 of 26 389 controls (0.3 %) (RD 0.4 %, 95 % CI 0.3–0.5; RR 2.46, 95 % CI 1.87–3.24; OR 2.47, 95 % CI 1.87–3.25; p < 0.001). In females, 436 of 66 373 Celiac disease patients (0.7 %) versus 214 of 66 475 controls (0.3 %) developed Alzheimer’s disease (RD 0.4 %, 95 % CI 0.3–0.4; RR 2.04, 95 % CI 1.73–2.40; OR 2.05, 95 % CI 1.74–2.41; p < 0.001).

*Transient Ischemic Attack (TIA)*
TIA was observed in 515 of 25 955 Celiac disease males (2.0 %) compared with 192 of 26 347 controls (0.7 %) (RD 1.3 %, 95 % CI 1.1–1.5; RR 2.72, 95 % CI 2.31–3.21; OR 2.76, 95 % CI 2.33–3.26; p < 0.001). In the female cohort, 1 262 of 65 548 Celiac disease patients (1.9 %) versus 497 of 66 426 controls (0.7 %) experienced TIA (RD 1.2 %, 95 % CI 1.1–1.3; RR 2.57, 95 % CI 2.32–2.85; OR 2.60, 95 % CI 2.35–2.89; p < 0.001).

*Stroke*
Stroke incidence was 705 of 25 774 Celiac disease males (2.7 %) versus 457 of 26 183 controls (1.7 %) (RD 1.0 %, 95 % CI 0.7–1.2; RR 1.57, 95 % CI 1.40–1.76; OR 1.58, 95 % CI 1.41–1.78; p < 0.001). Among females, 1 563 of 65 398 Celiac disease patients (2.4 %) suffered stroke compared with 830 of 66 230 controls (1.3 %) (RD 1.1 %, 95 % CI 1.0–1.3; RR 1.91, 95 % CI 1.75–2.07; OR 1.93, 95 % CI 1.77–2.10; p < 0.001).

**Rheumatoid Arthritis (RA)**

After 1:1 propensity score matching, the male cohorts consisted of 156 935 patients with RA and 156 935 matched controls; the female cohorts comprised 439 134 patients with RA and 439 134 matched controls. Characteristics were well balanced on age and sex after matching (standardized differences < 0.01). After matching, the mean current age of male RA patients and matched controls was 69.7 years (SD 9.1); for females, the mean current age was 68.4 years (SD 9.4).

*Parkinson’s Disease*In the male cohort, 1 414 of 155 343 RA patients (0.9 %) and 1 026 of 156 478 controls (0.7 %) developed Parkinson’s disease, yielding a risk difference of 0.3 % (95 % CI 0.2–0.3), a risk ratio of 1.39 (95 % CI 1.28–1.50), and an odds ratio of 1.39 (95 % CI 1.28–1.50); p < 0.001.
In the female cohort, 2 474 of 436 726 RA patients (0.6 %) versus 1 535 of 438 448 controls (0.4 %) were diagnosed with Parkinson’s disease (RD 0.2 %, 95 % CI 0.2–0.2; RR 1.62, 95 % CI 1.52–1.72; OR 1.62, 95 % CI 1.52–1.73; p < 0.001).

*Alzheimer’s Disease*
In the male cohort, 1 075 of 156 336 RA patients (0.7 %) compared with 587 of 156 797 controls (0.4 %) developed Alzheimer’s disease (RD 0.3 %, 95 % CI 0.2–0.4; RR 1.84, 95 % CI 1.66–2.03; OR 1.84, 95 % CI 1.67–2.04; p < 0.001).
In the female cohort, 3 312 of 437 527 RA patients (0.8 %) versus 1 912 of 438 664 controls (0.4 %) were diagnosed with Alzheimer’s disease (RD 0.4 %, 95 % CI 0.3–0.4; RR 1.74, 95 % CI 1.64–1.84; OR 1.74, 95 % CI 1.65–1.84; p < 0.001).

*Transient Ischemic Attack (TIA)*
In the male cohort, 3 206 of 153 835 RA patients (2.1 %) versus 1 446 of 156 522 controls (0.9 %) experienced TIA (RD 1.2 %, 95 % CI 1.1–1.2; RR 2.26, 95 % CI 2.12–2.40; OR 2.28, 95 % CI 2.15–2.43; p < 0.001).
In the female cohort, 9 711 of 430 863 RA patients (2.3 %) versus 3 631 of 438 314 controls (0.8 %) experienced TIA (RD 1.4 %, 95 % CI 1.4–1.5; RR 2.72, 95 % CI 2.62–2.83; OR 2.76, 95 % CI 2.66–2.87; p < 0.001).

*Stroke*
In the male cohort, 5 675 of 151 125 RA patients (3.8 %) versus 3 037 of 155 522 controls (2.0 %) suffered stroke (RD 1.8 %, 95 % CI 1.7–1.9; RR 1.92, 95 % CI 1.84–2.01; OR 1.96, 95 % CI 1.87–2.05; p < 0.001).
In the female cohort, 14 678 of 426 079 RA patients (3.4 %) versus 6 184 of 436 723 controls (1.4 %) suffered stroke (RD 2.0 %, 95 % CI 2.0–2.1; RR 2.43, 95 % CI 2.36–2.51; OR 2.48, 95 % CI 2.41–2.56; p < 0.001).

**Type 1 Diabetes Mellitus (T1DM)**

After 1:1 propensity score matching, the male cohorts consisted of 291 708 patients with Type 1 Diabetes Mellitus and 291 708 matched controls; the female cohorts comprised 262 372 patients with Type 1 Diabetes Mellitus and 262 372 matched controls. The cohorts were well balanced on age and sex after matching (standardized differences < 0.01). After matching, the mean current age of male T1DM patients and matched controls was 68.0 years (SD 9.5) in both groups; for females, the mean current age was 68.3 years (SD 9.6) in both T1DM and control cohorts.

*Parkinson’s Disease*
In the male cohort, 3 139 of 289 573 T1DM patients (1.1 %) and 1 804 of 291 048 controls (0.6 %) developed Parkinson’s disease, yielding a risk difference of 0.5 % (95 % CI 0.4–0.5), a risk ratio of 1.75 (95 % CI 1.65–1.85), and an odds ratio of 1.76 (95 % CI 1.66–1.86); p < 0.001. In the female cohort, 2 174 of 261 049 T1DM patients (0.8 %) versus 1 064 of 262 025 controls (0.4 %) were diagnosed with Parkinson’s disease (RD 0.4 %, 95 % CI 0.4–0.5; RR 2.05, 95 % CI 1.91–2.21; OR 2.06, 95 % CI 1.91–2.22; p < 0.001).

*Alzheimer’s Disease*
Among males, Alzheimer’s disease occurred in 2 393 of 290 788 T1DM patients (0.8 %) compared with 1 027 of 291 542 controls (0.4 %) (RD 0.5 %, 95 % CI 0.4–0.5; RR 2.34, 95 % CI 2.17–2.51; OR 2.35, 95 % CI 2.18–2.53; p < 0.001). In females, 2 913 of 261 295 T1DM patients (1.1 %) versus 1 229 of 262 166 controls (0.5 %) developed Alzheimer’s disease (RD 0.6 %, 95 % CI 0.6–0.7; RR 2.38, 95 % CI 2.23–2.54; OR 2.39, 95 % CI 2.24–2.56; p < 0.001).

*Transient Ischemic Attack (TIA)*TIA was observed in 8 633 of 285 249 T1DM males (3.0 %) compared with 2 835 of 291 085 controls (1.0 %) (RD 2.1 %, 95 % CI 2.0–2.1; RR 3.11, 95 % CI 2.98–3.24; OR 3.17, 95 % CI 3.04–3.31; p < 0.001). In the female cohort, 9 295 of 255 794 T1DM patients (3.6 %) versus 2 527 of 261 851 controls (1.0 %) experienced TIA (RD 2.7 %, 95 % CI 2.6–2.8; RR 3.77, 95 % CI 3.61–3.93; OR 3.87, 95 % CI 3.70–4.05; p < 0.001).

*Stroke*
Stroke incidence was 18 031 of 276 130 T1DM males (6.5 %) versus 5 969 of 289 542 controls (2.1 %) (RD 4.5 %, 95 % CI 4.4–4.6; RR 3.17, 95 % CI 3.08–3.26; OR 3.32, 95 % CI 3.22–3.42; p < 0.001). Among females, 17 232 of 248 978 T1DM patients (6.9 %) suffered stroke compared with 4 162 of 261 094 controls (1.6 %) (RD 5.3 %, 95 % CI 5.2–5.4; RR 4.34, 95 % CI 4.20–4.49; OR 4.59, 95 % CI 4.44–4.75; p < 0.001).

**Autoimmune Thyroiditis**

After 1:1 propensity score matching, the male cohorts consisted of 39 809 patients with Autoimmune Thyroiditis and 39 809 matched controls; the female cohorts comprised 229 736 patients with Autoimmune Thyroiditis and 229 736 matched controls. The characteristics of both cohorts were well balanced on age and sex after matching (standardized differences < 0.01). After matching, the mean current age of male Autoimmune Thyroiditis patients and matched controls was 66.3 years (SD 9.4) in both groups; for females, the mean current age was 64.6 years (SD 9.3) in both Autoimmune Thyroiditis and control cohorts.

*Parkinson’s Disease*In the male cohort, 361 of 39 429 Autoimmune Thyroiditis patients (0.9 %) and 162 of 39 716 controls (0.4 %) developed Parkinson’s disease, yielding a risk difference of 0.5 % (95 % CI 0.4–0.6), a risk ratio of 2.25 (95 % CI 1.87–2.70), and an odds ratio of 2.26 (95 % CI 1.87–2.72); p < 0.001. In the female cohort, 997 of 228 880 Autoimmune Thyroiditis patients (0.4 %) versus 566 of 229 459 controls (0.2 %) were diagnosed with Parkinson’s disease (RD 0.2 %, 95 % CI 0.2–0.2; RR 1.77, 95 % CI 1.59–1.96; OR 1.77, 95 % CI 1.60–1.96; p < 0.001).

*Alzheimer’s Disease*
Among males, Alzheimer’s disease occurred in 261 of 39 640 Autoimmune Thyroiditis patients (0.7 %) compared with 104 of 39 782 controls (0.3 %) (RD 0.4 %, 95 % CI 0.3–0.5; RR 2.52, 95 % CI 2.01–3.17; OR 2.53, 95 % CI 2.01–3.18; p < 0.001). In females, 1 120 of 229 193 Autoimmune Thyroiditis patients (0.5 %) versus 589 of 229 575 controls (0.3 %) developed Alzheimer’s disease (RD 0.2 %, 95 % CI 0.2–0.3; RR 1.91, 95 % CI 1.72–2.10; OR 1.91, 95 % CI 1.73–2.11; p < 0.001).

*Transient Ischemic Attack (TIA)*
TIA was observed in 785 of 38 947 Autoimmune Thyroiditis males (2.0 %) compared with 267 of 39 736 controls (0.7 %) (RD 1.3 %, 95 % CI 1.2–1.4; RR 3.00, 95 % CI 2.61–3.45; OR 3.04, 95 % CI 2.65–3.50; p < 0.001). In the female cohort, 3 815 of 226 022 Autoimmune Thyroiditis patients (1.7 %) versus 1 450 of 229 370 controls (0.6 %) experienced TIA (RD 1.1 %, 95 % CI 1.0–1.1; RR 2.67, 95 % CI 2.51–2.84; OR 2.70, 95 % CI 2.54–2.87; p < 0.001).

*Stroke*
Stroke incidence was 1 095 of 38 491 Autoimmune Thyroiditis males (2.8 %) versus 602 of 39 497 controls (1.5 %) (RD 1.3 %, 95 % CI 1.1–1.5; RR 1.87, 95 % CI 1.69–2.06; OR 1.89, 95 % CI 1.71–2.09; p < 0.001). Among females, 4 398 of 225 574 Autoimmune Thyroiditis patients (1.9 %) suffered stroke compared with 2 611 of 228 724 controls (1.1 %) (RD 0.8 %, 95 % CI 0.7–0.9; RR 1.71, 95 % CI 1.63–1.79; OR 1.72, 95 % CI 1.64–1.81; p < 0.001).

**Lupus erythematosus**

After 1:1 propensity score matching, the male cohorts consisted of 9 586 patients with Lupus erythematosus and 9 586 matched controls; the female cohorts comprised 44 703 patients with Lupus erythematosus and 44 703 matched controls. The characteristics of both cohorts were well balanced on age and sex after matching (standardized differences < 0.01). After matching, the mean current age of male Lupus erythematosus patients and matched controls was 66.6 years (SD 9.3) in both groups; for females, the mean current age was 65.2 years (SD 9.3) in both Lupus erythematosus and control cohorts.

*Parkinson’s Disease*
In the male cohort, 85 of 9 537 Lupus erythematosus patients (0.9 %) and 36 of 9 559 controls (0.4 %) developed Parkinson’s disease, yielding a risk difference of 0.5 % (95 % CI 0.3–0.7), a risk ratio of 2.37 (95 % CI 1.60–3.49), and an odds ratio of 2.38 (95 % CI 1.61–3.52); p < 0.001. In the female cohort, 197 of 44 526 Lupus erythematosus patients (0.4 %) versus 111 of 44 656 controls (0.2 %) were diagnosed with Parkinson’s disease (RD 0.2 %, 95 % CI 0.1–0.3; RR 1.78, 95 % CI 1.41–2.25; OR 1.78, 95 % CI 1.42–2.25; p < 0.001).

*Alzheimer’s Disease*
Among males, Alzheimer’s disease occurred in 58 of 9 558 Lupus erythematosus patients (0.6 %) compared with 22 of 9 577 controls (0.2 %) (RD 0.4 %, 95 % CI 0.2–0.6; RR 2.65, 95 % CI 1.62–4.31; OR 2.65, 95 % CI 1.62–4.34; p < 0.001). In the female cohort, 235 of 44 605 Lupus erythematosus patients (0.5 %) versus 158 of 44 666 controls (0.4 %) developed Alzheimer’s disease (RD 0.2 %, 95 % CI 0.1–0.3; RR 1.49, 95 % CI 1.22–1.82; OR 1.49, 95 % CI 1.22–1.82; p < 0.001).

*Transient Ischemic Attack (TIA)*
TIA was observed in 223 of 9 359 Lupus erythematosus males (2.4 %) compared with 85 of 9 556 controls (0.9 %) (RD 1.5 %, 95 % CI 1.1–1.9; RR 2.68, 95 % CI 2.09–3.43; OR 2.72, 95 % CI 2.12–3.50; p < 0.001). In the female cohort, 1 273 of 43 576 Lupus erythematosus patients (2.9 %) versus 291 of 44 619 controls (0.7 %) experienced TIA (RD 2.2 %, 95 % CI 2.1–2.4; RR 4.48, 95 % CI 3.95–5.08; OR 4.59, 95 % CI 4.03–5.21; p < 0.001).

*Stroke*
Stroke incidence was 413 of 9 182 Lupus erythematosus males (4.5 %) versus 156 of 9 515 controls (1.6 %) (RD 2.9 %, 95 % CI 2.4–3.4; RR 2.83, 95 % CI 2.35–3.41; OR 2.83, 95 % CI 2.34–3.41; p < 0.001). Among females, 2 052 of 43 034 Lupus erythematosus patients (4.8 %) suffered stroke compared with 585 of 44 496 controls (1.3 %) (RD 3.5 %, 95 % CI 3.2–3.7; RR 3.62, 95 % CI 3.31–3.97; OR 3.76, 95 % CI 3.42–4.13; p < 0.001).

**Ulcerative Colitis**

After 1:1 propensity score matching, the male cohorts consisted of 113 439 patients with Ulcerative Colitis and 113 439 matched controls; the female cohorts comprised 131 091 patients with Ulcerative Colitis and 131 091 matched controls. The characteristics of both cohorts were well balanced on age and sex after matching (standardized differences < 0.01). After matching, the mean current age of male Ulcerative Colitis patients and matched controls was 67.0 years (SD 9.6) in both groups; for females, the mean current age was 67.0 years (SD 9.6) in both Ulcerative Colitis and control cohorts.

*Parkinson’s Disease*
In the male cohort, 834 of 112 433 Ulcerative Colitis patients (0.7 %) and 648 of 113 146 controls (0.6 %) developed Parkinson’s disease, yielding a risk difference of 0.2 % (95 % CI 0.1–0.2), a risk ratio of 1.30 (95 % CI 1.17–1.44), and an odds ratio of 1.30 (95 % CI 1.17–1.44); p < 0.001. In the female cohort, 611 of 130 384 Ulcerative Colitis patients (0.5 %) versus 392 of 130 893 controls (0.3 %) were diagnosed with Parkinson’s disease (RD 0.2 %, 95 % CI 0.1–0.2; RR 1.56, 95 % CI 1.38–1.78; OR 1.57, 95 % CI 1.38–1.78; p < 0.001).

*Alzheimer’s Disease*
Among males, Alzheimer’s disease occurred in 603 of 113 079 Ulcerative Colitis patients (0.5 %) compared with 333 of 113 356 controls (0.3 %) (RD 0.2 %, 95 % CI 0.2–0.3; RR 1.82, 95 % CI 1.59–2.08; OR 1.82, 95 % CI 1.59–2.08; p < 0.001). In females, 792 of 130 559 Ulcerative Colitis patients (0.6 %) versus 466 of 130 947 controls (0.4 %) developed Alzheimer’s disease (RD 0.2 %, 95 % CI 0.2–0.3; RR 1.70, 95 % CI 1.52–1.91; OR 1.71, 95 % CI 1.52–1.92; p < 0.001).

*Transient Ischemic Attack (TIA)*
TIA was observed in 1 741 of 111 566 male Ulcerative Colitis patients (1.6 %) compared with 818 of 113 179 controls (0.7 %) (RD 0.8 %, 95 % CI 0.7–0.9; RR 2.16, 95 % CI 1.99–2.35; OR 2.18, 95 % CI 2.00–2.37; p < 0.001). In the female cohort, 2 390 of 128 472 Ulcerative Colitis patients (1.9 %) versus 880 of 130 835 controls (0.7 %) experienced TIA (RD 1.2 %, 95 % CI 1.1–1.3; RR 2.76, 95 % CI 2.56–2.99; OR 2.80, 95 % CI 2.59–2.94; p < 0.001).

*Stroke*
Stroke incidence was 2 955 of 109 906 male Ulcerative Colitis patients (2.7 %) versus 1 895 of 112 557 controls (1.7 %) (RD 1.0 %, 95 % CI 0.9–1.1; RR 1.60, 95 % CI 1.51–1.69; OR 1.61, 95 % CI 1.52–1.71; p < 0.001). Among females, 3 459 of 127 266 Ulcerative Colitis patients (2.7 %) suffered stroke compared with 1 640 of 130 381 controls (1.3 %) (RD 1.5 %, 95 % CI 1.4–1.6; RR 2.16, 95 % CI 2.04–2.29; OR 2.19, 95 % CI 2.07–2.33; p < 0.001).

**Crohn’s Disease**

*Results*

After 1:1 propensity score matching, the male cohorts consisted of 79 240 patients with Crohn’s disease and 79 240 matched controls; the female cohorts comprised 106 711 patients with Crohn’s disease and 106 711 matched controls. The characteristics of both cohorts were well balanced on age and sex after matching (standardized differences < 0.01). After matching, the mean current age of male Crohn’s disease patients and matched controls was 65.7 years (SD 9.7) in both groups; for females, the mean current age was 65.6 years (SD 9.6) in both Crohn’s disease and control cohorts.

*Parkinson’s Disease*
In the male cohort, 569 of 78 693 Crohn’s disease patients (0.7 %) and 389 of 79 081 controls (0.5 %) developed Parkinson’s disease, yielding a risk difference of 0.2 % (95 % CI 0.1–0.3), a risk ratio of 1.47 (95 % CI 1.29–1.67), and an odds ratio of 1.47 (95 % CI 1.29–1.68); p < 0.001. In the female cohort, 499 of 106 301 Crohn’s disease patients (0.5 %) versus 321 of 106 568 controls (0.3 %) were diagnosed with Parkinson’s disease (RD 0.2 %, 95 % CI 0.1–0.2; RR 1.56, 95 % CI 1.36–1.79; OR 1.56, 95 % CI 1.36–1.79; p < 0.001).

*Alzheimer’s Disease*Among males, Alzheimer’s disease occurred in 364 of 79 079 Crohn’s disease patients (0.5 %) compared with 212 of 79 194 controls (0.3 %) (RD 0.2 %, 95 % CI 0.1–0.3; RR 1.72, 95 % CI 1.45–2.04; OR 1.72, 95 % CI 1.45–2.04; p < 0.001). In females, 590 of 106 441 Crohn’s disease patients (0.6 %) versus 340 of 106 632 controls (0.3 %) developed Alzheimer’s disease (RD 0.3 %, 95 % CI 0.2–0.3; RR 1.74, 95 % CI 1.52–1.99; OR 1.74, 95 % CI 1.52–1.99; p < 0.001).

*Transient Ischemic Attack (TIA)*TIA was observed in 1 342 of 78 293 Crohn’s disease males (1.7 %) compared with 658 of 79 096 controls (0.8 %) (RD 0.9 %, 95 % CI 0.8–1.0; RR 2.06, 95 % CI 1.88–2.26; OR 2.08, 95 % CI 1.89–2.28; p < 0.001). In the female cohort, 1 987 of 105 306 Crohn’s disease patients (1.9 %) versus 860 of 106 546 controls (0.8 %) experienced TIA (RD 1.1 %, 95 % CI 1.0–1.2; RR 2.34, 95 % CI 2.16–2.53; OR 2.36, 95 % CI 2.17–2.67; p < 0.001).

*Stroke*
Stroke incidence was 2 236 of 77 583 Crohn’s disease males (2.9 %) versus 1 371 of 78 684 controls (1.7 %) (RD 1.2 %, 95 % CI 1.0–1.3; RR 1.65, 95 % CI 1.55–1.77; OR 1.67, 95 % CI 1.56–1.79; p < 0.001). Among females, 2 899 of 104 595 Crohn’s disease patients (2.8 %) suffered stroke compared with 1 434 of 106 249 controls (1.3 %) (RD 1.5 %, 95 % CI 1.4–1.6; RR 2.05, 95 % CI 1.93–2.19; OR 2.08, 95 % CI 1.95–2.22; p < 0.001).

**EXPERIMENT 1B**

**Chronic Inflammatory Demyelinating Polyneuropathy (CIDP)**
After 1:1 propensity score matching (matched on current age and diseases of the circulatory system), the male cohorts consisted of 15,165 patients with CIDP and 15,165 matched controls; the female cohorts comprised 10,523 patients with CIDP and 10,523 matched controls. After matching, the mean current age of male CIDP patients and matched controls was 70.0 years (SD 9.0) in both groups; for females, the mean current age was 67.8 years (SD 9.5) in both cohorts.

*Parkinson’s Disease*
In the male cohort, 232 of 14,881 CIDP patients (1.6%) and 175 of 15,097 controls (1.2%) developed Parkinson’s disease, yielding a risk difference (RD) of 0.4% (95% CI 0.1–0.7), a risk ratio (RR) of 1.34 (95% CI 1.11–1.63), and an odds ratio (OR) of 1.35 (95% CI 1.11–1.64); p = 0.003. In the female cohort, 102 of 10,376 CIDP patients (1.0%) versus 72 of 10,496 controls (0.7%) were diagnosed with Parkinson’s disease (RD 0.3%, 95% CI 0.1–0.5; RR 1.43, 95% CI 1.06–1.93; OR 1.44, 95% CI 1.06–1.95; p = 0.018).

*Alzheimer’s Disease*
Among males, Alzheimer’s disease occurred in 122 of 15,104 CIDP patients (0.8%) compared with 107 of 15,135 controls (0.7%) (RD 0.1%, 95% CI −0.1–0.3; RR 1.14, 95% CI 0.88–1.48; OR 1.14, 95% CI 0.88–1.48; p = 0.312). In females, 70 of 10,493 CIDP patients (0.7%) versus 81 of 10,509 controls (0.8%) developed Alzheimer’s disease (RD −0.1%, 95% CI −0.3–0.1; RR 0.87, 95% CI 0.63–1.19; OR 0.86, 95% CI 0.63–1.19; p = 0.374).

*Transient Ischemic Attack (TIA)*TIA was observed in 373 of 14,747 CIDP males (2.5%) compared with 288 of 15,033 controls (1.9%) (RD 0.6%, 95% CI 0.3–0.9; RR 1.32, 95% CI 1.13–1.54; OR 1.33, 95% CI 1.14–1.55; p < 0.001). In the female cohort, 265 of 10,215 CIDP patients (2.6%) versus 218 of 10,435 controls (2.1%) experienced TIA (RD 0.5%, 95% CI 0.1–0.9; RR 1.24, 95% CI 1.04–1.48; OR 1.25, 95% CI 1.04–1.50; p = 0.016).

*Stroke*
Stroke incidence was 599 of 14,402 CIDP males (4.2%) versus 518 of 14,618 controls (3.5%) (RD 0.6%, 95% CI 0.2–1.1; RR 1.17, 95% CI 1.05–1.32; OR 1.18, 95% CI 1.05–1.33; p = 0.006). Among females, 443 of 10,027 CIDP patients (4.4%) suffered stroke compared with 314 of 10,178 controls (3.1%) (RD 1.3%, 95% CI 0.8–1.9; RR 1.43, 95% CI 1.24–1.65; OR 1.45, 95% CI 1.25–1.68; p < 0.001).

**Guillain–Barré Syndrome (GBS)**After 1:1 propensity score matching (matched on current age and diseases of the circulatory system), the male cohorts consisted of 15,958 patients with GBS and 15,958 matched controls; the female cohorts comprised 13,330 patients with GBS and 13,330 matched controls. After matching, the mean current age of male GBS patients and matched controls was 68.3 years (SD 9.5) in both groups; for females, the mean current age was 67.3 years (SD 9.6) in both cohorts.

*Parkinson’s Disease*
In the male cohort, 151 of 15,753 GBS patients (1.0%) and 140 of 15,906 controls (0.9%) developed Parkinson’s disease, yielding a risk difference (RD) of 0.1% (95% CI −0.1–0.3), a risk ratio (RR) of 1.09 (95% CI 0.87–1.37), and an odds ratio (OR) of 1.09 (95% CI 0.87–1.37); p = 0.465. In the female cohort, 102 of 13,202 GBS patients (0.8%) versus 61 of 13,299 controls (0.5%) were diagnosed with Parkinson’s disease (RD 0.3%, 95% CI 0.1–0.5; RR 1.68, 95% CI 1.23–2.31; OR 1.69, 95% CI 1.23–2.32; p = 0.001).

*Alzheimer’s Disease*Among males, Alzheimer’s disease occurred in 74 of 15,926 GBS patients (0.5%) compared with 83 of 15,934 controls (0.5%) (RD −0.1%, 95% CI −0.2–0.1; RR 0.89, 95% CI 0.65–1.22; OR 0.89, 95% CI 0.65–1.22; p = 0.473). In females, 74 of 13,290 GBS patients (0.6%) versus 90 of 13,303 controls (0.7%) developed Alzheimer’s disease (RD −0.1%, 95% CI −0.3–0.1; RR 0.82, 95% CI 0.61–1.12; OR 0.82, 95% CI 0.60–1.12; p = 0.212).

*Transient Ischemic Attack (TIA)*
TIA was observed in 304 of 15,564 GBS males (2.0%) compared with 325 of 15,832 controls (2.1%) (RD −0.1%, 95% CI −0.4–0.2; RR 0.95, 95% CI 0.81–1.11; OR 0.95, 95% CI 0.81–1.11; p = 0.529). In the female cohort, 302 of 13,009 GBS patients (2.3%) versus 274 of 13,204 controls (2.1%) experienced TIA (RD 0.2%, 95% CI −0.1–0.6; RR 1.12, 95% CI 0.95–1.32; OR 1.12, 95% CI 0.95–1.32; p = 0.174).

*Stroke*
Stroke incidence was 623 of 15,005 GBS males (4.2%) versus 511 of 15,412 controls (3.3%) (RD 0.8%, 95% CI 0.4–1.3; RR 1.25, 95% CI 1.12–1.40; OR 1.26, 95% CI 1.12–1.42; p < 0.001). Among females, 514 of 12,622 GBS patients (4.1%) suffered stroke compared with 359 of 12,958 controls (2.8%) (RD 1.3%, 95% CI 0.9–1.7; RR 1.47, 95% CI 1.28–1.70; OR 1.49, 95% CI 1.29–1.72; p < 0.001).

**Myasthenia Gravis (MG)**
After 1:1 propensity score matching, the male cohorts consisted of 30,747 patients with myasthenia gravis and 30,747 matched controls; the female cohorts comprised 31,455 patients with myasthenia gravis and 31,455 matched controls. Post-matching, current age was identical between cases and controls (males: 71.3 years [SD 9.1] in both groups; females: 68.6 years [SD 9.8] in both groups; standardized differences < 0.001).

*Parkinson’s Disease*In the male cohort, 522 of 30,102 MG patients (1.7 %) and 350 of 30,609 controls (1.1 %) developed Parkinson’s disease, yielding RD 0.6 % (95 % CI 0.4–0.8), RR 1.52 (95 % CI 1.33–1.74), and OR 1.53 (95 % CI 1.33–1.75); p < 0.001. In the female cohort, 347 of 31,081 MG patients (1.1 %) versus 170 of 31,380 controls (0.5 %) were diagnosed with Parkinson’s disease (RD 0.6 %, 95 % CI 0.4–0.7; RR 2.06, 95 % CI 1.72–2.48; OR 2.07, 95 % CI 1.72–2.49; p < 0.001).

*Alzheimer’s Disease*Among males, Alzheimer’s disease occurred in 230 of 30,625 MG patients (0.8 %) compared with 254 of 30,690 controls (0.8 %) (RD −0.1 %, 95 % CI −0.2–0.1; RR 0.91, 95 % CI 0.76–1.08; OR 0.91, 95 % CI 0.76–1.08; p = 0.284). In females, 224 of 31,341 MG patients (0.7 %) versus 232 of 31,395 controls (0.7 %) developed Alzheimer’s disease (RD 0.0 %, 95 % CI −0.2–0.1; RR 0.97, 95 % CI 0.81–1.16; OR 0.97, 95 % CI 0.80–1.16; p = 0.721).

*Transient Ischemic Attack (TIA)*TIA was observed in 699 of 29,767 MG males (2.3 %) compared with 562 of 30,518 controls (1.8 %) (RD 0.5 %, 95 % CI 0.3–0.7; RR 1.28, 95 % CI 1.14–1.42; OR 1.28, 95 % CI 1.15–1.43; p < 0.001). In the female cohort, 744 of 30,411 MG patients (2.4 %) versus 522 of 31,198 controls (1.7 %) experienced TIA (RD 0.8 %, 95 % CI 0.5–1.0; RR 1.46, 95 % CI 1.31–1.63; OR 1.47, 95 % CI 1.32–1.65; p < 0.001).

*Stroke*
Stroke incidence was 1,137 of 29,014 MG males (3.9 %) versus 1,045 of 29,815 controls (3.5 %) (RD 0.4 %, 95 % CI 0.1–0.7; RR 1.12, 95 % CI 1.03–1.21; OR 1.12, 95 % CI 1.03–1.22; p = 0.008). Among females, 1,044 of 29,947 MG patients (3.5 %) suffered stroke compared with 848 of 30,705 controls (2.8 %) (RD 0.7 %, 95 % CI 0.4–1.0; RR 1.26, 95 % CI 1.15–1.38; OR 1.27, 95 % CI 1.16–1.39; p < 0.001).

**Multiple Sclerosis (MS)**After 1:1 propensity score matching (matched on current age and diseases of the circulatory system), the male cohorts consisted of 54,379 patients with MS and 54,379 matched controls; the female cohorts comprised 152,608 patients with MS and 152,608 matched controls. After matching, the mean current age of male MS patients and matched controls was 64.7 years (SD 9.1) in both groups; for females, the mean current age was 64.4 years (SD 9.0) in both cohorts.

*Parkinson’s Disease*In the male cohort, 544 of 53,688 MS patients (1.0%) and 331 of 54,266 controls (0.6%) developed Parkinson’s disease, yielding a risk difference (RD) of 0.4% (95% CI 0.3–0.5), a risk ratio (RR) of 1.66 (95% CI 1.45–1.90), and an odds ratio (OR) of 1.67 (95% CI 1.45–1.91); p < 0.001. In the female cohort, 981 of 151,793 MS patients (0.6%) versus 517 of 152,406 controls (0.3%) were diagnosed with Parkinson’s disease (RD 0.3%, 95% CI 0.3–0.4; RR 1.90, 95% CI 1.71–2.12; OR 1.91, 95% CI 1.72–2.13; p < 0.001).

*Alzheimer’s Disease*
Among males, Alzheimer’s disease occurred in 258 of 54,218 MS patients (0.5%) compared with 169 of 54,334 controls (0.3%) (RD 0.2%, 95% CI 0.1–0.2; RR 1.53, 95% CI 1.26–1.86; OR 1.53, 95% CI 1.26–1.86; p < 0.001). In females, 765 of 152,312 MS patients (0.5%) versus 520 of 152,487 controls (0.3%) developed Alzheimer’s disease (RD 0.2%, 95% CI 0.1–0.2; RR 1.47, 95% CI 1.32–1.64; OR 1.47, 95% CI 1.32–1.65; p < 0.001).

*Transient Ischemic Attack (TIA)*
TIA was observed in 916 of 53,488 MS males (1.7%) compared with 686 of 54,127 controls (1.3%) (RD 0.4%, 95% CI 0.3–0.6; RR 1.35, 95% CI 1.23–1.49; OR 1.36, 95% CI 1.23–1.50; p < 0.001). In the female cohort, 2,843 of 150,338 MS patients (1.9%) versus 1,897 of 151,950 controls (1.2%) experienced TIA (RD 0.6%, 95% CI 0.6–0.7; RR 1.52, 95% CI 1.43–1.61; OR 1.52, 95% CI 1.44–1.62; p < 0.001).

Stroke
Stroke incidence was 1,894 of 52,200 MS males (3.6%) versus 1,313 of 53,312 controls (2.5%) (RD 1.2%, 95% CI 1.0–1.4; RR 1.47, 95% CI 1.37–1.58; OR 1.49, 95% CI 1.39–1.60; p < 0.001). Among females, 4,959 of 148,216 MS patients (3.3%) suffered stroke compared with 2,886 of 150,362 controls (1.9%) (RD 1.4%, 95% CI 1.3–1.5; RR 1.74, 95% CI 1.67–1.82; OR 1.77, 95% CI 1.69–1.85; p < 0.001).

**Vitiligo**
After 1:1 propensity score matching (matched on current age and diseases of the circulatory system), the male cohorts consisted of 27,320 patients with vitiligo and 27,320 matched controls; the female cohorts comprised 37,491 patients with vitiligo and 37,491 matched controls. After matching, the mean current age was identical between cases and controls (males: 65.9 years [SD 9.5] in both groups; females: 65.9 years [SD 9.4] in both groups).

*Parkinson’s Disease*In the male cohort, 189 of 27,161 vitiligo patients (0.7%) and 214 of 27,219 controls (0.8%) developed Parkinson’s disease, yielding a risk difference (RD) of −0.1% (95% CI −0.2 to 0.1), a risk ratio (RR) of 0.88 (95% CI 0.73–1.08), and an odds ratio (OR) of 0.88 (95% CI 0.73–1.08); p = 0.219. In the female cohort, 162 of 37,393 vitiligo patients (0.4%) versus 176 of 37,422 controls (0.5%) were diagnosed with Parkinson’s disease (RD −0.0%, 95% CI −0.1 to 0.1; RR 0.92, 95% CI 0.74–1.14; OR 0.92, 95% CI 0.74–1.14; p = 0.450).

*Alzheimer’s Disease*
Among males, Alzheimer’s disease occurred in 126 of 27,258 vitiligo patients (0.5%) compared with 124 of 27,286 controls (0.5%) (RD 0.0%, 95% CI −0.1 to 0.1; RR 1.02, 95% CI 0.79–1.30; OR 1.02, 95% CI 0.79–1.30; p = 0.893). In females, 197 of 37,415 vitiligo patients (0.5%) versus 199 of 37,431 controls (0.5%) developed Alzheimer’s disease (RD −0.0%, 95% CI −0.1 to 0.1; RR 0.99, 95% CI 0.81–1.20; OR 0.99, 95% CI 0.81–1.21; p = 0.923).

*Transient Ischemic Attack (TIA)*TIA was observed in 442 of 26,916 vitiligo males (1.6%) compared with 463 of 27,123 controls (1.7%) (RD −0.1%, 95% CI −0.3 to 0.2; RR 0.96, 95% CI 0.85–1.10; OR 0.96, 95% CI 0.84–1.10; p = 0.557). In the female cohort, 719 of 36,860 vitiligo patients (2.0%) versus 627 of 37,233 controls (1.7%) experienced TIA (RD 0.3%, 95% CI 0.1–0.5; RR 1.16, 95% CI 1.04–1.29; OR 1.16, 95% CI 1.04–1.29; p = 0.007).

*Stroke*
Stroke incidence was 700 of 26,677 vitiligo males (2.6%) versus 831 of 26,476 controls (3.1%) (RD −0.5%, 95% CI −0.8 to −0.2; RR 0.84, 95% CI 0.76–0.92; OR 0.83, 95% CI 0.75–0.92; p < 0.001). Among females, 861 of 36,790 vitiligo patients (2.3%) suffered stroke compared with 930 of 36,483 controls (2.5%) (RD −0.2%, 95% CI −0.4 to 0.0; RR 0.92, 95% CI 0.84–1.01; OR 0.92, 95% CI 0.83–1.01; p = 0.067).

**Rheumatoid Vasculitis (RV)**After 1:1 propensity score matching (matched on current age and diseases of the circulatory system), the male cohorts consisted of 2,610 patients with RV and 2,610 matched controls; the female cohorts comprised 7,881 patients with RV and 7,881 matched controls. Post-matching, current age was identical between cases and controls (males: 68.9 years [SD 9.2] in both groups; females: 67.9 years [SD 9.5] in both groups; standardized differences < 0.001).

*Parkinson’s Disease*In the male cohort, 21 of 2,584 RV patients (0.8%) and 21 of 2,595 controls (0.8%) developed Parkinson’s disease (RD 0.0%, 95% CI −0.5–0.5; RR 1.00, 95% CI 0.55–1.83; OR 1.00, 95% CI 0.55–1.85; p = 0.989). In the female cohort, 46 of 7,830 RV patients (0.6%) versus 48 of 7,849 controls (0.6%) were diagnosed with Parkinson’s disease (RD 0.0%, 95% CI −0.3–0.2; RR 0.96, 95% CI 0.64–1.44; OR 0.96, 95% CI 0.64–1.44; p = 0.845).

*Alzheimer’s Disease*
Among males, Alzheimer’s disease occurred in 30 of 2,601 RV patients (1.2%) compared with 14 of 2,601 controls (0.5%) (RD 0.6%, 95% CI 0.1–1.1; RR 2.14, 95% CI 1.14–4.03; OR 2.16, 95% CI 1.14–4.08; p = 0.015). In females, 93 of 7,848 RV patients (1.2%) versus 58 of 7,865 controls (0.7%) developed Alzheimer’s disease (RD 0.4%, 95% CI 0.1–0.8; RR 1.61, 95% CI 1.16–2.23; OR 1.61, 95% CI 1.16–2.24; p = 0.004).

*Transient Ischemic Attack (TIA)*TIA was observed in 66 of 2,521 RV males (2.6%) compared with 54 of 2,592 controls (2.1%) (RD 0.5%, 95% CI −0.3–1.4; RR 1.26, 95% CI 0.88–1.79; OR 1.26, 95% CI 0.88–1.82; p = 0.207). In the female cohort, 206 of 7,617 RV patients (2.7%) versus 181 of 7,796 controls (2.3%) experienced TIA (RD 0.4%, 95% CI −0.1–0.9; RR 1.17, 95% CI 0.96–1.42; OR 1.17, 95% CI 0.96–1.43; p = 0.129).

*Stroke*
Stroke incidence was 96 of 2,479 RV males (3.9%) versus 104 of 2,475 controls (4.2%) (RD −0.3%, 95% CI −1.4–0.8; RR 0.92, 95% CI 0.70–1.21; OR 0.92, 95% CI 0.69–1.22; p = 0.556). Among females, 321 of 7,519 RV patients (4.3%) suffered stroke compared with 258 of 7,589 controls (3.4%) (RD 0.9%, 95% CI 0.3–1.5; RR 1.26, 95% CI 1.07–1.47; OR 1.27, 95% CI 1.07–1.50; p = 0.005).

**Graves’ Disease (GD)**After 1:1 propensity score matching (matched on current age and diseases of the circulatory system), the male cohorts consisted of 144,362 patients with GD and 144,362 matched controls; the female cohorts comprised 403,640 patients with GD and 403,640 matched controls. After matching, the mean current age of male GD patients and matched controls was 67.7 years (SD 9.6) in both groups; for females, the mean current age was 66.6 years (SD 9.7) in both cohorts.

*Parkinson’s Disease*In the male cohort, 1,407 of 142,794 GD patients (1.0%) and 1,321 of 143,706 controls (0.9%) developed Parkinson’s disease, yielding a risk difference (RD) of 0.1% (95% CI 0.0–0.1), a risk ratio (RR) of 1.07 (95% CI 1.00–1.15), and an odds ratio (OR) of 1.07 (95% CI 1.00–1.16); p = 0.069. In the female cohort, 2,296 of 401,588 GD patients (0.6%) versus 1,980 of 402,732 controls (0.5%) were diagnosed with Parkinson’s disease (RD 0.1%, 95% CI 0.0–0.1; RR 1.16, 95% CI 1.10–1.23; OR 1.16, 95% CI 1.10–1.24; p < 0.001).

*Alzheimer’s Disease*Among males, Alzheimer’s disease occurred in 1,080 of 143,688 GD patients (0.8%) compared with 850 of 144,113 controls (0.6%) (RD 0.2%, 95% CI 0.1–0.2; RR 1.28, 95% CI 1.17–1.39; OR 1.28, 95% CI 1.17–1.40; p < 0.001). In females, 2,945 of 402,004 GD patients (0.7%) versus 2,518 of 402,943 controls (0.6%) developed Alzheimer’s disease (RD 0.1%, 95% CI 0.1–0.1; RR 1.17, 95% CI 1.11–1.24; OR 1.17, 95% CI 1.11–1.24; p < 0.001).

*Transient Ischemic Attack (TIA)*TIA was observed in 3,082 of 140,960 GD males (2.2%) compared with 2,819 of 143,043 controls (2.0%) (RD 0.2%, 95% CI 0.1–0.3; RR 1.11, 95% CI 1.05–1.17; OR 1.11, 95% CI 1.06–1.17; p < 0.001). In the female cohort, 8,371 of 395,879 GD patients (2.1%) versus 7,085 of 400,415 controls (1.8%) experienced TIA (RD 0.3%, 95% CI 0.3–0.4; RR 1.20, 95% CI 1.16–1.23; OR 1.20, 95% CI 1.16–1.24; p < 0.001).

*Stroke*
Stroke incidence was 5,527 of 136,911 GD males (4.0%) versus 4,921 of 138,924 controls (3.5%) (RD 0.5%, 95% CI 0.4–0.6; RR 1.15, 95% CI 1.10–1.18; OR 1.15, 95% CI 1.10–1.19; p < 0.001). Among females, 12,197 of 390,936 GD patients (3.1%) suffered stroke compared with 10,678 of 392,300 controls (2.7%) (RD 0.4%, 95% CI 0.3–0.5; RR 1.15, 95% CI 1.12–1.18; OR 1.15, 95% CI 1.12–1.18; p < 0.001).

**Vasculitis**

After 1:1 propensity score matching (matched on current age and diseases of the circulatory system), the male cohorts consisted of 23,513 patients with vasculitis and 23,513 matched controls; the female cohorts comprised 51,358 patients with vasculitis and 51,358 matched controls. After matching, the mean current age was identical between cases and controls (males: 68.7 years [SD 9.6] in both groups; females: 68.1 years [SD 9.6] in both groups).

*Parkinson’s Disease*
In the male cohort, 244 of 23,274 vasculitis patients (1.0%) and 251 of 23,366 controls (1.1%) developed Parkinson’s disease, yielding a risk difference (RD) of −0.0% (95% CI −0.2–0.2), a risk ratio (RR) of 0.98 (95% CI 0.82–1.16), and an odds ratio (OR) of 0.98 (95% CI 0.82–1.16); p = 0.785. In the female cohort, 395 of 51,091 vasculitis patients (0.8%) versus 311 of 51,183 controls (0.6%) were diagnosed with Parkinson’s disease (RD 0.2%, 95% CI 0.1–0.3; RR 1.27, 95% CI 1.10–1.47; OR 1.27, 95% CI 1.10–1.48; p = 0.001).

*Alzheimer’s Disease*Among males, Alzheimer’s disease occurred in 152 of 23,433 vasculitis patients (0.6%) compared with 178 of 23,452 controls (0.8%) (RD −0.1%, 95% CI −0.3–0.0; RR 0.85, 95% CI 0.69–1.06; OR 0.85, 95% CI 0.69–1.06; p = 0.153). In females, 535 of 51,171 vasculitis patients (1.0%) versus 402 of 51,238 controls (0.8%) developed Alzheimer’s disease (RD 0.3%, 95% CI 0.1–0.4; RR 1.33, 95% CI 1.17–1.52; OR 1.34, 95% CI 1.17–1.52; p < 0.001).

*Transient Ischemic Attack (TIA)*
TIA was observed in 576 of 22,920 vasculitis males (2.5%) compared with 536 of 23,257 controls (2.3%) (RD 0.2%, 95% CI −0.1–0.5; RR 1.09, 95% CI 0.97–1.23; OR 1.09, 95% CI 0.97–1.23; p = 0.144). In the female cohort, 1,436 of 49,935 vasculitis patients (2.9%) versus 1,155 of 50,839 controls (2.3%) experienced TIA (RD 0.6%, 95% CI 0.4–0.8; RR 1.27, 95% CI 1.17–1.37; OR 1.27, 95% CI 1.18–1.38; p < 0.001).

*Stroke*
Stroke incidence was 947 of 22,464 vasculitis males (4.2%) versus 933 of 22,508 controls (4.1%) (RD 0.1%, 95% CI −0.3–0.4; RR 1.02, 95% CI 0.93–1.11; OR 1.02, 95% CI 0.93–1.12; p = 0.709). Among females, 2,063 of 49,583 vasculitis patients (4.2%) suffered stroke compared with 1,729 of 49,397 controls (3.5%) (RD 0.7%, 95% CI 0.4–0.9; RR 1.19, 95% CI 1.12–1.27; OR 1.20, 95% CI 1.12–1.28; p < 0.001).

**Dermatomyositis**
After 1:1 propensity score matching (matched on current age and diseases of the circulatory system), the male cohorts consisted of 13,341 patients with dermatomyositis and 13,341 matched controls; the female cohorts comprised 27,377 patients with dermatomyositis and 27,377 matched controls. After matching, the mean current age was identical between cases and controls (males: 68.3 years [SD 9.5] in both groups; females: 67.3 years [SD 9.5] in both groups).

*Parkinson’s Disease*
In the male cohort, 118 of 13,245 dermatomyositis patients (0.9%) and 133 of 13,287 controls (1.0%) developed Parkinson’s disease, yielding a risk difference (RD) of −0.1% (95% CI −0.3 to 0.1), a risk ratio (RR) of 0.89 (95% CI 0.70–1.14), and an odds ratio (OR) of 0.89 (95% CI 0.69–1.14); p = 0.354. In the female cohort, 144 of 27,240 dermatomyositis patients (0.5%) versus 145 of 27,318 controls (0.5%) were diagnosed with Parkinson’s disease (RD −0.0%, 95% CI −0.1 to 0.1; RR 1.00, 95% CI 0.79–1.25; OR 1.00, 95% CI 0.79–1.25; p = 0.972).

*Alzheimer’s Disease*Among males, Alzheimer’s disease occurred in 71 of 13,307 dermatomyositis patients (0.5%) compared with 79 of 13,322 controls (0.6%) (RD −0.1%, 95% CI −0.2 to 0.1; RR 0.90, 95% CI 0.65–1.24; OR 0.90, 95% CI 0.65–1.24; p = 0.517). In females, 179 of 27,322 dermatomyositis patients (0.7%) versus 197 of 27,321 controls (0.7%) developed Alzheimer’s disease (RD −0.1%, 95% CI −0.2 to 0.1; RR 0.91, 95% CI 0.74–1.11; OR 0.91, 95% CI 0.74–1.11; p = 0.351).

*Transient Ischemic Attack (TIA)*
TIA was observed in 295 of 13,088 dermatomyositis males (2.3%) compared with 251 of 13,233 controls (1.9%) (RD 0.4%, 95% CI 0.0–0.7; RR 1.19, 95% CI 1.01–1.40; OR 1.19, 95% CI 1.01–1.41; p = 0.042). In the female cohort, 603 of 26,866 dermatomyositis patients (2.2%) versus 446 of 27,159 controls (1.6%) experienced TIA (RD 0.6%, 95% CI 0.4–0.8; RR 1.37, 95% CI 1.21–1.54; OR 1.38, 95% CI 1.22–1.56; p < 0.001).

*Stroke*
Stroke incidence was 578 of 12,678 dermatomyositis males (4.6%) versus 436 of 12,871 controls (3.4%) (RD 1.2%, 95% CI 0.7–1.7; RR 1.35, 95% CI 1.19–1.52; OR 1.36, 95% CI 1.20–1.55; p < 0.001). Among females, 967 of 26,524 dermatomyositis patients (3.6%) suffered stroke compared with 695 of 26,638 controls (2.6%) (RD 1.0%, 95% CI 0.7–1.3; RR 1.40, 95% CI 1.27–1.54; OR 1.41, 95% CI 1.28–1.56; p < 0.001).

**Arthropathic Psoriasis**
After 1:1 propensity score matching (matched on current age and diseases of the circulatory system), the male cohorts consisted of 62,348 patients with arthropathic psoriasis and 62,348 matched controls; the female cohorts comprised 87,495 patients with arthropathic psoriasis and 87,495 matched controls. After matching, the mean current age of male arthropathic psoriasis patients and matched controls was 65.8 years (SD 9.1) in both groups; for females, the mean current age was 65.3 years (SD 9.1) in both cohorts.

*Parkinson’s Disease*
In the male cohort, 499 of 62,020 arthropathic psoriasis patients (0.8 %) and 450 of 62,124 controls (0.7 %) developed Parkinson’s disease, yielding a risk difference of 0.1 % (95 % CI 0.0–0.2), a risk ratio of 1.11 (95 % CI 0.98–1.26), and an odds ratio of 1.11 (95 % CI 0.98–1.26); p = 0.105. In the female cohort, 403 of 87,206 arthropathic psoriasis patients (0.5 %) versus 353 of 87,324 controls (0.4 %) were diagnosed with Parkinson’s disease (RD 0.1 %, 95 % CI 0.0–0.1; RR 1.14, 95 % CI 0.99–1.32; OR 1.14, 95 % CI 0.99–1.32; p = 0.066).

*Alzheimer’s Disease*
Among males, Alzheimer’s disease occurred in 262 of 62,266 arthropathic psoriasis patients (0.4 %) compared with 282 of 62,272 controls (0.5 %) (RD −0.0 %, 95 % CI −0.1–0.0; RR 0.93, 95 % CI 0.79–1.10; OR 0.93, 95 % CI 0.78–1.10; p = 0.391). In females, 397 of 87,365 arthropathic psoriasis patients (0.5 %) versus 428 of 87,364 controls (0.5 %) developed Alzheimer’s disease (RD −0.0 %, 95 % CI −0.1–0.0; RR 0.93, 95 % CI 0.81–1.06; OR 0.93, 95 % CI 0.81–1.06; p = 0.279).

*Transient Ischemic Attack (TIA)*TIA was observed in 1,110 of 61,443 arthropathic psoriasis males (1.8 %) compared with 982 of 61,933 controls (1.6 %) (RD 0.2 %, 95 % CI 0.1–0.4; RR 1.14, 95 % CI 1.05–1.25; OR 1.14, 95 % CI 1.05–1.25; p = 0.003). In the female cohort, 1,632 of 86,082 arthropathic psoriasis patients (1.9 %) versus 1,438 of 86,880 controls (1.7 %) experienced TIA (RD 0.2 %, 95 % CI 0.1–0.4; RR 1.15, 95 % CI 1.07–1.23; OR 1.15, 95 % CI 1.07–1.23; p < 0.001).

*Stroke*
Stroke incidence was 1,674 of 60,989 arthropathic psoriasis males (2.7 %) versus 1,812 of 60,500 controls (3.0 %) (RD −0.3 %, 95 % CI −0.4–−0.1; RR 0.92, 95 % CI 0.85–0.98; OR 0.91, 95 % CI 0.85–0.98; p = 0.009). Among females, 2,193 of 85,755 arthropathic psoriasis patients (2.6 %) suffered stroke compared with 2,206 of 85,188 controls (2.6 %) (RD −0.0 %, 95 % CI −0.2–0.1; RR 0.99, 95 % CI 0.93–1.05; OR 0.99, 95 % CI 0.93–1.05; p = 0.673).

**Addison’s Disease**
After 1:1 propensity score matching (matched on current age and diseases of the circulatory system), the male cohorts consisted of 19,453 patients with Addison’s disease and 19,453 matched controls; the female cohorts comprised 29,303 patients with Addison’s disease and 29,303 matched controls. After matching, current age was identical between cases and controls (males: 68.8 years [SD 9.3] in both groups; females: 67.0 years [SD 9.6] in both groups).

*Parkinson’s Disease*In the male cohort, 236 of 19,166 Addison’s disease patients (1.2%) and 209 of 19,358 controls (1.1%) developed Parkinson’s disease, yielding a risk difference (RD) of 0.2% (95% CI −0.1–0.4), a risk ratio (RR) of 1.14 (95% CI 0.95–1.37), and an odds ratio (OR) of 1.14 (95% CI 0.95–1.38); p = 0.164. In the female cohort, 244 of 29,058 Addison’s disease patients (0.8%) versus 134 of 29,231 controls (0.5%) were diagnosed with Parkinson’s disease (RD 0.4%, 95% CI 0.3–0.5; RR 1.83, 95% CI 1.48–2.26; OR 1.84, 95% CI 1.49–2.27; p < 0.001).

*Alzheimer’s Disease*Among males, Alzheimer’s disease occurred in 114 of 19,349 Addison’s disease patients (0.6%) compared with 131 of 19,423 controls (0.7%) (RD −0.1%, 95% CI −0.2–0.1; RR 0.87, 95% CI 0.68–1.12; OR 0.87, 95% CI 0.68–1.12; p = 0.289). In females, 253 of 29,192 Addison’s disease patients (0.9%) versus 189 of 29,252 controls (0.6%) developed Alzheimer’s disease (RD 0.2%, 95% CI 0.1–0.4; RR 1.34, 95% CI 1.11–1.62; OR 1.34, 95% CI 1.11–1.62; p = 0.002).

*Transient Ischemic Attack (TIA)*TIA was observed in 448 of 18,873 Addison’s disease males (2.4%) compared with 447 of 19,268 controls (2.3%) (RD 0.1%, 95% CI −0.3–0.4; RR 1.02, 95% CI 0.90–1.16; OR 1.02, 95% CI 0.90–1.17; p = 0.728). In the female cohort, 761 of 28,439 Addison’s disease patients (2.7%) versus 591 of 29,016 controls (2.0%) experienced TIA (RD 0.6%, 95% CI 0.4–0.9; RR 1.31, 95% CI 1.18–1.46; OR 1.32, 95% CI 1.19–1.48; p < 0.001).

*Stroke*
Stroke incidence was 919 of 18,179 Addison’s disease males (5.1%) versus 720 of 18,618 controls (3.9%) (RD 1.2%, 95% CI 0.8–1.6; RR 1.31, 95% CI 1.19–1.44; OR 1.32, 95% CI 1.20–1.46; p < 0.001). Among females, 1,304 of 27,834 Addison’s disease patients (4.7%) suffered stroke compared with 870 of 28,311 controls (3.1%) (RD 1.6%, 95% CI 1.3–1.9; RR 1.52, 95% CI 1.40–1.66; OR 1.55, 95% CI 1.42–1.69; p < 0.001).

**Psoriasis**
After 1:1 propensity score matching (matched on current age and diseases of the circulatory system), the male cohorts consisted of 267,325 patients with psoriasis and 267,325 matched controls; the female cohorts comprised 319,298 patients with psoriasis and 319,298 matched controls. After matching, the mean current age was identical between cases and controls (males: 66.4 years [SD 9.4] in both groups; females: 66.4 years [SD 9.4] in both cohorts).

*Parkinson’s Disease*In the male cohort, 2,044 of 265,756 psoriasis patients (0.8%) and 1,991 of 266,412 controls (0.7%) developed Parkinson’s disease, yielding a risk difference (RD) of 0.0% (95% CI −0.0–0.1), a risk ratio (RR) of 1.03 (95% CI 0.97–1.09), and an odds ratio (OR) of 1.03 (95% CI 0.97–1.10); p = 0.360. In the female cohort, 1,529 of 318,210 psoriasis patients (0.5%) versus 1,504 of 318,572 controls (0.5%) were diagnosed with Parkinson’s disease (RD 0.0%, 95% CI −0.0–0.0; RR 1.02, 95% CI 0.95–1.09; OR 1.02, 95% CI 0.95–1.09; p = 0.627).

*Alzheimer’s Disease*
Among males, Alzheimer’s disease occurred in 1,381 of 266,766 psoriasis patients (0.5%) compared with 1,252 of 267,024 controls (0.5%) (RD 0.0%, 95% CI 0.0–0.1; RR 1.10, 95% CI 1.02–1.19; OR 1.10, 95% CI 1.02–1.19; p = 0.011). In females, 1,966 of 318,586 psoriasis patients (0.6%) versus 1,856 of 318,830 controls (0.6%) developed Alzheimer’s disease (RD 0.0%, 95% CI −0.0–0.1; RR 1.06, 95% CI 1.00–1.13; OR 1.06, 95% CI 1.00–1.13; p = 0.071).

*Transient Ischemic Attack (TIA)*TIA was observed in 4,901 of 263,415 psoriasis males (1.9%) compared with 4,373 of 265,375 controls (1.6%) (RD 0.2%, 95% CI 0.1–0.3; RR 1.13, 95% CI 1.08–1.18; OR 1.13, 95% CI 1.09–1.18; p < 0.001). In the female cohort, 6,066 of 314,241 psoriasis patients (1.9%) versus 5,481 of 316,898 controls (1.7%) experienced TIA (RD 0.2%, 95% CI 0.1–0.3; RR 1.12, 95% CI 1.08–1.16; OR 1.12, 95% CI 1.08–1.16; p < 0.001).

*Stroke*
Stroke incidence was 7,983 of 260,666 psoriasis males (3.1%) versus 7,820 of 259,158 controls (3.0%) (RD 0.0%, 95% CI −0.0–0.1; RR 1.02, 95% CI 0.98–1.05; OR 1.02, 95% CI 0.98–1.05; p = 0.344). Among females, 8,438 of 312,660 psoriasis patients (2.7%) suffered stroke compared with 8,369 of 310,755 controls (2.7%) (RD 0.0%, 95% CI −0.1–0.1; RR 1.00, 95% CI 0.97–1.03; OR 1.00, 95% CI 0.97–1.03; p = 0.890).

**Sjögren Syndrome**
After 1:1 propensity score matching (matched on current age and diseases of the circulatory system), the male cohorts consisted of 36,703 patients with Sjögren syndrome and 36,703 matched controls; the female cohorts comprised 224,884 patients with Sjögren syndrome and 224,884 matched controls. After matching, the mean current age of male Sjögren patients and matched controls was 68.9 years (SD 9.4) in both groups; for females, the mean current age was 67.2 years (SD 9.4) in both cohorts.

*Parkinson’s Disease*
In the male cohort, 498 of 36,152 Sjögren patients (1.4%) and 363 of 36,542 controls (1.0%) developed Parkinson’s disease, yielding a risk difference (RD) of 0.4% (95% CI 0.2–0.5), a risk ratio (RR) of 1.39 (95% CI 1.21–1.59), and an odds ratio (OR) of 1.39 (95% CI 1.22–1.59); p < 0.001. In the female cohort, 1,579 of 223,623 Sjögren patients (0.7%) versus 1,102 of 224,373 controls (0.5%) were diagnosed with Parkinson’s disease (RD 0.2%, 95% CI 0.2–0.3; RR 1.44, 95% CI 1.33–1.55; OR 1.44, 95% CI 1.33–1.56; p < 0.001).

*Alzheimer’s Disease*Among males, Alzheimer’s disease occurred in 232 of 36,579 Sjögren patients (0.6%) compared with 220 of 36,633 controls (0.6%) (RD 0.0%, 95% CI −0.1–0.1; RR 1.06, 95% CI 0.88–1.27; OR 1.06, 95% CI 0.88–1.27; p = 0.561). In females, 1,504 of 224,427 Sjögren patients (0.7%) versus 1,473 of 224,499 controls (0.7%) developed Alzheimer’s disease (RD 0.0%, 95% CI 0.0–0.1; RR 1.02, 95% CI 0.95–1.10; OR 1.02, 95% CI 0.95–1.10; p = 0.563).

*Transient Ischemic Attack (TIA)*TIA was observed in 906 of 35,586 Sjögren males (2.5%) compared with 783 of 36,349 controls (2.2%) (RD 0.4%, 95% CI 0.2–0.6; RR 1.18, 95% CI 1.08–1.30; OR 1.19, 95% CI 1.08–1.31; p = 0.001). In the female cohort, 5,683 of 219,684 Sjögren patients (2.6%) versus 3,930 of 223,123 controls (1.8%) experienced TIA (RD 0.8%, 95% CI 0.7–0.9; RR 1.47, 95% CI 1.41–1.53; OR 1.48, 95% CI 1.42–1.54; p < 0.001).

*Stroke*
Stroke incidence was 1,246 of 35,072 Sjögren males (3.6%) versus 1,279 of 35,288 controls (3.6%) (RD −0.1%, 95% CI −0.3–0.2; RR 0.98, 95% CI 0.90–1.06; OR 0.98, 95% CI 0.90–1.06; p = 0.609). Among females, 6,296 of 219,371 Sjögren patients (2.9%) suffered stroke compared with 5,901 of 218,590 controls (2.7%) (RD 0.2%, 95% CI 0.1–0.3; RR 1.06, 95% CI 1.03–1.10; OR 1.06, 95% CI 1.03–1.10; p = 0.001).

**Scleroderma**

After 1:1 propensity score matching (matched on current age and diseases of the circulatory system), the male cohorts consisted of 11,220 patients with scleroderma and 11,220 matched controls; the female cohorts comprised 50,666 patients with scleroderma and 50,666 matched controls. After matching, the mean current age was identical between cases and controls (males: 68.2 years [SD 9.1] in both groups; females: 67.8 years [SD 9.4] in both groups).

*Parkinson’s Disease*
In the male cohort, 84 of 11,119 scleroderma patients (0.8%) and 99 of 11,168 controls (0.9%) developed Parkinson’s disease, yielding a risk difference (RD) of −0.1% (95% CI −0.4 to 0.1), a risk ratio (RR) of 0.85 (95% CI 0.64–1.14), and an odds ratio (OR) of 0.85 (95% CI 0.64–1.14); p = 0.279. In the female cohort, 225 of 50,454 scleroderma patients (0.4%) versus 261 of 50,532 controls (0.5%) were diagnosed with Parkinson’s disease (RD −0.1%, 95% CI −0.2 to 0.0; RR 0.86, 95% CI 0.72–1.03; OR 0.86, 95% CI 0.72–1.03; p = 0.105).

*Alzheimer’s Disease*
Among males, Alzheimer’s disease occurred in 63 of 11,186 scleroderma patients (0.6%) compared with 61 of 11,202 controls (0.5%) (RD 0.0%, 95% CI −0.2 to 0.2; RR 1.03, 95% CI 0.73–1.47; OR 1.03, 95% CI 0.73–1.47; p = 0.851). In females, 249 of 50,584 scleroderma patients (0.5%) versus 347 of 50,586 controls (0.7%) developed Alzheimer’s disease (RD −0.2%, 95% CI −0.3 to −0.1; RR 0.72, 95% CI 0.61–0.84; OR 0.72, 95% CI 0.61–0.84; p < 0.001).

*Transient Ischemic Attack (TIA)*TIA was observed in 181 of 11,006 scleroderma males (1.6%) compared with 219 of 11,146 controls (2.0%) (RD −0.3%, 95% CI −0.7 to 0.0; RR 0.84, 95% CI 0.69–1.02; OR 0.83, 95% CI 0.68–1.02; p = 0.073). In the female cohort, 1,021 of 49,902 scleroderma patients (2.0%) versus 933 of 50,258 controls (1.9%) experienced TIA (RD 0.2%, 95% CI 0.0 to 0.4; RR 1.10, 95% CI 1.01–1.20; OR 1.10, 95% CI 1.01–1.21; p = 0.030).

*Stroke*
Stroke incidence was 382 of 10,749 scleroderma males (3.6%) versus 410 of 10,834 controls (3.8%) (RD −0.2%, 95% CI −0.7 to 0.3; RR 0.94, 95% CI 0.82–1.08; OR 0.94, 95% CI 0.81–1.08; p = 0.368). Among females, 1,529 of 49,343 scleroderma patients (3.1%) suffered stroke compared with 1,374 of 49,249 controls (2.8%) (RD 0.3%, 95% CI 0.1–0.5; RR 1.11, 95% CI 1.03–1.19; OR 1.11, 95% CI 1.04–1.20; p = 0.004).

**Myositis**
After 1:1 propensity score matching (matched on current age and diseases of the circulatory system), the male cohorts consisted of 197,674 patients with myositis and 197,674 matched controls; the female cohorts comprised 424,804 patients with myositis and 424,804 matched controls. After matching, the mean current age of male myositis patients and matched controls was 66.4 years (SD 9.5) in both groups; for females, the mean current age was 66.4 years (SD 9.4) in both cohorts.

*Parkinson’s Disease*In the male cohort, 2,207 of 196,113 myositis patients (1.1%) and 1,597 of 196,862 controls (0.8%) developed Parkinson’s disease, yielding a risk difference (RD) of 0.3% (95% CI 0.3–0.4), a risk ratio (RR) of 1.39 (95% CI 1.30–1.48), and an odds ratio (OR) of 1.39 (95% CI 1.30–1.48); p < 0.001. In the female cohort, 3,616 of 423,074 myositis patients (0.9%) versus 2,085 of 423,896 controls (0.5%) were diagnosed with Parkinson’s disease (RD 0.4%, 95% CI 0.3–0.4; RR 1.74, 95% CI 1.65–1.83; OR 1.75, 95% CI 1.65–1.84; p < 0.001).

*Alzheimer’s Disease*
Among males, Alzheimer’s disease occurred in 1,212 of 197,310 myositis patients (0.6%) compared with 1,011 of 197,387 controls (0.5%) (RD 0.1%, 95% CI 0.1–0.1; RR 1.20, 95% CI 1.10–1.30; OR 1.20, 95% CI 1.10–1.31; p < 0.001). In females, 3,272 of 424,199 myositis patients (0.8%) versus 2,519 of 424,131 controls (0.6%) developed Alzheimer’s disease (RD 0.2%, 95% CI 0.1–0.2; RR 1.30, 95% CI 1.23–1.37; OR 1.30, 95% CI 1.23–1.37; p < 0.001).

*Transient Ischemic Attack (TIA)*TIA was observed in 5,912 of 192,793 myositis males (3.1%) compared with 3,562 of 195,924 controls (1.8%) (RD 1.2%, 95% CI 1.2–1.3; RR 1.69, 95% CI 1.62–1.76; OR 1.71, 95% CI 1.64–1.78; p < 0.001). In the female cohort, 13,775 of 416,151 myositis patients (3.3%) versus 7,252 of 421,576 controls (1.7%) experienced TIA (RD 1.6%, 95% CI 1.5–1.7; RR 1.92, 95% CI 1.87–1.98; OR 1.96, 95% CI 1.90–2.01; p < 0.001).

*Stroke*
Stroke incidence was 8,744 of 190,663 myositis males (4.6%) versus 6,295 of 190,476 controls (3.3%) (RD 1.3%, 95% CI 1.2–1.4; RR 1.39, 95% CI 1.34–1.43; OR 1.41, 95% CI 1.36–1.45; p < 0.001). Among females, 17,339 of 415,443 myositis patients (4.2%) suffered stroke compared with 10,829 of 413,244 controls (2.6%) (RD 1.6%, 95% CI 1.5–1.6; RR 1.59, 95% CI 1.56–1.63; OR 1.62, 95% CI 1.58–1.66; p < 0.001).

**Celiac Disease**
After 1:1 propensity score matching (matched on current age and diseases of the circulatory system), the male cohorts consisted of 31,565 patients with celiac disease and 31,565 matched controls; the female cohorts comprised 75,600 patients with celiac disease and 75,600 matched controls. After matching, the mean current age of male celiac disease patients and matched controls was 66.5 years (SD 9.7) in both groups; for females, the mean current age was 65.1 years (SD 9.6) in both cohorts.

*Parkinson’s Disease*In the male cohort, 301 of 31,296 celiac disease patients (1.0%) and 243 of 31,454 controls (0.8%) developed Parkinson’s disease, yielding a risk difference of 0.2% (95% CI 0.0–0.3), a risk ratio of 1.25 (95% CI 1.05–1.47), and an odds ratio of 1.25 (95% CI 1.05–1.48); p = 0.011. In the female cohort, 416 of 75,315 celiac disease patients (0.6%) versus 288 of 75,448 controls (0.4%) were diagnosed with Parkinson’s disease (RD 0.2%, 95% CI 0.1–0.2; RR 1.45, 95% CI 1.25–1.68; OR 1.45, 95% CI 1.25–1.69; p < 0.001).

*Alzheimer’s Disease*Among males, Alzheimer’s disease occurred in 219 of 31,469 celiac disease patients (0.7%) compared with 150 of 31,530 controls (0.5%) (RD 0.2%, 95% CI 0.1–0.3; RR 1.46, 95% CI 1.19–1.80; OR 1.47, 95% CI 1.19–1.81; p < 0.001). In females, 504 of 75,423 celiac disease patients (0.7%) versus 373 of 75,496 controls (0.5%) developed Alzheimer’s disease (RD 0.2%, 95% CI 0.1–0.3; RR 1.35, 95% CI 1.18–1.55; OR 1.36, 95% CI 1.18–1.55; p < 0.001).

*Transient Ischemic Attack (TIA)*TIA was observed in 617 of 31,017 celiac disease males (2.0%) compared with 490 of 31,338 controls (1.6%) (RD 0.4%, 95% CI 0.2–0.6; RR 1.27, 95% CI 1.13–1.43; OR 1.28, 95% CI 1.13–1.44; p < 0.001). In the female cohort, 1,447 of 74,466 celiac disease patients (1.9%) versus 1,130 of 75,123 controls (1.5%) experienced TIA (RD 0.4%, 95% CI 0.3–0.6; RR 1.29, 95% CI 1.20–1.39; OR 1.30, 95% CI 1.20–1.40; p < 0.001).

*Stroke*
Stroke incidence was 856 of 30,847 celiac disease males (2.8%) versus 938 of 30,608 controls (3.1%) (RD −0.3%, 95% CI −0.6–0.0; RR 0.91, 95% CI 0.83–0.99; OR 0.90, 95% CI 0.82–0.99; p = 0.033). Among females, 1,786 of 74,310 celiac disease patients (2.4%) suffered stroke compared with 1,678 of 73,972 controls (2.3%) (RD 0.1%, 95% CI 0.0–0.3; RR 1.06, 95% CI 0.99–1.13; OR 1.06, 95% CI 0.99–1.14; p = 0.085).

**Rheumatoid Arthritis**
After 1:1 propensity score matching (matched on current age and diseases of the circulatory system), the male cohorts consisted of 168,380 patients with RA and 168,380 matched controls; the female cohorts comprised 471,796 patients with RA and 471,796 matched controls. After matching, the mean current age of male RA patients and matched controls was 69.7 years (SD 9.1) in both groups; for females, the mean current age was 68.4 years (SD 9.5) in both cohorts.

*Parkinson’s Disease*
In the male cohort, 1,598 of 166,748 RA patients (1.0%) and 1,611 of 167,596 controls (1.0%) developed Parkinson’s disease, yielding a risk difference (RD) of −0.0% (95% CI −0.1 to 0.1), a risk ratio (RR) of 1.00 (95% CI 0.93–1.07), and an odds ratio (OR) of 1.00 (95% CI 0.93–1.07); p = 0.931. In the female cohort, 2,796 of 469,285 RA patients (0.6%) versus 2,459 of 470,630 controls (0.5%) were diagnosed with Parkinson’s disease (RD 0.1%, 95% CI 0.0–0.1; RR 1.14, 95% CI 1.08–1.20; OR 1.14, 95% CI 1.08–1.21; p < 0.001).

*Alzheimer’s Disease*Among males, Alzheimer’s disease occurred in 1,329 of 167,706 RA patients (0.8%) compared with 1,064 of 168,100 controls (0.6%) (RD 0.2%, 95% CI 0.1–0.2; RR 1.25, 95% CI 1.16–1.36; OR 1.25, 95% CI 1.16–1.36; p < 0.001). In females, 3,831 of 470,129 RA patients (0.8%) versus 3,338 of 470,905 controls (0.7%) developed Alzheimer’s disease (RD 0.1%, 95% CI 0.1–0.1; RR 1.15, 95% CI 1.10–1.20; OR 1.15, 95% CI 1.10–1.21; p < 0.001).

*Transient Ischemic Attack (TIA)*
TIA was observed in 3,684 of 164,919 RA males (2.2%) compared with 3,281 of 166,965 controls (2.0%) (RD 0.3%, 95% CI 0.2–0.4; RR 1.14, 95% CI 1.08–1.19; OR 1.14, 95% CI 1.09–1.20; p < 0.001). In the female cohort, 11,125 of 462,776 RA patients (2.4%) versus 8,463 of 468,233 controls (1.8%) experienced TIA (RD 0.6%, 95% CI 0.5–0.7; RR 1.33, 95% CI 1.29–1.37; OR 1.34, 95% CI 1.30–1.38; p < 0.001).

*Stroke*
Stroke incidence was 6,952 of 161,576 RA males (4.3%) versus 5,710 of 162,612 controls (3.5%) (RD 0.8%, 95% CI 0.7–0.9; RR 1.22, 95% CI 1.18–1.27; OR 1.24, 95% CI 1.19–1.28; p < 0.001). Among females, 17,230 of 457,114 RA patients (3.8%) suffered stroke compared with 12,851 of 459,024 controls (2.8%) (RD 1.0%, 95% CI 0.9–1.0; RR 1.35, 95% CI 1.32–1.38; OR 1.36, 95% CI 1.33–1.39; p < 0.001).

**Type 1 Diabetes Mellitus (T1DM)**After 1:1 propensity score matching (matched on current age and diseases of the circulatory system), the male cohorts consisted of 297,119 patients with T1DM and 297,119 matched controls; the female cohorts comprised 267,077 patients with T1DM and 267,077 matched controls. After matching, the mean current age of male T1DM patients and matched controls was 68.0 years (SD 9.5) in both groups; for females, the mean current age was 68.3 years (SD 9.6) in both cohorts.

*Parkinson’s Disease*In the male cohort, 3,170 of 295,018 T1DM patients (1.1%) and 2,742 of 295,750 controls (0.9%) developed Parkinson’s disease, yielding a risk difference (RD) of 0.1% (95% CI 0.1–0.2), a risk ratio (RR) of 1.16 (95% CI 1.10–1.22), and an odds ratio (OR) of 1.16 (95% CI 1.10–1.22); p < 0.001. In the female cohort, 2,221 of 265,797 T1DM patients (0.8%) versus 1,507 of 266,323 controls (0.6%) were diagnosed with Parkinson’s disease (RD 0.3%, 95% CI 0.2–0.3; RR 1.48, 95% CI 1.38–1.58; OR 1.48, 95% CI 1.39–1.58; p < 0.001).

*Alzheimer’s Disease*
Among males, Alzheimer’s disease occurred in 2,360 of 296,207 T1DM patients (0.8%) compared with 1,762 of 296,630 controls (0.6%) (RD 0.2%, 95% CI 0.2–0.2; RR 1.34, 95% CI 1.26–1.43; OR 1.34, 95% CI 1.26–1.43; p < 0.001). In females, 2,905 of 266,011 T1DM patients (1.1%) versus 2,035 of 266,462 controls (0.8%) developed Alzheimer’s disease (RD 0.3%, 95% CI 0.3–0.4; RR 1.43, 95% CI 1.35–1.51; OR 1.44, 95% CI 1.36–1.52; p < 0.001).

*Transient Ischemic Attack (TIA)*TIA was observed in 8,778 of 290,563 T1DM males (3.0%) compared with 5,733 of 294,338 controls (1.9%) (RD 1.1%, 95% CI 1.0–1.2; RR 1.55, 95% CI 1.50–1.60; OR 1.57, 95% CI 1.52–1.62; p < 0.001). In the female cohort, 9,388 of 260,454 T1DM patients (3.6%) versus 5,563 of 264,463 controls (2.1%) experienced TIA (RD 1.5%, 95% CI 1.4–1.6; RR 1.71, 95% CI 1.66–1.77; OR 1.74, 95% CI 1.68–1.80; p < 0.001).

*Stroke*
Stroke incidence was 18,680 of 281,118 T1DM males (6.6%) versus 9,782 of 285,573 controls (3.4%) (RD 3.2%, 95% CI 3.1–3.3; RR 1.94, 95% CI 1.89–1.99; OR 2.01, 95% CI 1.96–2.06; p < 0.001). Among females, 17,749 of 253,355 T1DM patients (7.0%) suffered stroke compared with 8,067 of 257,575 controls (3.1%) (RD 3.9%, 95% CI 3.8–4.0; RR 2.24, 95% CI 2.18–2.29; OR 2.33, 95% CI 2.27–2.39; p < 0.001).

**Autoimmune Thyroiditis**

After 1:1 propensity score matching, the male cohorts consisted of 48,787 patients with autoimmune thyroiditis and 48,787 matched controls; the female cohorts comprised 274,166 patients with autoimmune thyroiditis and 274,166 matched controls. After matching, the mean current age was identical between cases and controls (males: 66.4 years [SD 9.5] in both groups; females: 64.7 years [SD 9.3] in both groups).

*Parkinson’s Disease*
In the male cohort, 465 of 48,350 autoimmune thyroiditis patients (1.0%) and 407 of 48,599 controls (0.8%) developed Parkinson’s disease, yielding a risk difference (RD) of 0.1% (95% CI 0.0–0.2), a risk ratio (RR) of 1.15 (95% CI 1.01–1.31), and an odds ratio (OR) of 1.15 (95% CI 1.01–1.31); p = 0.040. In the female cohort, 1,242 of 273,185 autoimmune thyroiditis patients (0.5%) versus 993 of 273,645 controls (0.4%) were diagnosed with Parkinson’s disease (RD 0.1%, 95% CI 0.1–0.1; RR 1.25, 95% CI 1.15–1.36; OR 1.25, 95% CI 1.15–1.36; p < 0.001).

*Alzheimer’s Disease*
Among males, Alzheimer’s disease occurred in 310 of 48,592 autoimmune thyroiditis patients (0.6%) compared with 258 of 48,726 controls (0.5%) (RD 0.1%, 95% CI 0.0–0.2; RR 1.20, 95% CI 1.02–1.42; OR 1.21, 95% CI 1.02–1.42; p = 0.026). In females, 1,385 of 273,525 autoimmune thyroiditis patients (0.5%) versus 947 of 273,872 controls (0.3%) developed Alzheimer’s disease (RD 0.2%, 95% CI 0.1–0.2; RR 1.46, 95% CI 1.35–1.59; OR 1.47, 95% CI 1.35–1.59; p < 0.001).

*Transient Ischemic Attack (TIA)*TIA was observed in 987 of 47,651 autoimmune thyroiditis males (2.1%) compared with 941 of 48,314 controls (1.9%) (RD 0.1%, 95% CI −0.1–0.3; RR 1.06, 95% CI 0.97–1.16; OR 1.06, 95% CI 0.97–1.17; p = 0.172). In the female cohort, 4,858 of 269,611 autoimmune thyroiditis patients (1.8%) versus 2,738 of 273,042 controls (1.0%) experienced TIA (RD 0.8%, 95% CI 0.7–0.9; RR 1.80, 95% CI 1.72–1.88; OR 1.81, 95% CI 1.73–1.90; p < 0.001).

*Stroke*
Stroke incidence was 1,359 of 47,025 autoimmune thyroiditis males (2.9%) versus 1,563 of 46,904 controls (3.3%) (RD −0.4%, 95% CI −0.7–−0.2; RR 0.87, 95% CI 0.81–0.93; OR 0.86, 95% CI 0.80–0.93; p < 0.001). Among females, 5,513 of 268,841 autoimmune thyroiditis patients (2.1%) suffered stroke compared with 4,593 of 271,786 controls (1.7%) (RD 0.4%, 95% CI 0.3–0.4; RR 1.21, 95% CI 1.17–1.26; OR 1.22, 95% CI 1.17–1.27; p < 0.001).

**Lupus Erythematosus**
After 1:1 propensity score matching (matched on current age and diseases of the circulatory system), the male cohorts consisted of 10,767 patients with lupus erythematosus and 10,767 matched controls; the female cohorts comprised 51,063 patients with lupus erythematosus and 51,063 matched controls. After matching, the mean current age of male lupus patients and matched controls was 66.6 years (SD 9.3) in both groups; for females, the mean current age was 65.4 years (SD 9.3) in both cohorts.

*Parkinson’s Disease*In the male cohort, 91 of 10,706 lupus patients (0.8%) and 78 of 10,722 controls (0.7%) developed Parkinson’s disease, yielding a risk difference (RD) of 0.1% (95% CI −0.1–0.4), a risk ratio (RR) of 1.17 (95% CI 0.86–1.58), and an odds ratio (OR) of 1.17 (95% CI 0.86–1.58); p = 0.311. In the female cohort, 240 of 50,874 lupus patients (0.5%) versus 233 of 50,939 controls (0.5%) were diagnosed with Parkinson’s disease (RD 0.0%, 95% CI −0.1–0.1; RR 1.03, 95% CI 0.86–1.23; OR 1.03, 95% CI 0.86–1.24; p = 0.736).

*Alzheimer’s Disease*Among males, Alzheimer’s disease occurred in 66 of 10,742 lupus patients (0.6%) compared with 48 of 10,757 controls (0.4%) (RD 0.2%, 95% CI 0.0–0.4; RR 1.38, 95% CI 0.95–2.00; OR 1.38, 95% CI 0.95–2.00; p = 0.090). In females, 292 of 50,961 lupus patients (0.6%) versus 286 of 50,996 controls (0.6%) developed Alzheimer’s disease (RD 0.0%, 95% CI −0.1–0.1; RR 1.02, 95% CI 0.87–1.20; OR 1.02, 95% CI 0.87–1.20; p = 0.796).

*Transient Ischemic Attack (TIA)*TIA was observed in 256 of 10,520 lupus males (2.4%) compared with 178 of 10,678 controls (1.7%) (RD 0.8%, 95% CI 0.4–1.1; RR 1.46, 95% CI 1.21–1.76; OR 1.47, 95% CI 1.21–1.79; p < 0.001). In the female cohort, 1,513 of 49,806 lupus patients (3.0%) versus 910 of 50,635 controls (1.8%) experienced TIA (RD 1.2%, 95% CI 1.1–1.4; RR 1.69, 95% CI 1.56–1.83; OR 1.71, 95% CI 1.57–1.86; p < 0.001).

*Stroke*
Stroke incidence was 460 of 10,323 lupus males (4.5%) versus 311 of 10,396 controls (3.0%) (RD 1.5%, 95% CI 0.9–2.0; RR 1.49, 95% CI 1.29–1.72; OR 1.51, 95% CI 1.31–1.75; p < 0.001). Among females, 2,329 of 49,193 lupus patients (4.7%) suffered stroke compared with 1,373 of 49,514 controls (2.8%) (RD 2.0%, 95% CI 1.7–2.2; RR 1.71, 95% CI 1.60–1.82; OR 1.74, 95% CI 1.63–1.87; p < 0.001).

**Ulcerative Colitis**
After 1:1 propensity score matching (matched on current age and diseases of the circulatory system), the male cohorts consisted of 128,039 patients with UC and 128,039 matched controls; the female cohorts comprised 147,297 patients with UC and 147,297 matched controls. After matching, the mean current age was identical between cases and controls (males: 67.0 years [SD 9.6] in both groups; females: 67.0 years [SD 9.6] in both groups).

*Parkinson’s Disease*
In the male cohort, 999 of 126,927 UC patients (0.8%) and 1,018 of 127,554 controls (0.8%) developed Parkinson’s disease (RD −0.0%, 95% CI −0.1 to 0.1; RR 0.99, 95% CI 0.90–1.08; OR 0.99, 95% CI 0.90–1.08; p = 0.754). In the female cohort, 710 of 146,506 UC patients (0.5%) versus 699 of 146,950 controls (0.5%) were diagnosed with Parkinson’s disease (RD 0.0%, 95% CI −0.0 to 0.1; RR 1.02, 95% CI 0.92–1.13; OR 1.02, 95% CI 0.92–1.13; p = 0.726).

*Alzheimer’s Disease*Among males, Alzheimer’s disease occurred in 681 of 127,651 UC patients (0.5%) compared with 644 of 127,875 controls (0.5%) (RD 0.0%, 95% CI −0.0 to 0.1; RR 1.06, 95% CI 0.95–1.18; OR 1.06, 95% CI 0.95–1.18; p = 0.293). In females, 1,004 of 146,761 UC patients (0.7%) versus 983 of 147,033 controls (0.7%) developed Alzheimer’s disease (RD 0.0%, 95% CI −0.0 to 0.1; RR 1.02, 95% CI 0.94–1.12; OR 1.02, 95% CI 0.94–1.12; p = 0.607).

*Transient Ischemic Attack (TIA)*TIA was observed in 2,118 of 125,809 UC males (1.7%) compared with 2,327 of 127,047 controls (1.8%) (RD −0.1%, 95% CI −0.3 to −0.0; RR 0.92, 95% CI 0.87–0.97; OR 0.92, 95% CI 0.87–0.97; p = 0.005). In the female cohort, 2,901 of 144,241 UC patients (2.0%) versus 2,679 of 146,142 controls (1.8%) experienced TIA (RD 0.2%, 95% CI 0.1–0.3; RR 1.10, 95% CI 1.04–1.16; OR 1.10, 95% CI 1.04–1.16; p < 0.001).

*Stroke*
Stroke incidence was 3,519 of 123,987 UC males (2.8%) versus 3,963 of 124,063 controls (3.2%) (RD −0.4%, 95% CI −0.5 to −0.2; RR 0.89, 95% CI 0.85–0.93; OR 0.88, 95% CI 0.85–0.93; p < 0.001). Among females, 3,993 of 142,925 UC patients (2.8%) suffered stroke compared with 3,945 of 143,059 controls (2.8%) (RD 0.0%, 95% CI −0.1 to 0.2; RR 1.01, 95% CI 0.97–1.06; OR 1.01, 95% CI 0.97–1.06; p = 0.556).

**Crohn’s Disease**After 1:1 propensity score matching (matched on current age and diseases of the circulatory system), the male cohorts consisted of 86,758 patients with Crohn’s disease and 86,758 matched controls; the female cohorts comprised 116,125 patients with Crohn’s disease and 116,125 matched controls. After matching, the mean current age of male Crohn’s disease patients and matched controls was 65.8 years (SD 9.7) in both groups; for females, the mean current age was 65.6 years (SD 9.6) in both cohorts.

*Parkinson’s Disease*In the male cohort, 678 of 86,173 Crohn’s disease patients (0.8%) and 574 of 86,484 controls (0.7%) developed Parkinson’s disease, yielding a risk difference (RD) of 0.1% (95% CI 0.0–0.2), a risk ratio (RR) of 1.19 (95% CI 1.06–1.32), and an odds ratio (OR) of 1.19 (95% CI 1.06–1.33); p = 0.003. In the female cohort, 577 of 115,698 Crohn’s disease patients (0.5%) versus 487 of 115,923 controls (0.4%) were diagnosed with Parkinson’s disease (RD 0.1%, 95% CI 0.0–0.1; RR 1.19, 95% CI 1.05–1.34; OR 1.19, 95% CI 1.05–1.34; p = 0.005).

*Alzheimer’s Disease*Among males, Alzheimer’s disease occurred in 393 of 86,576 Crohn’s disease patients (0.5%) compared with 354 of 86,674 controls (0.4%) (RD 0.0%, 95% CI 0.0–0.1; RR 1.11, 95% CI 0.96–1.28; OR 1.11, 95% CI 0.96–1.28; p = 0.148). In females, 665 of 115,867 Crohn’s disease patients (0.6%) versus 641 of 115,982 controls (0.6%) developed Alzheimer’s disease (RD 0.0%, 95% CI −0.0–0.1; RR 1.04, 95% CI 0.93–1.16; OR 1.04, 95% CI 0.93–1.16; p = 0.494).

*Transient Ischemic Attack (TIA)*TIA was observed in 1,601 of 85,749 Crohn’s disease males (1.9%) compared with 1,225 of 86,271 controls (1.4%) (RD 0.4%, 95% CI 0.3–0.6; RR 1.31, 95% CI 1.22–1.42; OR 1.32, 95% CI 1.23–1.42; p < 0.001). In the female cohort, 2,297 of 114,572 Crohn’s disease patients (2.0%) versus 1,612 of 115,472 controls (1.4%) experienced TIA (RD 0.6%, 95% CI 0.5–0.7; RR 1.44, 95% CI 1.35–1.53; OR 1.45, 95% CI 1.36–1.54; p < 0.001).

*Stroke*
Stroke incidence was 2,597 of 84,920 Crohn’s disease males (3.1%) versus 2,306 of 84,664 controls (2.7%) (RD 0.3%, 95% CI 0.2–0.5; RR 1.12, 95% CI 1.06–1.19; OR 1.13, 95% CI 1.06–1.19; p < 0.001). Among females, 3,195 of 113,776 Crohn’s disease patients (2.8%) suffered stroke compared with 2,647 of 113,737 controls (2.3%) (RD 0.5%, 95% CI 0.4–0.6; RR 1.21, 95% CI 1.15–1.27; OR 1.21, 95% CI 1.15–1.28; p < 0.001).

**EXPERIMENT 1C**

**Chronic Inflammatory Demyelinating Polyneuropathy (CIPD)**

After 1:1 propensity score matching (matched on *current age* and *immune suppressants*), the male cohorts consisted of 15 164 CIPD patients and 15 164 matched controls; the female cohorts comprised 10 523 CIPD patients and 10 523 matched controls. After matching, the mean current age in males was 70.0 years (SD 9.0) in both CIPD and control cohorts; in females, the mean current age was 67.8 years (SD 9.5) in both cohorts.

*Parkinson’s Disease*

In the male cohort, 232 of 14 880 CIPD patients (1.6%) and 125 of 15 122 controls (0.8%) developed Parkinson’s disease, yielding a risk difference (RD) of 0.7% (95% CI 0.5–1.0), a risk ratio (RR) of 1.89 (95% CI 1.52–2.34), and an odds ratio (OR) of 1.90 (95% CI 1.53–2.36); p < 0.001. In the female cohort, 102 of 10 376 CIPD patients (1.0%) versus 41 of 10 499 controls (0.4%) were diagnosed with Parkinson’s disease (RD 0.6%, 95% CI 0.4–0.8; RR 2.52, 95% CI 1.76–3.61; OR 2.53, 95% CI 1.77–3.65; p < 0.001).

*Alzheimer’s Disease*

Among males, Alzheimer’s disease occurred in 122 of 15 103 CIPD patients (0.8%) compared with 78 of 15 151 controls (0.5%) (RD 0.3%, 95% CI 0.1–0.5; RR 1.57, 95% CI 1.18–2.08; OR 1.58, 95% CI 1.19–2.10; p = 0.002). In females, 70 of 10 493 CIPD patients (0.7%) versus 55 of 10 519 controls (0.5%) developed Alzheimer’s disease (RD 0.1%, 95% CI −0.1–0.4; RR 1.28, 95% CI 0.90–1.81; OR 1.28, 95% CI 0.90–1.82; p = 0.174).

*Transient Ischemic Attack (TIA)*

TIA was observed in 373 of 14 746 CIPD males (2.5%) compared with 180 of 15 134 controls (1.2%) (RD 1.3%, 95% CI 1.0–1.6; RR 2.13, 95% CI 1.78–2.54; OR 2.16, 95% CI 1.80–2.57; p < 0.001). In the female cohort, 265 of 10 215 CIPD patients (2.6%) versus 112 of 10 494 controls (1.1%) experienced TIA (RD 1.5%, 95% CI 1.2–1.9; RR 2.43, 95% CI 1.95–3.04; OR 2.47, 95% CI 1.97–3.10; p < 0.001).

*Stroke*

Stroke incidence was 599 of 14 401 CIPD males (4.2%) versus 346 of 15 037 controls (2.3%) (RD 1.9%, 95% CI 1.5–2.3; RR 1.84, 95% CI 1.63–2.09; OR 1.88, 95% CI 1.65–2.14; p < 0.001). Among females, 443 of 10 027 CIPD patients (4.4%) suffered stroke compared with 185 of 10 466 controls (1.8%) (RD 2.7%, 95% CI 2.2–3.1; RR 2.51, 95% CI 2.12–2.96; OR 2.57, 95% CI 2.17–3.04; p < 0.001).

**Guillain–Barré Syndrome (GBS)**

After 1:1 propensity score matching (matched on *current age* and *immune suppressants*), the male cohorts consisted of 15 957 GBS patients and 15 957 matched controls; the female cohorts comprised 13 331 GBS patients and 13 331 matched controls. After matching, the mean current age in males was 68.3 years (SD 9.5) in both GBS and control cohorts; in females, the mean current age was 67.3 years (SD 9.6) in both cohorts.

*Parkinson’s Disease*

In the male cohort, 151 of 15 752 GBS patients (1.0%) and 113 of 15 913 controls (0.7%) developed Parkinson’s disease, yielding a risk difference (RD) of 0.2% (95% CI 0.0–0.4), a risk ratio (RR) of 1.35 (95% CI 1.06–1.72), and an odds ratio (OR) of 1.35 (95% CI 1.06–1.73); p = 0.015. In the female cohort, 102 of 13 203 GBS patients (0.8%) versus 47 of 13 312 controls (0.4%) were diagnosed with Parkinson’s disease (RD 0.4%, 95% CI 0.2–0.6; RR 2.19, 95% CI 1.55–3.09; OR 2.20, 95% CI 1.56–3.11; p < 0.001).

*Alzheimer’s Disease*

Among males, Alzheimer’s disease occurred in 74 of 15 925 GBS patients (0.5%) compared with 71 of 15 939 controls (0.4%) (RD 0.0%, 95% CI −0.1–0.2; RR 1.04, 95% CI 0.75–1.44; OR 1.04, 95% CI 0.75–1.45; p = 0.799). In females, 74 of 13 291 GBS patients (0.6%) versus 56 of 13 321 controls (0.4%) developed Alzheimer’s disease (RD 0.1%, 95% CI 0.0–0.3; RR 1.33, 95% CI 0.94–1.87; OR 1.33, 95% CI 0.94–1.88; p = 0.111).

*Transient Ischemic Attack (TIA)*

TIA was observed in 304 of 15 563 GBS males (2.0%) compared with 174 of 15 912 controls (1.1%) (RD 0.9%, 95% CI 0.6–1.1; RR 1.79, 95% CI 1.48–2.15; OR 1.80, 95% CI 1.50–2.17; p < 0.001). In the female cohort, 302 of 13 010 GBS patients (2.3%) versus 140 of 13 303 controls (1.1%) experienced TIA (RD 1.3%, 95% CI 1.0–1.6; RR 2.21, 95% CI 1.81–2.69; OR 2.23, 95% CI 1.83–2.73; p < 0.001).

*Stroke*

Stroke incidence was 623 of 15 004 GBS males (4.2%) versus 364 of 15 828 controls (2.3%) (RD 1.9%, 95% CI 1.5–2.2; RR 1.81, 95% CI 1.59–2.05; OR 1.84, 95% CI 1.61–2.10; p < 0.001). Among females, 514 of 12 623 GBS patients (4.1%) suffered stroke compared with 219 of 13 272 controls (1.7%) (RD 2.4%, 95% CI 2.0–2.8; RR 2.47, 95% CI 2.11–2.88; OR 2.53, 95% CI 2.16–2.97; p < 0.001).

**Myasthenia Gravis (MG)**

After 1:1 propensity score matching (matched on *current age* and *immune suppressants*), the male cohorts consisted of 30 752 MG patients and 30 752 matched controls; the female cohorts comprised 31 455 MG patients and 31 455 matched controls. The cohorts were well balanced on current age and immune suppressants after matching (standardized differences < 0.01). After matching, the mean current age of male MG patients and matched controls was 71.3 years (SD 9.1) in both groups; for females, the mean current age was 68.6 years (SD 9.8) in both MG and control cohorts.

*Parkinson’s Disease*

In the male cohort, 522 of 30 107 MG patients (1.7%) and 276 of 30 647 controls (0.9%) developed Parkinson’s disease, yielding a risk difference of 0.8% (95% CI 0.7–1.0), a risk ratio of 1.93 (95% CI 1.67–2.23), and an odds ratio of 1.94 (95% CI 1.68–2.25); p < 0.001. In the female cohort, 347 of 31 081 MG patients (1.1%) versus 120 of 31 414 controls (0.4%) were diagnosed with Parkinson’s disease (RD 0.7%, 95% CI 0.6–0.9; RR 2.92, 95% CI 2.38–3.60; OR 2.94, 95% CI 2.39–3.62; p < 0.001).

*Alzheimer’s Disease*

Among males, Alzheimer’s disease occurred in 230 of 30 630 MG patients (0.8%) compared with 176 of 30 714 controls (0.6%) (RD 0.2%, 95% CI 0.0–0.3; RR 1.31, 95% CI 1.08–1.59; OR 1.31, 95% CI 1.08–1.60; p = 0.007). In females, 224 of 31 341 MG patients (0.7%) versus 173 of 31 421 controls (0.6%) developed Alzheimer’s disease (RD 0.2%, 95% CI 0.0–0.3; RR 1.30, 95% CI 1.06–1.58; OR 1.30, 95% CI 1.07–1.59; p = 0.010).

*Transient Ischemic Attack (TIA)*

TIA was observed in 699 of 29 772 MG males (2.3%) compared with 385 of 30 662 controls (1.3%) (RD 1.1%, 95% CI 0.9–1.3; RR 1.87, 95% CI 1.65–2.11; OR 1.89, 95% CI 1.67–2.14; p < 0.001). In the female cohort, 744 of 30 411 MG patients (2.4%) versus 339 of 31 369 controls (1.1%) experienced TIA (RD 1.4%, 95% CI 1.2–1.6; RR 2.26, 95% CI 1.99–2.57; OR 2.29, 95% CI 2.02–2.61; p < 0.001).

*Stroke*

Stroke incidence was 1 137 of 29 019 MG males (3.9%) versus 807 of 30 481 controls (2.6%) (RD 1.3%, 95% CI 1.0–1.6; RR 1.48, 95% CI 1.36–1.62; OR 1.50, 95% CI 1.37–1.64; p < 0.001). Among females, 1 044 of 29 947 MG patients (3.5%) suffered stroke compared with 576 of 31 269 controls (1.8%) (RD 1.6%, 95% CI 1.4–1.9; RR 1.89, 95% CI 1.71–2.09; OR 1.92, 95% CI 1.74–2.13; p < 0.001).

**Multiple Sclerosis (MS)**

After 1:1 propensity score matching (matched on *current age* and *immune suppressants*), the male cohorts consisted of 54 379 MS patients and 54 379 matched controls; the female cohorts comprised 150 267 MS patients and 150 267 matched controls. The cohorts were well balanced on current age and immune suppressants after matching (standardized differences < 0.01). After matching, the mean current age of male MS patients and matched controls was 64.7 years (SD 9.1) in both groups; for females, the mean current age was 64.4 years (SD 9.0) in both MS and control cohorts.

*Parkinson’s Disease*

In the male cohort, 544 of 53 688 MS patients (1.0%) and 250 of 54 294 controls (0.5%) developed Parkinson’s disease, yielding a risk difference of 0.6% (95% CI 0.5–0.7), a risk ratio of 2.20 (95% CI 1.89–2.56), and an odds ratio of 2.21 (95% CI 1.90–2.57); p < 0.001. In the female cohort, 964 of 149 466 MS patients (0.6%) versus 428 of 150 107 controls (0.3%) were diagnosed with Parkinson’s disease (RD 0.4%, 95% CI 0.3–0.4; RR 2.26, 95% CI 2.02–2.53; OR 2.27, 95% CI 2.02–2.54; p < 0.001).

*Alzheimer’s Disease*

Among males, Alzheimer’s disease occurred in 258 of 54 218 MS patients (0.5%) compared with 146 of 54 362 controls (0.3%) (RD 0.2%, 95% CI 0.1–0.3; RR 1.77, 95% CI 1.45–2.17; OR 1.78, 95% CI 1.45–2.17; p < 0.001). In females, 735 of 149 977 MS patients (0.5%) versus 418 of 150 186 controls (0.3%) developed Alzheimer’s disease (RD 0.2%, 95% CI 0.2–0.3; RR 1.76, 95% CI 1.56–1.98; OR 1.76, 95% CI 1.56–1.99; p < 0.001).

*Transient Ischemic Attack (TIA)*

TIA was observed in 916 of 53 488 MS males (1.7%) compared with 486 of 54 270 controls (0.9%) (RD 0.8%, 95% CI 0.7–1.0; RR 1.91, 95% CI 1.72–2.13; OR 1.93, 95% CI 1.73–2.16; p < 0.001). In the female cohort, 2 785 of 148 026 MS patients (1.9%) versus 1 343 of 150 017 controls (0.9%) experienced TIA (RD 1.0%, 95% CI 0.9–1.1; RR 2.10, 95% CI 1.97–2.24; OR 2.12, 95% CI 1.99–2.27; p < 0.001).

*Stroke*

Stroke incidence was 1 894 of 52 200 MS males (3.6%) versus 1 066 of 54 001 controls (2.0%) (RD 1.7%, 95% CI 1.5–1.9; RR 1.84, 95% CI 1.71–1.98; OR 1.87, 95% CI 1.73–2.02; p < 0.001). Among females, 4 863 of 145 950 MS patients (3.3%) suffered stroke compared with 2 089 of 149 625 controls (1.4%) (RD 1.9%, 95% CI 1.8–2.0; RR 2.39, 95% CI 2.27–2.51; OR 2.43, 95% CI 2.31–2.56; p < 0.001).

**Vitiligo**

After 1:1 propensity score matching (matched on *current age* and *immune suppressants*), the male cohorts consisted of 27 318 vitiligo patients and 27 318 matched controls; the female cohorts comprised 37 489 vitiligo patients and 37 489 matched controls. After matching, the mean current age in males was 65.9 years (SD 9.5) in both vitiligo and control cohorts; in females, the mean current age was 65.9 years (SD 9.4) in both cohorts.

*Parkinson’s Disease*

In the male cohort, 189 of 27 159 vitiligo patients (0.7%) and 163 of 27 253 controls (0.6%) developed Parkinson’s disease, yielding a risk difference (RD) of 0.1% (95% CI 0.0–0.2), a risk ratio (RR) of 1.16 (95% CI 0.94–1.43), and an odds ratio (OR) of 1.16 (95% CI 0.94–1.44); p = 0.155. In the female cohort, 162 of 37 391 vitiligo patients (0.4%) versus 137 of 37 441 controls (0.4%) were diagnosed with Parkinson’s disease (RD 0.1%, 95% CI 0.0–0.2; RR 1.18, 95% CI 0.94–1.49; OR 1.18, 95% CI 0.94–1.49; p = 0.144).

*Alzheimer’s Disease*

Among males, Alzheimer’s disease occurred in 126 of 27 256 vitiligo patients (0.5%) compared with 75 of 27 304 controls (0.3%) (RD 0.2%, 95% CI 0.1–0.3; RR 1.68, 95% CI 1.27–2.24; OR 1.69, 95% CI 1.27–2.25; p < 0.001). In females, 197 of 37 413 vitiligo patients (0.5%) versus 148 of 37 466 controls (0.4%) developed Alzheimer’s disease (RD 0.1%, 95% CI 0.0–0.2; RR 1.33, 95% CI 1.08–1.65; OR 1.34, 95% CI 1.08–1.65; p = 0.008).

*Transient Ischemic Attack (TIA)*

TIA was observed in 442 of 26 914 vitiligo males (1.6%) compared with 268 of 27 252 controls (1.0%) (RD 0.7%, 95% CI 0.5–0.9; RR 1.67, 95% CI 1.44–1.94; OR 1.68, 95% CI 1.44–1.96; p < 0.001). In the female cohort, 719 of 36 858 vitiligo patients (2.0%) versus 370 of 37 416 controls (1.0%) experienced TIA (RD 1.0%, 95% CI 0.8–1.1; RR 1.97, 95% CI 1.74–2.23; OR 1.99, 95% CI 1.75–2.26; p < 0.001).

*Stroke*

Stroke incidence was 700 of 26 675 vitiligo males (2.6%) versus 517 of 27 106 controls (1.9%) (RD 0.7%, 95% CI 0.5–1.0; RR 1.38, 95% CI 1.23–1.54; OR 1.39, 95% CI 1.24–1.56; p < 0.001). Among females, 861 of 36 788 vitiligo patients (2.3%) suffered stroke compared with 572 of 37 291 controls (1.5%) (RD 0.8%, 95% CI 0.6–1.0; RR 1.53, 95% CI 1.37–1.69; OR 1.54, 95% CI 1.38–1.71; p < 0.001).

**Rheumatoid Vasculitis**

After 1:1 propensity score matching, the female cohorts consisted of 7,881 patients with rheumatoid vasculitis and 7,881 matched controls. The cohorts were balanced on the matching variables after PSM (e.g., IMMUNE SUPPRESSANTS: 788/7,881 [10.0%] in both cohorts; p = 1; standardized difference < 0.001). For males, the matched cohorts comprised 2,610 rheumatoid vasculitis patients and 2,610 matched controls; after matching, mean current age was 68.9 years (SD 9.2) in both cohorts, and IMMUNE SUPPRESSANTS were 216/2,610 (8.3%) in both cohorts (p = 1; standardized difference = 0).

*Parkinson’s Disease*

In the male cohort, 21 of 2,584 rheumatoid vasculitis patients (0.8%) versus 25 of 2,604 controls (1.0%) developed Parkinson’s disease (RD −0.1%, 95% CI −0.7–0.4; RR 0.85, 95% CI 0.48–1.51; OR 0.85, 95% CI 0.47–1.51; p = 0.571). In the female cohort, 46 of 7,830 rheumatoid vasculitis patients (0.6%) versus 27 of 7,869 controls (0.3%) developed Parkinson’s disease (RD 0.2%, 95% CI 0.0–0.5; RR 1.71, 95% CI 1.07–2.75; OR 1.72, 95% CI 1.07–2.76; p = 0.024).

*Alzheimer’s Disease*

In the female cohort, 93 of 7,848 rheumatoid vasculitis patients (1.2%) versus 31 of 7,874 controls (0.4%) developed Alzheimer’s disease (RD 0.8%, 95% CI 0.5–1.1; RR 3.01, 95% CI 2.01–4.50; OR 3.03, 95% CI 2.02–4.57; p < 0.001). For males, Alzheimer’s disease could not be reported here because there were insufficient data for that outcome.

*Transient Ischemic Attack (TIA)*

In the male cohort, 66 of 2,521 rheumatoid vasculitis patients (2.6%) versus 30 of 2,602 controls (1.2%) experienced TIA (RD 1.5%, 95% CI 0.7–2.2; RR 2.27, 95% CI 1.48–3.48; OR 2.30, 95% CI 1.49–3.56; p < 0.001). In the female cohort, 206 of 7,617 rheumatoid vasculitis patients (2.7%) versus 81 of 7,848 controls (1.0%) experienced TIA (RD 1.7%, 95% CI 1.2–2.1; RR 2.62, 95% CI 2.03–3.38; OR 2.67, 95% CI 2.06–3.45; p < 0.001).

*Stroke*

In the male cohort, 96 of 2,479 rheumatoid vasculitis patients (3.9%) versus 58 of 2,575 controls (2.3%) had stroke (RD 1.6%, 95% CI 0.7–2.6; RR 1.72, 95% CI 1.25–2.37; OR 1.75, 95% CI 1.26–2.43; p < 0.001). In the female cohort, 321 of 7,519 rheumatoid vasculitis patients (4.3%) versus 131 of 7,824 controls (1.7%) had stroke (RD 2.6%, 95% CI 2.1–3.1; RR 2.55, 95% CI 2.09–3.12; OR 2.62, 95% CI 2.13–3.22; p < 0.001).

**Graves’ Disease**

After 1:1 propensity score matching (matched on *current age* and *immune suppressants*), the male cohorts consisted of 144 366 Graves’ disease patients and 144 366 matched controls; the female cohorts comprised 403 642 Graves’ disease patients and 403 642 matched controls. After matching, the mean current age in males was 67.7 years (SD 9.6) in both cohorts (immune suppressant use 2.4% in both); in females, the mean current age was 66.6 years (SD 9.7) in both cohorts (immune suppressant use 2.5% in both).

*Parkinson’s Disease*

In the male cohort, 1 407 of 142 798 Graves’ disease patients (1.0%) and 921 of 143 985 controls (0.6%) developed Parkinson’s disease, yielding a risk difference (RD) of 0.3% (95% CI 0.3–0.4), a risk ratio (RR) of 1.54 (95% CI 1.42–1.67), and an odds ratio (OR) of 1.55 (95% CI 1.42–1.68); p < 0.001. In the female cohort, 2 295 of 401 590 Graves’ disease patients (0.6%) versus 1 400 of 403 101 controls (0.3%) were diagnosed with Parkinson’s disease (RD 0.2%, 95% CI 0.2–0.3; RR 1.64, 95% CI 1.54–1.76; OR 1.65, 95% CI 1.54–1.76; p < 0.001).

*Alzheimer’s Disease*

Among males, Alzheimer’s disease occurred in 1 080 of 143 692 Graves’ disease patients (0.8%) compared with 537 of 144 279 controls (0.4%) (RD 0.4%, 95% CI 0.3–0.4; RR 2.02, 95% CI 1.82–2.24; OR 2.03, 95% CI 1.83–2.25; p < 0.001). In females, 2 946 of 402 006 Graves’ disease patients (0.7%) versus 1 688 of 403 369 controls (0.4%) developed Alzheimer’s disease (RD 0.3%, 95% CI 0.3–0.3; RR 1.75, 95% CI 1.65–1.86; OR 1.76, 95% CI 1.66–1.87; p < 0.001).

*Transient Ischemic Attack (TIA)*

TIA was observed in 3 083 of 140 963 Graves’ disease males (2.2%) compared with 1 544 of 143 993 controls (1.1%) (RD 1.1%, 95% CI 1.0–1.2; RR 2.04, 95% CI 1.92–2.17; OR 2.06, 95% CI 1.94–2.19; p < 0.001). In the female cohort, 8 372 of 395 881 Graves’ disease patients (2.1%) versus 3 941 of 402 824 controls (1.0%) experienced TIA (RD 1.1%, 95% CI 1.1–1.2; RR 2.16, 95% CI 2.08–2.24; OR 2.19, 95% CI 2.11–2.27; p < 0.001).

*Stroke*

Stroke incidence was 5 528 of 136 914 Graves’ disease males (4.0%) versus 3 193 of 143 122 controls (2.2%) (RD 1.8%, 95% CI 1.7–1.9; RR 1.81, 95% CI 1.73–1.89; OR 1.85, 95% CI 1.76–1.93; p < 0.001). Among females, 12 196 of 390 937 Graves’ disease patients (3.1%) suffered stroke compared with 6 520 of 401 640 controls (1.6%) (RD 1.5%, 95% CI 1.4–1.6; RR 1.92, 95% CI 1.87–1.98; OR 1.95, 95% CI 1.89–2.01; p < 0.001).

**Vasculitis**

After 1:1 propensity score matching (matched on *current age* and *immune suppressants*), the male cohorts consisted of 23 510 vasculitis patients and 23 510 matched controls; the female cohorts comprised 51 354 vasculitis patients and 51 354 matched controls. The cohorts were well balanced on current age and immune suppressants after matching. After matching, the mean current age in males was 68.7 years (SD 9.6) in both cohorts (immune suppressant use 6.0% in both); in females, the mean current age was 68.1 years (SD 9.6) in both cohorts (immune suppressant use 7.5% in both).

*Parkinson’s Disease*

In the male cohort, 244 of 23 271 vasculitis patients (1.0%) and 174 of 23 450 controls (0.7%) developed Parkinson’s disease, yielding a risk difference (RD) of 0.3% (95% CI 0.1–0.5), a risk ratio (RR) of 1.41 (95% CI 1.16–1.71), and an odds ratio (OR) of 1.42 (95% CI 1.17–1.72); p < 0.001. In the female cohort, 394 of 51 087 vasculitis patients (0.8%) versus 193 of 51 278 controls (0.4%) were diagnosed with Parkinson’s disease (RD 0.4%, 95% CI 0.3–0.5; RR 2.05, 95% CI 1.73–2.43; OR 2.06, 95% CI 1.73–2.45; p < 0.001).

*Alzheimer’s Disease*

Among males, Alzheimer’s disease occurred in 152 of 23 430 vasculitis patients (0.6%) compared with 98 of 23 498 controls (0.4%) (RD 0.2%, 95% CI 0.1–0.4; RR 1.56, 95% CI 1.21–2.00; OR 1.56, 95% CI 1.21–2.01; p = 0.001). In females, 535 of 51 167 vasculitis patients (1.0%) versus 286 of 51 304 controls (0.6%) developed Alzheimer’s disease (RD 0.5%, 95% CI 0.4–0.6; RR 1.88, 95% CI 1.63–2.16; OR 1.88, 95% CI 1.63–2.18; p < 0.001).

*Transient Ischemic Attack (TIA)*

TIA was observed in 576 of 22 917 vasculitis males (2.5%) compared with 294 of 23 443 controls (1.3%) (RD 1.3%, 95% CI 1.0–1.5; RR 2.00, 95% CI 1.74–2.30; OR 2.03, 95% CI 1.76–2.34; p < 0.001). In the female cohort, 1 435 of 49 931 vasculitis patients (2.9%) versus 567 of 51 221 controls (1.1%) experienced TIA (RD 1.8%, 95% CI 1.6–1.9; RR 2.60, 95% CI 2.36–2.86; OR 2.65, 95% CI 2.40–2.92; p < 0.001).

*Stroke*

Stroke incidence was 946 of 22 461 vasculitis males (4.2%) versus 545 of 23 332 controls (2.3%) (RD 1.9%, 95% CI 1.5–2.2; RR 1.80, 95% CI 1.63–2.00; OR 1.84, 95% CI 1.65–2.04; p < 0.001). Among females, 2 063 of 49 580 vasculitis patients (4.2%) suffered stroke compared with 903 of 51 072 controls (1.8%) (RD 2.4%, 95% CI 2.2–2.6; RR 2.35, 95% CI 2.18–2.54; OR 2.41, 95% CI 2.23–2.61; p < 0.001).

**Dermatomyositis**

After 1:1 propensity score matching (matched on *current age* and *immune suppressants*), the male cohorts consisted of 13 344 dermatomyositis patients and 13 344 matched controls; the female cohorts comprised 27 375 dermatomyositis patients and 27 375 matched controls. After matching, the mean current age in males was 68.3 years (SD 9.5) in both cohorts (immune suppressant use 8.7% in both); in females, the mean current age was 67.3 years (SD 9.5) in both cohorts (immune suppressant use 11.0% in both).

*Parkinson’s Disease*

In the male cohort, 118 of 13 248 dermatomyositis patients (0.9%) and 84 of 13 299 controls (0.6%) developed Parkinson’s disease, yielding a risk difference (RD) of 0.3% (95% CI 0.0–0.5), a risk ratio (RR) of 1.41 (95% CI 1.07–1.86), and an odds ratio (OR) of 1.41 (95% CI 1.07–1.87); p = 0.015. In the female cohort, 144 of 27 238 dermatomyositis patients (0.5%) versus 101 of 27 327 controls (0.4%) were diagnosed with Parkinson’s disease (RD 0.2%, 95% CI 0.0–0.3; RR 1.43, 95% CI 1.11–1.85; OR 1.43, 95% CI 1.11–1.85; p = 0.005).

*Alzheimer’s Disease*

Among males, Alzheimer’s disease occurred in 71 of 13 310 dermatomyositis patients (0.5%) compared with 60 of 13 333 controls (0.5%) (RD 0.1%, 95% CI −0.1–0.3; RR 1.18, 95% CI 0.84–1.67; OR 1.19, 95% CI 0.84–1.68; p = 0.330). In females, 179 of 27 320 dermatomyositis patients (0.7%) versus 114 of 27 353 controls (0.4%) developed Alzheimer’s disease (RD 0.2%, 95% CI 0.1–0.4; RR 1.57, 95% CI 1.24–1.99; OR 1.57, 95% CI 1.25–2.00; p < 0.001).

*Transient Ischemic Attack (TIA)*

TIA was observed in 295 of 13 091 dermatomyositis males (2.3%) compared with 147 of 13 301 controls (1.1%) (RD 1.1%, 95% CI 0.8–1.5; RR 2.04, 95% CI 1.68–2.48; OR 2.06, 95% CI 1.69–2.52; p < 0.001). In the female cohort, 603 of 26 864 dermatomyositis patients (2.2%) versus 236 of 27 301 controls (0.9%) experienced TIA (RD 1.4%, 95% CI 1.2–1.6; RR 2.60, 95% CI 2.24–3.01; OR 2.63, 95% CI 2.26–3.07; p < 0.001).

*Stroke*

Stroke incidence was 578 of 12 681 dermatomyositis males (4.6%) versus 294 of 13 223 controls (2.2%) (RD 2.3%, 95% CI 1.9–2.8; RR 2.05, 95% CI 1.79–2.35; OR 2.10, 95% CI 1.82–2.42; p < 0.001). Among females, 967 of 26 522 dermatomyositis patients (3.6%) suffered stroke compared with 485 of 27 241 controls (1.8%) (RD 1.9%, 95% CI 1.6–2.1; RR 2.05, 95% CI 1.84–2.28; OR 2.09, 95% CI 1.87–2.33; p < 0.001).

**Arthropathic Psoriasis**

After 1:1 propensity score matching (matched on *current age* and *immune suppressants*), the male cohorts consisted of 62 341 arthropathic psoriasis patients and 62 341 matched controls; the female cohorts comprised 87 489 arthropathic psoriasis patients and 87 489 matched controls. After matching, the mean current age in males was 65.8 years (SD 9.1) in both cohorts (immune suppressant use 6.8% in both); in females, the mean current age was 65.3 years (SD 9.1) in both cohorts (immune suppressant use 8.7% in both).

*Parkinson’s Disease*

In the male cohort, 499 of 62 013 arthropathic psoriasis patients (0.8%) and 348 of 62 199 controls (0.6%) developed Parkinson’s disease, yielding a risk difference (RD) of 0.2% (95% CI 0.2–0.3), a risk ratio (RR) of 1.44 (95% CI 1.25–1.65), and an odds ratio (OR) of 1.44 (95% CI 1.26–1.65); p < 0.001. In the female cohort, 403 of 87 200 arthropathic psoriasis patients (0.5%) versus 288 of 87 384 controls (0.3%) were diagnosed with Parkinson’s disease (RD 0.1%, 95% CI 0.1–0.2; RR 1.40, 95% CI 1.21–1.63; OR 1.40, 95% CI 1.21–1.63; p < 0.001).

*Alzheimer’s Disease*

Among males, Alzheimer’s disease occurred in 262 of 62 259 arthropathic psoriasis patients (0.4%) compared with 177 of 62 301 controls (0.3%) (RD 0.1%, 95% CI 0.1–0.2; RR 1.48, 95% CI 1.22–1.79; OR 1.48, 95% CI 1.23–1.80; p < 0.001). In females, 397 of 87 359 arthropathic psoriasis patients (0.5%) versus 283 of 87 433 controls (0.3%) developed Alzheimer’s disease (RD 0.1%, 95% CI 0.1–0.2; RR 1.40, 95% CI 1.21–1.63; OR 1.41, 95% CI 1.21–1.64; p < 0.001).

*Transient Ischemic Attack (TIA)*

TIA was observed in 1 110 of 61 437 arthropathic psoriasis males (1.8%) compared with 621 of 62 189 controls (1.0%) (RD 0.8%, 95% CI 0.7–0.9; RR 1.81, 95% CI 1.64–2.00; OR 1.83, 95% CI 1.65–2.01; p < 0.001). In the female cohort, 1 632 of 86 076 arthropathic psoriasis patients (1.9%) versus 802 of 87 277 controls (0.9%) experienced TIA (RD 1.0%, 95% CI 0.9–1.1; RR 2.06, 95% CI 1.90–2.24; OR 2.08, 95% CI 1.92–2.27; p < 0.001).

*Stroke*

Stroke incidence was 1 674 of 60 983 arthropathic psoriasis males (2.7%) versus 1 280 of 61 841 controls (2.1%) (RD 0.7%, 95% CI 0.5–0.8; RR 1.33, 95% CI 1.24–1.42; OR 1.34, 95% CI 1.24–1.44; p < 0.001). Among females, 2 193 of 85 749 arthropathic psoriasis patients (2.6%) suffered stroke compared with 1 354 of 87 057 controls (1.6%) (RD 1.0%, 95% CI 0.9–1.1; RR 1.65, 95% CI 1.54–1.76; OR 1.66, 95% CI 1.55–1.78; p < 0.001).

**Addison’s Disease**

After 1:1 propensity score matching (matched on *current age* and *immune suppressants*), the male cohorts consisted of 19 453 Addison’s disease patients and 19 453 matched controls; the female cohorts comprised 29 301 Addison’s disease patients and 29 301 matched controls. After matching, the mean current age in males was 68.8 years (SD 9.3) in both cohorts (immune suppressant use 8.4% in both); in females, the mean current age was 67.0 years (SD 9.6) in both cohorts (immune suppressant use 8.5% in both).

*Parkinson’s Disease*

In the male cohort, 236 of 19 166 Addison’s disease patients (1.2%) and 122 of 19 383 controls (0.6%) developed Parkinson’s disease, yielding a risk difference (RD) of 0.6% (95% CI 0.4–0.8), a risk ratio (RR) of 1.96 (95% CI 1.58–2.43), and an odds ratio (OR) of 1.97 (95% CI 1.58–2.45); p < 0.001. In the female cohort, 244 of 29 056 Addison’s disease patients (0.8%) versus 99 of 29 250 controls (0.3%) were diagnosed with Parkinson’s disease (RD 0.5%, 95% CI 0.4–0.6; RR 2.48, 95% CI 1.97–3.14; OR 2.49, 95% CI 1.97–3.15; p < 0.001).

*Alzheimer’s Disease*

Among males, Alzheimer’s disease occurred in 114 of 19 349 Addison’s disease patients (0.6%) compared with 76 of 19 442 controls (0.4%) (RD 0.2%, 95% CI 0.1–0.3; RR 1.51, 95% CI 1.13–2.01; OR 1.51, 95% CI 1.13–2.02; p = 0.005). In females, 253 of 29 190 Addison’s disease patients (0.9%) versus 144 of 29 279 controls (0.5%) developed Alzheimer’s disease (RD 0.4%, 95% CI 0.2–0.5; RR 1.76, 95% CI 1.44–2.16; OR 1.77, 95% CI 1.44–2.17; p < 0.001).

*Transient Ischemic Attack (TIA)*

TIA was observed in 448 of 18 873 Addison’s disease males (2.4%) compared with 211 of 19 405 controls (1.1%) (RD 1.3%, 95% CI 1.0–1.5; RR 2.18, 95% CI 1.86–2.57; OR 2.21, 95% CI 1.88–2.61; p < 0.001). In the female cohort, 761 of 28 437 Addison’s disease patients (2.7%) versus 295 of 29 240 controls (1.0%) experienced TIA (RD 1.7%, 95% CI 1.4–1.9; RR 2.65, 95% CI 2.32–3.03; OR 2.69, 95% CI 2.36–3.09; p < 0.001).

*Stroke*

Stroke incidence was 919 of 18 179 Addison’s disease males (5.1%) versus 411 of 19 282 controls (2.1%) (RD 2.9%, 95% CI 2.5–3.3; RR 2.37, 95% CI 2.11–2.66; OR 2.44, 95% CI 2.17–2.76; p < 0.001). Among females, 1 303 of 27 832 Addison’s disease patients (4.7%) suffered stroke compared with 462 of 29 125 controls (1.6%) (RD 3.1%, 95% CI 2.8–3.4; RR 2.95, 95% CI 2.66–3.28; OR 3.05, 95% CI 2.74–3.39; p < 0.001).

**Psoriasis**

After 1:1 propensity score matching (matched on *current age* and *immune suppressants*), the male cohorts consisted of 266 380 psoriasis patients and 266 380 matched controls; the female cohorts comprised 318 414 psoriasis patients and 318 414 matched controls. The cohorts were well balanced on current age and immune suppressants after matching (standardized differences < 0.01). After matching, the mean current age of male psoriasis patients and matched controls was 66.5 years (SD 9.4) in both groups (immune suppressant use 2.7% in both); for females, the mean current age was 66.4 years (SD 9.4) in both cohorts (immune suppressant use 4.2% in both).

*Parkinson’s Disease*

In the male cohort, 2 041 of 264 813 psoriasis patients (0.8%) and 1 562 of 265 808 controls (0.6%) developed Parkinson’s disease, yielding a risk difference of 0.2% (95% CI 0.1–0.2), a risk ratio of 1.31 (95% CI 1.23–1.40), and an odds ratio of 1.31 (95% CI 1.23–1.40); p < 0.001. In the female cohort, 1 527 of 317 327 psoriasis patients (0.5%) versus 1 145 of 317 972 controls (0.4%) were diagnosed with Parkinson’s disease (RD 0.1%, 95% CI 0.1–0.2; RR 1.34, 95% CI 1.24–1.44; OR 1.34, 95% CI 1.24–1.45; p < 0.001).

*Alzheimer’s Disease*

Among males, Alzheimer’s disease occurred in 1 380 of 265 822 psoriasis patients (0.5%) compared with 861 of 266 249 controls (0.3%) (RD 0.2%, 95% CI 0.2–0.2; RR 1.61, 95% CI 1.48–1.75; OR 1.61, 95% CI 1.48–1.75; p < 0.001). In females, 1 965 of 317 702 psoriasis patients (0.6%) versus 1 183 of 318 169 controls (0.4%) developed Alzheimer’s disease (RD 0.2%, 95% CI 0.2–0.3; RR 1.66, 95% CI 1.55–1.79; OR 1.67, 95% CI 1.55–1.79; p < 0.001).

*Transient Ischemic Attack (TIA)*

TIA was observed in 4 893 of 262 490 psoriasis males (1.9%) compared with 2 620 of 265 728 controls (1.0%) (RD 0.9%, 95% CI 0.8–0.9; RR 1.89, 95% CI 1.80–1.98; OR 1.91, 95% CI 1.82–2.01; p < 0.001). In the female cohort, 6 063 of 313 375 psoriasis patients (1.9%) versus 3 103 of 317 738 controls (1.0%) experienced TIA (RD 1.0%, 95% CI 0.9–1.0; RR 1.98, 95% CI 1.90–2.07; OR 2.00, 95% CI 1.92–2.09; p < 0.001).

*Stroke*

Stroke incidence was 7 970 of 259 745 psoriasis males (3.1%) versus 5 528 of 264 394 controls (2.1%) (RD 1.0%, 95% CI 0.9–1.1; RR 1.47, 95% CI 1.42–1.52; OR 1.48, 95% CI 1.43–1.53; p < 0.001). Among females, 8 419 of 311 789 psoriasis patients (2.7%) suffered stroke compared with 4 970 of 316 876 controls (1.6%) (RD 1.1%, 95% CI 1.1–1.2; RR 1.72, 95% CI 1.66–1.78; OR 1.74, 95% CI 1.68–1.81; p < 0.001).

**Sjögren Syndrome**

After 1:1 propensity score matching (matched on *current age* and *immune suppressants*), the male cohorts consisted of 36 705 Sjögren syndrome patients and 36 705 matched controls; the female cohorts comprised 219 384 Sjögren syndrome patients and 219 384 matched controls. After matching, the mean current age of male Sjögren syndrome patients and matched controls was 68.9 years (SD 9.4) in both groups (immune suppressant use 9.1% in both); for females, the mean current age was 67.2 years (SD 9.4) in both cohorts (immune suppressant use 7.1% in both).

*Parkinson’s Disease*

In the male cohort, 498 of 36 154 Sjögren syndrome patients (1.4%) and 263 of 36 592 controls (0.7%) developed Parkinson’s disease, yielding a risk difference (RD) of 0.7% (95% CI 0.5–0.8), a risk ratio (RR) of 1.92 (95% CI 1.65–2.22), and an odds ratio (OR) of 1.93 (95% CI 1.66–2.24); p < 0.001. In the female cohort, 1 539 of 218 169 Sjögren syndrome patients (0.7%) versus 832 of 219 051 controls (0.4%) were diagnosed with Parkinson’s disease (RD 0.3%, 95% CI 0.3–0.4; RR 1.86, 95% CI 1.71–2.02; OR 1.86, 95% CI 1.71–2.03; p < 0.001).

*Alzheimer’s Disease*

Among males, Alzheimer’s disease occurred in 232 of 36 581 Sjögren syndrome patients (0.6%) compared with 169 of 36 674 controls (0.5%) (RD 0.2%, 95% CI 0.1–0.3; RR 1.38, 95% CI 1.13–1.68; OR 1.38, 95% CI 1.13–1.68; p = 0.001). In females, 1 466 of 218 940 Sjögren syndrome patients (0.7%) versus 936 of 219 191 controls (0.4%) developed Alzheimer’s disease (RD 0.2%, 95% CI 0.2–0.3; RR 1.57, 95% CI 1.45–1.70; OR 1.57, 95% CI 1.45–1.71; p < 0.001).

*Transient Ischemic Attack (TIA)*

TIA was observed in 906 of 35 588 Sjögren syndrome males (2.5%) compared with 436 of 36 612 controls (1.2%) (RD 1.4%, 95% CI 1.2–1.6; RR 2.14, 95% CI 1.91–2.39; OR 2.17, 95% CI 1.93–2.43; p < 0.001). In the female cohort, 5 562 of 214 348 Sjögren syndrome patients (2.6%) versus 2 235 of 218 868 controls (1.0%) experienced TIA (RD 1.6%, 95% CI 1.5–1.7; RR 2.54, 95% CI 2.42–2.67; OR 2.58, 95% CI 2.46–2.71; p < 0.001).

*Stroke*

Stroke incidence was 1 246 of 35 074 Sjögren syndrome males (3.6%) versus 775 of 36 368 controls (2.1%) (RD 1.4%, 95% CI 1.2–1.7; RR 1.67, 95% CI 1.53–1.82; OR 1.69, 95% CI 1.55–1.85; p < 0.001). Among females, 6 122 of 214 073 Sjögren syndrome patients (2.9%) suffered stroke compared with 3 710 of 218 200 controls (1.7%) (RD 1.2%, 95% CI 1.1–1.2; RR 1.68, 95% CI 1.62–1.75; OR 1.70, 95% CI 1.63–1.77; p < 0.001).

**Scleroderma**

After 1:1 propensity score matching (matched on *current age* and *immune suppressants*), the male cohorts consisted of 11 219 scleroderma patients and 11 219 matched controls; the female cohorts comprised 47 879 scleroderma patients and 47 879 matched controls. After matching, the mean current age in males was 68.2 years (SD 9.1) in both cohorts (immune suppressant use 9.8% in both); in females, the mean current age was 67.8 years (SD 9.4) in both cohorts (immune suppressant use 8.2% in both).

*Parkinson’s Disease*

In the male cohort, 84 of 11 118 scleroderma patients (0.8%) and 77 of 11 189 controls (0.7%) developed Parkinson’s disease, yielding a risk difference (RD) of 0.1% (95% CI −0.2–0.3), a risk ratio (RR) of 1.10 (95% CI 0.81–1.49), and an odds ratio (OR) of 1.10 (95% CI 0.81–1.50); p = 0.552. In the female cohort, 207 of 47 682 scleroderma patients (0.4%) versus 192 of 47 809 controls (0.4%) were diagnosed with Parkinson’s disease (RD 0.0%, 95% CI 0.0–0.1; RR 1.08, 95% CI 0.89–1.32; OR 1.08, 95% CI 0.89–1.32; p = 0.436).

*Alzheimer’s Disease*

Among males, Alzheimer’s disease occurred in 63 of 11 185 scleroderma patients (0.6%) compared with 36 of 11 210 controls (0.3%) (RD 0.2%, 95% CI 0.1–0.4; RR 1.75, 95% CI 1.17–2.64; OR 1.76, 95% CI 1.17–2.65; p = 0.006). In females, 227 of 47 799 scleroderma patients (0.5%) versus 209 of 47 842 controls (0.4%) developed Alzheimer’s disease (RD 0.0%, 95% CI 0.0–0.1; RR 1.09, 95% CI 0.90–1.31; OR 1.09, 95% CI 0.90–1.31; p = 0.382).

*Transient Ischemic Attack (TIA)*

TIA was observed in 181 of 11 005 scleroderma males (1.6%) compared with 122 of 11 200 controls (1.1%) (RD 0.6%, 95% CI 0.2–0.9; RR 1.51, 95% CI 1.20–1.90; OR 1.52, 95% CI 1.20–1.91; p < 0.001). In the female cohort, 953 of 47 160 scleroderma patients (2.0%) versus 493 of 47 778 controls (1.0%) experienced TIA (RD 1.0%, 95% CI 0.8–1.1; RR 1.96, 95% CI 1.76–2.18; OR 1.98, 95% CI 1.77–2.21; p < 0.001).

*Stroke*

Stroke incidence was 382 of 10 748 scleroderma males (3.6%) versus 258 of 11 124 controls (2.3%) (RD 1.2%, 95% CI 0.8–1.7; RR 1.53, 95% CI 1.31–1.79; OR 1.55, 95% CI 1.32–1.82; p < 0.001). Among females, 1 426 of 46 612 scleroderma patients (3.1%) suffered stroke compared with 764 of 47 618 controls (1.6%) (RD 1.5%, 95% CI 1.3–1.6; RR 1.91, 95% CI 1.75–2.08; OR 1.93, 95% CI 1.77–2.11; p < 0.001).

**Myositis**

After 1:1 propensity score matching (matched on *current age* and *immune suppressants*), the male cohorts consisted of 197 662 myositis patients and 197 662 matched controls; the female cohorts comprised 424 819 myositis patients and 424 819 matched controls. After matching, the mean current age in males was 66.4 years (SD 9.5) in both cohorts (immune suppressant use 2.8% in both); in females, the mean current age was 66.4 years (SD 9.4) in both cohorts (immune suppressant use 3.3% in both).

*Parkinson’s Disease*

In the male cohort, 2 207 of 196 101 myositis patients (1.1%) and 1 114 of 197 189 controls (0.6%) developed Parkinson’s disease, yielding a risk difference (RD) of 0.6% (95% CI 0.5–0.6), a risk ratio (RR) of 1.99 (95% CI 1.86–2.14), and an odds ratio (OR) of 2.01 (95% CI 1.86–2.16); p < 0.001. In the female cohort, 3 615 of 423 086 myositis patients (0.9%) versus 1 437 of 424 269 controls (0.3%) were diagnosed with Parkinson’s disease (RD 0.5%, 95% CI 0.5–0.5; RR 2.53, 95% CI 2.37–2.68; OR 2.54, 95% CI 2.39–2.70; p < 0.001).

*Alzheimer’s Disease*

Among males, Alzheimer’s disease occurred in 1 212 of 197 298 myositis patients (0.6%) compared with 596 of 197 563 controls (0.3%) (RD 0.3%, 95% CI 0.3–0.4; RR 2.04, 95% CI 1.85–2.25; OR 2.05, 95% CI 1.85–2.26; p < 0.001). In females, 3 273 of 424 214 myositis patients (0.8%) versus 1 678 of 424 519 controls (0.4%) developed Alzheimer’s disease (RD 0.4%, 95% CI 0.3–0.4; RR 1.96, 95% CI 1.84–2.08; OR 1.97, 95% CI 1.85–2.10; p < 0.001).

*Transient Ischemic Attack (TIA)*

TIA was observed in 5 912 of 192 781 myositis males (3.1%) compared with 1 867 of 197 224 controls (0.9%) (RD 2.1%, 95% CI 2.0–2.2; RR 3.25, 95% CI 3.08–3.42; OR 3.31, 95% CI 3.13–3.50; p < 0.001). In the female cohort, 13 781 of 416 166 myositis patients (3.3%) versus 4 019 of 423 968 controls (0.9%) experienced TIA (RD 2.4%, 95% CI 2.3–2.4; RR 3.49, 95% CI 3.37–3.61; OR 3.60, 95% CI 3.48–3.73; p < 0.001).

*Stroke*

Stroke incidence was 8 743 of 190 651 myositis males (4.6%) versus 4 086 of 196 151 controls (2.1%) (RD 2.5%, 95% CI 2.4–2.6; RR 2.21, 95% CI 2.14–2.29; OR 2.31, 95% CI 2.23–2.40; p < 0.001). Among females, 17 339 of 415 458 myositis patients (4.2%) suffered stroke compared with 6 890 of 422 637 controls (1.6%) (RD 2.5%, 95% CI 2.5–2.6; RR 2.56, 95% CI 2.49–2.64; OR 2.63, 95% CI 2.56–2.71; p < 0.001).

**Celiac Disease (ICD-10 K90.0; Age 50–85 years)**

After 1:1 propensity score matching (matched on *current age* and *immune suppressants*), the male cohorts consisted of 31 562 celiac disease patients and 31 562 matched controls; the female cohorts comprised 78 214 celiac disease patients and 78 214 matched controls. After matching, the mean current age in males was 66.5 years (SD 9.7) in both cohorts (immune suppressant use 2.1% in both); in females, the mean current age was 65.1 years (SD 9.6) in both cohorts (immune suppressant use 3.0% in both).

*Parkinson’s Disease*

In the male cohort, 301 of 31 293 celiac disease patients (1.0%) and 196 of 31 494 controls (0.6%) developed Parkinson’s disease, yielding a risk difference (RD) of 0.3% (95% CI 0.2–0.5), a risk ratio (RR) of 1.55 (95% CI 1.29–1.85), and an odds ratio (OR) of 1.55 (95% CI 1.30–1.86); p < 0.001. In the female cohort, 430 of 77 920 celiac disease patients (0.6%) versus 242 of 78 118 controls (0.3%) were diagnosed with Parkinson’s disease (RD 0.2%, 95% CI 0.2–0.3; RR 1.78, 95% CI 1.52–2.08; OR 1.79, 95% CI 1.52–2.09; p < 0.001).

*Alzheimer’s Disease*

Among males, Alzheimer’s disease occurred in 219 of 31 466 celiac disease patients (0.7%) compared with 107 of 31 538 controls (0.3%) (RD 0.4%, 95% CI 0.2–0.5; RR 2.05, 95% CI 1.63–2.58; OR 2.06, 95% CI 1.63–2.60; p < 0.001). In females, 517 of 78 027 celiac disease patients (0.7%) versus 242 of 78 161 controls (0.3%) developed Alzheimer’s disease (RD 0.4%, 95% CI 0.3–0.4; RR 2.14, 95% CI 1.84–2.49; OR 2.15, 95% CI 1.84–2.50; p < 0.001).

*Transient Ischemic Attack (TIA)*

TIA was observed in 617 of 31 014 celiac disease males (2.0%) compared with 348 of 31 498 controls (1.1%) (RD 0.9%, 95% CI 0.7–1.1; RR 1.80, 95% CI 1.58–2.05; OR 1.82, 95% CI 1.59–2.07; p < 0.001). In the female cohort, 1 544 of 77 030 celiac disease patients (2.0%) versus 678 of 78 043 controls (0.9%) experienced TIA (RD 1.1%, 95% CI 1.0–1.3; RR 2.31, 95% CI 2.11–2.53; OR 2.34, 95% CI 2.13–2.56; p < 0.001).

*Stroke*

Stroke incidence was 856 of 30 844 celiac disease males (2.8%) versus 672 of 31 330 controls (2.1%) (RD 0.6%, 95% CI 0.4–0.9; RR 1.29, 95% CI 1.17–1.43; OR 1.30, 95% CI 1.18–1.44; p < 0.001). Among females, 1 877 of 76 863 celiac disease patients (2.4%) suffered stroke compared with 1 261 of 77 841 controls (1.6%) (RD 0.8%, 95% CI 0.7–1.0; RR 1.51, 95% CI 1.40–1.62; OR 1.52, 95% CI 1.41–1.63; p < 0.001).

**Rheumatoid Arthritis (RA)**
After 1:1 propensity score matching, the male cohorts consisted of 168 380 patients with RA and 168 380 matched controls; the female cohorts comprised 467 380 patients with RA and 467 380 matched controls. The characteristics of both cohorts were well balanced on current age and immune suppressants use after matching (standardized differences < 0.001). After matching, the mean current age of male RA patients and matched controls was 69.7 years (SD 9.1) in both groups; for females, the mean current age was 68.4 years (SD 9.5) in both RA and control cohorts. Immune suppressants use was identical after matching (males: 3.4 % in both cohorts; females: 3.3 % in both cohorts).

*Parkinson’s Disease*
In the male cohort, 1 598 of 166 749 RA patients (1.0 %) and 1 288 of 167 890 controls (0.8 %) developed Parkinson’s disease, yielding a risk difference of 0.2 % (95 % CI 0.1–0.3), a risk ratio of 1.25 (95 % CI 1.16–1.34), and an odds ratio of 1.25 (95 % CI 1.16–1.35); p < 0.001. In the female cohort, 2 767 of 464 905 RA patients (0.6 %) versus 1 957 of 466 673 controls (0.4 %) were diagnosed with Parkinson’s disease (RD 0.2 %, 95 % CI 0.1–0.2; RR 1.42, 95 % CI 1.34–1.50; OR 1.42, 95 % CI 1.34–1.51; p < 0.001).

*Alzheimer’s Disease*Among males, Alzheimer’s disease occurred in 1 329 of 167 706 RA patients (0.8 %) compared with 667 of 168 262 controls (0.4 %) (RD 0.4 %, 95 % CI 0.3–0.4; RR 2.00, 95 % CI 1.82–2.19; OR 2.01, 95 % CI 1.83–2.20; p < 0.001). In females, 3 804 of 465 726 RA patients (0.8 %) versus 2 364 of 466 961 controls (0.5 %) developed Alzheimer’s disease (RD 0.3 %, 95 % CI 0.3–0.3; RR 1.61, 95 % CI 1.53–1.70; OR 1.62, 95 % CI 1.54–1.70; p < 0.001).

*Transient Ischemic Attack (TIA)*
TIA was observed in 3 684 of 164 918 RA males (2.2 %) compared with 1 862 of 167 940 controls (1.1 %) (RD 1.1 %, 95 % CI 1.0–1.2; RR 2.02, 95 % CI 1.90–2.13; OR 2.04, 95 % CI 1.93–2.16; p < 0.001). In the female cohort, 11 011 of 458 505 RA patients (2.4 %) versus 4 936 of 466 302 controls (1.1 %) experienced TIA (RD 1.3 %, 95 % CI 1.3–1.4; RR 2.27, 95 % CI 2.19–2.35; OR 2.30, 95 % CI 2.22–2.38; p < 0.001).

*Stroke*
Stroke incidence was 6 952 of 161 575 RA males (4.3 %) versus 3 981 of 166 967 controls (2.4 %) (RD 1.9 %, 95 % CI 1.8–2.0; RR 1.81, 95 % CI 1.74–1.88; OR 1.84, 95 % CI 1.77–1.92; p < 0.001). Among females, 17 063 of 452 908 RA patients (3.8 %) suffered stroke compared with 8 100 of 464 858 controls (1.7 %) (RD 2.0 %, 95 % CI 2.0–2.1; RR 2.16, 95 % CI 2.11–2.22; OR 2.21, 95 % CI 2.15–2.27; p < 0.001).

**Type 1 Diabetes Mellitus**

After 1:1 propensity score matching (matched on *current age* and *immune suppressants*), the male cohorts consisted of 295 332 T1DM patients and 295 332 matched controls; the female cohorts comprised 267 074 T1DM patients and 267 074 matched controls. After matching, the cohorts were well balanced on current age and immune suppressant use (standardized differences < 0.001). The mean current age in males was 68.0 years (SD 9.5) in both cohorts (immune suppressants 2.7% in both), and in females 68.3 years (SD 9.6) in both cohorts (immune suppressants 3.3% in both).

*Parkinson’s Disease*

In the male cohort, 3 150 of 293 248 T1DM patients (1.1%) versus 2 013 of 294 589 controls (0.7%) developed Parkinson’s disease, yielding a risk difference (RD) of 0.4% (95% CI 0.3–0.4), a risk ratio (RR) of 1.57 (95% CI 1.49–1.66), and an odds ratio (OR) of 1.58 (95% CI 1.49–1.67); p < 0.001. In the female cohort, 2 220 of 265 794 T1DM patients (0.8%) versus 1 076 of 266 687 controls (0.4%) were diagnosed with Parkinson’s disease (RD 0.4%, 95% CI 0.4–0.5; RR 2.07, 95% CI 1.93–2.23; OR 2.08, 95% CI 1.93–2.24; p < 0.001).

*Alzheimer’s Disease*

Among males, Alzheimer’s disease occurred in 2 347 of 294 423 T1DM patients (0.8%) compared with 1 082 of 295 175 controls (0.4%) (RD 0.4%, 95% CI 0.4–0.5; RR 2.17, 95% CI 2.02–2.34; OR 2.18, 95% CI 2.03–2.35; p < 0.001). In females, 2 906 of 266 008 T1DM patients (1.1%) versus 1 385 of 266 841 controls (0.5%) developed Alzheimer’s disease (RD 0.6%, 95% CI 0.5–0.6; RR 2.11, 95% CI 1.98–2.24; OR 2.12, 95% CI 1.98–2.26; p < 0.001).

*Transient Ischemic Attack (TIA)*

TIA was observed in 8 719 of 288 838 T1DM males (3.0%) compared with 3 044 of 294 593 controls (1.0%) (RD 2.0%, 95% CI 1.9–2.1; RR 2.92, 95% CI 2.80–3.04; OR 2.99, 95% CI 2.86–3.11; p < 0.001). In the female cohort, 9 390 of 260 449 T1DM patients (3.6%) versus 2 838 of 266 501 controls (1.1%) experienced TIA (RD 2.5%, 95% CI 2.5–2.6; RR 3.39, 95% CI 3.25–3.53; OR 3.47, 95% CI 3.33–3.62; p < 0.001).

*Stroke*

Stroke incidence was 18 566 of 279 461 T1DM males (6.6%) versus 6 272 of 292 984 controls (2.1%) (RD 4.5%, 95% CI 4.4–4.6; RR 3.11, 95% CI 3.02–3.19; OR 3.26, 95% CI 3.16–3.34; p < 0.001). Among females, 17 749 of 253 352 T1DM patients (7.0%) suffered stroke compared with 4 662 of 265 650 controls (1.8%) (RD 5.3%, 95% CI 5.1–5.4; RR 3.98, 95% CI 3.86–4.12; OR 4.22, 95% CI 4.08–4.37; p < 0.001).

**Autoimmune Thyroiditis**

After 1:1 propensity score matching (matched on *current age* and *immune suppressants*), the male cohorts consisted of 48 784 autoimmune thyroiditis patients and 48 784 matched controls; the female cohorts comprised 274 131 autoimmune thyroiditis patients and 274 131 matched controls. After matching, the mean current age in males was 66.4 years (SD 9.5) in both cohorts (immune suppressant use 2.9% in both); in females, the mean current age was 64.7 years (SD 9.3) in both cohorts (immune suppressant use 2.9% in both).

*Parkinson’s Disease*

In the male cohort, 466 of 48 347 autoimmune thyroiditis patients (1.0%) and 289 of 48 652 controls (0.6%) developed Parkinson’s disease, yielding a risk difference (RD) of 0.4% (95% CI 0.3–0.5), a risk ratio (RR) of 1.62 (95% CI 1.40–1.88), and an odds ratio (OR) of 1.63 (95% CI 1.41–1.89); p < 0.001. In the female cohort, 1 242 of 273 150 autoimmune thyroiditis patients (0.5%) versus 834 of 273 832 controls (0.3%) were diagnosed with Parkinson’s disease (RD 0.2%, 95% CI 0.1–0.2; RR 1.49, 95% CI 1.37–1.63; OR 1.50, 95% CI 1.37–1.63; p < 0.001).

*Alzheimer’s Disease*

Among males, Alzheimer’s disease occurred in 310 of 48 589 autoimmune thyroiditis patients (0.6%) compared with 154 of 48 764 controls (0.3%) (RD 0.3%, 95% CI 0.2–0.4; RR 2.02, 95% CI 1.67–2.45; OR 2.03, 95% CI 1.67–2.46; p < 0.001). In females, 1 385 of 273 491 autoimmune thyroiditis patients (0.5%) versus 885 of 273 939 controls (0.3%) developed Alzheimer’s disease (RD 0.2%, 95% CI 0.1–0.2; RR 1.57, 95% CI 1.44–1.71; OR 1.57, 95% CI 1.44–1.71; p < 0.001).

*Transient Ischemic Attack (TIA)*

TIA was observed in 987 of 47 648 autoimmune thyroiditis males (2.1%) compared with 479 of 48 672 controls (1.0%) (RD 1.1%, 95% CI 0.9–1.2; RR 2.11, 95% CI 1.89–2.35; OR 2.13, 95% CI 1.91–2.38; p < 0.001). In the female cohort, 4 858 of 269 576 autoimmune thyroiditis patients (1.8%) versus 2 397 of 273 623 controls (0.9%) experienced TIA (RD 0.9%, 95% CI 0.9–1.0; RR 2.06, 95% CI 1.96–2.16; OR 2.07, 95% CI 1.98–2.18; p < 0.001).

*Stroke*

Stroke incidence was 1 358 of 47 021 autoimmune thyroiditis males (2.9%) versus 996 of 48 394 controls (2.1%) (RD 0.8%, 95% CI 0.6–1.0; RR 1.40, 95% CI 1.29–1.52; OR 1.41, 95% CI 1.30–1.54; p < 0.001). Among females, 5 513 of 268 806 autoimmune thyroiditis patients (2.1%) suffered stroke compared with 4 000 of 272 881 controls (1.5%) (RD 0.6%, 95% CI 0.5–0.7; RR 1.40, 95% CI 1.34–1.46; OR 1.41, 95% CI 1.35–1.47; p < 0.001).

**Lupus Erythematosus**

After 1:1 propensity score matching (matched on *current age* and *immune suppressants*), the male cohorts consisted of 10 766 lupus erythematosus patients and 10 766 matched controls; the female cohorts comprised 51 057 lupus erythematosus patients and 51 057 matched controls. After matching, the mean current age in males was 66.6 years (SD 9.3) in both cohorts (immune suppressant use 8.7% in both); in females, the mean current age was 65.4 years (SD 9.3) in both cohorts (immune suppressant use 11.8% in both).

*Parkinson’s Disease*

In the male cohort, 91 of 10 705 lupus erythematosus patients (0.9%) and 64 of 10 733 controls (0.6%) developed Parkinson’s disease, yielding a risk difference (RD) of 0.3% (95% CI 0.0–0.5), a risk ratio (RR) of 1.43 (95% CI 1.04–1.96), and an odds ratio (OR) of 1.43 (95% CI 1.04–1.97); p = 0.028. In the female cohort, 240 of 50 868 lupus erythematosus patients (0.5%) versus 166 of 50 978 controls (0.3%) were diagnosed with Parkinson’s disease (RD 0.1%, 95% CI 0.1–0.2; RR 1.45, 95% CI 1.19–1.77; OR 1.45, 95% CI 1.19–1.77; p < 0.001).

*Alzheimer’s Disease*

Among males, Alzheimer’s disease occurred in 66 of 10 741 lupus erythematosus patients (0.6%) compared with 44 of 10 760 controls (0.4%) (RD 0.2%, 95% CI 0.0–0.4; RR 1.50, 95% CI 1.03–2.20; OR 1.51, 95% CI 1.03–2.21; p = 0.035). In females, 292 of 50 955 lupus erythematosus patients (0.6%) versus 200 of 51 021 controls (0.4%) developed Alzheimer’s disease (RD 0.2%, 95% CI 0.1–0.3; RR 1.46, 95% CI 1.22–1.75; OR 1.46, 95% CI 1.22–1.75; p < 0.001).

*Transient Ischemic Attack (TIA)*

TIA was observed in 256 of 10 519 lupus erythematosus males (2.4%) compared with 114 of 10 739 controls (1.1%) (RD 1.4%, 95% CI 1.0–1.7; RR 2.29, 95% CI 1.84–2.86; OR 2.33, 95% CI 1.86–2.91; p < 0.001). In the female cohort, 1 514 of 49 800 lupus erythematosus patients (3.0%) versus 501 of 50 942 controls (1.0%) experienced TIA (RD 2.1%, 95% CI 1.9–2.2; RR 3.10, 95% CI 2.79–3.41; OR 3.15, 95% CI 2.85–3.50; p < 0.001).

*Stroke*

Stroke incidence was 460 of 10 322 lupus erythematosus males (4.5%) versus 241 of 10 674 controls (2.3%) (RD 2.2%, 95% CI 1.7–2.7; RR 1.97, 95% CI 1.69–2.30; OR 2.02, 95% CI 1.72–2.36; p < 0.001). Among females, 2 328 of 49 187 lupus erythematosus patients (4.7%) suffered stroke compared with 806 of 50 808 controls (1.6%) (RD 3.1%, 95% CI 2.9–3.4; RR 2.99, 95% CI 2.76–3.23; OR 3.09, 95% CI 2.84–3.34; p < 0.001).

**Ulcerative Colitis**

After 1:1 propensity score matching (matched on *current age* and *immune suppressants*), the male cohorts consisted of 128 029 ulcerative colitis patients and 128 029 matched controls; the female cohorts comprised 147 287 ulcerative colitis patients and 147 287 matched controls. After matching, the mean current age in males was 67.0 years (SD 9.6) in both cohorts (immune suppressant use 4.5% in both); in females, the mean current age was 67.0 years (SD 9.6) in both cohorts (immune suppressant use 5.1% in both).

*Parkinson’s Disease*

In the male cohort, 999 of 126 917 ulcerative colitis patients (0.8%) and 800 of 127 713 controls (0.6%) developed Parkinson’s disease, yielding a risk difference (RD) of 0.2% (95% CI 0.1–0.2), a risk ratio (RR) of 1.26 (95% CI 1.15–1.38), and an odds ratio (OR) of 1.26 (95% CI 1.15–1.38); p < 0.001. In the female cohort, 710 of 146 497 ulcerative colitis patients (0.5%) versus 577 of 147 083 controls (0.4%) were diagnosed with Parkinson’s disease (RD 0.1%, 95% CI 0.0–0.1; RR 1.24, 95% CI 1.11–1.38; OR 1.24, 95% CI 1.11–1.38; p < 0.001).

*Alzheimer’s Disease*

Among males, Alzheimer’s disease occurred in 682 of 127 641 ulcerative colitis patients (0.5%) compared with 431 of 127 961 controls (0.3%) (RD 0.2%, 95% CI 0.1–0.2; RR 1.59, 95% CI 1.41–1.79; OR 1.59, 95% CI 1.41–1.79; p < 0.001). In females, 1 004 of 146 751 ulcerative colitis patients (0.7%) versus 659 of 147 169 controls (0.4%) developed Alzheimer’s disease (RD 0.2%, 95% CI 0.2–0.3; RR 1.53, 95% CI 1.39–1.69; OR 1.53, 95% CI 1.39–1.69; p < 0.001).

*Transient Ischemic Attack (TIA)*

TIA was observed in 2 118 of 125 800 ulcerative colitis males (1.7%) compared with 1 358 of 127 721 controls (1.1%) (RD 0.6%, 95% CI 0.5–0.7; RR 1.58, 95% CI 1.48–1.70; OR 1.59, 95% CI 1.49–1.71; p < 0.001). In the female cohort, 2 901 of 144 231 ulcerative colitis patients (2.0%) versus 1 457 of 146 978 controls (1.0%) experienced TIA (RD 1.0%, 95% CI 0.9–1.1; RR 2.03, 95% CI 1.91–2.16; OR 2.05, 95% CI 1.92–2.18; p < 0.001).

*Stroke*

Stroke incidence was 3 519 of 123 978 ulcerative colitis males (2.8%) versus 2 677 of 127 010 controls (2.1%) (RD 0.7%, 95% CI 0.6–0.9; RR 1.35, 95% CI 1.28–1.41; OR 1.36, 95% CI 1.29–1.43; p < 0.001). Among females, 3 993 of 142 915 ulcerative colitis patients (2.8%) suffered stroke compared with 2 482 of 146 577 controls (1.7%) (RD 1.1%, 95% CI 1.0–1.2; RR 1.65, 95% CI 1.57–1.73; OR 1.67, 95% CI 1.59–1.75; p < 0.001).

**Crohn’s Disease**

After 1:1 propensity score matching (matched on *current age* and *immune suppressants*), the male cohorts consisted of 86 759 Crohn’s disease patients and 86 759 matched controls; the female cohorts comprised 116 119 Crohn’s disease patients and 116 119 matched controls. After matching, the mean current age was 65.8 years (SD 9.7) in both male cohorts (immune suppressants 5.1% in both) and 65.6 years (SD 9.6) in both female cohorts (immune suppressants 5.3% in both).

*Parkinson’s Disease*

In the male cohort, 679 of 86 174 Crohn’s disease patients (0.8%) and 475 of 86 555 controls (0.5%) developed Parkinson’s disease, yielding a risk difference of 0.2% (95% CI 0.2–0.3), a risk ratio of 1.44 (95% CI 1.28–1.61), and an odds ratio of 1.44 (95% CI 1.28–1.62); p < 0.001. In the female cohort, 577 of 115 692 Crohn’s disease patients (0.5%) versus 380 of 115 984 controls (0.3%) were diagnosed with Parkinson’s disease (RD 0.2%, 95% CI 0.1–0.2; RR 1.52, 95% CI 1.34–1.73; OR 1.52, 95% CI 1.34–1.74; p < 0.001).

*Alzheimer’s Disease*

Among males, Alzheimer’s disease occurred in 393 of 86 577 Crohn’s disease patients (0.5%) compared with 257 of 86 713 controls (0.3%) (RD 0.2%, 95% CI 0.1–0.2; RR 1.53, 95% CI 1.31–1.79; OR 1.53, 95% CI 1.31–1.80; p < 0.001). In females, 664 of 115 861 Crohn’s disease patients (0.6%) versus 441 of 116 044 controls (0.4%) developed Alzheimer’s disease (RD 0.2%, 95% CI 0.1–0.2; RR 1.51, 95% CI 1.34–1.70; OR 1.51, 95% CI 1.34–1.70; p < 0.001).

*Transient Ischemic Attack (TIA)*

TIA was observed in 1 601 of 85 749 Crohn’s disease males (1.9%) compared with 829 of 86 531 controls (1.0%) (RD 0.9%, 95% CI 0.8–1.0; RR 1.95, 95% CI 1.79–2.12; OR 1.97, 95% CI 1.81–2.14; p < 0.001). In the female cohort, 2 297 of 114 566 Crohn’s disease patients (2.0%) versus 1 075 of 115 890 controls (0.9%) experienced TIA (RD 1.1%, 95% CI 1.0–1.2; RR 2.16, 95% CI 2.01–2.32; OR 2.18, 95% CI 2.03–2.35; p < 0.001).

*Stroke*

Stroke incidence was 2 597 of 84 920 Crohn’s disease males (3.1%) versus 1 743 of 86 094 controls (2.0%) (RD 1.0%, 95% CI 0.9–1.2; RR 1.51, 95% CI 1.42–1.61; OR 1.53, 95% CI 1.43–1.62; p < 0.001). Among females, 3 195 of 113 771 Crohn’s disease patients (2.8%) suffered stroke compared with 1 778 of 115 531 controls (1.5%) (RD 1.3%, 95% CI 1.1–1.4; RR 1.82, 95% CI 1.72–1.93; OR 1.85, 95% CI 1.74–1.96; p < 0.001).

**EXPERIMENT 2**

**Chronic Inflammatory Demyelinating Polyneuropathy (CIDP)**After 1:1 propensity score matching, the male cohorts consisted of 863 CIDP patients in the No CRP cohort and 863 matched CIDP patients in the CRP cohort; the female cohorts comprised 669 patients in each cohort. A small number of patients were excluded because the index event occurred more than 20 years prior (males: 2 in Cohort 1 and 5 in Cohort 2; females: 1 and 5, respectively). Baseline characteristics were well balanced after matching (after-match standardized mean differences up to 0.062). After matching, the mean current age was 69.8 years (SD 9.0) in No-CRP males and 69.7 years (SD 9.1) in CRP males; in females, mean current age was 67.3 years (SD 9.6) in No-CRP and 67.4 years (SD 9.5) in CRP.

There was not enough outcome data for Alzheimer’s and Parkinson’s disease.

*Transient Ischemic Attack (TIA)*In the male cohort, 13 of 825 No-CRP patients (1.6%) and 13 of 810 CRP patients (1.6%) developed TIA (RD −0.03%, 95% CI −1.24 to 1.18; RR 0.98, 95% CI 0.46–2.10; OR 0.98, 95% CI 0.45–2.13; *p* = 0.962). In the female cohort, 10 of 634 No-CRP patients (1.6%) versus 11 of 626 CRP patients (1.8%) developed TIA (RD −0.18%, 95% CI −1.59 to 1.23; RR 0.90, 95% CI 0.38–2.10; OR 0.90, 95% CI 0.38–2.12; *p* = 0.803).

*Stroke*
In the male cohort, 24 of 810 No-CRP patients (3.0%) versus 29 of 771 CRP patients (3.8%) developed I63 stroke (RD −0.80%, 95% CI −2.58 to 0.98; RR 0.79, 95% CI 0.46–1.34; OR 0.78, 95% CI 0.45–1.35; *p* = 0.378). In the female cohort, 23 of 621 No-CRP patients (3.7%) versus 30 of 607 CRP patients (4.9%) developed I63 stroke (RD −1.24%, 95% CI −3.51 to 1.04; RR 0.75, 95% CI 0.44–1.28; OR 0.74, 95% CI 0.42–1.29; *p* = 0.286).

**Guillain–Barré Syndrome (GBS)**
After 1:1 propensity score matching, the male cohorts consisted of 1 123 GBS patients with low CRP (≤ 1 mg/L) and 1 123 matched GBS patients with elevated CRP (3–10 mg/L); the female cohorts comprised 959 low-CRP GBS patients and 959 matched high-CRP GBS patients. After matching, age was closely balanced (current age SMD 0.011 in males and 0.006 in females; age at index SMD 0.006 in males and 0.009 in females), while immune suppressants showed small residual imbalance (SMD 0.097 in males and 0.020 in females). After matching, the mean current age was 67.6 years (SD 9.5) in the male low-CRP cohort and 67.5 years (SD 9.5) in the male high-CRP cohort; for females, mean current age was 67.2 years (SD 9.8) and 67.1 years (SD 9.8) in the low- and high-CRP cohorts, respectively.

There was not enough outcome data for Alzheimer’s and Parkinson’s disease.

*Transient Ischemic Attack (TIA)*In the male cohort, 13 of 1 077 patients (1.2 %) in the low-CRP group versus 17 of 1 080 (1.6 %) in the high-CRP group developed TIA (RD -0.4 %, 95 % CI -1.4–0.7; RR 0.77, 95 % CI 0.37–1.57; OR 0.76, 95 % CI 0.37–1.58; p = 0.467). In the female cohort, 18 of 918 patients (2.0 %) versus 16 of 914 (1.8 %) developed TIA (RD 0.2 %, 95 % CI -1.0–1.4; RR 1.12, 95 % CI 0.57–2.18; OR 1.12, 95 % CI 0.57–2.22; p = 0.739).

*Stroke*
In the male cohort, 23 of 997 patients (2.3 %) in the low-CRP group versus 41 of 1 015 (4.0 %) in the high-CRP group developed stroke (RD -1.7 %, 95 % CI -3.3–-0.2; RR 0.57, 95 % CI 0.35–0.95; OR 0.56, 95 % CI 0.33–0.95; p = 0.027). In the female cohort, 23 of 869 patients (2.6 %) versus 32 of 862 (3.7 %) developed stroke (RD -1.1 %, 95 % CI -3.0–0.7; RR 0.71, 95 % CI 0.42–1.21; OR 0.71, 95 % CI 0.41–1.22; p = 0.206).

**Myasthenia Gravis**After 1:1 propensity score matching, the male cohorts consisted of 1 848 myasthenia gravis patients with no CRP (≤ 1.0 mg/L) and 1 848 matched myasthenia gravis patients with elevated CRP (3–10 mg/L); the female cohorts comprised 2 324 patients in each group. The characteristics of both cohorts were well balanced after matching across current age, age at index, and immune suppressant use (standardized differences ≤ 0.020). After matching, the mean current age in the male cohorts was 70.6 years (SD 9.2) in the no-CRP group and 70.6 years (SD 9.4) in the CRP group; for females, the mean current age was 68.4 years (SD 9.9) and 68.4 years (SD 9.9), respectively. Immune suppressant use after matching was 378/1 848 (20.5 %) versus 383/1 848 (20.7 %) in males, and 568/2 324 (24.4 %) versus 548/2 324 (23.6 %) in females.

There was not enough outcome data for Alzheimer’s disease.

*Parkinson’s Disease*In the male cohort, 22 of 1 783 (1.2 %) in the no-CRP group and 16 of 1 800 (0.9 %) in the CRP group developed Parkinson’s disease, yielding a risk difference of 0.3 % (95 % CI -0.3–1.0), a risk ratio of 1.39 (95 % CI 0.73–2.63), and an odds ratio of 1.39 (95 % CI 0.73–2.66); p = 0.313. In the female cohort, 22 of 2 276 (1.0 %) versus 12 of 2 287 (0.5 %) developed Parkinson’s disease (RD 0.4 %, 95 % CI -0.1–0.9; RR 1.84, 95 % CI 0.91–3.71; OR 1.85, 95 % CI 0.91–3.75; p = 0.083).

*Transient Ischemic Attack (TIA)*In the male cohort, 34 of 1 720 (2.0 %) in the no-CRP group and 37 of 1 736 (2.1 %) in the CRP group developed TIA, yielding a risk difference of -0.2 % (95 % CI -1.1–0.8), a risk ratio of 0.93 (95 % CI 0.59–1.47), and an odds ratio of 0.93 (95 % CI 0.58–1.48); p = 0.749. In the female cohort, 37 of 2 171 (1.7 %) versus 41 of 2 164 (1.9 %) developed TIA (RD -0.2 %, 95 % CI -1.0–0.6; RR 0.90, 95 % CI 0.58–1.40; OR 0.90, 95 % CI 0.57–1.41; p = 0.637).

*Stroke*
In the male cohort, 47 of 1 576 (3.0 %) in the no-CRP group and 67 of 1 594 (4.2 %) in the CRP group developed ischemic stroke (I63), yielding a risk difference of -1.2 % (95 % CI -2.5–0.1), a risk ratio of 0.71 (95 % CI 0.49–1.02), and an odds ratio of 0.70 (95 % CI 0.48–1.02); p = 0.065. In the female cohort, 55 of 2 063 (2.7 %) versus 63 of 2 082 (3.0 %) developed ischemic stroke (I63) (RD -0.4 %, 95 % CI -1.4–0.7; RR 0.88, 95 % CI 0.62–1.26; OR 0.88, 95 % CI 0.61–1.27; p = 0.486).

**Multiple Sclerosis (MS)**
After 1:1 propensity score matching, the male cohorts consisted of 2 590 patients with MS and low CRP (No CRP) and 2 590 matched MS patients with elevated CRP (CRP); the female cohorts comprised 7 497 MS patients (No CRP) and 7 497 matched MS patients (CRP). After matching, female cohorts were closely balanced on age, age at index, and immune suppressants (standardized differences 0.006–0.028). In males, balance was slightly less tight but still small-to-moderate (standardized differences 0.019–0.045). After matching, the mean current age in males was 64.5 years (SD 9.0) in the No-CRP cohort versus 64.7 years (SD 9.1) in the CRP cohort; for females, the mean current age was 64.0 years (SD 9.0) in both cohorts. Mean age at index was 57.9 years (SD 9.7) vs 58.1 years (SD 9.7) in males, and 57.4 years (SD 9.6) vs 57.3 years (SD 9.5) in females. Immune suppressants were recorded in 147/2 590 (5.7%) vs 121/2 590 (4.7%) of males, and 702/7 497 (9.4%) vs 643/7 497 (8.6%) of females (No CRP vs CRP).

*Alzheimer’s Disease*In the female cohort, 21 of 7 465 No-CRP patients (0.3%) versus 25 of 7 475 CRP patients (0.3%) developed Alzheimer’s disease (RD −0.1%, 95% CI −0.2 to 0.1; RR 0.84, 95% CI 0.47–1.50; OR 0.84, 95% CI 0.47–1.50; p = 0.558). There was not enough outcome data for the male cohort.

*Parkinson’s Disease*In the male cohort, 23 of 2 537 No-CRP patients (0.9%) and 20 of 2 531 CRP patients (0.8%) developed Parkinson’s disease, yielding an RD of 0.1% (95% CI −0.4 to 0.6), an RR of 1.15 (95% CI 0.64–2.07), and an OR of 1.15 (95% CI 0.64–2.08); p = 0.652. In the female cohort, 39 of 7 421 No-CRP patients (0.5%) versus 31 of 7 411 CRP patients (0.4%) were diagnosed with Parkinson’s disease (RD 0.1%, 95% CI −0.1 to 0.3; RR 1.26, 95% CI 0.79–2.01; OR 1.26, 95% CI 0.78–2.02; p = 0.341).

*Transient Ischemic Attack (TIA)*TIA was observed in 42 of 2 479 No-CRP males (1.7%) compared with 44 of 2 485 CRP males (1.8%) (RD −0.1%, 95% CI −0.8 to 0.6; RR 0.96, 95% CI 0.63–1.46; OR 0.96, 95% CI 0.62–1.46; p = 0.837). In the female cohort, 88 of 7 191 No-CRP patients (1.2%) versus 98 of 7 152 CRP patients (1.4%) experienced TIA (RD −0.1%, 95% CI −0.5 to 0.2; RR 0.89, 95% CI 0.67–1.19; OR 0.89, 95% CI 0.67–1.19; p = 0.438).

*Stroke*
Stroke incidence was 50 of 2 376 No-CRP males (2.1%) versus 63 of 2 333 CRP males (2.7%) (RD −0.6%, 95% CI −1.5 to 0.3; RR 0.78, 95% CI 0.54–1.12; OR 0.77, 95% CI 0.53–1.13; p = 0.181). Among females, 143 of 7 014 No-CRP patients (2.0%) suffered stroke compared with 176 of 6 923 CRP patients (2.5%) (RD −0.5%, 95% CI −1.0 to −0.0; RR 0.80, 95% CI 0.65–1.00; OR 0.80, 95% CI 0.64–1.00; p = 0.047).

**Vitiligo**
After 1:1 propensity score matching, the male cohorts consisted of 1 429 vitiligo patients in the No-CRP group and 1 429 in the CRP group; the female cohorts comprised 2 438 and 2 438 patients, respectively. Baseline characteristics were well balanced after matching (female standardized mean differences ≤ 0.010; male ≤ 0.033). After matching, the mean current age of male vitiligo patients was 67.4 years (SD 9.3) in the No-CRP group and 67.7 years (SD 9.2) in the CRP group; for females, the mean current age was 66.4 years (SD 9.6) and 66.4 years (SD 9.3), respectively.

There was not enough outcome data for Alzheimer’s and Parkinson’s disease.

*Transient Ischemic Attack (TIA)*In the male cohort, 18 of 1 363 patients in the No-CRP group (1.3 %) and 16 of 1 363 in the CRP group (1.2 %) developed TIA, yielding an RD of 0.1 % (95 % CI -0.7–1.0), an RR of 1.12 (95 % CI 0.58–2.20), and an OR of 1.13 (95 % CI 0.57–2.22); p = 0.730. In the female cohort, 43 of 2 303 No-CRP patients (1.9 %) versus 47 of 2 321 in the CRP group (2.0 %) developed TIA (RD -0.2 %, 95 % CI -1.0–0.6; RR 0.92, 95 % CI 0.61–1.39; OR 0.92, 95 % CI 0.61–1.40; p = 0.698).

*Stroke*

In the male cohort, cerebral infarction (I63) occurred in 35 of 1 356 No-CRP patients (2.6 %) and 35 of 1 324 in the CRP group (2.6 %), corresponding to an RD of -0.1 % (95 % CI -1.3–1.1), an RR of 0.98 (95 % CI 0.61–1.55), and an OR of 0.98 (95 % CI 0.61–1.57); p = 0.919. In the female cohort, 44 of 2 329 No-CRP patients (1.9 %) versus 45 of 2 304 in the CRP group (2.0 %) developed cerebral infarction (RD -0.1 %, 95 % CI -0.9–0.7; RR 0.97, 95 % CI 0.64–1.46; OR 0.97, 95 % CI 0.64–1.47; p = 0.874).

**Rheumatoid Vasculitis**After 1:1 propensity score matching, the male cohorts consisted of 307 patients with *rheumatoid vasculitis (Male), No CRP* and 307 matched patients with *rheumatoid vasculitis (Male), CRP*; the female cohorts comprised 1 035 patients with *rheumatoid vasculitis (Female), No CRP* and 1 035 matched patients with *rheumatoid vasculitis (Female), CRP*. After matching, the mean current age of male patients was 69.1 years (SD 8.7) in the *No CRP* cohort and 68.3 years (SD 8.7) in the *CRP* cohort (standardized mean difference [SMD] for current age 0.100). For females, the mean current age was 67.6 years (SD 9.4) in the *No CRP* cohort and 67.5 years (SD 9.4) in the *CRP* cohort (SMD for current age 0.008).

There was not enough outcome data for Alzheimer’s and Parkinson’s disease.

Transient Ischemic Attack (TIA)
*Female cohort (per the provided Measures of Association table):* 15 of 984 *No CRP* patients (1.5%) versus 12 of 973 *CRP* patients (1.2%) experienced TIA, yielding a risk difference of 0.3% (95% CI −0.7 to 1.3), a risk ratio of 1.24 (95% CI 0.58–2.63), and an odds ratio of 1.24 (95% CI 0.58–2.66); p = 0.581.

*Stroke*
*Female cohort (per the provided Measures of Association table):* 23 of 967 *No CRP* patients (2.4%) versus 28 of 957 *CRP* patients (2.9%) developed the outcome, yielding a risk difference of −0.5% (95% CI −2.0 to 0.9), a risk ratio of 0.81 (95% CI 0.47–1.40), and an odds ratio of 0.81 (95% CI 0.46–1.41); p = 0.455.

**Grave’s Disease**
After 1:1 propensity score matching, the male cohorts consisted of 9 837 patients with Grave’s disease and CRP ≤ 1.0 mg/L and 9 837 matched patients with CRP 3.0–10.0 mg/L; the female cohorts comprised 25 040 patients in each CRP group. The characteristics of both cohorts were well balanced after matching (standardized differences: males—current age 0.022, age at index 0.020, immune suppressants 0.047; females—current age 0.003, age at index 0.003, immune suppressants 0.015). After matching, the mean current age of males was 68.9 years (SD 9.7) in the low-CRP cohort and 69.1 years (SD 9.6) in the higher-CRP cohort; for females, the mean current age was 67.5 years (SD 9.8) in both cohorts.

*Parkinson’s Disease*

In the male cohort, 77 of 9 643 low-CRP patients (0.8 %) and 67 of 9 679 higher-CRP patients (0.7 %) developed Parkinson’s disease, yielding a risk difference of 0.1 % (95 % CI −0.1–0.3), a risk ratio of 1.154 (95 % CI 0.832–1.599), and an odds ratio of 1.155 (95 % CI 0.831–1.604); p = 0.390. In the female cohort, 131 of 24 723 low-CRP patients (0.5 %) versus 94 of 24 795 higher-CRP patients (0.4 %) were diagnosed with Parkinson’s disease (RD 0.2 %, 95 % CI 0.0–0.3; RR 1.398, 95 % CI 1.073–1.821; OR 1.400, 95 % CI 1.073–1.825; p = 0.013).

*Alzheimer’s Disease*

Among males, Alzheimer’s disease occurred in 70 of 9 722 low-CRP patients (0.7 %) compared with 56 of 9 741 higher-CRP patients (0.6 %) (RD 0.1 %, 95 % CI −0.1–0.4; RR 1.252, 95 % CI 0.882–1.778; OR 1.254, 95 % CI 0.882–1.784; p = 0.207). In females, 144 of 24 834 low-CRP patients (0.6 %) versus 103 of 24 876 higher-CRP patients (0.4 %) developed Alzheimer’s disease (RD 0.2 %, 95 % CI 0.0–0.3; RR 1.400, 95 % CI 1.088–1.802; OR 1.403, 95 % CI 1.089–1.808; p = 0.009).

*Transient Ischemic Attack (TIA)*

TIA was observed in 159 of 9 339 low-CRP males (1.7 %) compared with 128 of 9 332 higher-CRP males (1.4 %) (RD 0.3 %, 95 % CI −0.0–0.7; RR 1.241, 95 % CI 0.985–1.564; OR 1.245, 95 % CI 0.985–1.575; p = 0.066). In the female cohort, 396 of 23 719 low-CRP patients (1.7 %) versus 409 of 23 748 higher-CRP patients (1.7 %) experienced TIA (RD −0.1 %, 95 % CI −0.3–0.2; RR 0.969, 95 % CI 0.845–1.112; OR 0.969, 95 % CI 0.843–1.114; p = 0.657).

*Stroke*

Cerebral infarction occurred in 351 of 8 556 low-CRP males (4.1 %) versus 322 of 8 617 higher-CRP males (3.7 %), yielding RD 0.4 % (95 % CI −0.2–0.9), RR 1.098 (95 % CI 0.947–1.273), and OR 1.102 (95 % CI 0.944–1.286); p = 0.217. Among females, 571 of 23 098 low-CRP patients (2.5 %) suffered cerebral infarction compared with 598 of 22 999 higher-CRP patients (2.6 %) (RD −0.1 %, 95 % CI −0.4–0.2; RR 0.951, 95 % CI 0.849–1.065; OR 0.950, 95 % CI 0.845–1.066; p = 0.382).

**Vasculitis**

After 1:1 propensity score matching, the male cohorts consisted of 1 649 patients with vasculitis and low CRP (“No CRP”) and 1 649 matched patients with elevated CRP (“CRP”); the female cohorts comprised 3 648 and 3 648 patients, respectively. In males, the cohorts were closely balanced after matching (standardized mean differences 0.016 for current age, 0.015 for age at index, and 0.037 for immune suppressants); the mean current age after matching was 68.7 years (SD 9.5) in the No-CRP cohort and 68.6 years (SD 9.4) in the CRP cohort. In females, matching achieved very close balance (e.g., standardized mean difference 0.006 for current age); the mean current age after matching was 68.0 years (SD 9.8) in both cohorts.

*Alzheimer’s Disease*In the female cohort, Alzheimer’s disease occurred in 21 of 3 635 No-CRP patients (0.6%) versus 15 of 3 630 CRP patients (0.4%) (RD 0.2%, 95% CI -0.2–0.5; RR 1.398, 95% CI 0.722–2.708; OR 1.400, 95% CI 0.721–2.721; p = 0.318). There was not enough outcome data in the male cohort for comparison.

*Parkinson’s Disease*
In the female cohort, Parkinson’s disease was observed in 20 of 3 616 No-CRP patients (0.6%) versus 14 of 3 618 CRP patients (0.4%) (RD 0.2%, 95% CI -0.1–0.5; RR 1.429, 95% CI 0.723–2.825; OR 1.432, 95% CI 0.722–2.839; p = 0.302). There was not enough outcome data in the male cohort for comparison.

*Transient Ischemic Attack (TIA)*In males, TIA occurred in 31 of 1 551 No-CRP patients (2.0%) versus 30 of 1 563 CRP patients (1.9%) (RD 0.1%, 95% CI -0.9–1.1; RR 1.041, 95% CI 0.633–1.712; OR 1.042, 95% CI 0.628–1.730; p = 0.873). In females, TIA was observed in 64 of 3 440 No-CRP patients (1.9%) versus 51 of 3 427 CRP patients (1.5%) (RD 0.4%, 95% CI -0.2–1.0; RR 1.250, 95% CI 0.868–1.801; OR 1.255, 95% CI 0.866–1.819; p = 0.229).

*Stroke*In males, cerebral infarction occurred in 42 of 1 540 No-CRP patients (2.7%) versus 56 of 1 522 CRP patients (3.7%) (RD -1.0%, 95% CI -2.2–0.3; RR 0.741, 95% CI 0.500–1.099; OR 0.734, 95% CI 0.489–1.102; p = 0.135). In females, cerebral infarction occurred in 70 of 3 420 No-CRP patients (2.0%) versus 108 of 3 402 CRP patients (3.2%) (RD -1.1%, 95% CI -1.9–-0.4; RR 0.645, 95% CI 0.479–0.868; OR 0.637, 95% CI 0.470–0.864; p = 0.003).

**Dermatomyositis**
After 1:1 propensity score matching, the male cohorts consisted of 2 192 patients with dermatomyositis and no CRP and 2 192 matched dermatomyositis patients with CRP; the female cohorts comprised 4 699 patients with dermatomyositis and no CRP and 4 699 matched dermatomyositis patients with CRP. The characteristics of both cohorts were well balanced after matching (maximum standardized mean difference 0.0042 in males and 0.0048 in females). After matching, the mean current age in the male No-CRP and CRP cohorts was 68.3 years (SD 9.5) and 68.3 years (SD 9.5), respectively; in females, the mean current age was 66.7 years (SD 9.4) in the No-CRP cohort and 66.7 years (SD 9.5) in the CRP cohort.

*Alzheimer’s Disease*
In the female cohort, 18 of 4 680 No-CRP patients (0.4%) and 16 of 4 680 CRP patients (0.3%) developed Alzheimer’s disease (RD 0.04%, 95% CI −0.20 to 0.29; RR 1.13, 95% CI 0.57–2.20; OR 1.13, 95% CI 0.57–2.21; p = 0.731). There was not enough outcome data in the male cohort for comparison.

There was not enough outcome data for Parkinson’s disease.

*Transient Ischemic Attack (TIA)*In the male cohort, 16 of 2 109 No-CRP patients (0.8%) and 23 of 2 125 CRP patients (1.1%) experienced TIA (RD −0.32%, 95% CI −0.90 to 0.25; RR 0.70, 95% CI 0.37–1.32; OR 0.70, 95% CI 0.37–1.33; p = 0.270). In the female cohort, 55 of 4 520 No-CRP patients (1.2%) versus 55 of 4 543 CRP patients (1.2%) experienced TIA (RD 0.01%, 95% CI −0.44 to 0.46; RR 1.01, 95% CI 0.69–1.46; OR 1.01, 95% CI 0.69–1.46; p = 0.979).

*Stroke*

In the male cohort, cerebral infarction occurred in 68 of 1 992 No-CRP patients (3.4%) and 66 of 1 995 CRP patients (3.3%) (RD 0.11%, 95% CI −1.01 to 1.22; RR 1.03, 95% CI 0.74–1.44; OR 1.03, 95% CI 0.73–1.46; p = 0.854). In the female cohort, 99 of 4 391 No-CRP patients (2.3%) versus 93 of 4 452 CRP patients (2.1%) developed cerebral infarction (RD 0.17%, 95% CI −0.44 to 0.77; RR 1.08, 95% CI 0.82–1.43; OR 1.08, 95% CI 0.80–1.46; p = 0.593).

**Arthropathic Psoriasis**
After 1:1 propensity score matching, the male cohorts consisted of 7 799 arthropathic psoriasis patients with CRP ≤ 1.0 mg/L and 7 799 matched arthropathic psoriasis patients with CRP 3.0–10.0 mg/L; the female cohorts comprised 10 209 patients in each CRP group. The characteristics of both cohorts were well balanced after matching (standardized differences ≤ 0.019). After matching, the mean current age of male patients was 65.5 years (SD 9.0) in both CRP groups; for females, the mean current age was 65.3 years (SD 9.1) in the CRP ≤1.0 group and 65.2 years (SD 9.1) in the CRP 3.0–10.0 group.

*Parkinson’s Disease*

In the male cohort, 36 of 7 752 low-CRP patients (0.5 %) and 26 of 7 742 higher-CRP patients (0.3 %) developed Parkinson’s disease, yielding a risk difference of 0.1 % (95 % CI −0.1–0.3), a risk ratio of 1.383 (95 % CI 0.836–2.288), and an odds ratio of 1.385 (95 % CI 0.835–2.295); p = 0.205. In the female cohort, 38 of 10 169 low-CRP patients (0.4 %) versus 30 of 10 169 higher-CRP patients (0.3 %) were diagnosed with Parkinson’s disease (RD 0.1 %, 95 % CI −0.1–0.2; RR 1.267, 95 % CI 0.785–2.043; OR 1.268, 95 % CI 0.785–2.048; p = 0.331).

*Alzheimer’s Disease*

Among males, Alzheimer’s disease occurred in 15 of 7 783 low-CRP patients (0.2 %) compared with 15 of 7 792 higher-CRP patients (0.2 %) (RD 0.0 %, 95 % CI −0.1–0.1; RR 1.001, 95 % CI 0.490–2.047; OR 1.001, 95 % CI 0.489–2.049; p = 0.997). In females, 24 of 10 187 low-CRP patients (0.2 %) versus 26 of 10 190 higher-CRP patients (0.3 %) developed Alzheimer’s disease (RD −0.0 %, 95 % CI −0.2–0.1; RR 0.923, 95 % CI 0.531–1.607; OR 0.923, 95 % CI 0.530–1.609; p = 0.778).

*Transient Ischemic Attack (TIA)*

TIA was observed in 89 of 7 615 low-CRP males (1.2 %) compared with 81 of 7 640 higher-CRP males (1.1 %) (RD 0.1 %, 95 % CI −0.2–0.4; RR 1.102, 95 % CI 0.817–1.487; OR 1.104, 95 % CI 0.815–1.494; p = 0.523). In the female cohort, 108 of 9 930 low-CRP patients (1.1 %) versus 103 of 9 934 higher-CRP patients (1.0 %) experienced TIA (RD 0.1 %, 95 % CI −0.2–0.3; RR 1.049, 95 % CI 0.802–1.372; OR 1.050, 95 % CI 0.800–1.377; p = 0.727).

*Stroke*

Stroke (I63) incidence was 89 of 7 581 low-CRP males (1.2 %) versus 110 of 7 564 higher-CRP males (1.5 %) (RD −0.3 %, 95 % CI −0.6–0.1; RR 0.807, 95 % CI 0.612–1.066; OR 0.805, 95 % CI 0.608–1.066; p = 0.130). Among females, 115 of 9 930 low-CRP patients (1.2 %) suffered I63 compared with 155 of 9 878 higher-CRP patients (1.6 %) (RD −0.4 %, 95 % CI −0.7–−0.1; RR 0.738, 95 % CI 0.581–0.938; OR 0.735, 95 % CI 0.577–0.937; p = 0.013).

**Addison’s Disease**

After 1:1 propensity score matching, the male cohorts consisted of 1 070 patients in the No-CRP cohort and 1 070 matched patients in the CRP cohort; the female cohorts comprised 2 341 patients in each group (No-CRP: 2 341 matched to CRP: 2 341; pre-matching CRP cohort n = 4 112). The characteristics of both cohorts were well balanced after matching on current age, age at index, and immune suppressant use (standardized differences ≤ 0.057). After matching, the mean current age in males was 66.3 years (SD 9.2) in the No-CRP cohort versus 65.8 years (SD 8.8) in the CRP cohort; in females, the mean current age was 66.9 years (SD 9.5) versus 67.1 years (SD 9.4) in No-CRP and CRP cohorts, respectively.

*Parkinson’s Disease*In the female cohort, 14 of 2 304 No-CRP patients (0.6 %) versus 15 of 2 312 CRP patients (0.6 %) developed Parkinson’s disease (RD -0.0 %, 95 % CI -0.5–0.4; RR 0.94, 95 % CI 0.45–1.94; OR 0.94, 95 % CI 0.45–1.94; p = 0.860). There was not enough outcome data in the male cohort.

*Alzheimer’s Disease*
In the female cohort, Alzheimer’s disease occurred in 14 of 2 323 No-CRP patients (0.6 %) compared with 14 of 2 320 CRP patients (0.6 %) (RD -0.0 %, 95 % CI -0.4–0.4; RR 1.00, 95 % CI 0.48–2.09; OR 1.00, 95 % CI 0.47–2.10; p = 0.997). There was not enough outcome data in the male cohort.

*Transient Ischemic Attack (TIA)*In the male cohort, TIA was observed in 10 of 1 015 No-CRP patients (1.0 %) versus 16 of 1 022 CRP patients (1.6 %) (RD -0.6 %, 95 % CI -1.6–0.4; RR 0.63, 95 % CI 0.29–1.38; OR 0.63, 95 % CI 0.28–1.39; p = 0.243). In the female cohort, 48 of 2 193 No-CRP patients (2.2 %) versus 49 of 2 196 CRP patients (2.2 %) experienced TIA (RD -0.0 %, 95 % CI -0.9–0.8; RR 0.98, 95 % CI 0.66–1.45; OR 0.98, 95 % CI 0.66–1.47; p = 0.924).

*Stroke*

In the male cohort, cerebral infarction occurred in 29 of 999 No-CRP patients (2.9 %) compared with 18 of 979 CRP patients (1.8 %) (RD 1.1 %, 95 % CI -0.3–2.4; RR 1.58, 95 % CI 0.88–2.82; OR 1.60, 95 % CI 0.88–2.89; p = 0.120). In the female cohort, 54 of 2 146 No-CRP patients (2.5 %) versus 68 of 2 116 CRP patients (3.2 %) developed cerebral infarction (RD -0.7 %, 95 % CI -1.7–0.3; RR 0.78, 95 % CI 0.55–1.11; OR 0.78, 95 % CI 0.54–1.12; p = 0.172).

**Psoriasis**
After 1:1 propensity score matching, the male cohorts consisted of 17 257 psoriasis patients with CRP ≤ 1.0 mg/L and 17 257 matched psoriasis patients with CRP 3.0–10.0 mg/L; the female cohorts comprised 21 518 patients in each CRP group. The characteristics of both cohorts were well balanced after matching (standardized differences: males—current age 0.004, age at index 0.001, immune suppressants 0.008; females—current age 0.001, age at index 0.002, immune suppressants < 0.001). After matching, the mean current age of males was 66.5 years (SD 9.3) in both groups; for females, the mean current age was 66.3 years (SD 9.4) in both groups.

*Parkinson’s Disease*
In the male cohort, 80 of 17 094 low-CRP patients (0.5 %) and 77 of 17 095 higher-CRP patients (0.5 %) developed Parkinson’s disease, yielding a risk difference of 0.0 % (95 % CI −0.1–0.2), a risk ratio of 1.039 (95 % CI 0.760–1.420), and an odds ratio of 1.039 (95 % CI 0.759–1.422); p = 0.810. In the female cohort, 85 of 21 391 low-CRP patients (0.4 %) versus 52 of 21 400 higher-CRP patients (0.2 %) were diagnosed with Parkinson’s disease (RD 0.2 %, 95 % CI 0.0–0.3; RR 1.635, 95 % CI 1.159–2.308; OR 1.638, 95 % CI 1.159–2.314; p = 0.005).

*Alzheimer’s Disease*
Among males, Alzheimer’s disease occurred in 41 of 17 196 low-CRP patients (0.2 %) compared with 41 of 17 210 higher-CRP patients (0.2 %) (RD 0.0 %, 95 % CI −0.1–0.1; RR 1.001, 95 % CI 0.649–1.542; OR 1.001, 95 % CI 0.649–1.544; p = 0.997). In females, 76 of 21 444 low-CRP patients (0.4 %) versus 69 of 21 443 higher-CRP patients (0.3 %) developed Alzheimer’s disease (RD 0.0 %, 95 % CI −0.1–0.1; RR 1.101, 95 % CI 0.795–1.525; OR 1.102, 95 % CI 0.795–1.527; p = 0.561).

*Transient Ischemic Attack (TIA)*TIA was observed in 215 of 16 705 low-CRP males (1.3 %) compared with 174 of 16 769 higher-CRP males (1.0 %) (RD 0.2 %, 95 % CI 0.0–0.5; RR 1.240, 95 % CI 1.017–1.513; OR 1.244, 95 % CI 1.017–1.520; p = 0.033). In the female cohort, 252 of 20 805 low-CRP patients (1.2 %) versus 261 of 20 742 higher-CRP patients (1.3 %) experienced TIA (RD −0.0 %, 95 % CI −0.3–0.2; RR 0.963, 95 % CI 0.810–1.143; OR 0.962, 95 % CI 0.808–1.145; p = 0.664).

*Stroke*
Stroke (I63) incidence was 267 of 16 527 low-CRP males (1.6 %) versus 356 of 16 434 higher-CRP males (2.2 %) (RD −0.6 %, 95 % CI −0.8–−0.3; RR 0.746, 95 % CI 0.637–0.873; OR 0.742, 95 % CI 0.632–0.870; p < 0.001). Among females, 280 of 20 738 low-CRP patients (1.4 %) suffered I63 compared with 401 of 20 651 higher-CRP patients (1.9 %) (RD −0.6 %, 95 % CI −0.8–−0.3; RR 0.695, 95 % CI 0.598–0.809; OR 0.691, 95 % CI 0.593–0.806; p < 0.001).

**Sjögren Syndrome**
After 1:1 propensity score matching, the male cohorts consisted of 5,342 Sjögren syndrome patients with CRP ≤1.0 mg/L and 5,342 matched Sjögren syndrome patients with CRP 3.0–10.0 mg/L; the female cohorts comprised 36,495 patients in each CRP group. The characteristics of both cohorts were well balanced after matching (standardized differences: males—current age 0.008, age at index 0.007, immune suppressants 0.009; females—current age 0.010, age at index 0.008, immune suppressants 0.004). After matching, the mean current age in males was 69.1 years (SD 9.2 vs 9.5) and in females 67.0 years (SD 9.4 in both groups).

*Parkinson’s Disease*

In the male cohort, 43 of 5,238 low-CRP patients (0.8%) and 44 of 5,232 higher-CRP patients (0.8%) developed Parkinson’s disease, yielding a risk difference (RD) −0.0% (95% CI −0.4 to 0.3), a risk ratio (RR) 0.976 (95% CI 0.642–1.484), and an odds ratio (OR) 0.976 (95% CI 0.640–1.488); p = 0.910. In the female cohort, 190 of 36,167 low-CRP patients (0.5%) versus 136 of 36,229 higher-CRP patients (0.4%) were diagnosed with Parkinson’s disease (RD 0.1%, 95% CI 0.1–0.2; RR 1.399, 95% CI 1.123–1.743; OR 1.402, 95% CI 1.124–1.748; p = 0.003).

*Alzheimer’s Disease*

Among males, Alzheimer’s disease occurred in 18 of 5,312 low-CRP patients (0.3%) compared with 16 of 5,306 higher-CRP patients (0.3%) (RD 0.0%, 95% CI −0.2 to 0.3; RR 1.124, 95% CI 0.574–2.201; OR 1.124, 95% CI 0.573–2.207; p = 0.734). In females, 135 of 36,380 low-CRP patients (0.4%) versus 112 of 36,398 higher-CRP patients (0.3%) developed Alzheimer’s disease (RD 0.1%, 95% CI −0.0 to 0.1; RR 1.206, 95% CI 0.939–1.549; OR 1.207, 95% CI 0.939–1.551; p = 0.142).

*Transient Ischemic Attack (TIA)*

TIA was observed in 89 of 5,000 low-CRP males (1.8%) compared with 83 of 5,061 higher-CRP males (1.6%) (RD 0.1%, 95% CI −0.4 to 0.6; RR 1.085, 95% CI 0.807–1.460; OR 1.087, 95% CI 0.804–1.470; p = 0.588). In the female cohort, 547 of 34,692 low-CRP patients (1.6%) versus 561 of 35,011 higher-CRP patients (1.6%) experienced TIA (RD −0.0%, 95% CI −0.2 to 0.2; RR 0.984, 95% CI 0.876–1.106; OR 0.984, 95% CI 0.874–1.108; p = 0.787).

Stroke

Cerebral infarction occurred in 144 of 4,925 low-CRP males (2.9%) versus 166 of 4,888 higher-CRP males (3.4%) (RD −0.5%, 95% CI −1.2 to 0.2; RR 0.861, 95% CI 0.691–1.072; OR 0.857, 95% CI 0.683–1.075; p = 0.181). Among females, 541 of 35,014 low-CRP patients (1.5%) developed I63 compared with 713 of 34,832 higher-CRP patients (2.0%) (RD −0.5%, 95% CI −0.7 to −0.3; RR 0.755, 95% CI 0.676–0.843; OR 0.751, 95% CI 0.671–0.841; p < 0.001).

**Scleroderma**
After 1:1 propensity score matching, the male cohorts consisted of 1 469 scleroderma patients with CRP ≤ 1.0 mg/L and 1 469 matched scleroderma patients with CRP 3.0–10.0 mg/L; the female cohorts comprised 6 849 patients in each CRP group. Characteristics were balanced after matching (e.g., standardized mean differences for current age were 0.030 in males and 0.013 in females). After matching, the mean current age of the male cohorts was 68.6 years (SD 9.5) in the low-CRP group and 68.3 years (SD 9.3) in the higher-CRP group; in females, mean current age was 67.5 years (SD 9.5) versus 67.6 years (SD 9.4), respectively.

*Parkinson’s Disease*In the female cohort, 16 of 6 789 low-CRP patients (0.2 %) versus 17 of 6 809 higher-CRP patients (0.2 %) were diagnosed with Parkinson’s disease (RD -0.0 % [95 % CI -0.2–0.2]; RR 0.94 [95 % CI 0.48–1.87]; OR 0.94 [95 % CI 0.48–1.87]; p = 0.868). There was not enough outcome data in the male cohort.

*Alzheimer’s Disease*
In the male cohort, Alzheimer’s disease occurred in 10 of 1 454 low-CRP patients (0.7 %) and 12 of 1 455 higher-CRP patients (0.8 %) (RD -0.1 % [95 % CI -0.8–0.5]; RR 0.83 [95 % CI 0.36–1.92]; OR 0.83 [95 % CI 0.36–1.93]; p = 0.670). In the female cohort, 18 of 6 823 low-CRP patients (0.3 %) versus 14 of 6 824 higher-CRP patients (0.2 %) developed Alzheimer’s disease (RD 0.1 % [95 % CI -0.1–0.2]; RR 1.29 [95 % CI 0.64–2.58]; OR 1.29 [95 % CI 0.64–2.59]; p = 0.479).

*Transient Ischemic Attack (TIA)*In the male cohort, 15 of 1 419 low-CRP patients (1.1 %) and 15 of 1 421 higher-CRP patients (1.1 %) experienced TIA (RD 0.0 % [95 % CI -0.8–0.8]; RR 1.00 [95 % CI 0.49–2.04]; OR 1.00 [95 % CI 0.49–2.06]; p = 0.997). In the female cohort, 72 of 6 650 low-CRP patients (1.1 %) versus 93 of 6 655 higher-CRP patients (1.4 %) had TIA (RD -0.3 % [95 % CI -0.7–0.1]; RR 0.78 [95 % CI 0.57–1.05]; OR 0.77 [95 % CI 0.57–1.05]; p = 0.101).

*Stroke*

In the male cohort, cerebral infarction occurred in 45 of 1 312 low-CRP patients (3.4 %) compared with 44 of 1 332 higher-CRP patients (3.3 %) (RD 0.1 % [95 % CI -1.2–1.5]; RR 1.04 [95 % CI 0.69–1.56]; OR 1.04 [95 % CI 0.68–1.59]; p = 0.857). In the female cohort, 102 of 6 480 low-CRP patients (1.6 %) versus 138 of 6 521 higher-CRP patients (2.1 %) developed cerebral infarction (RD -0.5 % [95 % CI -1.0–-0.1]; RR 0.74 [95 % CI 0.58–0.96]; OR 0.74 [95 % CI 0.57–0.96]; p = 0.022).

**Myositis**

After 1:1 propensity score matching, the male cohorts consisted of 20,291 patients with myositis and CRP ≤1.0 mg/L and 20,291 matched patients with CRP 3.0–10.0 mg/L; the female cohorts comprised 47,651 patients with CRP ≤1.0 mg/L and 47,651 matched patients with CRP 3.0–10.0 mg/L. After matching, cohort characteristics were well balanced: in males, mean current age was 67.8 years (SD 9.3) vs 67.9 (SD 9.3) and immune suppressants 7.2% vs 6.7% (standardized differences: current age 0.016, age at index 0.015, immune suppressants 0.019); in females, mean current age was 67.3 years (SD 9.5) in both cohorts and immune suppressants 11.6% vs 11.6% (standardized differences 0.001 for current age, age at index, and immune suppressants).

*Parkinson’s Disease*

In the male cohort, 137 of 19,987 CRP ≤1.0 patients (0.7%) and 107 of 20,001 CRP 3.0–10.0 patients (0.5%) developed Parkinson’s disease, yielding RD 0.2% (95% CI −0.0 to 0.3), RR 1.281 (95% CI 0.996–1.649), and OR 1.283 (95% CI 0.996–1.654); p = 0.053. In the female cohort, 245 of 47,125 CRP ≤1.0 patients (0.5%) versus 177 of 47,232 CRP 3.0–10.0 patients (0.4%) developed Parkinson’s disease (RD 0.1%, 95% CI 0.1–0.2; RR 1.387, 95% CI 1.144–1.683; OR 1.389, 95% CI 1.145–1.686; p = 0.001).

*Alzheimer’s Disease*

Among males, Alzheimer’s disease occurred in 82 of 20,219 CRP ≤1.0 patients (0.4%) compared with 65 of 20,215 CRP 3.0–10.0 patients (0.3%) (RD 0.1%, 95% CI −0.0 to 0.2; RR 1.261, 95% CI 0.911–1.746; OR 1.262, 95% CI 0.911–1.749; p = 0.160). In females, 182 of 47,452 CRP ≤1.0 patients (0.4%) versus 163 of 47,472 CRP 3.0–10.0 patients (0.3%) developed Alzheimer’s disease (RD 0.0%, 95% CI −0.0 to 0.1; RR 1.117, 95% CI 0.905–1.379; OR 1.117, 95% CI 0.904–1.381; p = 0.304).

*Transient Ischemic Attack (TIA)*

TIA was observed in 446 of 19,011 CRP ≤1.0 males (2.3%) compared with 358 of 19,142 CRP 3.0–10.0 males (1.9%) (RD 0.5%, 95% CI 0.2–0.8; RR 1.254, 95% CI 1.093–1.439; OR 1.261, 95% CI 1.095–1.451; p = 0.001). In the female cohort, 901 of 44,764 CRP ≤1.0 patients (2.0%) versus 788 of 45,108 CRP 3.0–10.0 patients (1.7%) experienced TIA (RD 0.3%, 95% CI 0.1–0.4; RR 1.152, 95% CI 1.048–1.267; OR 1.155, 95% CI 1.049–1.272; p = 0.003).

*Stroke*

Cerebral infarction occurred in 491 of 18,927 CRP ≤1.0 males (2.6%) versus 573 of 18,648 CRP 3.0–10.0 males (3.1%), yielding RD −0.5% (95% CI −0.8 to −0.1), RR 0.844 (95% CI 0.750–0.951), and OR 0.840 (95% CI 0.743–0.949); p = 0.005. In females, 854 of 45,301 CRP ≤1.0 patients (1.9%) versus 1,067 of 44,848 CRP 3.0–10.0 patients (2.4%) developed I63 (RD −0.5%, 95% CI −0.7 to −0.3; RR 0.792, 95% CI 0.725–0.866; OR 0.788, 95% CI 0.720–0.863; p < 0.001).

**Celiac Disease**

After 1:1 propensity score matching, the male cohorts consisted of 1,927 patients with celiac disease and CRP ≤1.0 mg/L and 1,927 matched patients with CRP 3.0–10.0 mg/L; the female cohorts comprised 5,929 patients with CRP ≤1.0 mg/L and 5,929 matched patients with CRP 3.0–10.0 mg/L. After matching, mean current age in males was 66.4 years (SD 9.5) vs 66.3 years (SD 9.6), and mean age at index was 60.7 (SD 9.9) vs 60.7 (SD 10.1); immune suppressants were recorded in 167/1,927 (8.7%) vs 154/1,927 (8.0%) (standardized mean differences after matching: current age 0.013, age at index 0.009, immune suppressants 0.024). In females, mean current age was 65.0 years (SD 9.4) vs 65.0 years (SD 9.5); mean age at index was 58.8 (SD 10.1) vs 58.8 (SD 10.0); immune suppressants were 11.5% in both cohorts.

*Parkinson’s Disease*

In the male cohort, 11 of 1,899 CRP ≤1.0 patients (0.6%) and 10 of 1,908 CRP 3.0–10.0 patients (0.5%) developed Parkinson’s disease, yielding RD 0.1% (95% CI −0.4 to 0.5), RR 1.11 (95% CI 0.47–2.60), and OR 1.11 (95% CI 0.47–2.61); p = 0.818. In the female cohort, 21 of 5,897 CRP ≤1.0 patients (0.4%) versus 17 of 5,904 CRP 3.0–10.0 patients (0.3%) developed Parkinson’s disease (RD 0.1%, 95% CI −0.1 to 0.3; RR 1.237, 95% CI 0.653–2.342; OR 1.238, 95% CI 0.652–2.348; p = 0.513).

*Alzheimer’s Disease*

In the female cohort, Alzheimer’s disease occurred in 20 of 5,911 CRP ≤1.0 patients (0.3%) compared with 15 of 5,903 CRP 3.0–10.0 patients (0.3%) (RD 0.1%, 95% CI −0.1 to 0.3; RR 1.332, 95% CI 0.682–2.598; OR 1.333, 95% CI 0.682–2.605; p = 0.400). There was not enough outcome data in the male cohort.

*Transient Ischemic Attack (TIA)*

TIA was observed in 28 of 1,855 CRP ≤1.0 males (1.5%) compared with 33 of 1,855 CRP 3.0–10.0 males (1.8%) (RD −0.3%, 95% CI −1.1 to 0.5; RR 0.85, 95% CI 0.51–1.40; OR 0.85, 95% CI 0.51–1.41; p = 0.519). In the female cohort, 66 of 5,728 CRP ≤1.0 patients (1.2%) versus 88 of 5,703 CRP 3.0–10.0 patients (1.5%) experienced TIA (RD −0.4%, 95% CI −0.8 to 0.0; RR 0.747, 95% CI 0.544–1.025; OR 0.744, 95% CI 0.539–1.026; p = 0.070).

*Stroke*

Cerebral infarction occurred in 26 of 1,847 CRP ≤1.0 males (1.4%) versus 59 of 1,832 CRP 3.0–10.0 males (3.2%), yielding RD −1.8% (95% CI −2.8 to −0.8), RR 0.44 (95% CI 0.28–0.69), and OR 0.43 (95% CI 0.27–0.68); p < 0.001. In females, 88 of 5,731 CRP ≤1.0 patients (1.5%) versus 110 of 5,678 CRP 3.0–10.0 patients (1.9%) developed I63 (RD −0.4%, 95% CI −0.9 to 0.1; RR 0.793, 95% CI 0.600–1.047; OR 0.789, 95% CI 0.595–1.047; p = 0.100).

**Rheumatoid Arthritis**

After 1:1 propensity score matching, the male cohorts consisted of 19,966 rheumatoid arthritis patients with CRP ≤1.0 mg/L and 19,966 matched rheumatoid arthritis patients with CRP 3.0–10.0 mg/L; the female cohorts comprised 58,316 patients with CRP ≤1.0 mg/L and 58,316 matched patients with CRP 3.0–10.0 mg/L. Cohort characteristics were well balanced after matching (standardized differences: males—current age 0.001, age at index 0.002, immune suppressants 0.002; females—current age 0.001, age at index <0.001, immune suppressants 0.001). After matching, mean current age was 69.3 years (SD 9.3 vs 9.2) in males and 68.2 years (SD 9.4 in both groups) in females; mean age at index was 62.6 (SD 9.9 vs 9.8) in males and 61.4 (SD 10.0 in both groups) in females. Immune suppressants were recorded in 1,871/19,966 (9.4%) vs 1,857/19,966 (9.3%) in males and 7,543/58,316 (12.9%) vs 7,514/58,316 (12.9%) in females (CRP ≤1.0 vs CRP 3.0–10.0).

*Parkinson’s Disease*

In the male cohort, 122/19,719 (0.6%) CRP ≤1.0 patients and 112/19,753 (0.6%) CRP 3.0–10.0 patients developed Parkinson’s disease, yielding RD 0.1% (95% CI −0.1 to 0.2), RR 1.091 (95% CI 0.845–1.409), and OR 1.092 (95% CI 0.844–1.412); p = 0.504. In the female cohort, 222/57,898 (0.4%) CRP ≤1.0 patients versus 176/57,935 (0.3%) CRP 3.0–10.0 patients were diagnosed with Parkinson’s disease (RD 0.1%, 95% CI 0.0 to 0.1; RR 1.262, 95% CI 1.036–1.538; OR 1.263, 95% CI 1.036–1.540; p = 0.021).

*Alzheimer’s Disease*

Among males, Alzheimer’s disease occurred in 129/19,822 (0.7%) CRP ≤1.0 patients compared with 85/19,866 (0.4%) CRP 3.0–10.0 patients (RD 0.2%, 95% CI 0.1 to 0.4; RR 1.521, 95% CI 1.158–1.999; OR 1.524, 95% CI 1.158–2.006; p = 0.002). In females, 289/57,983 (0.5%) CRP ≤1.0 patients versus 233/58,069 (0.4%) CRP 3.0–10.0 patients developed Alzheimer’s disease (RD 0.1%, 95% CI 0.0 to 0.2; RR 1.242, 95% CI 1.046–1.476; OR 1.243, 95% CI 1.046–1.478; p = 0.013).

*Transient Ischemic Attack (TIA)*

TIA was observed in 247/19,307 (1.3%) CRP ≤1.0 males compared with 266/19,347 (1.4%) CRP 3.0–10.0 males (RD −0.1%, 95% CI −0.3 to 0.1; RR 0.930, 95% CI 0.783–1.105; OR 0.930, 95% CI 0.781–1.107; p = 0.412). Among females, 740/56,362 (1.3%) CRP ≤1.0 patients versus 792/56,426 (1.4%) CRP 3.0–10.0 patients experienced TIA (RD −0.1%, 95% CI −0.2 to 0.0; RR 0.935, 95% CI 0.847–1.033; OR 0.935, 95% CI 0.845–1.034; p = 0.188).

*Stroke*

Cerebral infarction occurred in 549/18,561 (3.0%) CRP ≤1.0 males versus 479/18,740 (2.6%) CRP 3.0–10.0 males (RD 0.4%, 95% CI 0.1 to 0.7; RR 1.157, 95% CI 1.025–1.306; OR 1.162, 95% CI 1.026–1.316; p = 0.018). In females, 1,165/55,338 (2.1%) CRP ≤1.0 patients versus 1,232/55,499 (2.2%) CRP 3.0–10.0 patients developed cerebral infarction (RD −0.1%, 95% CI −0.3 to 0.1; RR 0.948, 95% CI 0.876–1.027; OR 0.947, 95% CI 0.874–1.027; p = 0.190).

**Type 1 Diabetes mellitus**

After 1:1 propensity score matching, the male cohorts consisted of 14,458 patients with type 1 diabetes mellitus and CRP ≤1.0 mg/L and 14,458 matched patients with CRP 3.0–10.0 mg/L; the female cohorts comprised 12,790 patients with CRP ≤1.0 mg/L and 12,790 matched patients with CRP 3.0–10.0 mg/L. Cohort characteristics were well balanced after matching (standardized differences: males—current age 0.004, age at index 0.004, immune suppressants 0.005; females—current age <0.001, age at index 0.002, immune suppressants <0.001). After matching, mean current age was 68.6 years (SD 9.5) in both male cohorts and 68.9 years (SD 9.7) in both female cohorts; mean age at index was 62.0 (SD 9.9) in both male cohorts and 62.2 (SD 10.1) in both female cohorts.

*Parkinson’s Disease*

In the male cohort, 121 of 14,263 CRP ≤1.0 patients (0.8%) and 113 of 14,258 CRP 3.0–10.0 patients (0.8%) developed Parkinson’s disease, yielding RD 0.1% (95% CI −0.2 to 0.3), RR 1.070 (95% CI 0.829–1.382), and OR 1.071 (95% CI 0.828–1.386); p = 0.601. In the female cohort, 83 of 12,626 CRP ≤1.0 patients (0.7%) versus 73 of 12,630 CRP 3.0–10.0 patients (0.6%) were diagnosed with Parkinson’s disease (RD 0.1%, 95% CI −0.1 to 0.3; RR 1.137, 95% CI 0.831–1.556; OR 1.138, 95% CI 0.830–1.560; p = 0.421).

*Alzheimer’s Disease*

Among males, Alzheimer’s disease occurred in 101 of 14,369 CRP ≤1.0 patients (0.7%) compared with 83 of 14,383 CRP 3.0–10.0 patients (0.6%) (RD 0.1%, 95% CI −0.1 to 0.3; RR 1.218, 95% CI 0.912–1.627; OR 1.220, 95% CI 0.911–1.632; p = 0.181). In females, 102 of 12,677 CRP ≤1.0 patients (0.8%) versus 84 of 12,692 CRP 3.0–10.0 patients (0.7%) developed Alzheimer’s disease (RD 0.1%, 95% CI −0.1 to 0.4; RR 1.216, 95% CI 0.912–1.621; OR 1.217, 95% CI 0.911–1.627; p = 0.183).

*Transient Ischemic Attack (TIA)*

Among females, 275 of 11,944 CRP ≤1.0 patients (2.3%) versus 292 of 11,921 CRP 3.0–10.0 patients (2.4%) experienced TIA (RD −0.1%, 95% CI −0.5 to 0.2; RR 0.940, 95% CI 0.799–1.106; OR 0.939, 95% CI 0.794–1.109; p = 0.456). There was not enough outcome data in the male cohort.

*Stroke*

Cerebral infarction occurred in 544 of 12,813 CRP ≤1.0 males (4.2%) versus 620 of 12,701 CRP 3.0–10.0 males (4.9%) (RD −0.6%, 95% CI −1.1 to −0.1; RR 0.870, 95% CI 0.777–0.973; OR 0.864, 95% CI 0.768–0.972; p = 0.015). In females, 458 of 11,356 CRP ≤1.0 patients (4.0%) versus 556 of 11,265 CRP 3.0–10.0 patients (4.9%) developed cerebral infarction (RD −0.9%, 95% CI −1.4 to −0.4; RR 0.817, 95% CI 0.724–0.922; OR 0.809, 95% CI 0.713–0.919; p = 0.001).

**Autoimmune thyroiditis**

After 1:1 propensity score matching, the male cohorts consisted of 2,895 patients with autoimmune thyroiditis and CRP ≤1.0 mg/L and 2,895 matched patients with CRP 3.0–10.0 mg/L; the female cohorts comprised 17,757 patients with CRP ≤1.0 mg/L and 17,757 matched patients with CRP 3.0–10.0 mg/L. Cohort characteristics were well balanced after matching (standardized differences: males—current age 0.006, age at index 0.005, immune suppressants 0.006; females—current age 0.007, age at index 0.005, immune suppressants 0.012). After matching, the mean current age of male patients and matched controls was 67.9 years (SD 9.6 vs 9.5), and mean age at index was 62.9 (SD 10.1) vs 63.0 (SD 10.0). For females, mean current age was 65.4 (SD 9.4) vs 65.5 (SD 9.4), and mean age at index was 60.0 (SD 10.0) vs 60.1 (SD 9.9).

*Parkinson’s Disease*

In the male cohort, 28 of 2,836 CRP ≤1.0 patients (1.0%) and 29 of 2,839 CRP 3.0–10.0 patients (1.0%) developed Parkinson’s disease, yielding RD −0.0% (95% CI −0.6 to 0.5), RR 0.967 (95% CI 0.577–1.620), and OR 0.966 (95% CI 0.573–1.628); p = 0.897. In the female cohort, 61 of 17,626 CRP ≤1.0 patients (0.3%) versus 56 of 17,627 CRP 3.0–10.0 patients (0.3%) were diagnosed with Parkinson’s disease (RD 0.0%, 95% CI −0.1 to 0.1; RR 1.089, 95% CI 0.758–1.565; OR 1.090, 95% CI 0.758–1.567; p = 0.643).

*Alzheimer’s Disease*

Among males, Alzheimer’s disease occurred in 17 of 2,856 CRP ≤1.0 patients (0.6%) compared with 13 of 2,870 CRP 3.0–10.0 patients (0.5%) (RD 0.1%, 95% CI −0.2 to 0.5; RR 1.314, 95% CI 0.639–2.700; OR 1.316, 95% CI 0.638–2.714; p = 0.456). In females, 64 of 17,670 CRP ≤1.0 patients (0.4%) versus 57 of 17,684 CRP 3.0–10.0 patients (0.3%) developed Alzheimer’s disease (RD 0.0%, 95% CI −0.1 to 0.2; RR 1.124, 95% CI 0.787–1.605; OR 1.124, 95% CI 0.786–1.607; p = 0.521).

*Transient Ischemic Attack (TIA)*

TIA was observed in 49 of 2,753 CRP ≤1.0 males (1.8%) compared with 51 of 2,752 CRP 3.0–10.0 males (1.9%) (RD −0.1%, 95% CI −0.8 to 0.6; RR 0.960, 95% CI 0.651–1.416; OR 0.960, 95% CI 0.646–1.426; p = 0.839). Among females, 233 of 17,052 CRP ≤1.0 patients (1.4%) versus 243 of 17,060 CRP 3.0–10.0 patients (1.4%) experienced TIA (RD −0.1%, 95% CI −0.3 to 0.2; RR 0.959, 95% CI 0.803–1.147; OR 0.959, 95% CI 0.800–1.149; p = 0.648).

*Stroke*

Cerebral infarction occurred in 66 of 2,638 CRP ≤1.0 males (2.5%) versus 74 of 2,627 CRP 3.0–10.0 males (2.8%) (RD −0.3%, 95% CI −1.2 to 0.6; RR 0.888, 95% CI 0.640–1.232; OR 0.885, 95% CI 0.632–1.239; p = 0.477). In females, 287 of 16,940 CRP ≤1.0 patients (1.7%) versus 339 of 16,889 CRP 3.0–10.0 patients (2.0%) developed cerebral infarction (RD −0.3%, 95% CI −0.6 to −0.0; RR 0.844, 95% CI 0.722–0.986; OR 0.841, 95% CI 0.718–0.986; p = 0.033).

**Lupus erythematosus (CRP-stratified)**

After 1:1 propensity score matching, the female cohorts consisted of 6,390 patients with lupus erythematosus and CRP ≤ 1.00 mg/L (“No CRP”) and 6,390 matched patients with CRP 3.00–10.00 mg/L (“CRP”). The characteristics were well balanced after matching (standardized differences: current age 0.007, age at index 0.003, immune suppressants 0.002). After matching, mean current age was 64.7 years (SD 9.3) in the No-CRP cohort and 64.6 years (SD 9.1) in the CRP cohort; mean age at index was 58.1 years (SD 9.9) vs 58.1 years (SD 9.7). Immune suppressants were recorded in 1,920/6,390 (30.0%) vs 1,927/6,390 (30.2%).

*Parkinson’s disease*

In the female cohort, 25/6,358 (0.4%) No-CRP patients versus 17/6,364 (0.3%) CRP patients developed Parkinson’s disease, yielding RD 0.1% (95% CI −0.1 to 0.3), RR 1.472 (95% CI 0.796–2.723), and OR 1.474 (95% CI 0.795–2.732); p = 0.215. There was not enough outcome data in the male cohort.

*Alzheimer’s disease*

Among females, Alzheimer’s disease occurred in 20/6,369 (0.3%) No-CRP patients compared with 22/6,378 (0.3%) CRP patients (RD −0.0%, 95% CI −0.2 to 0.2; RR 0.910, 95% CI 0.497–1.666; OR 0.910, 95% CI 0.496–1.669; p = 0.761). There was not enough outcome data in the male cohort.

*Transient ischemic attack (TIA)*

In females, TIA was observed in 112/6,077 (1.8%) No-CRP patients versus 110/6,071 (1.8%) CRP patients (RD 0.0%, 95% CI −0.4 to 0.5; RR 1.017, 95% CI 0.784–1.320; OR 1.017, 95% CI 0.780–1.327; p = 0.898). In males, 10/1,015 (1.0%) No-CRP patients versus 16/1,022 (1.6%) CRP patients developed TIA (RD −0.6%, 95% CI −1.6 to 0.4; RR 0.63, 95% CI 0.29–1.38; OR 0.63, 95% CI 0.28–1.38; p = 0.243).

*Stroke*

Among females, I63 occurred in 141/5,992 (2.4%) No-CRP patients compared with 180/5,957 (3.0%) CRP patients (RD −0.7%, 95% CI −1.2 to −0.1; RR 0.779, 95% CI 0.627–0.968; OR 0.773, 95% CI 0.619–0.967; p = 0.024). In males, 29/999 (2.9%) No-CRP patients versus 18/979 (1.8%) CRP patients developed I63 (RD 1.1%, 95% CI −0.3 to 2.4; RR 1.58, 95% CI 0.88–2.82; OR 1.60, 95% CI 0.88–2.89; p = 0.120).

**Ulcerative Colitis**

After 1:1 propensity score matching, the male cohorts consisted of 10,771 patients with ulcerative colitis and CRP ≤1.0 mg/L and 10,771 matched patients with ulcerative colitis and CRP 3.0–10.0 mg/L; the female cohorts comprised 12,608 patients with CRP ≤1.0 mg/L and 12,608 matched patients with CRP 3.0–10.0 mg/L. Cohort characteristics were well balanced after matching (standardized differences < 0.01). In males, mean current age was 65.7 years (SD 9.6) in both groups, and mean age at index was 59.2 (SD 10.5) in both groups; in females, mean current age was 65.9 (SD 9.7) in both groups, and mean age at index was 59.6 (SD 10.6) vs 59.5 (SD 10.6) (low vs higher CRP).

*Parkinson’s Disease*

In the male cohort, 46 of 10,654 CRP ≤1.0 patients (0.4%) and 51 of 10,671 CRP 3.0–10.0 patients (0.5%) developed Parkinson’s disease, yielding a risk difference (RD) −0.0% (95% CI −0.2 to 0.1), a risk ratio (RR) 0.903 (95% CI 0.607–1.345), and an odds ratio (OR) 0.903 (95% CI 0.606–1.346); p = 0.616. In the female cohort, 49 of 12,534 CRP ≤1.0 patients (0.4%) versus 32 of 12,533 CRP 3.0–10.0 patients (0.3%) were diagnosed with Parkinson’s disease (RD 0.1%, 95% CI −0.0 to 0.3; RR 1.531, 95% CI 0.981–2.389; OR 1.533, 95% CI 0.981–2.395; p = 0.059).

*Alzheimer’s Disease*

Among males, Alzheimer’s disease occurred in 32 of 10,742 CRP ≤1.0 patients (0.3%) compared with 22 of 10,744 CRP 3.0–10.0 patients (0.2%) (RD 0.1%, 95% CI −0.0 to 0.2; RR 1.455, 95% CI 0.846–2.502; OR 1.456, 95% CI 0.846–2.508; p = 0.173). In females, 47 of 12,553 CRP ≤1.0 patients (0.4%) versus 45 of 12,565 CRP 3.0–10.0 patients (0.4%) developed Alzheimer’s disease (RD 0.0%, 95% CI −0.1 to 0.2; RR 1.045, 95% CI 0.695–1.572; OR 1.046, 95% CI 0.694–1.575; p = 0.831).

*Transient Ischemic Attack (TIA)*

TIA was observed in 110 of 10,527 CRP ≤1.0 males (1.0%) compared with 117 of 10,504 CRP 3.0–10.0 males (1.1%) (RD −0.1%, 95% CI −0.3 to 0.2; RR 0.938, 95% CI 0.724–1.215; OR 0.937, 95% CI 0.722–1.218; p = 0.629). Among females, 137 of 12,231 CRP ≤1.0 patients (1.1%) versus 157 of 12,238 CRP 3.0–10.0 patients (1.3%) experienced TIA (RD −0.2%, 95% CI −0.4 to 0.1; RR 0.873, 95% CI 0.695–1.096; OR 0.872, 95% CI 0.692–1.098; p = 0.243).

*Stroke*

Cerebral infarction occurred in 142 of 10,397 CRP ≤1.0 males (1.4%) versus 200 of 10,317 CRP 3.0–10.0 males (1.9%), yielding an RD −0.6% (95% CI −0.9 to −0.2), RR 0.705 (95% CI 0.569–0.872), and OR 0.700 (95% CI 0.564–0.870); p = 0.001. In females, 159 of 12,184 CRP ≤1.0 patients (1.3%) versus 206 of 12,087 CRP 3.0–10.0 patients (1.7%) developed cerebral infarction (RD −0.4%, 95% CI −0.7 to −0.1; RR 0.766, 95% CI 0.624–0.940; OR 0.763, 95% CI 0.619–0.939; p = 0.011).

**Crohn’s Disease**

After 1:1 propensity score matching, the male cohorts consisted of 9,598 patients with Crohn’s disease and CRP ≤1.0 mg/L and 9,598 matched patients with Crohn’s disease and CRP 3.0–10.0 mg/L; the female cohorts comprised 13,034 patients with CRP ≤1.0 mg/L and 13,034 matched patients with CRP 3.0–10.0 mg/L. After matching, cohort characteristics were well balanced (e.g., standardized differences: males—current age 0.008, age at index 0.009, immune suppressants 0.007; females—current age 0.003, age at index 0.003, immune suppressants 0.001). In males, mean current age was 64.9 years (SD 9.5) in the CRP ≤1.0 group vs 64.8 years (SD 9.4) in the CRP 3.0–10.0 group; mean age at index was 57.7 (SD 10.3) vs 57.6 (SD 10.3). In females, mean current age was 64.9 (SD 9.5) in both groups, and mean age at index was 57.7 (SD 10.3) in both groups.

*Parkinson’s Disease*

In the male cohort, 34 of 9,528 CRP ≤1.0 patients (0.4%) and 37 of 9,535 CRP 3.0–10.0 patients (0.4%) developed Parkinson’s disease, yielding a risk difference (RD) −0.0% (95% CI −0.2 to 0.1), a risk ratio (RR) 0.920 (95% CI 0.578–1.464), and an odds ratio (OR) 0.919 (95% CI 0.577–1.466); p = 0.724. In the female cohort, 32 of 12,976 CRP ≤1.0 patients (0.2%) versus 24 of 12,971 CRP 3.0–10.0 patients (0.2%) were diagnosed with Parkinson’s disease (RD 0.1%, 95% CI −0.1 to 0.2; RR 1.333, 95% CI 0.786–2.261; OR 1.334, 95% CI 0.785–2.265; p = 0.285).

*Alzheimer’s Disease*

Among males, Alzheimer’s disease occurred in 18 of 9,585 CRP ≤1.0 patients (0.2%) compared with 14 of 9,577 CRP 3.0–10.0 patients (0.1%) (RD 0.0%, 95% CI −0.1 to 0.2; RR 1.285, 95% CI 0.639–2.581; OR 1.285, 95% CI 0.639–2.585; p = 0.481). In females, 38 of 12,997 CRP ≤1.0 patients (0.3%) versus 34 of 13,007 CRP 3.0–10.0 patients (0.3%) developed Alzheimer’s disease (RD 0.0%, 95% CI −0.1 to 0.2; RR 1.119, 95% CI 0.705–1.775; OR 1.119, 95% CI 0.704–1.778; p = 0.635).

*Transient Ischemic Attack (TIA)*

TIA was observed in 90 of 9,378 CRP ≤1.0 males (1.0%) compared with 97 of 9,393 CRP 3.0–10.0 males (1.0%) (RD −0.1%, 95% CI −0.4 to 0.2; RR 0.929, 95% CI 0.699–1.236; OR 0.929, 95% CI 0.696–1.239; p = 0.615). Among females, 134 of 12,693 CRP ≤1.0 patients (1.1%) versus 131 of 12,726 CRP 3.0–10.0 patients (1.0%) experienced TIA (RD 0.0%, 95% CI −0.2 to 0.3; RR 1.026, 95% CI 0.807–1.303; OR 1.026, 95% CI 0.805–1.307; p = 0.836).

*Stroke*

Cerebral infarction occurred in 116 of 9,330 CRP ≤1.0 males (1.2%) versus 160 of 9,298 CRP 3.0–10.0 males (1.7%), yielding an RD −0.5% (95% CI −0.8 to −0.1), RR 0.723 (95% CI 0.570–0.916), and OR 0.719 (95% CI 0.565–0.915); p = 0.007. In females, 154 of 12,649 CRP ≤1.0 patients (1.2%) versus 161 of 12,634 CRP 3.0–10.0 patients (1.3%) developed cerebral infarction (RD −0.1%, 95% CI −0.3 to 0.2; RR 0.955, 95% CI 0.767–1.190; OR 0.955, 95% CI 0.765–1.193; p = 0.684).

**EXPERIMENT 3**

**Chronic Inflammatory Demyelinating Polyneuropathy (CIDP)**

After 1:1 propensity score matching, the female cohorts consisted of 93 CIDP patients without cortisone and 93 matched CIDP patients treated with cortisone; the male cohorts comprised 93 CIDP patients treated with cortisone and 93 matched CIDP patients without cortisone. The characteristics of all cohorts were well balanced on age after matching (standardized differences < 0.01). After matching, the mean current age of the female no-cortisone and cortisone cohorts was 69.1 years (SD 9.9) and 69.0 years (SD 9.9), respectively; for males, the mean current age of both the cortisone and no-cortisone cohorts was 72.3 years (SD 8.4).

*Parkinson’s Disease*In the female cohort, 10 of 92 patients without cortisone (10.9 %) versus 0 of 90 patients with cortisone (0.0 %) developed Parkinson’s disease (RD 10.9 %, 95 % CI 4.5–17.2; p = 0.001). In the male cohort, 0 % vs. 0 %.

*Alzheimer’s Disease*In the female cohort, 10 of 93 patients without cortisone (10.8 %) and 10 of 93 patients with cortisone (10.8 %) were diagnosed with Alzheimer’s disease (RD 0.0 %, 95 % CI –8.9–8.9; p = 1.000). In the male cohort, 10 of 93 patients without cortisone (10.8 %) versus 0 of 93 patients with cortisone (0.0 %) developed Alzheimer’s disease (RD –10.8 %, 95 % CI –17.0––4.5; p = 0.001).

*Transient Ischemic Attack (TIA)*In the female cohort, 10 of 91 no-cortisone patients (11.0 %) versus 10 of 84 cortisone patients (11.9 %) experienced TIA (RD –0.9 %, 95 % CI –10.4–8.5; p = 0.849). In the male cohort, 10 of 91 no-cortisone patients (11.0 %) and 10 of 86 cortisone patients (11.6 %) experienced TIA (RD –0.6 %, 95 % CI –8.7–10.0; p = 0.893).

*Stroke*
In the female cohort, 10 of 89 no-cortisone patients (11.2 %) and 10 of 86 cortisone patients (11.6 %) suffered stroke (RD –0.4 %, 95 % CI –9.8–9.0; p = 0.935). In the male cohort, 10 of 89 no-cortisone patients (11.2 %) versus 10 of 85 cortisone patients (11.8 %) suffered stroke (RD –0.5 %, 95 % CI –9.0–10.0; p = 0.913).

Across all analyses, exclusion of patients with the outcome prior to the time window ranged from 0 to 10 per cohort. Together, these findings indicate that cortisone treatment in CIDP patients was associated with a significant reduction in Parkinson’s disease incidence in females and in Alzheimer’s disease incidence in males, but had no significant effect on TIA or stroke in either sex.

**Guillain–Barré Syndrome (GBS)**

After 1:1 propensity score matching, the female cohorts consisted of 113 GBS patients without cortisone and 113 matched GBS patients treated with cortisone; the male cohorts comprised 135 GBS patients treated with cortisone and 135 matched GBS patients without cortisone. The characteristics of all cohorts were well balanced on age after matching (standardized differences < 0.01). After matching, the mean current age of both the female no-cortisone and cortisone cohorts was 71.8 years (SD 9.5); for males, the mean current age of both the cortisone and no-cortisone cohorts was 70.2 years (SD 9.6).

*Parkinson’s Disease*In the female cohort, 10 of 113 patients without cortisone (8.8 %) versus 10 of 112 patients with cortisone (8.9 %) developed Parkinson’s disease (RD 0.1 %, 95 % CI –7.4–7.5; p = 0.983). In the male cohort, 10 of 134 patients without cortisone (7.5 %) versus 10 of 132 patients with cortisone (7.6 %) developed Parkinson’s disease (RD –0.1 %, 95 % CI –6.5–6.2; p = 0.972).

*Alzheimer’s Disease*In the female cohort, 0 of 113 patients without cortisone (0.0 %) versus 10 of 113 patients with cortisone (8.8 %) were diagnosed with Alzheimer’s disease (RD 8.8 %, 95 % CI 3.6–14.1; p = 0.001). In the male cohort, 0 of 134 patients without cortisone (0.0 %) versus 10 of 135 patients with cortisone (7.4 %) developed Alzheimer’s disease (RD –7.4 %, 95 % CI –11.8–3.0; p = 0.001).

*Transient Ischemic Attack (TIA)*
In the female cohort, 10 of 110 patients without cortisone (9.1 %) versus 10 of 105 patients with cortisone (9.5 %) experienced TIA (RD 0.4 %, 95 % CI –7.3–8.2; p = 0.913). In the male cohort, 10 of 133 patients without cortisone (7.5 %) versus 10 of 125 patients with cortisone (8.0 %) experienced TIA (RD –0.5 %, 95 % CI –7.0–6.1; p = 0.885).

*Stroke*
In the female cohort, 10 of 107 patients without cortisone (9.3 %) and 10 of 91 patients with cortisone (11.0 %) suffered stroke (RD 1.6 %, 95 % CI –6.8–10.1; p = 0.702). In the male cohort, 10 of 130 patients without cortisone (7.7 %) versus 10 of 124 patients with cortisone (8.1 %) suffered stroke (RD –0.4 %, 95 % CI –7.0–6.3; p = 0.912).

**Myasthenia Gravis**

After 1:1 propensity score matching, the female cohorts consisted of 225 Myasthenia Gravis patients treated with cortisone and 225 matched Myasthenia Gravis patients without cortisone; the male cohorts comprised 199 Myasthenia Gravis patients without cortisone and 199 matched Myasthenia Gravis patients treated with cortisone. The characteristics of all cohorts were well balanced on age after matching (standardized differences < 0.01). After matching, the mean current age of the female cortisone and no-cortisone cohorts was 70.6 years (SD 9.3) and 70.6 years (SD 9.3), respectively; for males, the mean current age of both cohorts was 72.3 years (SD 9.0).

*Parkinson’s Disease*In the female cohort, 10 of 223 patients treated with cortisone (4.5 %) versus 10 of 223 patients without cortisone (4.5 %) developed Parkinson’s disease (RD 0.0 %, 95 % CI –3.8–3.8; p = 1.000). In the male cohort, 10 of 197 patients without cortisone (5.1 %) versus 10 of 195 patients with cortisone (5.1 %) developed Parkinson’s disease (RD –0.1 %, 95 % CI –4.4–4.3; p = 0.981).

*Alzheimer’s Disease*In the female cohort, 10 of 223 patients without cortisone (4.5 %) versus 10 of 222 patients treated with cortisone (4.5 %) were diagnosed with Alzheimer’s disease (RD 0.0 %, 95 % CI –3.8–3.9; p = 0.992). In the male cohort, 10 of 197 patients without cortisone (5.1 %) versus 10 of 199 patients with cortisone (5.0 %) developed Alzheimer’s disease (RD 0.1 %, 95 % CI –4.3–4.4; p = 0.982).

*Transient Ischemic Attack (TIA)*In the female cohort, 10 of 219 patients without cortisone (4.6 %) versus 10 of 202 patients with cortisone (5.0 %) experienced TIA (RD –0.4 %, 95 % CI –3.7–4.5; p = 0.853). In the male cohort, 10 of 187 patients without cortisone (5.3 %) versus 10 of 179 patients with cortisone (5.6 %) experienced TIA (RD –0.2 %, 95 % CI –4.9–4.4; p = 0.920).

*Stroke*
In the female cohort, 10 of 216 patients without cortisone (4.6 %) versus 13 of 212 patients with cortisone (6.1 %) suffered stroke (RD –1.5 %, 95 % CI –5.8–2.8; p = 0.491). In the male cohort, 10 of 186 patients without cortisone (5.4 %) versus 10 of 179 patients with cortisone (5.6 %) suffered stroke (RD –0.2 %, 95 % CI –4.9–4.5; p = 0.930).

**Multiple Sclerosis**

After 1:1 propensity score matching, the female cohorts consisted of 665 MS patients without cortisone and 665 matched MS patients treated with cortisone; the male cohorts comprised 665 MS patients treated with cortisone and 665 matched MS patients without cortisone. Cohort characteristics were well balanced on age after matching (standardized differences < 0.01). After matching, the mean current age of both the female no-cortisone and cortisone cohorts was 64.3 years (SD 9.1); for males, the mean current age of both the cortisone and no-cortisone cohorts was 64.3 years (SD 9.1).

*Parkinson’s Disease*
In the female cohort, 10 of 658 patients without cortisone (1.5 %) versus 10 of 659 patients with cortisone (1.5 %) developed Parkinson’s disease (RD 0.0 %, 95 % CI –1.3–1.3; p = 0.997).
In the male cohort, 10 of 658 no-cortisone patients (1.5 %) versus 10 of 659 cortisone patients (1.5 %) developed Parkinson’s disease (RD 0.0 %, 95 % CI –1.3–1.3; p = 0.997).

*Alzheimer’s Disease*In the female cohort, 10 of 662 patients without cortisone (1.5 %) and 10 of 665 with cortisone (1.5 %) were diagnosed with Alzheimer’s disease (RD 0.0 %, 95 % CI –1.3–1.3; p = 0.992).
In the male cohort, 10 of 662 no-cortisone patients (1.5 %) versus 10 of 665 cortisone patients (1.5 %) developed Alzheimer’s disease (RD 0.0 %, 95 % CI –1.3–1.3; p = 0.992).

*Transient Ischemic Attack (TIA)*In the female cohort, 13 of 655 no-cortisone patients (2.0 %) versus 11 of 638 cortisone patients (1.7 %) experienced TIA (RD 0.3 %, 95 % CI –1.2–1.7; p = 0.729).
In the male cohort, 10 of 655 no-cortisone patients (1.5 %) and 10 of 638 cortisone patients (1.6 %) experienced TIA (RD –0.1 %, 95 % CI –1.2–1.0; p = 0.893).

*Stroke*
In the female cohort, 20 of 639 no-cortisone patients (3.1 %) and 19 of 611 cortisone patients (3.1 %) suffered stroke (RD 0.0 %, 95 % CI –1.9–1.9; p = 0.984).
In the male cohort, 20 of 639 no-cortisone patients (3.1 %) versus 19 of 611 cortisone patients (3.1 %) suffered stroke (RD 0.0 %, 95 % CI –1.8–2.0; p = 0.913).

**Vitiligo**

After 1:1 propensity score matching, the female cohorts consisted of 266 Vitiligo patients treated with cortisone and 266 matched Vitiligo patients without cortisone; the male cohorts comprised 140 Vitiligo patients treated with cortisone and 140 matched Vitiligo patients without cortisone. The characteristics of all cohorts were well balanced on age after matching (standardized differences < 0.01). After matching, the mean current age of both female cohorts was 68.5 years (SD 9.4); for males, the mean current age of both cohorts was 69.6 years (SD 8.7).

*Parkinson’s Disease*In the female cohort, 10 of 261 patients treated with cortisone (3.8 %) versus 10 of 266 patients without cortisone (3.8 %) developed Parkinson’s disease (RD 0.001, 95 % CI –0.032–0.033; p = 0.965). In the male cohort, 0 of 140 patients treated with cortisone (0.0 %) versus 10 of 138 patients without cortisone (7.2 %) developed Parkinson’s disease (RD 0.072, 95 % CI 0.029–0.116; p = 0.001).

*Alzheimer’s Disease*In the female cohort, 10 of 266 patients treated with cortisone (3.8 %) and 10 of 264 patients without cortisone (3.8 %) were diagnosed with Alzheimer’s disease (RD –0.000, 95 % CI –0.033–0.032; p = 1.000). In the male cohort, no patients in either the cortisone or no-cortisone group developed Alzheimer’s disease (0 % vs. 0 %).

*Transient Ischemic Attack (TIA)*In the female cohort, 10 of 237 patients treated with cortisone (4.2 %) versus 10 of 261 patients without cortisone (3.8 %) experienced TIA (RD 0.004, 95 % CI –0.031–0.038; p = 0.826). In the male cohort, 10 of 130 patients treated with cortisone (7.7 %) and 10 of 136 patients without cortisone (7.4 %) experienced TIA (RD –0.003, 95 % CI –0.067–0.060; p = 0.916).

*Stroke*
In the female cohort, 10 of 257 patients treated with cortisone (3.9 %) and 11 of 258 patients without cortisone (4.3 %) suffered stroke (RD –0.004, 95 % CI –0.038–0.030; p = 0.831). In the male cohort, 10 of 134 patients treated with cortisone (7.5 %) versus 10 of 136 patients without cortisone (7.4 %) suffered stroke (RD –0.001, 95 % CI –0.064–0.061; p = 0.973).

**Rheumatoid Vasculitis**

After 1:1 propensity score matching, the female cohorts consisted of 85 Rheumatoid Vasculitis patients treated with cortisone and 85 matched Rheumatoid Vasculitis patients without cortisone; the male cohorts comprised 25 Rheumatoid Vasculitis patients without cortisone and 25 matched Rheumatoid Vasculitis patients treated with cortisone. The characteristics of all cohorts were well balanced on age after matching (standardized differences < 0.01). After matching, the mean current age of the female cortisone and no-cortisone cohorts was 68.8 years (SD 9.3); for males, the mean current age of the no-cortisone and cortisone cohorts was 70.5 years (SD 9.7) and 70.5 years (SD 9.6), respectively.

*Parkinson’s Disease*In the female cohort, no patients in either the cortisone or no-cortisone group developed Parkinson’s disease (0.0 % vs. 0.0 %). In the male cohort, 10 of 23 patients without cortisone (43.5 %) versus 0 of 24 patients with cortisone (0.0 %) developed Parkinson’s disease (RD 43.5 %, 95 % CI 23.2–63.7; p < 0.001).

*Alzheimer’s Disease*In the female cohort, 10 of 85 patients treated with cortisone (11.8 %) versus 0 of 85 patients without cortisone (0.0 %) were diagnosed with Alzheimer’s disease (RD 11.8 %, 95 % CI 4.9–18.6; p = 0.001). In the male cohort, no patients in either group developed Alzheimer’s disease (0.0 % vs. 0.0 %).

*Transient Ischemic Attack (TIA)*In the female cohort, 10 of 82 patients without cortisone (12.2 %) versus 10 of 81 patients with cortisone (12.3 %) experienced TIA (RD –0.1 %, 95 % CI –9.9–10.2; p = 0.977). In the male cohort, no patients in either group experienced TIA (0.0 % vs. 0.0 %).

*Stroke*
In the female cohort, 10 of 79 patients without cortisone (12.7 %) and 10 of 77 patients with cortisone (13.0 %) suffered stroke (RD –0.3 %, 95 % CI –10.2–10.8; p = 0.951). In the male cohort, 10 of 24 patients without cortisone (41.7 %) versus 0 of 24 patients with cortisone (0.0 %) suffered stroke (RD 41.7 %, 95 % CI 21.9–61.4; p < 0.001).

**Graves’ Disease**

After 1:1 propensity score matching, the female cohorts consisted of 2 647 Graves’ Disease patients without cortisone and 2 647 matched Graves’ Disease patients treated with cortisone; the male cohorts comprised 970 Graves’ Disease patients treated with cortisone and 970 matched Graves’ Disease patients without cortisone. Cohort characteristics were well balanced on age after matching (standardized differences < 0.01). After matching, the mean current age of the female no-cortisone and cortisone cohorts was 69.5 years (SD 9.3); for males, the mean current age of both the cortisone and no-cortisone cohorts was 69.8 years (SD 9.1).

*Parkinson’s Disease*In the female cohort, 20 of 2 630 patients without cortisone (0.8 %) versus 29 of 2 610 patients with cortisone (1.1 %) developed Parkinson’s disease (RD 0.4 %, 95 % CI –0.2–0.9; p = 0.187). In the male cohort, 10 of 965 patients without cortisone (1.0 %) versus 18 of 946 patients with cortisone (1.9 %) developed Parkinson’s disease (RD 0.9 %, 95 % CI –0.2–1.9; p = 0.115).

*Alzheimer’s Disease*
In the female cohort, 28 of 2 633 patients without cortisone (1.1 %) versus 16 of 2 633 patients with cortisone (0.6 %) were diagnosed with Alzheimer’s disease (RD –0.5 %, 95 % CI –0.9–0.0; p = 0.069). In the male cohort, 10 of 963 patients without cortisone (1.0 %) versus 10 of 960 patients with cortisone (1.0 %) developed Alzheimer’s disease (RD 0.0 %, 95 % CI –0.9–0.9; p = 0.994).

*Transient Ischemic Attack (TIA)*In the female cohort, 62 of 2 606 patients without cortisone (2.4 %) versus 90 of 2 422 patients with cortisone (3.7 %) experienced TIA (RD 1.3 %, 95 % CI 0.4–2.3; p = 0.006). In the male cohort, 15 of 945 patients without cortisone (1.6 %) versus 29 of 900 patients with cortisone (3.2 %) experienced TIA (RD 1.6 %, 95 % CI 0.2–3.0; p = 0.021).

*Stroke*
In the female cohort, 93 of 2 568 patients without cortisone (3.6 %) versus 115 of 2 440 patients with cortisone (4.7 %) suffered stroke (RD 1.1 %, 95 % CI –0.0–2.2; p = 0.053). In the male cohort, 39 of 927 patients without cortisone (4.2 %) versus 35 of 866 patients with cortisone (4.0 %) suffered stroke (RD –0.2 %, 95 % CI –2.0–1.7; p = 0.860).

**Vasculitis**

After 1:1 propensity score matching, the female cohorts consisted of 1 344 Vasculitis patients treated with cortisone and 1 344 matched Vasculitis patients without cortisone; the male cohorts comprised 484 Vasculitis patients without cortisone and 484 matched Vasculitis patients treated with cortisone. The characteristics of all cohorts were well balanced on age after matching (standardized differences < 0.01). After matching, the mean current age of the female cortisone and no-cortisone cohorts was 71.8 years (SD 9.3); for males, the mean current age of both the no-cortisone and cortisone cohorts was 72.0 years (SD 9.0).

*Parkinson’s Disease*
In the female cohort, 13 of 1 332 patients treated with cortisone (1.0 %) versus 14 of 1 341 patients without cortisone (1.0 %) developed Parkinson’s disease (RD –0.1 %, 95 % CI –0.8–0.7; p = 0.860). In the male cohort, 12 of 482 patients without cortisone (2.5 %) versus 10 of 473 patients with cortisone (2.1 %) developed Parkinson’s disease (RD 0.4 %, 95 % CI –1.5–2.3; p = 0.699).

*Alzheimer’s Disease*In the female cohort, 23 of 1 342 patients treated with cortisone (1.7 %) versus 20 of 1 339 patients without cortisone (1.5 %) were diagnosed with Alzheimer’s disease (RD 0.2 %, 95 % CI –0.7–1.2; p = 0.650). In the male cohort, 10 of 484 patients without cortisone (2.1 %) versus 10 of 482 patients with cortisone (2.1 %) developed Alzheimer’s disease (RD 0.0 %, 95 % CI –1.8–1.8; p = 0.993).

*Transient Ischemic Attack (TIA)*In the female cohort, 64 of 1 275 patients treated with cortisone (5.0 %) versus 53 of 1 300 patients without cortisone (4.1 %) experienced TIA (RD 0.9 %, 95 % CI –0.7–2.6; p = 0.251). In the male cohort, 14 of 472 patients without cortisone (3.0 %) versus 18 of 459 patients with cortisone (3.9 %) experienced TIA (RD –0.9 %, 95 % CI –3.3–1.4; p = 0.424).

*Stroke*
In the female cohort, 84 of 1 282 patients treated with cortisone (6.6 %) versus 75 of 1 302 patients without cortisone (5.8 %) suffered stroke (RD 0.8 %, 95 % CI –1.1–2.6; p = 0.402). In the male cohort, 23 of 472 patients without cortisone (4.9 %) versus 33 of 459 patients with cortisone (7.2 %) suffered stroke (RD –2.3 %, 95 % CI –5.4–0.7; p = 0.137).

**Systemic Sclerosis (Scleroderma)**

After 1:1 propensity score matching, the female cohorts consisted of 293 Scleroderma patients without cortisone and 293 matched Scleroderma patients treated with cortisone; the male cohorts comprised 65 Scleroderma patients treated with cortisone and 65 matched Scleroderma patients without cortisone. The characteristics of all cohorts were well balanced on age after matching (standardized differences < 0.01). After matching, the mean current age of the female no-cortisone and cortisone cohorts was 68.9 years (SD 9.3); for males, the mean current age of the cortisone and no-cortisone cohorts was 70.7 years (SD 9.3) and 70.8 years (SD 9.4), respectively.

*Parkinson’s Disease*In the female cohort, 10 of 292 patients without cortisone (3.4 %) versus 10 of 291 patients with cortisone (3.4 %) developed Parkinson’s disease (RD –0.0 %, 95 % CI –3.0 %–2.9 %; p = 0.994). In the male cohort, 10 of 63 patients treated with cortisone (15.9 %) versus 10 of 65 patients without cortisone (15.4 %) developed Parkinson’s disease (RD 0.5 %, 95 % CI –12.1 %–13.1 %; p = 0.939).

*Alzheimer’s Disease*In the female cohort, 10 of 293 patients without cortisone (3.4 %) and 10 of 293 patients with cortisone (3.4 %) were diagnosed with Alzheimer’s disease (RD 0.0 %, 95 % CI –2.9 %–2.9 %; p = 1.000). In the male cohort, 10 of 64 patients treated with cortisone (15.6 %) versus 0 of 65 patients without cortisone (0.0 %) developed Alzheimer’s disease (RD 15.6 %, 95 % CI 6.7 %–24.5 %; p = 0.001).

*Transient Ischemic Attack (TIA)*In the female cohort, 10 of 290 no-cortisone patients (3.4 %) versus 10 of 267 cortisone patients (3.7 %) experienced TIA (RD –0.3 %, 95 % CI –3.4 %–2.8 %; p = 0.851). In the male cohort, 10 of 60 cortisone patients (16.7 %) and 10 of 65 no-cortisone patients (15.4 %) experienced TIA (RD 1.3 %, 95 % CI –11.6 %–14.2 %; p = 0.845).

*Stroke*
In the female cohort, 10 of 280 no-cortisone patients (3.6 %) and 10 of 276 cortisone patients (3.6 %) suffered stroke (RD –0.1 %, 95 % CI –3.1 %–3.0 %; p = 0.974). In the male cohort, 10 of 62 cortisone patients (16.1 %) versus 10 of 62 no-cortisone patients (16.1 %) suffered stroke (RD 0.0 %, 95 % CI –12.9 %–12.9 %; p = 1.000).

**Dermatomyositis**

After 1:1 propensity score matching, the female cohorts consisted of 253 Dermatomyositis patients without cortisone and 253 matched Dermatomyositis patients treated with cortisone; the male cohorts comprised 110 Dermatomyositis patients treated with cortisone and 110 matched Dermatomyositis patients without cortisone. The characteristics of all cohorts were well balanced on age after matching (standardized differences < 0.01). After matching, the mean current age of the female no-cortisone and cortisone cohorts was 68.8 years (SD 9.4); for males, the mean current age of both the cortisone and no-cortisone cohorts was 70.1 years (SD 9.3).

*Parkinson’s Disease*In the female cohort, 10 of 252 patients without cortisone (4.0 %) versus 10 of 251 patients with cortisone (4.0 %) developed Parkinson’s disease (RD 0.0 %, 95 % CI –3.4–3.4; p = 0.993).
In the male cohort, 10 of 109 patients without cortisone (9.2 %) versus 10 of 109 patients with cortisone (9.2 %) developed Parkinson’s disease (RD 0.0 %, 95 % CI –7.7–7.7; p = 1.000).

*Alzheimer’s Disease*In the female cohort, 10 of 253 patients without cortisone (4.0 %) and 10 of 253 patients with cortisone (4.0 %) were diagnosed with Alzheimer’s disease (RD 0.0 %, 95 % CI –3.4–3.4; p = 1.000).
In the male cohort, 10 of 109 patients without cortisone (9.2 %) versus 10 of 110 patients with cortisone (9.1 %) developed Alzheimer’s disease (RD 0.1 %, 95 % CI –7.5–7.7; p = 0.983).

*Transient Ischemic Attack (TIA)*In the female cohort, 10 of 247 no-cortisone patients (4.0 %) versus 10 of 234 cortisone patients (4.3 %) experienced TIA (RD –0.3 %, 95 % CI –3.8–3.3; p = 0.902).
In the male cohort, 10 of 108 no-cortisone patients (9.3 %) versus 10 of 104 cortisone patients (9.6 %) experienced TIA (RD –0.4 %, 95 % CI –8.2–7.5; p = 0.929).

*Stroke*
In the female cohort, 10 of 247 no-cortisone patients (4.0 %) and 13 of 244 cortisone patients (5.3 %) suffered stroke (RD –1.3 %, 95 % CI –5.0–2.5; p = 0.502).
In the male cohort, 10 of 102 no-cortisone patients (9.8 %) versus 10 of 97 cortisone patients (10.3 %) suffered stroke (RD –0.5 %, 95 % CI –8.9–7.9; p = 0.906).

**Arthropathic Psoriasis**

After 1:1 propensity score matching, the female cohorts consisted of 792 Arthropathic Psoriasis patients treated with cortisone and 792 matched Arthropathic Psoriasis patients without cortisone; the male cohorts comprised 382 Arthropathic Psoriasis patients treated with cortisone and 382 matched Arthropathic Psoriasis patients without cortisone. The characteristics of all cohorts were well balanced on age after matching (standardized differences < 0.01). After matching, the mean current age of the female cohorts was 66.7 years (SD 9.3); for males, the mean current age of both cohorts was 69.2 years (SD 9.1).

*Parkinson’s Disease*In the female cohort, 10 of 786 patients treated with cortisone (1.3 %) versus 10 of 792 patients without cortisone (1.3 %) developed Parkinson’s disease (RD 0.0 %, 95 % CI –1.1–1.1; p = 0.986). In the male cohort, 10 of 376 patients treated with cortisone (2.7 %) versus 10 of 378 patients without cortisone (2.6 %) developed Parkinson’s disease (RD 0.0 %, 95 % CI –2.3–2.3; p = 0.990).

*Alzheimer’s Disease*In the female cohort, 10 of 789 patients treated with cortisone (1.3 %) versus 10 of 792 patients without cortisone (1.3 %) were diagnosed with Alzheimer’s disease (RD 0.0 %, 95 % CI –1.1–1.1; p = 0.993). In the male cohort, 10 of 381 patients treated with cortisone (2.6 %) versus 10 of 382 patients without cortisone (2.6 %) developed Alzheimer’s disease (RD 0.0 %, 95 % CI –2.3–2.3; p = 0.995).

*Transient Ischemic Attack (TIA)*In the female cohort, 21 of 753 patients treated with cortisone (2.8 %) versus 15 of 778 patients without cortisone (1.9 %) experienced TIA (RD 0.9 %, 95 % CI –0.7–2.4; p = 0.266). In the male cohort, 10 of 360 patients treated with cortisone (2.8 %) versus 10 of 379 patients without cortisone (2.6 %) experienced TIA (RD 0.1 %, 95 % CI –2.2–2.5; p = 0.907).

*Stroke*
In the female cohort, 24 of 763 patients treated with cortisone (3.1 %) versus 17 of 762 patients without cortisone (2.2 %) suffered stroke (RD 0.9 %, 95 % CI –0.7–2.5; p = 0.270). In the male cohort, 17 of 350 patients treated with cortisone (4.9 %) versus 13 of 373 patients without cortisone (3.5 %) suffered stroke (RD 1.4 %, 95 % CI –1.5–4.3; p = 0.355).

**Psoriasis**

After 1:1 propensity score matching, the female cohorts consisted of 2 374 Psoriasis patients without cortisone and 2 374 matched Psoriasis patients treated with cortisone; the male cohorts comprised 1 564 Psoriasis patients treated with cortisone and 1 564 matched Psoriasis patients without cortisone. The characteristics of all cohorts were well balanced on age after matching (standardized differences < 0.01). After matching, the mean current age of the female no-cortisone and cortisone cohorts was 68.7 years (SD 9.6); for males, the mean current age of both the cortisone and no-cortisone cohorts was 69.5 years (SD 9.1).

*Parkinson’s Disease*In the female cohort, 13 of 2 356 patients without cortisone (0.6 %) versus 10 of 2 362 patients with cortisone (0.4 %) developed Parkinson’s disease (RD 0.1 %, 95 % CI –0.3–0.5; p = 0.527).
In the male cohort, 14 of 1 553 patients without cortisone (0.9 %) versus 15 of 1 544 patients with cortisone (1.0 %) developed Parkinson’s disease (RD –0.1 %, 95 % CI –0.7–0.6; p = 0.840).

*Alzheimer’s Disease*
In the female cohort, 13 of 2 362 patients without cortisone (0.6 %) and 17 of 2 370 patients with cortisone (0.7 %) were diagnosed with Alzheimer’s disease (RD –0.2 %, 95 % CI –0.6–0.3; p = 0.469).
In the male cohort, 10 of 1 563 patients without cortisone (0.6 %) versus 10 of 1 557 patients with cortisone (0.6 %) developed Alzheimer’s disease (RD 0.0 %, 95 % CI –0.6–0.6; p = 0.993).

*Transient Ischemic Attack (TIA)*In the female cohort, 63 of 2 261 patients without cortisone (2.8 %) versus 41 of 2 328 patients with cortisone (1.8 %) experienced TIA (RD 1.0 %, 95 % CI 0.2–1.9; p = 0.020).
In the male cohort, 22 of 1 535 patients without cortisone (1.4 %) and 35 of 1 486 patients with cortisone (2.4 %) experienced TIA (RD –0.9 %, 95 % CI –1.9–0.1; p = 0.063).

*Stroke*
In the female cohort, 81 of 2 260 patients without cortisone (3.6 %) and 65 of 2 315 patients with cortisone (2.8 %) suffered stroke (RD 0.8 %, 95 % CI –0.2–1.8; p = 0.135).
In the male cohort, 53 of 1 528 patients without cortisone (3.5 %) versus 62 of 1 449 patients with cortisone (4.3 %) suffered stroke (RD –0.8 %, 95 % CI –2.2–0.6; p = 0.252).

**Sjögren’s Syndrome**

After 1:1 propensity score matching, the female cohorts consisted of 1 615 Sjögren’s Syndrome patients without cortisone and 1 615 matched patients treated with cortisone; the male cohorts comprised 284 patients treated with cortisone and 284 matched patients without cortisone. Cohort characteristics were well balanced on age after matching (standardized differences < 0.01). After matching, the mean current age of the female no-cortisone and cortisone cohorts was 69.5 years (SD 9.0) and 69.5 years (SD 9.0), respectively; for males, the mean current age of the cortisone and no-cortisone cohorts was 72.0 years (SD 8.7) and 72.0 years (SD 8.7), respectively.

*Parkinson’s Disease*In the female cohort, 16 of 1 602 patients without cortisone (1.0 %) versus 22 of 1 596 patients with cortisone (1.4 %) developed Parkinson’s disease (RD –0.4 %, 95 % CI –1.1–0.3; p = 0.322).
In the male cohort, 10 of 275 patients treated with cortisone (3.6 %) versus 10 of 280 patients without cortisone (3.6 %) developed Parkinson’s disease (RD 0.1 %, 95 % CI –3.0–3.2; p = 0.967).

*Alzheimer’s Disease*
In the female cohort, 14 of 1 614 patients without cortisone (0.9 %) versus 15 of 1 611 patients with cortisone (0.9 %) were diagnosed with Alzheimer’s disease (RD –0.1 %, 95 % CI –0.7–0.6; p = 0.848).
In the male cohort, 10 of 282 patients treated with cortisone (3.5 %) versus 10 of 283 patients without cortisone (3.5 %) developed Alzheimer’s disease (RD 0.0 %, 95 % CI –3.0–3.1; p = 0.994).

*Transient Ischemic Attack (TIA)*
In the female cohort, 45 of 1 572 patients without cortisone (2.9 %) versus 49 of 1 410 patients with cortisone (3.5 %) experienced TIA (RD –0.6 %, 95 % CI –1.9–0.6; p = 0.339).
In the male cohort, 17 of 245 patients treated with cortisone (6.9 %) versus 10 of 271 patients without cortisone (3.7 %) experienced TIA (RD 3.2 %, 95 % CI –0.6–7.1; p = 0.098).

*Stroke*
In the female cohort, 62 of 1 577 patients without cortisone (3.9 %) versus 61 of 1 528 patients with cortisone (4.0 %) suffered stroke (RD –0.1 %, 95 % CI –1.4–1.3; p = 0.931).
In the male cohort, 12 of 260 patients treated with cortisone (4.6 %) versus 17 of 271 patients without cortisone (6.3 %) suffered stroke (RD –1.7 %, 95 % CI –5.5–2.2; p = 0.401).

**Addison’s Disease**

After 1:1 propensity score matching, the female cohorts consisted of 1 154 Addison’s Disease patients without cortisone and 1 154 matched Addison’s Disease patients treated with cortisone; the male cohorts comprised 931 Addison’s Disease patients without cortisone and 931 matched Addison’s Disease patients treated with cortisone. The characteristics of all cohorts were well balanced on age after matching (standardized differences < 0.01). After matching, the mean current age of the female no-cortisone and cortisone cohorts was 68.3 years (SD 9.7); for males, the mean current age of the no-cortisone and cortisone cohorts was 69.5 years (SD 9.3) and 69.6 years (SD 9.2), respectively.

*Parkinson’s Disease*
In the female cohort, 10 of 1 147 patients without cortisone (0.9 %) versus 17 of 1 142 patients with cortisone (1.5 %) developed Parkinson’s disease (RD 0.6 %, 95 % CI –0.3–1.5; p = 0.172). In the male cohort, 10 of 914 patients without cortisone (1.1 %) versus 11 of 897 patients with cortisone (1.2 %) developed Parkinson’s disease (RD –0.1 %, 95 % CI –1.1–0.9; p = 0.793).

*Alzheimer’s Disease*In the female cohort, 10 of 1 146 patients without cortisone (0.9 %) versus 14 of 1 148 patients with cortisone (1.2 %) were diagnosed with Alzheimer’s disease (RD –0.3 %, 95 % CI –1.2–0.5; p = 0.414). In the male cohort, 10 of 924 patients without cortisone (1.1 %) versus 10 of 925 patients with cortisone (1.1 %) developed Alzheimer’s disease (RD 0.0 %, 95 % CI –0.9–0.9; p = 0.998).

*Transient Ischemic Attack (TIA)*In the female cohort, 31 of 1 105 patients without cortisone (2.8 %) versus 36 of 1 099 patients with cortisone (3.3 %) experienced TIA (RD 0.5 %, 95 % CI –1.0–1.9; p = 0.520). In the male cohort, 19 of 904 patients without cortisone (2.1 %) versus 19 of 885 patients with cortisone (2.1 %) experienced TIA (RD 0.0 %, 95 % CI –1.4–1.3; p = 0.947).

*Stroke*
In the female cohort, 51 of 1 093 patients without cortisone (4.7 %) versus 64 of 1 066 patients with cortisone (6.0 %) suffered stroke (RD 1.3 %, 95 % CI –0.6–3.2; p = 0.166). In the male cohort, 46 of 862 patients without cortisone (5.3 %) versus 38 of 846 patients with cortisone (4.5 %) suffered stroke (RD 0.8 %, 95 % CI –1.2–2.9; p = 0.420).

**Celiac Disease**

After 1:1 propensity score matching, the female cohorts consisted of 353 Celiac Disease patients without cortisone and 353 matched Celiac Disease patients treated with cortisone; the male cohorts comprised of 145 Celiac Disease patients treated with cortisone and 145 matched Celiac Disease patients without cortisone. The characteristics of all cohorts were well balanced on age after matching (standardized differences < 0.01). After matching, the mean current age of the female no-cortisone and cortisone cohorts was 66.9 years (SD 9.5) and 66.9 years (SD 9.5), respectively; for males, the mean current age of both the cortisone and no-cortisone cohorts was 69.1 years (SD 9.5).

*Parkinson’s Disease*In the female cohort, 10 of 352 patients without cortisone (2.8 %) versus 10 of 350 patients with cortisone (2.9 %) developed Parkinson’s disease (RD –0.0 %, 95 % CI –2.5–2.4; p = 0.990). In the male cohort, 10 of 143 patients with cortisone (7.0 %) versus 10 of 145 patients without cortisone (6.9 %) developed Parkinson’s disease (RD 0.1 %, 95 % CI –5.8–6.0; p = 0.974).

*Alzheimer’s Disease*In the female cohort, 10 of 350 patients without cortisone (2.9 %) versus 10 of 350 patients with cortisone (2.9 %) were diagnosed with Alzheimer’s disease (RD 0.0 %, 95 % CI –2.5–2.5; p = 1.000). In the male cohort, 10 of 145 patients with cortisone (6.9 %) versus 0 of 144 patients without cortisone (0.0 %) developed Alzheimer’s disease (RD 6.9 %, 95 % CI 2.8–11.0; p = 0.001).

*Transient Ischemic Attack (TIA)*In the female cohort, 10 of 351 no-cortisone patients (2.8 %) versus 10 of 342 cortisone patients (2.9 %) experienced TIA (RD –0.1 %, 95 % CI –2.6–2.4; p = 0.953). In the male cohort, 10 of 139 cortisone patients (7.2 %) and 10 of 142 no-cortisone patients (7.0 %) experienced TIA (RD 0.2 %, 95 % CI –5.9–6.2; p = 0.960).

*Stroke*
In the female cohort, 10 of 346 no-cortisone patients (2.9 %) and 13 of 333 cortisone patients (3.9 %) suffered stroke (RD –1.0 %, 95 % CI –3.7–1.7; p = 0.465). In the male cohort, 10 of 135 cortisone patients (7.4 %) versus 10 of 139 no-cortisone patients (7.2 %) suffered stroke (RD 0.2 %, 95 % CI –5.9–6.4; p = 0.946).

**Myositis**

After 1:1 propensity score matching, the female cohorts consisted of 6 258 Myositis patients treated with cortisone and 6 258 matched Myositis patients without cortisone; the male cohorts comprised 3 432 Myositis patients without cortisone and 3 432 matched Myositis patients treated with cortisone. The characteristics of all cohorts were well balanced on age after matching (standardized differences < 0.01). After matching, the mean current age of the female cortisone and no-cortisone cohorts was 69.6 years (SD 9.5); for males, the mean current age of the no-cortisone and cortisone cohorts was 69.9 years (SD 9.3).

*Parkinson’s Disease*
In the female cohort, 70 of 6 223 patients treated with cortisone (1.1 %) versus 75 of 6 227 patients without cortisone (1.2 %) developed Parkinson’s disease (RD –0.1 %, 95 % CI –0.5–0.3; p = 0.679). In the male cohort, 46 of 3 411 patients without cortisone (1.3 %) versus 59 of 3 395 patients treated with cortisone (1.7 %) developed Parkinson’s disease (RD –0.4 %, 95 % CI –1.0–0.2; p = 0.193).

*Alzheimer’s Disease*
In the female cohort, 79 of 6 244 patients treated with cortisone (1.3 %) versus 68 of 6 247 patients without cortisone (1.1 %) were diagnosed with Alzheimer’s disease (RD 0.2 %, 95 % CI –0.2–0.6; p = 0.360). In the male cohort, 31 of 3 423 patients without cortisone (0.9 %) versus 40 of 3 422 patients treated with cortisone (1.2 %) developed Alzheimer’s disease (RD –0.3 %, 95 % CI –0.7–0.2; p = 0.282).

*Transient Ischemic Attack (TIA)*
In the female cohort, 269 of 5 850 patients treated with cortisone (4.6 %) versus 241 of 6 108 patients without cortisone (3.9 %) experienced TIA (RD 0.7 %, 95 % CI –0.1–1.4; p = 0.077). In the male cohort, 116 of 3 346 patients without cortisone (3.5 %) versus 185 of 3 260 patients treated with cortisone (5.7 %) experienced TIA (RD –2.2 %, 95 % CI –3.2––1.2; p < 0.001).

*Stroke*
In the female cohort, 350 of 6 005 patients treated with cortisone (5.8 %) versus 291 of 6 122 patients without cortisone (4.8 %) suffered stroke (RD 1.1 %, 95 % CI 0.3–1.9; p = 0.008). In the male cohort, 201 of 3 338 patients without cortisone (6.0 %) versus 235 of 3 269 patients treated with cortisone (7.2 %) suffered stroke (RD –1.2 %, 95 % CI –2.4–0.0; p = 0.056).

**Rheumatoid Arthritis**

After 1:1 propensity score matching, the female cohorts consisted of 3 270 Rheumatoid Arthritis patients without cortisone and 3 270 matched Rheumatoid Arthritis patients treated with cortisone; the male cohorts comprised 1 237 Rheumatoid Arthritis patients treated with cortisone and 1 237 matched Rheumatoid Arthritis patients without cortisone. The characteristics of all cohorts were well balanced on age after matching (standardized differences < 0.01). After matching, the mean current age of the female no-cortisone and cortisone cohorts was 70.5 years (SD 9.1); for males, the mean current age of both the cortisone and no-cortisone cohorts was 72.0 years (SD 8.4).

*Parkinson’s Disease*
In the female cohort, 20 of 3 242 patients without cortisone (0.6 %) versus 21 of 3 243 patients with cortisone (0.6 %) developed Parkinson’s disease (RD –0.0 %, 95 % CI –0.4–0.4; p = 0.876). In the male cohort, 10 of 1 210 patients with cortisone (0.8 %) versus 14 of 1 225 patients without cortisone (1.1 %) developed Parkinson’s disease (RD –0.3 %, 95 % CI –1.1–0.5; p = 0.429).

*Alzheimer’s Disease*In the female cohort, 18 of 3 258 patients without cortisone (0.6 %) versus 36 of 3 248 patients with cortisone (1.1 %) were diagnosed with Alzheimer’s disease (RD –0.6 %, 95 % CI –1.0––0.1; p = 0.013). In the male cohort, 17 of 1 229 patients with cortisone (1.4 %) versus 11 of 1 225 patients without cortisone (0.9 %) developed Alzheimer’s disease (RD 0.5 %, 95 % CI –0.4–1.3; p = 0.258).

*Transient Ischemic Attack (TIA)*
In the female cohort, 63 of 3 217 no-cortisone patients (2.0 %) versus 77 of 3 065 cortisone patients (2.5 %) experienced TIA (RD –0.6 %, 95 % CI –1.3–0.2; p = 0.137). In the male cohort, 34 of 1 161 cortisone patients (2.9 %) versus 20 of 1 219 no-cortisone patients (1.6 %) experienced TIA (RD 1.3 %, 95 % CI 0.1–2.5; p = 0.035).

*Stroke*
In the female cohort, 101 of 3 173 no-cortisone patients (3.2 %) versus 135 of 3 051 cortisone patients (4.4 %) suffered stroke (RD –1.2 %, 95 % CI –2.2––0.3; p = 0.010). In the male cohort, 65 of 1 128 cortisone patients (5.8 %) versus 51 of 1 195 no-cortisone patients (4.3 %) suffered stroke (RD 1.5 %, 95 % CI –0.3–3.3; p = 0.098).

**Lupus Erythematosus**

After 1:1 propensity score matching, the female cohorts consisted of 465 Lupus erythematosus patients without cortisone and 465 matched Lupus erythematosus patients treated with cortisone; the male cohorts comprised 95 Lupus erythematosus patients without cortisone and 95 matched Lupus erythematosus patients treated with cortisone. The characteristics of all cohorts were well balanced on age after matching (standardized differences < 0.01). After matching, the mean current age of the female no-cortisone and cortisone cohorts was 66.9 years (SD 9.5); for males, the mean current age of both the no-cortisone and cortisone cohorts was 69.1 years (SD 10.1)

*Parkinson’s Disease*
In the female cohort, 10 of 463 patients without cortisone (2.2 %) versus 10 of 463 patients with cortisone (2.2 %) developed Parkinson’s disease (RD 0.0 %, 95 % CI –1.9–1.9; p = 1.000). In the male cohort, 10 of 92 patients without cortisone (10.9 %) versus 10 of 94 patients with cortisone (10.6 %) developed Parkinson’s disease (RD 0.2 %, 95 % CI –8.7–9.1; p = 0.959).

*Alzheimer’s Disease*
In the female cohort, 10 of 465 patients without cortisone (2.2 %) and 10 of 462 patients with cortisone (2.2 %) were diagnosed with Alzheimer’s disease (RD 0.0 %, 95 % CI –1.9–1.9; p = 0.988). In the male cohort, no patients in either group developed Alzheimer’s disease (0.0 % vs. 0.0 %).

*Transient Ischemic Attack (TIA)*
In the female cohort, 12 of 456 patients without cortisone (2.6 %) versus 13 of 446 patients with cortisone (2.9 %) experienced TIA (RD 0.3 %, 95 % CI –1.9–2.4; p = 0.796). In the male cohort, 10 of 92 patients without cortisone (10.9 %) and 10 of 89 patients with cortisone (11.2 %) experienced TIA (RD –0.4 %, 95 % CI –9.5–8.8; p = 0.937).

*Stroke*
In the female cohort, 19 of 441 patients without cortisone (4.3 %) and 21 of 422 patients with cortisone (5.0 %) suffered stroke (RD 0.7 %, 95 % CI –2.1–3.5; p = 0.641). In the male cohort, 10 of 90 patients without cortisone (11.1 %) versus 10 of 85 patients with cortisone (11.8 %) suffered stroke (RD –0.7 %, 95 % CI –10.1–8.8; p = 0.892).

**Autoimmune Thyreoiditis**

After 1:1 propensity score matching, the female cohorts consisted of 1 254 Autoimmune Thyreoiditis patients without cortisone and 1 254 matched Autoimmune Thyreoiditis patients treated with cortisone; the male cohorts comprised 243 Autoimmune Thyreoiditis patients treated with cortisone and 243 matched Autoimmune Thyreoiditis patients without cortisone. The characteristics of all cohorts were well balanced on age after matching (standardized differences < 0.01). After matching, the mean current age of the female no-cortisone and cortisone cohorts was 67.2 years (SD 9.6); for males, the mean current age of the cortisone and no-cortisone cohorts was 68.5 years (SD 9.2) and 68.6 years (SD 9.2), respectively.

*Parkinson’s Disease*
In the female cohort, 10 of 1 250 patients without cortisone (0.8 %) versus 10 of 1 247 patients with cortisone (0.8 %) developed Parkinson’s disease (RD 0.0 %, 95 % CI –0.7–0.7; p = 0.996). In the male cohort, 10 of 242 patients with cortisone (4.1 %) versus 10 of 242 patients without cortisone (4.1 %) developed Parkinson’s disease (RD 0.0 %, 95 % CI –3.5–3.5; p = 1.000).

*Alzheimer’s Disease*
In the female cohort, 10 of 1 252 patients without cortisone (0.8 %) and 10 of 1 248 patients with cortisone (0.8 %) were diagnosed with Alzheimer’s disease (RD 0.0 %, 95 % CI –0.7–0.7; p = 0.994). In the male cohort, 10 of 241 patients with cortisone (4.1 %) versus 10 of 242 patients without cortisone (4.1 %) developed Alzheimer’s disease (RD 0.0 %, 95 % CI –3.5–3.6; p = 0.992).

*Transient Ischemic Attack (TIA)*In the female cohort, 25 of 1 226 no-cortisone patients (2.0 %) versus 25 of 1 180 cortisone patients (2.1 %) experienced TIA (RD –0.1 %, 95 % CI –1.2–1.1; p = 0.891). In the male cohort, 10 of 234 cortisone patients (4.3 %) and 10 of 239 no-cortisone patients (4.2 %) experienced TIA (RD 0.1 %, 95 % CI –3.5–3.7; p = 0.961).

*Stroke*
In the female cohort, 23 of 1 238 no-cortisone patients (1.9 %) and 32 of 1 188 cortisone patients (2.7 %) suffered stroke (RD –0.8 %, 95 % CI –2.0–0.4; p = 0.167). In the male cohort, 10 of 226 cortisone patients (4.4 %) versus 10 of 239 no-cortisone patients (4.2 %) suffered stroke (RD 0.2 %, 95 % CI –3.5–3.9; p = 0.898).

**Diabetes Mellitus Type 1**

After 1:1 propensity score matching, the female cohorts consisted of 2 633 Diabetes mellitus Type 1 patients without cortisone and 2 633 matched patients treated with cortisone; the male cohorts comprised 2 312 patients treated with cortisone and 2 312 matched patients without cortisone. The characteristics of all cohorts were well balanced on age after matching (standardized differences < 0.01). After matching, the mean current age of the female no-cortisone and cortisone cohorts was 71.3 years (SD 9.4); for males, the mean current age of both the cortisone and no-cortisone cohorts was 70.9 years (SD 9.3).

*Parkinson’s Disease*
In the female cohort, 17 of 2 614 patients without cortisone (0.7 %) versus 27 of 2 599 patients with cortisone (1.0 %) developed Parkinson’s disease (RD 0.4 %, 95 % CI –0.1 – 0.9; p = 0.125). In the male cohort, 23 of 2 275 patients with cortisone (1.0 %) versus 25 of 2 292 patients without cortisone (1.1 %) developed Parkinson’s disease (RD –0.1 %, 95 % CI –0.7 – 0.5; p = 0.792).

*Alzheimer’s Disease*
In the female cohort, 28 of 2 618 patients without cortisone (1.1 %) and 34 of 2 618 patients with cortisone (1.3 %) were diagnosed with Alzheimer’s disease (RD 0.2 %, 95 % CI –0.4 – 0.8; p = 0.443). In the male cohort, 14 of 2 304 patients with cortisone (0.6 %) versus 35 of 2 302 patients without cortisone (1.5 %) developed Alzheimer’s disease (RD 0.9 %, 95 % CI 0.3 – 1.5; p = 0.003).

*Transient Ischemic Attack (TIA)*
In the female cohort, 88 of 2 550 patients without cortisone (3.5 %) versus 117 of 2 486 patients with cortisone (4.7 %) experienced TIA (RD 1.3 %, 95 % CI 0.2 – 2.3; p = 0.024). In the male cohort, 82 of 2 182 patients with cortisone (3.8 %) versus 73 of 2 246 patients without cortisone (3.3 %) experienced TIA (RD –0.5 %, 95 % CI –1.6 – 0.6; p = 0.358).

*Stroke*
In the female cohort, 183 of 2 506 patients without cortisone (7.3 %) and 195 of 2 324 patients with cortisone (8.4 %) suffered stroke (RD 1.1 %, 95 % CI –0.4 – 2.6; p = 0.159). In the male cohort, 133 of 2 182 patients without cortisone (6.1 %) versus 152 of 2 054 patients with cortisone (7.4 %) suffered stroke (RD –1.3 %, 95 % CI –2.8 – 0.2; p = 0.090).

**Ulcerative Colitis**

After 1:1 propensity score matching, the female cohorts consisted of 1 355 Ulcerative Colitis patients without cortisone and 1 355 matched patients treated with cortisone; the male cohorts comprised 1 110 patients treated with cortisone and 1 110 matched patients without cortisone. The characteristics of all cohorts were well balanced on age after matching (standardized differences < 0.01). After matching, the mean current age of the female no-cortisone and cortisone cohorts was 68.6 years (SD 9.4); for males, the mean current age of the cortisone and no-cortisone cohorts was 67.9 years (SD 9.5).

*Parkinson’s Disease*
In the female cohort, 10 of 1 345 patients without cortisone (0.7 %) versus 10 of 1 340 patients with cortisone (0.7 %) developed Parkinson’s disease (RD 0.0 %, 95 % CI –0.7–0.6; p = 0.993). In the male cohort, 10 of 1 092 patients with cortisone (0.9 %) versus 10 of 1 105 patients without cortisone (0.9 %) developed Parkinson’s disease (RD 0.0 %, 95 % CI –0.8–0.8; p = 0.979).

*Alzheimer’s Disease*
In the female cohort, 10 of 1 349 patients without cortisone (0.7 %) and 10 of 1 349 patients with cortisone (0.7 %) were diagnosed with Alzheimer’s disease (RD 0.0 %, 95 % CI –0.6–0.6; p = 1.000). In the male cohort, 10 of 1 106 patients with cortisone (0.9 %) versus 10 of 1 108 patients without cortisone (0.9 %) developed Alzheimer’s disease (RD 0.0 %, 95 % CI –0.8–0.8; p = 0.997).

*Transient Ischemic Attack (TIA)*
In the female cohort, 33 of 1 307 no-cortisone patients (2.5 %) versus 36 of 1 298 cortisone patients (2.8 %) experienced TIA (RD –0.2 %, 95 % CI –1.5–1.0; p = 0.693). In the male cohort, 19 of 1 064 cortisone patients (1.8 %) and 19 of 1 092 no-cortisone patients (1.7 %) experienced TIA (RD 0.1 %, 95 % CI –1.1–1.2; p = 0.936).

*Stroke*
In the female cohort, 45 of 1 311 no-cortisone patients (3.4 %) and 40 of 1 261 cortisone patients (3.2 %) suffered stroke (RD 0.3 %, 95 % CI –1.1–1.6; p = 0.712). In the male cohort, 42 of 1 033 cortisone patients (4.1 %) versus 28 of 1 075 no-cortisone patients (2.6 %) suffered stroke (RD 1.5 %, 95 % CI –0.1–3.0; p = 0.061).

**Crohn’s Disease**

After 1:1 propensity score matching, the female cohorts consisted of 1,061 Crohn’s disease patients without cortisone and 1,061 matched patients treated with cortisone; the male cohorts comprised 819 cortisone-treated patients and 819 matched patients without cortisone. The characteristics of all cohorts were well balanced on age after matching (standardized differences < 0.01). After matching, the mean current age of the female no-cortisone and cortisone cohorts was 66.8 years (SD 9.5) each; for males, the mean current age of both the cortisone and no-cortisone cohorts was 67.2 years (SD 9.5).

*Parkinson’s Disease*
In the female cohort, 10 of 1,054 patients without cortisone (0.9 %) versus 10 of 1,057 patients with cortisone (0.9 %) developed Parkinson’s disease (RD 0.0 %, 95 % CI –0.8–0.8; p = 0.995). In the male cohort, 10 of 807 cortisone-treated patients (1.2 %) versus 10 of 812 no-cortisone patients (1.2 %) developed Parkinson’s disease (RD 0.0 %, 95 % CI –1.1–1.1; p = 0.989).

*Alzheimer’s Disease*
In the female cohort, 10 of 1,058 no-cortisone patients (0.9 %) and 10 of 1,056 cortisone patients (0.9 %) were diagnosed with Alzheimer’s disease (RD –0.0 %, 95 % CI –0.8–0.8; p = 0.997). In the male cohort, 0 of 815 cortisone patients (0.0 %) versus 10 of 815 no-cortisone patients (1.2 %) developed Alzheimer’s disease (RD –1.2 %, 95 % CI –2.0––0.5; p = 0.002).

*Transient Ischemic Attack (TIA)*In the female cohort, 16 of 1,045 no-cortisone patients (1.5 %) versus 20 of 1,027 cortisone patients (1.9 %) experienced TIA (RD –0.4 %, 95 % CI –1.5–0.7; p = 0.468). In the male cohort, 19 of 795 cortisone patients (2.4 %) and 10 of 813 no-cortisone patients (1.2 %) experienced TIA (RD 1.2 %, 95 % CI –0.1–2.5; p = 0.081).

*Stroke*
In the female cohort, 25 of 1,041 no-cortisone patients (2.4 %) versus 41 of 1,017 cortisone patients (4.0 %) suffered stroke (RD –1.6 %, 95 % CI –3.2––0.1; p = 0.036). In the male cohort, 34 of 781 cortisone patients (4.4 %) versus 21 of 798 no-cortisone patients (2.6 %) suffered stroke (RD 1.7 %, 95 % CI –0.1–3.5; p = 0.062).

**EXPERIMENT 4**

**Chronic Inflammatory Demyelinating Polyneuropathy**
After 1:1 propensity score matching, the male cohorts consisted of 594 CIDP patients without azathioprine and 594 CIDP patients treated with azathioprine; the female cohorts comprised 468 CIDP patients without azathioprine and 468 CIDP patients treated with azathioprine. The cohorts were balanced on age after matching (standardized mean difference for current age: 0.013 in males and 0.050 in females). After matching, the mean current age in the male cohorts was 69.7 years (SD 9.0) in the no-azathioprine group and 69.6 years (SD 9.2) in the azathioprine group; for females, the mean current age was 66.9 years (SD 9.4) versus 67.4 years (SD 9.7), respectively.

There was not enough outcome data for Alzheimer’s and Parkinson’s disease.

*Transient Ischemic Attack (TIA)*In the male cohort, 20 of 573 patients (3.5 %) without azathioprine and 11 of 576 patients (1.9 %) treated with azathioprine experienced TIA, yielding a risk difference of 1.6 % (95 % CI −0.3–3.5), a risk ratio of 1.83 (95 % CI 0.88–3.78), and an odds ratio of 1.86 (95 % CI 0.88–3.91); p = 0.098. There was not enough outcome data in the female cohort.

*Stroke*
In males, stroke occurred in 26 of 556 patients (4.7 %) without azathioprine versus 32 of 565 (5.7 %) treated with azathioprine (RD −1.0 %, 95 % CI −3.6–1.6; RR 0.83, 95 % CI 0.50–1.37; OR 0.82, 95 % CI 0.48–1.39; p = 0.455). In females, 20 of 440 patients (4.5 %) without azathioprine versus 25 of 445 (5.6 %) treated with azathioprine developed stroke (RD −1.1 %, 95 % CI −4.0–1.8; RR 0.81, 95 % CI 0.46–1.44; OR 0.80, 95 % CI 0.44–1.46; p = 0.468).

**Myasthenia Gravis**

After 1:1 propensity score matching, the male cohorts consisted of 3 840 myasthenia gravis patients without azathioprine and 3 840 matched myasthenia gravis patients treated with azathioprine; the female cohorts comprised 3 309 patients without azathioprine and 3 309 azathioprine-treated patients. The characteristics of both cohorts were well balanced on age and immune suppressants after matching (standardized differences < 0.01). After matching, the mean current age of male patients was 71.2 years (SD 9.0) in the no-azathioprine cohort and 71.3 years (SD 9.0) in the azathioprine cohort; for females, the mean current age was 68.2 years (SD 9.9) versus 68.3 years (SD 9.8), respectively.

*Parkinson’s Disease*

In the male cohort, 73 of 3 757 myasthenia gravis patients without azathioprine (1.9 %) and 46 of 3 785 azathioprine-treated patients (1.2 %) developed Parkinson’s disease, yielding a risk difference of 0.7 % (95 % CI 0.2–1.3), a risk ratio of 1.599 (95 % CI 1.109–2.306), and an odds ratio of 1.611 (95 % CI 1.111–2.336); p = 0.011. In the female cohort, 39 of 3 266 patients without azathioprine (1.2 %) versus 34 of 3 278 azathioprine-treated patients (1.0 %) were diagnosed with Parkinson’s disease (RD 0.2 %, 95 % CI −0.004–0.007; RR 1.151, 95 % CI 0.729–1.819; OR 1.153, 95 % CI 0.726–1.831; p = 0.546).

*Alzheimer’s Disease*

Among males, Alzheimer’s disease occurred in 29 of 3 822 myasthenia gravis patients without azathioprine (0.8 %) compared with 22 of 3 827 azathioprine-treated patients (0.6 %) (RD 0.2 %, 95 % CI −0.2–0.5; RR 1.320, 95 % CI 0.760–2.293; OR 1.322, 95 % CI 0.758–2.306; p = 0.323). In females, 23 of 3 298 patients without azathioprine (0.7 %) versus 15 of 3 302 azathioprine-treated patients (0.5 %) developed Alzheimer’s disease (RD 0.2 %, 95 % CI −0.1–0.6; RR 1.535, 95 % CI 0.802–2.937; OR 1.539, 95 % CI 0.802–2.955; p = 0.192).

*Transient Ischemic Attack (TIA)*

TIA was observed in 77 of 3 703 myasthenia gravis males without azathioprine (2.1 %) compared with 80 of 3 729 azathioprine-treated males (2.1 %) (RD −0.1 %, 95 % CI −0.7–0.6; RR 0.969, 95 % CI 0.711–1.321; OR 0.969, 95 % CI 0.706–1.329; p = 0.843). In the female cohort, 79 of 3 189 patients without azathioprine (2.5 %) versus 70 of 3 212 azathioprine-treated patients (2.2 %) experienced TIA (RD 0.3 %, 95 % CI −0.4–1.0; RR 1.137, 95 % CI 0.827–1.562; OR 1.140, 95 % CI 0.823–1.579; p = 0.429).

*Stroke*

Stroke incidence was 148 of 3 635 myasthenia gravis males without azathioprine (4.1 %) versus 124 of 3 634 azathioprine-treated males (3.4 %) (RD 0.7 %, 95 % CI −0.2–1.5; RR 1.193, 95 % CI 0.944–1.508; OR 1.201, 95 % CI 0.942–1.532; p = 0.139). Among females, 105 of 3 132 patients without azathioprine (3.4 %) suffered stroke compared with 93 of 3 194 azathioprine-treated patients (2.9 %) (RD 0.4 %, 95 % CI −0.4–1.3; RR 1.151, 95 % CI 0.875–1.515; OR 1.157, 95 % CI 0.871–1.536; p = 0.314).

**Multiple Sclerosis**

After 1:1 propensity score matching, the male cohorts consisted of 4 201 multiple sclerosis patients without ocrelizumab and 4 201 matched patients treated with ocrelizumab; the female cohorts comprised 8 609 patients without ocrelizumab and 8 609 matched patients treated with ocrelizumab. The characteristics of both cohorts were well balanced on age and immune suppressants after matching (standardized differences ≤ 0.010). After matching, the mean current age of male multiple sclerosis patients was 60.8 years (SD 7.5) in the no-ocrelizumab cohort and 60.8 years (SD 7.4) in the ocrelizumab cohort; for females, the mean current age was 60.1 years (SD 7.3) in both cohorts.

*Parkinson’s Disease*

In the male cohort, 28 of 4 160 multiple sclerosis patients (0.7 %) without ocrelizumab and 29 of 4 181 patients (0.7 %) treated with ocrelizumab developed Parkinson’s disease, yielding a risk difference of −0.0 % (95 % CI −0.4–0.3), a risk ratio of 0.970 (95 % CI 0.578–1.628), and an odds ratio of 0.970 (95 % CI 0.576–1.634); p = 0.909. In the female cohort, 40 of 8 558 patients (0.5 %) without ocrelizumab versus 40 of 8 566 patients (0.5 %) treated with ocrelizumab were diagnosed with Parkinson’s disease (RD 0.0 %, 95 % CI −0.2–0.2; RR 1.001, 95 % CI 0.646–1.550; OR 1.001, 95 % CI 0.645–1.553; p = 0.997).

*Alzheimer’s Disease*

Among males, Alzheimer’s disease occurred in 17 of 4 193 multiple sclerosis patients (0.4 %) without ocrelizumab compared with 11 of 4 200 patients (0.3 %) treated with ocrelizumab (RD 0.1 %, 95 % CI −0.1–0.4; RR 1.548, 95 % CI 0.726–3.301; OR 1.550, 95 % CI 0.725–3.314; p = 0.254). In females, 18 of 8 596 patients (0.2 %) without ocrelizumab versus 21 of 8 593 patients (0.2 %) treated with ocrelizumab developed Alzheimer’s disease (RD −0.0 %, 95 % CI −0.2–0.1; RR 0.857, 95 % CI 0.457–1.607; OR 0.857, 95 % CI 0.456–1.609; p = 0.630).

*Transient Ischemic Attack (TIA)*

TIA was observed in 53 of 4 124 multiple sclerosis males without ocrelizumab (1.3 %) compared with 46 of 4 142 males treated with ocrelizumab (1.1 %) (RD 0.2 %, 95 % CI −0.3–0.6; RR 1.157, 95 % CI 0.781–1.714; OR 1.159, 95 % CI 0.779–1.725; p = 0.466). In the female cohort, 110 of 8 455 patients (1.3 %) without ocrelizumab versus 89 of 8 488 patients (1.0 %) treated with ocrelizumab experienced TIA (RD 0.3 %, 95 % CI −0.1–0.6; RR 1.241, 95 % CI 0.940–1.638; OR 1.244, 95 % CI 0.939–1.648; p = 0.127).

Stroke

Stroke incidence was 133 of 3 982 multiple sclerosis males without ocrelizumab (3.3 %) versus 97 of 4 079 males treated with ocrelizumab (2.4 %) (RD 1.0 %, 95 % CI 0.2–1.7; RR 1.405, 95 % CI 1.085–1.818; OR 1.419, 95 % CI 1.088–1.850; p = 0.009). Among females, 213 of 8 296 patients without ocrelizumab (2.6 %) suffered stroke compared with 185 of 8 385 patients treated with ocrelizumab (2.2 %) (RD 0.4 %, 95 % CI −0.1–0.8; RR 1.164, 95 % CI 0.958–1.414; OR 1.168, 95 % CI 0.957–1.426; p = 0.126).

**Vitiligo**

After 1:1 propensity score matching, the male cohorts consisted of 3 805 vitiligo patients without tacrolimus and 3 805 matched vitiligo patients treated with tacrolimus; the female cohorts comprised 5 618 patients without tacrolimus and 5 618 tacrolimus-treated patients. The characteristics of both cohorts were well balanced after matching (standardized differences ≤ 0.009 in males and ≤ 0.005 in females for age variables; immune suppressants balanced with standardized differences ≤ 0.001). After matching, the mean current age of male vitiligo patients was 64.3 years (SD 9.3) in both cohorts; for females, the mean current age was 64.7 years (SD 9.1) in both cohorts.

*Parkinson’s Disease*

In the male cohort, 30 of 3 781 patients without tacrolimus (0.8 %) and 19 of 3 793 tacrolimus-treated patients (0.5 %) developed Parkinson’s disease, yielding a risk difference of 0.3 % (95 % CI −0.1–0.7), a risk ratio of 1.584 (95 % CI 0.893–2.809), and an odds ratio of 1.589 (95 % CI 0.893–2.827); p = 0.112. In the female cohort, 20 of 5 601 patients without tacrolimus (0.4 %) versus 14 of 5 598 tacrolimus-treated patients (0.3 %) were diagnosed with Parkinson’s disease (RD 0.1 %, 95 % CI −0.1–0.3; RR 1.428, 95 % CI 0.722–2.824; OR 1.429, 95 % CI 0.721–2.833; p = 0.303).

*Alzheimer’s Disease*

Among males, Alzheimer’s disease occurred in 11 of 3 797 patients without tacrolimus (0.3 %) compared with 10 of 3 798 tacrolimus-treated patients (0.3 %) (RD 0.0 %, 95 % CI −0.2–0.3; RR 1.100, 95 % CI 0.468–2.588; OR 1.101, 95 % CI 0.467–2.595; p = 0.827). In females, 22 of 5 607 patients without tacrolimus (0.4 %) versus 17 of 5 608 tacrolimus-treated patients (0.3 %) developed Alzheimer’s disease (RD 0.1 %, 95 % CI −0.1–0.3; RR 1.294, 95 % CI 0.688–2.435; OR 1.296, 95 % CI 0.687–2.442; p = 0.422).

*Transient Ischemic Attack (TIA)*

TIA was observed in 49 of 3 738 vitiligo males without tacrolimus (1.3 %) compared with 43 of 3 748 tacrolimus-treated males (1.1 %) (RD 0.2 %, 95 % CI −0.3–0.7; RR 1.143, 95 % CI 0.761–1.717; OR 1.144, 95 % CI 0.758–1.728; p = 0.521). In the female cohort, 82 of 5 509 patients without tacrolimus (1.5 %) versus 76 of 5 510 tacrolimus-treated patients (1.4 %) experienced TIA (RD 0.1 %, 95 % CI −0.3–0.6; RR 1.079, 95 % CI 0.792–1.471; OR 1.080, 95 % CI 0.789–1.479; p = 0.630).

*Stroke*

Stroke incidence was 90 of 3 718 vitiligo males without tacrolimus (2.4 %) versus 65 of 3 731 tacrolimus-treated males (1.7 %) (RD 0.7 %, 95 % CI 0.0–1.3; RR 1.389, 95 % CI 1.013–1.905; OR 1.399, 95 % CI 1.014–1.931; p = 0.040). Among females, 126 of 5 510 patients without tacrolimus (2.3 %) suffered stroke compared with 104 of 5 507 tacrolimus-treated patients (1.9 %) (RD 0.4 %, 95 % CI −0.1–0.9; RR 1.211, 95 % CI 0.936–1.566; OR 1.216, 95 % CI 0.935–1.581; p = 0.144).

**Rheumatoid Vasculitis**
After 1:1 propensity score matching, the male cohorts consisted of 161 patients in cohort 1 and 161 matched patients in cohort 2; the female cohorts comprised 380 patients in cohort 1 and 380 matched patients in cohort 2. Cohort characteristics were acceptably balanced after matching (maximum standardized mean difference 0.073 in males and 0.039 in females). After matching, the mean current age in males was 68.2 years (SD 9.5) in cohort 1 and 68.9 years (SD 9.2) in cohort 2; in females, the mean current age was 66.8 years (SD 9.0) in cohort 1 and 66.6 years (SD 9.2) in cohort 2.

There was not enough outcome data for any of the four outcomes.

**Graves’ Disease**

After 1:1 propensity score matching, the male cohorts consisted of 28 218 Graves’ disease patients without methimazole and 28 218 matched Graves’ disease patients treated with methimazole; the female cohorts comprised 78 888 patients without methimazole and 78 888 methimazole-treated patients. The characteristics of both cohorts were well balanced on current age, age at index, and immune suppressants after matching (standardized differences ≤ 0.003 in males and ≤ 0.001 in females). After matching, the mean current age of male Graves’ disease patients was 67.0 years (SD 9.6) in both cohorts; for females, the mean current age was 66.2 years (SD 9.7) in both cohorts.

*Parkinson’s Disease*

In the male cohort, 274 of 27 888 Graves’ disease patients without methimazole (1.0 %) and 213 of 27 895 methimazole-treated patients (0.8 %) developed Parkinson’s disease, yielding a risk difference of 0.2 % (95 % CI 0.1–0.4), a risk ratio of 1.287 (95 % CI 1.077–1.538), and an odds ratio of 1.290 (95 % CI 1.077–1.544); p = 0.005. In the female cohort, 428 of 78 461 patients without methimazole (0.5 %) versus 320 of 78 503 methimazole-treated patients (0.4 %) were diagnosed with Parkinson’s disease (RD 0.1 %, 95 % CI 0.1–0.2; RR 1.338, 95 % CI 1.158–1.546; OR 1.340, 95 % CI 1.159–1.549; p < 0.001).

*Alzheimer’s Disease*

Among males, Alzheimer’s disease occurred in 199 of 28 088 Graves’ disease patients without methimazole (0.7 %) compared with 185 of 28 085 methimazole-treated patients (0.7 %) (RD 0.0 %, 95 % CI −0.1–0.2; RR 1.076, 95 % CI 0.881–1.313; OR 1.076, 95 % CI 0.880–1.315; p = 0.474). In females, 521 of 78 544 patients without methimazole (0.7 %) versus 494 of 78 563 methimazole-treated patients (0.6 %) developed Alzheimer’s disease (RD 0.0 %, 95 % CI −0.0–0.1; RR 1.055, 95 % CI 0.933–1.193; OR 1.055, 95 % CI 0.933–1.194; p = 0.393).

*Transient Ischemic Attack (TIA)*

TIA was observed in 544 of 27 547 Graves’ disease males without methimazole (2.0 %) compared with 542 of 27 550 methimazole-treated males (2.0 %) (RD 0.0 %, 95 % CI −0.2–0.2; RR 1.004, 95 % CI 0.892–1.129; OR 1.004, 95 % CI 0.890–1.132; p = 0.950). In the female cohort, 1 526 of 77 284 patients without methimazole (2.0 %) versus 1 293 of 77 305 methimazole-treated patients (1.7 %) experienced TIA (RD 0.3 %, 95 % CI 0.2–0.4; RR 1.181, 95 % CI 1.097–1.270; OR 1.184, 95 % CI 1.099–1.276; p < 0.001).

*Stroke*

Stroke incidence was 962 of 26 683 Graves’ disease males without methimazole (3.6 %) versus 983 of 26 714 methimazole-treated males (3.7 %) (RD −0.1 %, 95 % CI −0.4–0.2; RR 0.980, 95 % CI 0.898–1.069; OR 0.979, 95 % CI 0.894–1.072; p = 0.646). Among females, 2 058 of 76 306 patients without methimazole (2.7 %) suffered stroke compared with 2 239 of 76 070 methimazole-treated patients (2.9 %) (RD −0.2 %, 95 % CI −0.4–−0.1; RR 0.916, 95 % CI 0.864–0.972; OR 0.914, 95 % CI 0.860–0.971; p = 0.004).

**Vasculitis**

After 1:1 propensity score matching, the male cohorts consisted of 509 vasculitis patients without rituximab and 509 vasculitis patients with rituximab; the female cohorts comprised 853 vasculitis patients without rituximab and 853 vasculitis patients with rituximab. The characteristics of both cohorts were well balanced after matching (standardized differences ≤ 0.01). After matching, the mean current age of male vasculitis patients was 68.3 years (SD 9.3) in the no-rituximab cohort and 68.2 years (SD 9.4) in the rituximab cohort; for females, the mean current age was 67.9 years (SD 9.6) in both the no-rituximab and rituximab cohorts.

There was not enough outcome data for Parkinson’s and Alzheimer’s disease.

*Transient Ischemic Attack (TIA)*

TIA results were available for the female cohort only: 17 of 876 females without rituximab (1.9 %) versus 22 of 859 females with rituximab (2.6 %) experienced TIA (RD -0.6 %, 95 % CI -1.9–0.7; RR 0.76, 95 % CI 0.41–1.42; OR 0.75, 95 % CI 0.40–1.42; p = 0.388). There was not enough outcome data in the male cohort.

*Stroke*

In the male cohort, stroke occurred in 20 of 476 vasculitis patients without rituximab (4.2 %) versus 29 of 478 with rituximab (6.1 %) (RD -1.9 %, 95 % CI -4.7–1.0; RR 0.69, 95 % CI 0.40–1.19; OR 0.68, 95 % CI 0.38–1.23; p = 0.194). In the female cohort, 48 of 833 vasculitis patients without rituximab (5.8 %) and 48 of 833 with rituximab (5.8 %) developed stroke (RD 0.0 %, 95 % CI -2.0–2.0; RR 1.00, 95 % CI 0.68–1.48; OR 1.00, 95 % CI 0.66–1.51; p = 0.942).

**Dermatomyositis**

After 1:1 propensity score matching, the male cohorts consisted of 1 852 dermatomyositis patients without methotrexate and 1 852 matched dermatomyositis patients treated with methotrexate; the female cohorts comprised 5 014 patients without methotrexate and 5 014 methotrexate-treated patients. The characteristics of both cohorts were well balanced on current age, age at index, and immune suppressants after matching (male standardized differences ≤ 0.039; female ≤ 0.008). After matching, the mean current age of male patients was 67.9 years (SD 9.2) in the no-methotrexate cohort and 67.7 years (SD 9.2) in the methotrexate cohort; for females, mean current age was 66.6 years (SD 9.4) versus 66.7 years (SD 9.3), respectively.

*Parkinson’s Disease*

In the male cohort, 16 of 1 842 patients without methotrexate (0.9 %) and 20 of 1 840 methotrexate-treated patients (1.1 %) developed Parkinson’s disease, yielding a risk difference of −0.2 % (95 % CI −0.9–0.4), a risk ratio of 0.80 (95 % CI 0.42–1.54), and an odds ratio of 0.80 (95 % CI 0.41–1.54); p = 0.501. In the female cohort, 26 of 4 977 patients without methotrexate (0.5 %) versus 19 of 4 996 methotrexate-treated patients (0.4 %) developed Parkinson’s disease (RD 0.1 %, 95 % CI −0.1–0.4; RR 1.37, 95 % CI 0.76–2.48; OR 1.38, 95 % CI 0.76–2.49; p = 0.290).

*Alzheimer’s Disease*

In the female cohort, Alzheimer’s disease occurred in 35 of 5 002 patients without methotrexate (0.7 %) compared with 29 of 5 009 methotrexate-treated patients (0.6 %) (RD 0.1 %, 95 % CI −0.2–0.4; RR 1.21, 95 % CI 0.74–1.97; OR 1.21, 95 % CI 0.74–1.98; p = 0.448). There was not enough outcome data in the male cohort.

*Transient Ischemic Attack (TIA)*

TIA was observed in 44 of 1 820 dermatomyositis males without methotrexate (2.4 %) compared with 43 of 1 810 methotrexate-treated males (2.4 %) (RD 0.0 %, 95 % CI −1.0–1.0; RR 1.02, 95 % CI 0.67–1.54; OR 1.02, 95 % CI 0.67–1.56; p = 0.934). In the female cohort, 111 of 4 930 patients without methotrexate (2.3 %) versus 117 of 4 910 methotrexate-treated patients (2.4 %) experienced TIA (RD −0.1 %, 95 % CI −0.7–0.5; RR 0.94, 95 % CI 0.73–1.22; OR 0.94, 95 % CI 0.73–1.23; p = 0.665).

*Stroke*

Stroke incidence was 74 of 1 766 dermatomyositis males without methotrexate (4.2 %) versus 70 of 1 779 methotrexate-treated males (3.9 %) (RD 0.3 %, 95 % CI −1.0–1.6; RR 1.06, 95 % CI 0.77–1.47; OR 1.07, 95 % CI 0.76–1.49; p = 0.700). Among females, 190 of 4 843 patients without methotrexate (3.9 %) suffered stroke compared with 147 of 4 892 methotrexate-treated patients (3.0 %) (RD 0.9 %, 95 % CI 0.2–1.6; RR 1.31, 95 % CI 1.06–1.61; OR 1.32, 95 % CI 1.06–1.64; p = 0.013).

**Arthropathic Psoriasis**
After 1:1 propensity score matching, the male cohorts consisted of 9,362 arthropathic psoriasis patients without adalimumab and 9,362 matched patients treated with adalimumab; the female cohorts consisted of 12,790 patients without adalimumab and 12,790 adalimumab-treated patients. After matching, characteristics were well balanced across current age and age at index (standardized differences ≤ 0.009 in males and ≤ 0.002 in females); immune suppressants were closely balanced (male standardized difference 0.011; female < 0.001). After matching, mean current age was 63.5 years (SD 8.3) in both male cohorts; in females, mean current age was 63.2 years (SD 8.4) in the no-adalimumab cohort and 63.3 years (SD 8.4) in the adalimumab cohort.

*Alzheimer’s Disease*
In the male cohort, Alzheimer’s disease occurred in 36 of 9,353 patients without adalimumab (0.4%) versus 30 of 9,351 adalimumab-treated patients (0.3%) (RD 0.1%, 95% CI −0.1 to 0.2; RR 1.200, 95% CI 0.740–1.946; OR 1.201, 95% CI 0.739–1.951; p = 0.460). In the female cohort, 34 of 12,776 patients without adalimumab (0.3%) versus 53 of 12,778 adalimumab-treated patients (0.4%) developed Alzheimer’s disease (RD −0.1%, 95% CI −0.3 to −0.0; RR 0.642, 95% CI 0.417–0.986; OR 0.641, 95% CI 0.416–0.986; p = 0.041).

*Parkinson’s Disease*
In the male cohort, 60 of 9,324 patients without adalimumab (0.6%) versus 55 of 9,332 adalimumab-treated patients (0.6%) developed Parkinson’s disease (RD 0.1%, 95% CI −0.2 to 0.3; RR 1.092, 95% CI 0.758–1.572; OR 1.092, 95% CI 0.757–1.577; p = 0.637). In the female cohort, 53 of 12,757 patients without adalimumab (0.4%) versus 65 of 12,752 adalimumab-treated patients (0.5%) developed Parkinson’s disease (RD −0.1%, 95% CI −0.3 to 0.1; RR 0.815, 95% CI 0.568–1.170; OR 0.814, 95% CI 0.566–1.171; p = 0.267).

*Transient Ischemic Attack (TIA)*TIA was observed in 155 of 9,225 males without adalimumab (1.7%) compared with 148 of 9,243 adalimumab-treated males (1.6%) (RD 0.1%, 95% CI −0.3 to 0.4; RR 1.049, 95% CI 0.839–1.312; OR 1.050, 95% CI 0.837–1.318; p = 0.673). In the female cohort, 204 of 12,601 patients without adalimumab (1.6%) versus 208 of 12,591 adalimumab-treated patients (1.7%) experienced TIA (RD 0.0%, 95% CI −0.3 to 0.3; RR 0.980, 95% CI 0.809–1.187; OR 0.980, 95% CI 0.806–1.190; p = 0.836).

*Stroke*
Stroke occurred in 221 of 9,174 males without adalimumab (2.4%) versus 213 of 9,205 adalimumab-treated males (2.3%) (RD 0.1%, 95% CI −0.3 to 0.5; RR 1.041, 95% CI 0.864–1.254; OR 1.042, 95% CI 0.861–1.261; p = 0.671). In the female cohort, 262 of 12,543 patients without adalimumab (2.1%) versus 295 of 12,573 adalimumab-treated patients (2.3%) had stroke (RD −0.3%, 95% CI −0.6 to 0.1; RR 0.890, 95% CI 0.755–1.049; OR 0.888, 95% CI 0.750–1.051; p = 0.166).

**Psoriasis**

After 1:1 propensity score matching, the male cohorts consisted of 7,491 psoriasis patients without secukinumab and 7,491 matched psoriasis patients treated with secukinumab; the female cohorts consisted of 11,116 patients without secukinumab and 11,116 secukinumab-treated patients. Characteristics were well balanced after matching across current age, age at index, and immune suppressants (standardized differences ~0.001–0.002; immune suppressants identical within sex after matching). After matching, mean current age in males was 63.8 years (SD 8.5) in both cohorts, with mean age at index 58.9 years (SD 8.8) in both cohorts; in females, mean current age was 63.4 years (SD 8.5) in both cohorts, with mean age at index 58.7 years (SD 8.6) vs 58.6 years (SD 8.6) (no secukinumab vs secukinumab).

*Alzheimer’s Disease*
In the male cohort, Alzheimer’s disease occurred in 26 of 7,473 patients without secukinumab (0.3%) versus 24 of 7,479 secukinumab-treated patients (0.3%), yielding a risk difference of 0.0% (95% CI −0.2 to 0.2), a risk ratio of 1.084 (95% CI 0.623–1.887), and an odds ratio of 1.084 (95% CI 0.622–1.891); p = 0.775. In the female cohort, 34 of 11,095 patients without secukinumab (0.3%) versus 27 of 11,102 secukinumab-treated patients (0.2%) developed Alzheimer’s disease (RD 0.1%, 95% CI −0.1 to 0.2; RR 1.260, 95% CI 0.761–2.087; OR 1.261, 95% CI 0.760–2.091; p = 0.368).

*Parkinson’s Disease*In the male cohort, 28 of 7,446 patients without secukinumab (0.4%) versus 42 of 7,441 secukinumab-treated patients (0.6%) developed Parkinson’s disease (RD −0.2%, 95% CI −0.4 to 0.0; RR 0.666, 95% CI 0.413–1.074; OR 0.665, 95% CI 0.412–1.074; p = 0.093). In the female cohort, Parkinson’s disease occurred in 38 of 11,082 patients without secukinumab (0.3%) versus 39 of 11,075 secukinumab-treated patients (0.4%) (RD −0.0%, 95% CI −0.2 to 0.1; RR 0.974, 95% CI 0.623–1.521; OR 0.974, 95% CI 0.622–1.523; p = 0.907).

*Transient Ischemic Attack (TIA)*TIA was observed in 112 of 7,353 psoriasis males without secukinumab (1.5%) compared with 109 of 7,342 secukinumab-treated males (1.5%) (RD 0.0%, 95% CI −0.4 to 0.4; RR 1.026, 95% CI 0.790–1.333; OR 1.026, 95% CI 0.787–1.339; p = 0.848). In the female cohort, 156 of 10,914 patients without secukinumab (1.4%) versus 128 of 10,877 secukinumab-treated patients (1.2%) experienced TIA (RD 0.3%, 95% CI −0.0 to 0.6; RR 1.215, 95% CI 0.963–1.532; OR 1.218, 95% CI 0.962–1.541; p = 0.100).

*Stroke*
Stroke occurred in 184 of 7,274 psoriasis males without secukinumab (2.5%) versus 169 of 7,257 secukinumab-treated males (2.3%) (RD 0.2%, 95% CI −0.3 to 0.7; RR 1.086, 95% CI 0.884–1.335; OR 1.088, 95% CI 0.881–1.345; p = 0.432). In the female cohort, 217 of 10,859 patients without secukinumab (2.0%) versus 206 of 10,846 secukinumab-treated patients (1.9%) had stroke (RD 0.1%, 95% CI −0.3 to 0.5; RR 1.052, 95% CI 0.871–1.271; OR 1.053, 95% CI 0.869–1.277; p = 0.598).

**Sjögren Syndrome**
After 1:1 propensity score matching, the male cohorts consisted of 6,010 Sjögren syndrome patients without hydroxychloroquine and 6,010 matched Sjögren syndrome patients treated with hydroxychloroquine; the female cohorts consisted of 62,163 patients without hydroxychloroquine and 62,163 hydroxychloroquine-treated patients. Characteristics were well balanced after matching (standardized differences ≤ 0.009 across current age, age at index, and immune suppressants). After matching, mean current age in males was 67.9 years (SD 9.3) in the no-hydroxychloroquine cohort and 68.0 years (SD 9.3) in the hydroxychloroquine cohort; in females, mean current age was 66.1 years (SD 9.3) in both cohorts.

*Parkinson’s Disease*
In the male cohort, 59 of 5,937 patients without hydroxychloroquine (1.0%) versus 83 of 5,944 hydroxychloroquine-treated patients (1.4%) developed Parkinson’s disease, yielding a risk difference of −0.4% (95% CI −0.8 to −0.0), a risk ratio of 0.712 (95% CI 0.511–0.992), and an odds ratio of 0.709 (95% CI 0.507–0.992); p = 0.043.
In the female cohort, 381 of 61,845 patients without hydroxychloroquine (0.6%) versus 462 of 61,847 hydroxychloroquine-treated patients (0.7%) developed Parkinson’s disease (RD −0.1%, 95% CI −0.2 to −0.0; RR 0.825, 95% CI 0.720–0.944; OR 0.824, 95% CI 0.719–0.944; p = 0.005).

*Alzheimer’s Disease*In the male cohort, Alzheimer’s disease occurred in 33 of 6,000 patients without hydroxychloroquine (0.6%) and 33 of 5,996 hydroxychloroquine-treated patients (0.6%) (RD −0.0%, 95% CI −0.3 to 0.3; RR 0.999, 95% CI 0.618–1.617; OR 0.999, 95% CI 0.616–1.621; p = 0.998). In the female cohort, 347 of 62,059 patients without hydroxychloroquine (0.6%) versus 368 of 62,073 hydroxychloroquine-treated patients (0.6%) developed Alzheimer’s disease (RD −0.0%, 95% CI −0.1 to 0.1; RR 0.943, 95% CI 0.815–1.092; OR 0.943, 95% CI 0.814–1.092; p = 0.433).

*Transient Ischemic Attack (TIA)*TIA was observed in 142 of 5,823 Sjögren syndrome males without hydroxychloroquine (2.4%) compared with 180 of 5,790 hydroxychloroquine-treated males (3.1%) (RD −0.7%, 95% CI −1.3 to −0.1; RR 0.784, 95% CI 0.631–0.974; OR 0.779, 95% CI 0.623–0.974; p = 0.028). In the female cohort, 1,380 of 60,824 patients without hydroxychloroquine (2.3%) versus 1,786 of 60,381 hydroxychloroquine-treated patients (3.0%) experienced TIA (RD −0.7%, 95% CI −0.9 to −0.5; RR 0.767, 95% CI 0.716–0.822; OR 0.762, 95% CI 0.709–0.818; p < 0.001).

*Stroke*
Stroke occurred in 171 of 5,759 Sjögren syndrome males without hydroxychloroquine (3.0%) versus 228 of 5,762 hydroxychloroquine-treated males (4.0%) (RD −1.0%, 95% CI −1.7 to −0.3; RR 0.750, 95% CI 0.618–0.912; OR 0.743, 95% CI 0.607–0.909; p = 0.004).
In the female cohort, 1,511 of 60,725 patients without hydroxychloroquine (2.5%) versus 1,825 of 60,462 hydroxychloroquine-treated patients (3.0%) had stroke (RD −0.5%, 95% CI −0.7 to −0.3; RR 0.824, 95% CI 0.771–0.882; OR 0.820, 95% CI 0.765–0.879; p < 0.001).

**Scleroderma (Systemic sclerosis)**

After 1:1 propensity score matching, the male cohorts consisted of 1,989 mycophenolate-treated patients and 1,989 matched patients without mycophenolate (before matching: 1) 9,266; 2) 1,995). In the female analysis, the cohorts comprised 7,562 mycophenolate-treated patients and 7,562 matched patients without mycophenolate (before matching: 1) 45,699; 2) 7,591). In females after matching, mean current age was 65.9 years (SD 9.0) in the mycophenolate cohort and 66.1 years (SD 8.9) in the no-mycophenolate cohort; mean age at index was 59.1 years (SD 9.7) vs 59.5 years (SD 9.7), respectively.

*Alzheimer’s Disease*In the female cohort, 21 of 7,553 mycophenolate-treated patients (0.3 %) versus 28 of 7,549 no-mycophenolate patients (0.4 %) were diagnosed with Alzheimer’s disease (RD -0.1 %, 95 % CI -0.3–0.1; RR 0.75, 95 % CI 0.43–1.32; OR 0.75, 95 % CI 0.42–1.32; p = 0.316). There was not enough outcome data in the male cohort.

*Parkinson’s Disease*
In the male cohort, 13 of 1,980 mycophenolate-treated patients (0.7 %) versus 18 of 1,966 no-mycophenolate patients (0.9 %) developed Parkinson’s disease (RD -0.3 %, 95 % CI -0.8–0.3; RR 0.72, 95 % CI 0.35–1.46; OR 0.72, 95 % CI 0.35–1.46; p = 0.357).
In the female cohort, 14 of 7,540 mycophenolate-treated patients (0.2 %) versus 29 of 7,529 no-mycophenolate patients (0.4 %) developed Parkinson’s disease (RD -0.2 %, 95 % CI -0.4–0.0; RR 0.48, 95 % CI 0.25–0.91; OR 0.48, 95 % CI 0.25–0.91; p = 0.022).

*Transient Ischemic Attack (TIA)*In the male cohort, 27 of 1,952 mycophenolate-treated patients (1.4 %) versus 26 of 1,938 no-mycophenolate patients (1.3 %) experienced TIA (RD 0.0 %, 95 % CI -0.7–0.8; RR 1.03, 95 % CI 0.60–1.76; OR 1.03, 95 % CI 0.60–1.77; p = 0.911). In the female cohort, 130 of 7,413 mycophenolate-treated patients (1.8 %) versus 128 of 7,423 no-mycophenolate patients (1.7 %) experienced TIA (RD 0.0 %, 95 % CI -0.4–0.5; RR 1.02, 95 % CI 0.80–1.30; OR 1.02, 95 % CI 0.80–1.30; p = 0.891).

*Stroke*
In the male cohort, stroke occurred in 43 of 1,923 mycophenolate-treated patients (2.2 %) versus 59 of 1,899 no-mycophenolate patients (3.1 %) (RD -0.9 %, 95 % CI -1.9–0.2; RR 0.72, 95 % CI 0.49–1.06; OR 0.71, 95 % CI 0.48–1.06; p = 0.095). In the female cohort, stroke occurred in 236 of 7,349 mycophenolate-treated patients (3.2 %) versus 193 of 7,326 no-mycophenolate patients (2.6 %) (RD 0.6 %, 95 % CI 0.0–1.1; RR 1.22, 95 % CI 1.01–1.47; OR 1.23, 95 % CI 1.01–1.49; p = 0.038).

**Myositis**

After 1:1 propensity score matching, the male cohorts consisted of 4,991 myositis patients without methotrexate and 4,991 matched myositis patients treated with methotrexate; the female cohorts consisted of 20,857 patients without methotrexate and 20,857 methotrexate-treated patients. After matching, baseline characteristics were well balanced for current age and age at index (standardized differences < 0.001 in both sexes), and immune suppressants were closely balanced (male standardized difference 0.001; female < 0.001). After matching, mean current age in males was 68.1 years (SD 9.2) in both cohorts; in females, mean current age was 66.3 years (SD 9.2) in both cohorts.

*Parkinson’s Disease*In the male cohort, 57 of 4,924 patients without methotrexate (1.2%) and 50 of 4,939 methotrexate-treated patients (1.0%) developed Parkinson’s disease, yielding a risk difference of 0.1% (95% CI −0.3 to 0.6), a risk ratio of 1.143 (95% CI 0.784–1.668), and an odds ratio of 1.145 (95% CI 0.782–1.678); p = 0.486. In the female cohort, 157 of 20,756 patients without methotrexate (0.8%) versus 183 of 20,764 methotrexate-treated patients (0.9%) were diagnosed with Parkinson’s disease (RD −0.1%, 95% CI −0.3 to 0.0; RR 0.858, 95% CI 0.694–1.061; OR 0.857, 95% CI 0.692–1.062; p = 0.158).

*Alzheimer’s Disease*
In the male cohort, Alzheimer’s disease occurred in 33 of 4,981 patients without methotrexate (0.7%) compared with 28 of 4,979 methotrexate-treated patients (0.6%) (RD 0.1%, 95% CI −0.2 to 0.4; RR 1.178, 95% CI 0.713–1.946; OR 1.179, 95% CI 0.712–1.954; p = 0.522). In the female cohort, 166 of 20,811 patients without methotrexate (0.8%) versus 146 of 20,822 methotrexate-treated patients (0.7%) developed Alzheimer’s disease (RD 0.1%, 95% CI −0.1 to 0.3; RR 1.138, 95% CI 0.912–1.420; OR 1.139, 95% CI 0.911–1.423; p = 0.254).

*Transient Ischemic Attack (TIA)*
In the male cohort, TIA was observed in 137 of 4,822 patients without methotrexate (2.8%) compared with 161 of 4,780 methotrexate-treated patients (3.4%) (RD −0.5%, 95% CI −1.2 to 0.2; RR 0.844, 95% CI 0.674–1.056; OR 0.839, 95% CI 0.666–1.057; p = 0.136). In the female cohort, 614 of 20,329 patients without methotrexate (3.0%) versus 582 of 20,124 methotrexate-treated patients (2.9%) experienced TIA (RD 0.1%, 95% CI −0.2 to 0.5; RR 1.044, 95% CI 0.934–1.168; OR 1.046, 95% CI 0.932–1.173; p = 0.446).

*Stroke*
In the male cohort, stroke occurred in 252 of 4,731 patients without methotrexate (5.3%) versus 252 of 4,751 methotrexate-treated patients (5.3%) (RD 0.0%, 95% CI −0.9 to 0.9; RR 1.004, 95% CI 0.847–1.190; OR 1.004, 95% CI 0.839–1.202; p = 0.961). In the female cohort, 747 of 20,269 patients without methotrexate (3.7%) compared with 896 of 20,158 methotrexate-treated patients (4.4%) had stroke (RD −0.8%, 95% CI −1.1 to −0.4; RR 0.829, 95% CI 0.754–0.912; OR 0.823, 95% CI 0.745–0.908; p < 0.001).

**Rheumatoid Arthritis**

After 1:1 propensity score matching, the male cohorts consisted of 34,580 rheumatoid arthritis patients without methotrexate and 34,580 matched rheumatoid arthritis patients treated with methotrexate; the female cohorts consisted of 103,594 patients without methotrexate and 103,594 methotrexate-treated patients. The characteristics of both cohorts were well balanced on current age, age at index, and immune suppressants after matching (standardized differences < 0.001 in both sexes). After matching, the mean current age in males was 69.7 years (SD 9.0) in both cohorts; in females, the mean current age was 68.2 years (SD 9.3–9.4) in both cohorts.

*Parkinson’s Disease*
In the male cohort, 303 of 34,220 patients without methotrexate (0.9%) and 393 of 34,267 methotrexate-treated patients (1.1%) developed Parkinson’s disease, yielding a risk difference of −0.3% (95% CI −0.4 to −0.1), a risk ratio of 0.772 (95% CI 0.665–0.896), and an odds ratio of 0.770 (95% CI 0.662–0.895); p = 0.001. In the female cohort, 544 of 103,039 patients without methotrexate (0.5%) versus 653 of 103,115 methotrexate-treated patients (0.6%) were diagnosed with Parkinson’s disease (RD −0.1%, 95% CI −0.2 to −0.0; RR 0.834, 95% CI 0.744–0.934; OR 0.833, 95% CI 0.743–0.933; p = 0.002).

*Alzheimer’s Disease*In the male cohort, Alzheimer’s disease occurred in 215 of 34,447 patients without methotrexate (0.6%) versus 307 of 34,482 methotrexate-treated patients (0.9%), with a risk difference of −0.3% (95% CI −0.4 to −0.1), a risk ratio of 0.701 (95% CI 0.589–0.834), and an odds ratio of 0.699 (95% CI 0.587–0.833); p < 0.001. In the female cohort, 793 of 103,212 patients without methotrexate (0.8%) versus 893 of 103,328 methotrexate-treated patients (0.9%) developed Alzheimer’s disease (RD −0.1%, 95% CI −0.2 to −0.0; RR 0.889, 95% CI 0.808–0.978; OR 0.888, 95% CI 0.807–0.978; p = 0.015).

*Transient Ischemic Attack (TIA)*In the male cohort, TIA was observed in 679 of 33,867 patients without methotrexate (2.0%) compared with 858 of 33,801 methotrexate-treated patients (2.5%) (RD −0.5%, 95% CI −0.8 to −0.3; RR 0.790, 95% CI 0.715–0.872; OR 0.786, 95% CI 0.709–0.870; p < 0.001). In the female cohort, 2,413 of 101,531 patients without methotrexate (2.4%) versus 2,493 of 101,420 methotrexate-treated patients (2.5%) experienced TIA (RD −0.1%, 95% CI −0.2 to 0.1; RR 0.967, 95% CI 0.915–1.022; OR 0.966, 95% CI 0.913–1.022; p = 0.232).

*Stroke*
In the male cohort, stroke occurred in 1,386 of 33,178 patients without methotrexate (4.2%) versus 1,418 of 33,346 methotrexate-treated patients (4.3%) (RD −0.1%, 95% CI −0.4 to 0.2; RR 0.982, 95% CI 0.914–1.056; OR 0.982, 95% CI 0.910–1.059; p = 0.631). In the female cohort, 3,771 of 100,242 patients without methotrexate (3.8%) versus 3,726 of 100,642 methotrexate-treated patients (3.7%) had stroke (RD 0.1%, 95% CI −0.1 to 0.2; RR 1.016, 95% CI 0.972–1.062; OR 1.017, 95% CI 0.971–1.065; p = 0.481).

**Type 1 Diabetes Mellitus**
After 1:1 propensity score matching, the male cohorts consisted of 99,370 type 1 diabetes mellitus patients without insulin and 99,370 matched patients treated with insulin; the female cohorts consisted of 91,096 patients without insulin and 91,096 insulin-treated patients. The characteristics of both cohorts were well balanced on current age, age at index, and immune suppressants after matching (standardized differences ≤ 0.002 in males and < 0.001 in females; immune suppressants identical within each sex). After matching, mean current age in males was 68.3 years (SD 9.5) in both cohorts; for females, the mean current age was 68.5 years (SD 9.6) in both cohorts.

*Parkinson’s Disease*In the male cohort, 578 of 98,660 patients without insulin (0.6%) versus 1,101 of 98,405 insulin-treated patients (1.1%) developed Parkinson’s disease, yielding a risk difference of −0.5% (95% CI −0.6 to −0.5), a risk ratio of 0.524 (95% CI 0.474–0.579), and an odds ratio of 0.521 (95% CI 0.471–0.576); p < 0.001. In the female cohort, 392 of 90,683 patients without insulin (0.4%) versus 783 of 90,456 insulin-treated patients (0.9%) were diagnosed with Parkinson’s disease (RD −0.4%, 95% CI −0.5 to −0.4; RR 0.499, 95% CI 0.443–0.564; OR 0.497, 95% CI 0.440–0.562; p < 0.001).

*Alzheimer’s Disease*In the male cohort, Alzheimer’s disease occurred in 374 of 99,011 patients without insulin (0.4%) compared with 883 of 98,911 insulin-treated patients (0.9%) (RD −0.5%, 95% CI −0.6 to −0.4; RR 0.423, 95% CI 0.375–0.477; OR 0.421, 95% CI 0.373–0.475; p < 0.001). In the female cohort, 499 of 90,697 patients without insulin (0.6%) versus 1,095 of 90,537 insulin-treated patients (1.2%) developed Alzheimer’s disease (RD −0.7%, 95% CI −0.7 to −0.6; RR 0.455, 95% CI 0.409–0.505; OR 0.452, 95% CI 0.406–0.503; p < 0.001).

*Transient Ischemic Attack (TIA)*In the male cohort, TIA was observed in 992 of 97,755 patients without insulin (1.0%) compared with 3,200 of 96,149 insulin-treated patients (3.3%) (RD −2.3%, 95% CI −2.4 to −2.2; RR 0.305, 95% CI 0.284–0.327; OR 0.298, 95% CI 0.277–0.320; p < 0.001). In the female cohort, 1,103 of 89,440 patients without insulin (1.2%) versus 3,480 of 87,672 insulin-treated patients (4.0%) experienced TIA (RD −2.7%, 95% CI −2.9 to −2.6; RR 0.311, 95% CI 0.291–0.332; OR 0.302, 95% CI 0.282–0.323; p < 0.001).

*Stroke*
In the male cohort, stroke occurred in 2,006 of 95,337 patients without insulin (2.1%) versus 6,719 of 91,850 insulin-treated patients (7.3%) (RD −5.2%, 95% CI −5.4 to −5.0; RR 0.288, 95% CI 0.274–0.302; OR 0.272, 95% CI 0.259–0.286; p < 0.001). In the female cohort, 1,882 of 87,855 patients without insulin (2.1%) compared with 6,608 of 84,153 insulin-treated patients (7.9%) suffered stroke (RD −5.7%, 95% CI −5.9 to −5.5; RR 0.273, 95% CI 0.259–0.287; OR 0.257, 95% CI 0.244–0.271; p < 0.001).

**Autoimmune Thyroiditis**

After 1:1 propensity score matching, the male cohorts consisted of 18,013 autoimmune thyroiditis patients without levothyroxine and 18,013 matched patients treated with levothyroxine; the female cohorts consisted of 110,144 patients without levothyroxine and 110,144 matched levothyroxine-treated patients. The characteristics of both cohorts were well balanced on current age, age at index, and immune suppressants after matching (male standardized differences ≤ 0.009; female ≤ 0.002; immune suppressants < 0.001). After matching, mean current age in males was 66.0 years (SD 9.4) in the no-levothyroxine cohort versus 65.9 years (SD 9.4) in the levothyroxine cohort; in females, mean current age was 64.0 years (SD 9.2) versus 64.0 years (SD 9.1), respectively.

*Parkinson’s Disease*
In the male cohort, 133 of 17,833 patients without levothyroxine (0.7%) and 143 of 17,838 levothyroxine-treated patients (0.8%) developed Parkinson’s disease, yielding a risk difference of −0.1% (95% CI −0.2 to 0.1), a risk ratio of 0.930 (95% CI 0.735–1.177), and an odds ratio of 0.930 (95% CI 0.734–1.179); p = 0.547. In the female cohort, 343 of 109,761 patients without levothyroxine (0.3%) versus 458 of 109,692 levothyroxine-treated patients (0.4%) were diagnosed with Parkinson’s disease (RD −0.1%, 95% CI −0.2 to −0.1; RR 0.748, 95% CI 0.651–0.861; OR 0.748, 95% CI 0.650–0.860; p < 0.001).

*Alzheimer’s Disease*In the male cohort, Alzheimer’s disease occurred in 89 of 17,932 patients without levothyroxine (0.5%) versus 82 of 17,934 levothyroxine-treated patients (0.5%) (RD 0.0%, 95% CI −0.1 to 0.2; RR 1.085, 95% CI 0.805–1.464; OR 1.086, 95% CI 0.804–1.467; p = 0.591). In the female cohort, 375 of 109,895 patients without levothyroxine (0.3%) versus 511 of 109,841 levothyroxine-treated patients (0.5%) developed Alzheimer’s disease (RD −0.1%, 95% CI −0.2 to −0.1; RR 0.733, 95% CI 0.642–0.838; OR 0.733, 95% CI 0.641–0.837; p < 0.001).

*Transient Ischemic Attack (TIA)*In the male cohort, TIA was observed in 252 of 17,616 patients without levothyroxine (1.4%) compared with 337 of 17,545 levothyroxine-treated patients (1.9%) (RD −0.5%, 95% CI −0.8 to −0.2; RR 0.745, 95% CI 0.633–0.876; OR 0.741, 95% CI 0.629–0.874; p < 0.001). In the female cohort, 1,217 of 108,514 patients without levothyroxine (1.1%) versus 1,862 of 107,934 levothyroxine-treated patients (1.7%) experienced TIA (RD −0.6%, 95% CI −0.7 to −0.5; RR 0.650, 95% CI 0.605–0.698; OR 0.646, 95% CI 0.601–0.695; p < 0.001).

*Stroke*
In the male cohort, stroke occurred in 355 of 17,346 patients without levothyroxine (2.0%) versus 479 of 17,344 levothyroxine-treated patients (2.8%) (RD −0.7%, 95% CI −1.0 to −0.4; RR 0.741, 95% CI 0.647–0.849; OR 0.736, 95% CI 0.640–0.845; p < 0.001). In the female cohort, 1,324 of 108,201 patients without levothyroxine (1.2%) versus 2,170 of 107,654 levothyroxine-treated patients (2.0%) had stroke (RD −0.8%, 95% CI −0.9 to −0.7; RR 0.607, 95% CI 0.567–0.650; OR 0.602, 95% CI 0.562–0.645; p < 0.001).

**Lupus Erythematosus**

After 1:1 propensity score matching, the male cohorts consisted of 3,498 lupus erythematosus patients without hydroxychloroquine and 3,498 matched patients treated with hydroxychloroquine; the female cohorts consisted of 20,108 patients without hydroxychloroquine and 20,108 matched hydroxychloroquine-treated patients. After matching, cohort characteristics were well balanced for age, age at index, and immune suppressants (standardized differences ≤ 0.023 in males and ≤ 0.007 in females; immune suppressants identical in both cohorts within each sex). After matching, mean current age in males was 66.4 years (SD 9.2) in the no-hydroxychloroquine cohort and 66.2 years (SD 9.3) in the hydroxychloroquine cohort; in females, mean current age was 65.1 years (SD 9.2) in both cohorts.

*Parkinson’s Disease*In the male cohort, 21 of 3,479 patients without hydroxychloroquine (0.6%) and 31 of 3,478 hydroxychloroquine-treated patients (0.9%) developed Parkinson’s disease, yielding a risk difference of −0.3% (95% CI −0.7 to 0.1), a risk ratio of 0.677 (95% CI 0.390–1.176), and an odds ratio of 0.675 (95% CI 0.387–1.177); p = 0.164. In the female cohort, 80 of 20,029 patients without hydroxychloroquine (0.4%) versus 101 of 20,036 hydroxychloroquine-treated patients (0.5%) were diagnosed with Parkinson’s disease (RD −0.1%, 95% CI −0.2 to 0.0; RR 0.792, 95% CI 0.591–1.062; OR 0.792, 95% CI 0.590–1.062; p = 0.118).

*Alzheimer’s Disease*In the male cohort, Alzheimer’s disease occurred in 21 of 3,492 patients without hydroxychloroquine (0.6%) and 21 of 3,488 hydroxychloroquine-treated patients (0.6%) (RD −0.0%, 95% CI −0.4 to 0.4; RR 0.999, 95% CI 0.547–1.826; OR 0.999, 95% CI 0.545–1.832; p = 0.997). In the female cohort, 110 of 20,070 patients without hydroxychloroquine (0.5%) versus 90 of 20,070 hydroxychloroquine-treated patients (0.4%) developed Alzheimer’s disease (RD 0.1%, 95% CI −0.0 to 0.2; RR 1.222, 95% CI 0.926–1.614; OR 1.223, 95% CI 0.925–1.618; p = 0.156).

*Transient Ischemic Attack (TIA)*In the male cohort, TIA was observed in 72 of 3,421 patients without hydroxychloroquine (2.1%) compared with 88 of 3,403 hydroxychloroquine-treated patients (2.6%) (RD −0.5%, 95% CI −1.2 to 0.2; RR 0.814, 95% CI 0.598–1.107; OR 0.810, 95% CI 0.591–1.110; p = 0.189). In the female cohort, 494 of 19,627 patients without hydroxychloroquine (2.5%) versus 608 of 19,481 hydroxychloroquine-treated patients (3.1%) experienced TIA (RD −0.6%, 95% CI −0.9 to −0.3; RR 0.806, 95% CI 0.717–0.907; OR 0.801, 95% CI 0.711–0.904; p < 0.001).

*Stroke*
In the male cohort, stroke occurred in 125 of 3,356 patients without hydroxychloroquine (3.7%) versus 163 of 3,315 hydroxychloroquine-treated patients (4.9%) (RD −1.2%, 95% CI −2.2 to −0.2; RR 0.758, 95% CI 0.603–0.952; OR 0.748, 95% CI 0.590–0.949; p = 0.017). In the female cohort, 755 of 19,403 patients without hydroxychloroquine (3.9%) versus 955 of 19,178 hydroxychloroquine-treated patients (5.0%) had stroke (RD −1.1%, 95% CI −1.5 to −0.7; RR 0.781, 95% CI 0.712–0.858; OR 0.773, 95% CI 0.701–0.852; p < 0.001).

**Ulcerative Colitis**

After 1:1 propensity score matching, the male cohorts consisted of 31,425 ulcerative colitis patients without mesalamine and 31,425 matched ulcerative colitis patients treated with mesalamine; the female cohorts comprised 34,013 patients without mesalamine and 34,013 mesalamine-treated patients. After matching, baseline characteristics were closely balanced for age and age at index (male standardized differences 0.002–0.004; female <0.001–0.003); immune suppressants were also balanced in males (standardized difference 0.008) and slightly imbalanced in females (standardized difference 0.016). After matching, mean current age in males was 66.5 years (SD 9.7) in both cohorts; in females, mean current age was 66.2 years (SD 9.7) in both cohorts.

*Parkinson’s Disease*
In the male cohort, 243 of 31,175 patients without mesalamine (0.8%) and 267 of 31,187 mesalamine-treated patients (0.9%) developed Parkinson’s disease, yielding a risk difference of −0.1% (95% CI −0.2 to 0.1), a risk ratio of 0.910 (95% CI 0.766–1.082), and an odds ratio of 0.910 (95% CI 0.764–1.083); p = 0.288. In the female cohort, 163 of 33,855 patients without mesalamine (0.5%) versus 166 of 33,859 mesalamine-treated patients (0.5%) were diagnosed with Parkinson’s disease (RD −0.0%, 95% CI −0.1 to 0.1; RR 0.982, 95% CI 0.792–1.218; OR 0.982, 95% CI 0.791–1.220; p = 0.869).

*Alzheimer’s Disease*In the male cohort, Alzheimer’s disease occurred in 168 of 31,345 patients without mesalamine (0.5%) versus 160 of 31,340 mesalamine-treated patients (0.5%), with a risk difference of 0.0% (95% CI −0.1 to 0.1), a risk ratio of 1.050 (95% CI 0.846–1.303), and an odds ratio of 1.050 (95% CI 0.845–1.305); p = 0.659. In the female cohort, 204 of 33,907 patients without mesalamine (0.6%) versus 237 of 33,914 mesalamine-treated patients (0.7%) developed Alzheimer’s disease (RD −0.1%, 95% CI −0.2 to 0.0; RR 0.861, 95% CI 0.714–1.038; OR 0.860, 95% CI 0.713–1.038; p = 0.115).

*Transient Ischemic Attack (TIA)*In the male cohort, TIA was observed in 496 of 30,902 patients without mesalamine (1.6%) compared with 599 of 30,929 mesalamine-treated patients (1.9%) (RD −0.3%, 95% CI −0.5 to −0.1; RR 0.829, 95% CI 0.737–0.933; OR 0.826, 95% CI 0.733–0.931; p = 0.002). In the female cohort, 638 of 33,365 patients without mesalamine (1.9%) versus 693 of 33,416 mesalamine-treated patients (2.1%) experienced TIA (RD −0.2%, 95% CI −0.4 to 0.1; RR 0.922, 95% CI 0.829–1.026; OR 0.921, 95% CI 0.826–1.026; p = 0.135).

*Stroke*
In the male cohort, stroke occurred in 792 of 30,457 patients without mesalamine (2.6%) versus 939 of 30,581 mesalamine-treated patients (3.1%) (RD −0.5%, 95% CI −0.7 to −0.2; RR 0.847, 95% CI 0.772–0.930; OR 0.843, 95% CI 0.766–0.928; p < 0.001). In the female cohort, 947 of 33,078 patients without mesalamine (2.9%) versus 917 of 33,260 mesalamine-treated patients (2.8%) had stroke (RD 0.1%, 95% CI −0.1 to 0.4; RR 1.038, 95% CI 0.949–1.136; OR 1.040, 95% CI 0.948–1.140; p = 0.409).

**Crohn’s Disease**

After 1:1 propensity score matching, the male cohorts comprised 6,007 Crohn’s disease patients without infliximab and 6,007 Crohn’s disease patients treated with infliximab; the female cohorts comprised 7,292 patients without infliximab and 7,292 infliximab-treated patients. Baseline characteristics were well balanced after matching (standardized differences ≤ 0.01), including current age, age at index, and immune suppressants. After matching, mean current age in males was 64.0 years (SD 9.3) in the no-infliximab cohort and 64.1 years (SD 9.3) in the infliximab cohort; in females, mean current age was 63.9 years (SD 9.4) versus 64.0 years (SD 9.4), respectively.

Alzheimer’s Disease
In the male cohort, Alzheimer’s disease occurred in 13 of 5,999 patients without infliximab (0.2%) versus 26 of 5,999 infliximab-treated patients (0.4%), yielding a risk difference of −0.2% (95% CI −0.4 to −0.0), a risk ratio of 0.50 (95% CI 0.257–0.972), and an odds ratio of 0.499 (95% CI 0.256–0.972); p = 0.037. In the female cohort, 39 of 7,281 patients without infliximab (0.5%) versus 25 of 7,285 infliximab-treated patients (0.3%) developed Alzheimer’s disease (RD 0.2%, 95% CI −0.0 to 0.4; RR 1.561, 95% CI 0.946–2.576; OR 1.564, 95% CI 0.945–2.587; p = 0.079).

Parkinson’s Disease
In the male cohort, 43 of 5,971 patients without infliximab (0.7%) and 40 of 5,973 infliximab-treated patients (0.7%) were diagnosed with Parkinson’s disease (RD 0.1%, 95% CI −0.2 to 0.3; RR 1.075, 95% CI 0.700–1.652; OR 1.076, 95% CI 0.698–1.657; p = 0.740). In the female cohort, Parkinson’s disease occurred in 33 of 7,264 patients without infliximab (0.5%) versus 31 of 7,276 infliximab-treated patients (0.4%) (RD 0.0%, 95% CI −0.2 to 0.2; RR 1.066, 95% CI 0.654–1.739; OR 1.067, 95% CI 0.653–1.743; p = 0.797).

Transient Ischemic Attack (TIA)
In the male cohort, TIA was observed in 84 of 5,934 patients without infliximab (1.4%) compared with 109 of 5,932 infliximab-treated patients (1.8%) (RD −0.4%, 95% CI −0.9 to 0.0; RR 0.770, 95% CI 0.581–1.022; OR 0.767, 95% CI 0.576–1.022; p = 0.069). In the female cohort, 140 of 7,192 patients without infliximab (1.9%) versus 112 of 7,191 infliximab-treated patients (1.6%) experienced TIA (RD 0.4%, 95% CI −0.0 to 0.8; RR 1.250, 95% CI 0.977–1.599; OR 1.255, 95% CI 0.977–1.612; p = 0.075).

Stroke
In the male cohort, stroke occurred in 153 of 5,888 patients without infliximab (2.6%) versus 171 of 5,896 infliximab-treated patients (2.9%) (RD −0.3%, 95% CI −0.9 to 0.3; RR 0.896, 95% CI 0.723–1.111; OR 0.893, 95% CI 0.716–1.114; p = 0.317). In the female cohort, 194 of 7,137 patients without infliximimab (2.7%) versus 174 of 7,154 infliximab-treated patients (2.4%) had stroke (RD 0.3%, 95% CI −0.2 to 0.8; RR 1.118, 95% CI 0.913–1.368; OR 1.121, 95% CI 0.911–1.379; p = 0.280).
